# Risk assessment of recent Egyptian H5N1 influenza viruses

**DOI:** 10.1038/srep38388

**Published:** 2016-12-06

**Authors:** A.-S. Arafa, S. Yamada, M. Imai, T. Watanabe, S. Yamayoshi, K. Iwatsuki-Horimoto, M. Kiso, Y. Sakai-Tagawa, M. Ito, T. Imamura, N. Nakajima, K. Takahashi, D. Zhao, K. Oishi, A. Yasuhara, C. A. Macken, G. Zhong, A. P. Hanson, S. Fan, J. Ping, M. Hatta, T. J. S. Lopes, Y. Suzuki, M. El-Husseiny, A. Selim, N. Hagag, M. Soliman, G. Neumann, H. Hasegawa, Y. Kawaoka

**Affiliations:** 1 National Laboratory for Veterinary Quality Control on Poultry Production, Animal Health Research Institute, Dokki, Giza, Egypt; 2 Division of Virology, Department of Microbiology and Immunology, Institute of Medical Science, University of Tokyo, Tokyo 108-8639, Japan; 3 Department of Pathology, National Institute of Infectious Diseases, Sinjuku-ku, Tokyo 162-8640, Japan; 4 Bioinformatics Institute, The University of Auckland, Auckland 1142, New Zealand; 5 Influenza Research Institute, Department of Pathobiological Sciences, School of Veterinary Medicine, University of Wisconsin-Madison, Madison, WI 53711, USA; 6 College of Life and Health Sciences, Chubu University, Aichi 487-8501, Japan; 7 General Organization for Veterinary Services, Dokki, Giza, Egypt; 8 Department of Special Pathogens, International Research Center for Infectious Diseases, Institute of Medical Science, University of Tokyo, Minato-ku, Tokyo 108-8639, Japan

## Abstract

Highly pathogenic avian influenza (HPAI) viruses of the H5N1 subtype are enzootic in poultry populations in different parts of the world, and have caused numerous human infections in recent years, particularly in Egypt. However, no sustained human-to-human transmission of these viruses has yet been reported. We tested nine naturally occurring Egyptian H5N1 viruses (isolated in 2014–2015) in ferrets and found that three of them transmitted via respiratory droplets, causing a fatal infection in one of the exposed animals. All isolates were sensitive to neuraminidase inhibitors. However, these viruses were not transmitted via respiratory droplets in three additional transmission experiments in ferrets. Currently, we do not know if the efficiency of transmission is very low or if subtle differences in experimental parameters contributed to these inconsistent results. Nonetheless, our findings heighten concern regarding the pandemic potential of recent Egyptian H5N1 influenza viruses.

Highly pathogenic avian influenza (HPAI) viruses of the H5N1 subtype are enzootic in poultry populations in different parts of the world, including several Southeast Asian countries and Egypt. Egypt, in particular, has seen numerous human H5N1 virus infections: As of February 25, 2016, 346 of 846 laboratory-confirmed human HPAI H5N1 virus infections have occurred in Egypt, including 173 of the 195 human HPAI H5N1 virus infections reported in 2014–2015 (http://www.who.int/influenza/human_animal_interface/2016_02_25_tableH5N1.pdf?ua=1)^1^. It is unclear whether the high number of human HPAI H5N1 infections in Egypt in 2014–2015 reflects socioeconomic changes resulting in increased contact between people and infected animals or if genetic changes in the virus have increased its predilection for human infections.

The HPAI H5N1 viruses were introduced into Egyptian poultry populations in 2006 as descendants of the Qinghai Lake lineage of H5N1 viruses, which belong to subclade 2.2 of the WHO classification system of HPAI H5N1 influenza viruses. Since then, extensive evolution of these viruses has produced several subclades (Supplementary Fig. S1)^2^,^3^,^4^,^5^,^6^,^7^,^8^. Almost all recent human cases in Egypt have been caused by viruses of subclades 2.2.1 and 2.2.1.2. In early 2015, a novel cluster within clade 2.2.1.2 was reported that contains all recent human isolates and may have replaced previously circulating clade 2.2.1.2 viruses^9^. Given that HPAI H5N1 viruses in Egypt evolve rapidly and have caused a substantial number of human infections, we here characterized the respiratory droplet transmissibility of nine Egyptian HPAI H5N1 influenza viruses in ferrets.

## Results

### Sequence analysis of recent Egyptian HPAI H5N1 viruses

We here characterized nine Egyptian HPAI H5N1 influenza viruses isolated from household poultry in 2014 and 2015 (Supplementary Table S1) for their respiratory droplet transmissibility in ferrets. We, first, established the consensus sequences of all nine isolates by Sanger sequencing. Phylogenetic analysis of the hemagglutinin (HA) gene placed all nine viruses in the novel cluster within subclade 2.2.1.2 (Supplementary Fig. S1). Avian influenza viruses including HPAI H5N1 viruses typically bind to sialic acids linked to galactose by an α2,3-linkage (Siaα2,3 Gal; expressed on epithelial cells of duck intestine)^10^. Our groups^11^ and others^12^,^13^,^14^ previously demonstrated that the ability to bind to sialic acids linked to galactose by an α2,6-linkage (Siaα2,6 Gal; expressed in the upper respiratory epithelia of humans^15^) is necessary for the respiratory droplet transmissibility in ferrets or guinea pigs of genetically modified H5 viruses. Specifically, the HA-N219K/Q221L (all HA amino acid position numbers refer to the reference sequence A/chicken/Egypt/0915-NLQP/2009^16^) or HA-Q221L/G223S^12^ mutations change the receptor-binding specificity of H5 viruses from avian- to human-type. The HA proteins of the Egyptian H5N1 viruses analyzed here encode the avian virus-characteristic N219, Q221, and G223 residues. Viruses of subclades 2.2.1 and 2.2.1.2 possess characteristic D43N, S120N/D, ΔS129 (Δ indicates the deletion of an amino acid compared with the H3 HA reference sequence), and I150T mutations in HA^4^,^17^; the ΔS129/I150T double mutation confers binding to Siaα2,6 Gal while retaining Siaα2,3 Gal binding^18^,^19^,^20^. The viruses analyzed here encode D43N, S120D, ΔS129, and I150T, suggesting that they bind to human-type receptors. Moreover, the viruses tested here lack the glycosylation site at positions 153–155 of HA; the lack of this site is a feature shared by all of the genetically modified mammalian-transmissible H5 viruses reported to date^11^,^12^,^13^,^14^. Highly pathogenic HPAI H5N1 viruses are also characterized by a multibasic cleavage site in HA, which allows cleavage of the HA precursor into the HA1 and HA2 subunits by ubiquitous proteases, thus allowing fatal systemic viral infections in terrestrial avian species. Most subclade 2.2.1 and 2.2.1.1 HA proteins possess a cleavage site of the sequence PQGERRRKKR↓G (‘↓’ denotes the cleavage site); in contrast, subclade 2.2.1.2 HA proteins encode the motif PQGEKRRKKR↓G; currently, it is not known if this difference affects the virulence or pathogenicity of these viruses.

Several mammalian-adapting amino acid changes substantially increase the replicative ability of avian influenza virus polymerase complexes in mammalian cells^21^,^22^,^23^,^24^. The significance of the viral polymerase complex for host adaptation is underscored by the fact that three of the four mammalian-transmissible viruses reported to date were genetically modified to express the mammalian PB2-E627K mutation or possessed a polymerase complex derived from human influenza viruses^11^,^12^,^14^. As descendants of the Qinghai Lake lineage of HPAI H5N1 viruses, all Egyptian H5N1 viruses encode the PB2-E627K mutation. Thus, many Egyptian H5N1 viruses, including the isolates characterized here, possess three molecular features that are now known to be important for genetically modified H5 viruses to transmit in mammals via respiratory droplets^11^,^12^,^13^,^14^: (*i*) an HA that binds to human-type receptors; (*ii*) lack of a *N*-glycosylation site at positions 153–155 of HA, which may facilitate receptor binding; and (*iii*) key amino acid residues in the viral polymerase complex for efficient avian influenza virus replication in mammals cells.

### Ferret transmissibility of recent Egyptian HPAI H5N1 viruses

**Figure 1:**
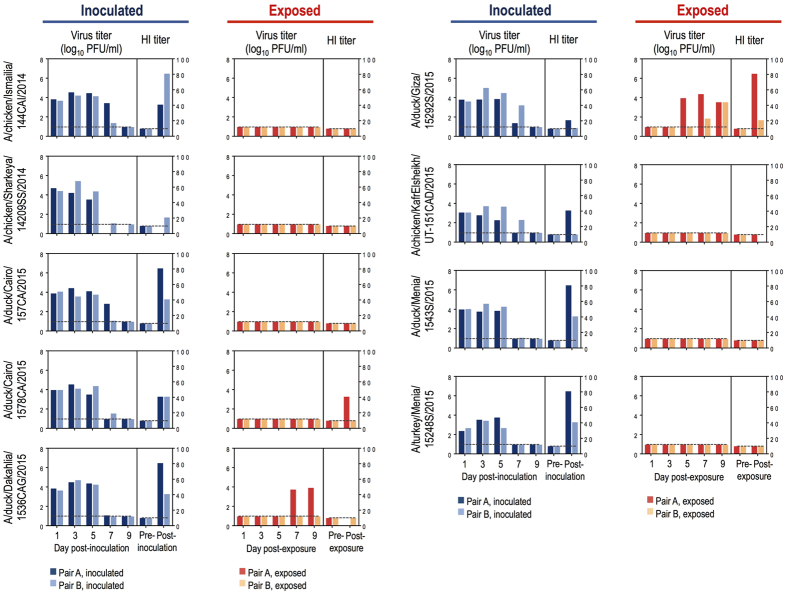
Respiratory droplet transmissibility of Egyptian H5N1 viruses in ferrets. Ferrets were inoculated with the indicated viruses (‘Inoculated’). One day later, one naïve ferret was placed in a cage next to an inoculated ferret (‘Exposed’). Virus titers were determined on the indicated days post-inoculation or -exposure, respectively. HI titers were measured pre-inoculation or -exposure, respectively, and on day 26 post-inoculation or on day 25 post-exposure. Horizontal lines indicate detection limits (i.e., a virus titer of 1.0 log_10_ PFU/ml or an HI titer of 10).

To date, no natural HPAI H5N1 virus has been reported to transmit in mammals via respiratory droplets. However, the profusion of recent human HPAI H5N1 infections in Egypt combined with the potential for these viruses to possess mammalian-adapting markers prompted us to test all nine isolates for respiratory droplet transmissibility in ferrets, the animal most frequently used for influenza virus transmission studies. Such studies with naturally occurring isolates are not subject to the Gain-of-Function Research Pause implemented by the US Government on October 17, 2014 (for additional information on biosafety and biosecurity, see Supplementary Information). We infected ferrets intranasally with 10^6^ plaque-forming units (PFU) of virus stocks generated in embryonated chicken eggs. One day later, naïve ferrets were placed in wireframe cages next to the infected animals. Per virus, two transmission pairs were tested. In this setting, virus transmission can occur via respiratory droplets, but not through direct contact among animals. Nasal wash samples of infected and exposed ferrets were tested for virus titers on day one after infection or exposure, and then every other day (Fig. 1). Generally, virus titers in nasal wash samples of inoculated ferrets were modest (Fig. 1); however, viruses were detected in the brains of infected animals (Supplementary Table S2) and pathological changes were observed (Supplementary Fig. S2). All inoculated animals developed fever and lost body weight, but recovered from virus infection, with the exception of one animal inoculated with A/chicken/Sharkeya/14209SS/2014, which succumbed to its infection on day 6 post-inoculation. Infectious virus and viral antigen were detected in the respiratory organs and brain of the dead animal, but not from any other organs (Supplementary Table S3, Supplementary Fig. S2).

A/duck/Giza/15292 S/2015 (H5N1; Giza) was isolated from both exposed animals, which recovered from the infection (Fig. 1). A/duck/Dakahlia/1536CAG/2015 (H5N1; Dakahlia) was transmitted to one exposed animal, which succumbed to its infection on day 11 post-exposure (Fig. 1). Virus titers in the organs of the dead animal revealed systemic spread with modest titers in the brain (Supplementary Table S4); pathological examination showed viral antigen in the brain, lungs, liver, and spleen (Supplementary Fig. S3). In addition, one animal exposed to a ferret inoculated with A/chicken/KafrElsheikh/UT-151CAD/2015 died on day 13 post-exposure. No virus was isolated and no viral antigen was detected in this animal; hence, the cause of death is uncertain.

Hemagglutination inhibition (HI) and virus neutralization (VN) assays with sera collected on day 26 post-infection demonstrated that all infected animals seroconverted (Fig. 1, Supplementary Table S5), although most HI titers were low. HI and virus neutralizing activity of sera collected on day 25 post-exposure were also detected for both animals exposed to Giza virus, consistent with the isolation of infectious viruses from these animals (note that the animal exposed to Dakahlia virus died before post-exposure serum was collected). In addition, one animal exposed to A/duck/Cairo/1578CA/2015 (H5N1; Cairo) seroconverted, suggesting virus infection (Fig. 1, Supplementary Table S5). Together, these findings suggest that HPAI H5N1 viruses circulating in nature may transmit among mammals via respiratory droplets. In mice, these viruses replicated systematically (Supplementary Table S6), with 50% lethal doses ranging from 5.6–100 PFU (Supplementary Table S6).

### Sequence analysis of H5N1 viruses isolated from exposed animals

To identify amino acid changes that may support respiratory droplet transmissibility among ferrets, we compared the consensus sequences of viruses isolated from infected and exposed animals. Compared with the virus stocks used for infection, a subpopulation of viruses isolated from nasal wash samples of ferrets infected with Dakahlia virus possessed an E60A mutation in NS1, whereas replication of Giza virus did not result in shared amino acid changes in the infected animals (Supplementary Tables S7 and S8). For both viruses, sequence analysis of individual viral plaques revealed additional amino acid changes, none of which were dominant in the viral populations (Supplementary Tables S7 and S8). In viruses isolated from exposed animals, mutations were detected in the polymerase PB2 protein of Dakahlia (PB2-A84T) and Giza (PB2-D146G) virus (Supplementary Tables S9 and S10); both mutations have been detected in natural avian and human influenza A viruses, albeit at low frequency (Supplementary Table S11). Again, individual viral plaques possessed additional amino acid changes that were not dominant in the respective viral populations (Supplementary Tables S9 and S10).

### Mini-replicon assays to assess mutants isolated from exposed animals

To assess the potential significance of the PB2-A84T and PB2-D146G mutations for viral replication, we performed mini-replicon assays in mammalian and avian cells. Human A549 and avian DF-1 cells were transfected with plasmids encoding the components of the Giza virus replication complex (i.e., the polymerase PB2, PB1, and PA proteins, and the nucleoprotein NP, which are all identical at the amino acid level between Giza and Dakahlia viruses), with a plasmid expressing the luciferase reporter protein from an influenza virus-like RNA, and with a control plasmid to normalize transfection efficiencies. The results of two experiments (both carried out in duplicate) were compared using one-way ANOVA, followed by Tukey’s Post-hoc test. Introduction of the A84T mutation into the PB2 proteins of Dakahlia and Giza virus, respectively (which differ by two synonymous nucleotide changes but are identical at the amino acid level), significantly increased the polymerase activity in human cells at 33 °C (the temperature of the upper human respiratory tract) and at 37 °C (Supplementary Fig. S4A), and in avian cells at 39 °C (Supplementary Fig. S4B). The PB2-D146G mutation significantly increased polymerase activity in human cells at 33 °C, but not at 37 °C (Supplementary Fig. S4A). In avian cells at 39 °C, PB2-D146G conferred lower polymerase activity compared with wild-type PB2, but this difference was statistically significant in only two of four experiments (Supplementary Fig. S4B). These findings suggest that the PB2-A84T and PB2-D146G mutations may enhance virus replication in the upper respiratory tract of mammals, which in turn could facilitate respiratory droplet transmission.

### Receptor-binding specificity of recent Egyptian HPAI H5N1 viruses

**Figure 2:**
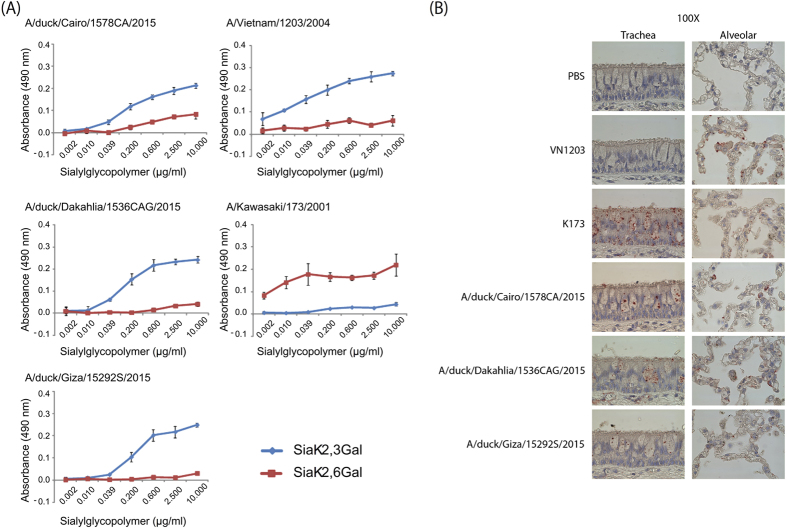
Receptor-binding properties of Egyptian H5N1 and control viruses. (**A**) Binding of viruses to sialylglycopolymers containing either α2,3-linked (blue) or α2,6-linked (red) sialic acids in solid-phase binding assays. Shown are the Dakahlia, Giza, and Cairo viruses, and control viruses expressing the human A/Kawasaki/173/2001 (K173; H1N1) or avian A/Vietnam/1203/2004 (VN1203; H5N1) HA and NA proteins in the background of A/Puerto Rico/8/34 (H1N1) virus. (**B**) Binding of viruses to human respiratory tissues. The viruses described above were labeled with FITC and incubated with human tissue sections. Tissue sections were subsequently incubated with HRP-conjugated, anti-FITC antibodies, and then with a chromogen substrate to detect virus binding. Dark red stain indicates virus binding.

Receptor-binding specificity of influenza viruses is a key determinant of host specificity and interspecies transmission (reviewed in refs 25,26). All inoculum viruses and samples isolated from exposed animals possess amino acids that facilitated the binding of Egyptian HPAI H5N1 viruses to human-type receptors^18^,^19^,^20^. To assess the receptor-binding specificity of the viruses tested here, we performed a solid phase binding assay in which α2,3- or α2,6-linked sialylglycopolymers were coated onto a microtiter

plate and incubated with viruses (Fig. 2A). As expected, viruses possessing the human A/Kawasaki/173/2001 (K173; H1N1) or avian A/Vietnam/1203/2004 (VN1203; H5N1) HA and neuraminidase (NA) proteins bound to α2,6- or α2,3-linked sialylglycopolymers, respectively. All isolates tested bound primarily to α2,3-linked sialylglycopolymers. However, all isolates (with the exception of A/chicken/KafrElsheikh/UT-151CAD/2015, H5N1) bound to chicken red blood cells treated with Siaα2,3-linkage-specific sialidase (New England Biolabs, Beverley, MA), hence leaving predominantly Siaα2,6 Gal (i.e., human-type receptors) (Supplementary Table S12).

To determine the effect of dual receptor-binding specificity on virus attachment to cells of the human respiratory tract, we exposed sections of tracheal and lung tissue to the H5N1 and control viruses (Fig. 2B). All viruses bound to alveolar cells. The avian VN1203 virus did not bind to epithelial cells in the trachea. In contrast, human K173 virus and the Egyptian H5N1 viruses bound to tracheal epithelial cells, although Giza virus bound only weakly (Fig. 2B). These data further support the limited dual receptor-binding specificity of the Egyptian H5N1 viruses characterized in this study.

### Antibody cross-reactivity with H5N1 vaccine viruses

Since HPAI H5N1 viruses first appeared in 1997, inactivated vaccines have been developed. The list of candidate vaccine viruses is continuously updated as novel, antigenically evolved HPAI H5N1 subclades emerge. Some of these HPAI H5N1 vaccine viruses have been approved in certain countries. Most of these vaccines are based on HPAI H5N1 viruses of subclades other than 2.2.1.2, so would they protect against infection with an Egyptian HPAI H5N1 virus? To address this critical question, we obtained sera from individuals vaccinated with inactivated, non-adjuvanted A/Vietnam/1203/2004 (clade 1) or A/Indonesia/05/2005 (clade 2.1.3.2) vaccines and tested them for their virus neutralization activity against the inoculum and transmitted viruses. We detected little-to-no virus neutralization activity (Supplementary Table S13), suggesting that these vaccines would mostly likely not protect against a current Egyptian subclade 2.2.1.2 virus. We also obtained sera from individuals vaccinated with an experimental inactivated, alum-adjuvanted whole-virus vaccine to A/Egypt/N03072/2010 (IDCDC-RG29; clade 2.2.1). Several sera obtained from vaccinated individuals possessed neutralizing activity against Dakahlia or Giza virus or both (Supplementary Tables S14 and S15); overall, antibody titers were relatively low, most likely reflecting the relatively low immunogenicity of H5 vaccines^27^,^28^. Whether this level of neutralizing activity would confer protection against viruses of subclade 2.2.1.2 is unclear.

### Sensitivity to neuraminidase inhibitors

If currently circulating HPAI H5N1 viruses acquire the ability to efficiently transmit among humans, antiviral compounds would be the first line of defense, prompting us to test the neuraminidase inhibitor sensitivity of the inoculated and transmitted viruses (Supplementary Table S16). All Egyptian isolates tested were sensitive to neuraminidase inhibitors, consistent with the absence of known amino acid mutations in NA that confer oseltamivir resistance.

### Additional ferret transmission studies of Egyptian H5N1 influenza viruses

Our data would be the first report on respiratory droplet transmission of natural HPAI H5N1 viruses in ferrets. To confirm this key finding, we performed three additional respiratory droplet transmission studies in ferrets. In study 2, the Giza, Dakahlia, and Cairo viruses were tested in four ferret transmission pairs each (Supplementary Fig. S5A). Due to the limited number of ferrets available, study 3 was performed with four transmission pairs for the Giza and Dakahlia viruses, and the Cairo virus was omitted from this study (Supplementary Fig. S5B). In study 4, all three viruses were tested in six transmission pairs each (Supplementary Fig. S5C). Viruses were isolated from the nasal wash samples of all virus-inoculated animals on days 1, 3, and 5 post-infection (Supplementary Fig. S5A–C), with the exception of one animal infected with Cairo virus (Supplementary Fig. S5A). All inoculated animals also seroconverted, based on HI titers (Supplementary Fig. S5A–C) and/or virus neutralization titers (Supplementary Tables S17 and S18; note that neutralization titers were not obtained in study 4). However, in contrast to our first transmission study, none of the exposed animals seroconverted and no viruses were recovered from these animals (Supplementary Fig. S5A–C).

## Discussion

None of the several highly pathogenic H5N1 viruses found in nature have previously been shown to transmit via respiratory droplets in ferrets or guinea pigs (reviewed in ref. 29). Previously, our groups speculated that Egyptian HPAI H5N1 viruses may have increased pandemic potential because they encode amino acids in HA that enable binding to human-type receptors, encode the PB2-E627K mutation that allows efficient replication in mammals, and lack a glycosylation site at positions 153–155 of HA^30^,^31^. This finding, together with the alarming increase in human HPAI H5N1 virus infections in Egypt in 2014–2015, prompted us to test the respiratory droplet transmissibility of recent Egyptian HPAI H5N1 viruses in ferrets. While our first study demonstrated respiratory droplet transmission among ferrets for three of nine HPAI H5N1 viruses, this finding was not reproducible in three additional transmission experiments. The transmission efficiency of these viruses in ferrets may be very low, such that subtle differences in experimental conditions (such as the air flow, and health of the animals, and/or animal handling) may have affected the outcomes of these studies. Collectively, these data suggest that contemporary Egyptian HPAI H5N1 viruses may possess the ability to transmit among mammals, although not at the level of seasonal human influenza viruses.

## Materials and Methods

### Cells and Tissues

Madin-Darby canine kidney (MDCK) cells and MDCK cells overexpressing Siaα2,6 Gal (MDCK/AX4)^32^,^33^ were maintained in Eagle’s minimal essential medium (MEM) containing 5% newborn calf serum (NCS) and antibiotics. Human epithelial HeLa cells were maintained in MEM containing 10% FBS and antibiotics. Adenocarcinomic human alveolar basal epithelial (A549) cells were maintained in a 1:1 mixture of Dulbecco’s modified essential medium (DMEM) and Ham’s F12 (DMEM/F12, Gibco) medium with 10% fetal calf serum (FCS) and antibiotics. Chicken fibroblast (DF-1) cells were grown in Dulbecco’s modified essential medium (DMEM) with 10% FCS and antibiotics. Cells were maintained at 37 °C or 39 °C (DF-1) in 5% CO_2_. Paraffin-embedded tissue sections of the human trachea and lung were purchased from US Biomax.

### Viruses

Oropharyngeal swabs were collected from household poultry as part of routine and targeted surveillance activities in different localities of Egypt (Supplementary Table S1). Swabs were placed in transport medium [phosphate-buffered saline with antibiotics], transported to the laboratory on ice, and stored at −80 °C. All samples were amplified in embryonated chicken eggs, and virus stock titers were determined by means of plaque assays in MDCK cells. Virus stocks were stored at −80 °C. The full genomic sequences of the amplified surveillance samples and viruses isolated from exposed animals were determined by Sanger sequencing. The sequence data were submitted to Genbank (Accession numbers LC106039 - LC106110).

Human and avian control viruses used in this study included A/Kawasaki/173/2001 (K173, H1N1)^11^, A/Vietnam/1203/2004 (VN1203, H5N1)^11^, A/California/04/2009 (H1N1)^34^, A/Kawasaki/UTK-4/2009 (H1N1)^34^, and A/Kawasaki/UTK-23/2008 (H1N1)^34^.

### Plaque Assay

Viruses were diluted 10-fold in MEM containing 0.3% BSA. Confluent monolayers of MDCK cells were washed with MEM containing 0.3% BSA, infected with diluted viruses, and incubated for 30–60 min at 37 °C. After the virus inoculum was removed, the cells were washed with MEM containing 0.3% BSA and overlayed with a 1:1 mixture of 2x MEM/0.6% BSA and 2% agarose containing 0.5–1 μg/ml Tosylsulfonyl Phenylalanyl Chloromethyl Ketone (TPCK)-trypsin. Plates were incubated at 37 °C for 48–72 h before virus plaques were counted; titers were calculated by using the method of Reed and Munch^35^.

### Hemagglutination (HA) Assay

Viruses (50 μl) were serially diluted two-fold with 50 μl of PBS in a U-well microtiter plate. Fifty microliters of 0.5% (vol/vol) of chicken red blood cells (CRBC) or turkey red blood cells (TRBCs) were added to each well. The plates were incubated at room temperature and hemagglutination was evaluated after 45 min. The HA titers were calculated as the highest dilution at which complete agglutination was observed.

### Hemagglutination Inhibition (HI) Assay

To detect hemagglutination inhibition activity, serum samples were treated with receptor-destroying enzyme (RDE; Denka Seiken Co., Ltd) at 37 °C for 16–20 h, followed by RDE inactivation at 56 °C for 30–60 min. The RDE-treated sera were then serially diluted two-fold in PBS, mixed with an amount of virus equivalent to eight hemagglutination units, and incubated at room temperature for 30–60 min. After the addition of 50 μl of 0.5% (vol/vol) CRBC or TRBC, the resulting mixture was gently mixed and incubated at 4 °C or room temperature for 30–45 min. HI titers were recorded as the inverse of the highest antibody dilution that inhibited 8 HA units of virus.

### Virus Neutralization Assay

Viral neutralization assays were performed by using the methodology outlined in the *WHO Manual on Animal Influenza Diagnosis and Surveillance* with the following modifications. Briefly, sera were treated with RDE at 37 °C for 18–20 h, followed by RDE inactivation at 56 °C for 30–60 min. Fifty microliters of virus (100 tissue culture infectious dose 50) was incubated with 50 μl of two-fold serial dilutions of RDE-treated sera for 30 min at 37 °C, and the mixtures were added to confluent MDCK cells in 96-well microplates, and incubated for 1 h at 37 °C. After the inoculum was removed, the cells were incubated with MEM containing 0.3% BSA and 0.75 μg/ml TPCK-trypsin at 37 °C for 48–72 h. Viral cytopathic effects were observed under an inverted microscope and virus neutralization titers were calculated as described in the WHO manual.

### Serological Tests

The following human serum samples were obtained from the NIH Biodefense and Emerging Infections Research Resources Repository (BEI Resources), NIAID, NIH: Polyclonal anti-monovalent influenza subvirion vaccine rgA/Vietnam/1203/2004 (H5N1), low titer pool, NR-4110, and high titer pool, NR-4109; and human reference antiserum to influenza A/Indonesia/05/2005 (H5N1), low titer, NR-33667, medium titer, NR-33668, and high titer, NR-33669. Human serum samples were also collected from volunteers who had received the alum-adjuvanted, inactivated whole H5N1 virus vaccine A/Egypt/N03072/2010 (H5N1; IDCDC-RG 29) under a research protocol approved by the Research Ethics Review Committee of the Institute of Medical Science, University of Tokyo (approval number 25-58-1205). Informed consent was obtained from all subjects. All methods were carried out in accordance with the “Ethical Guidelines for Medical and Health Research Involving Human Subjects” from Ministry of Education, Culture, Sports, Science and Technology, Japan. Samples were treated with RDE, heat-inactivated, and tested in HI and viral neutralization assays. The viruses indicated in Supplementary Tables S13 and S14 served as antigens.

### Mini-Replicon Assay

Human A549 and avian DF-1 cells were transfected with plasmids for the expression of Giza virus PB2, PB1, PA, and NP proteins. To test the PB2-A84T and -D146G mutations, the wild-type PB2 protein expression plasmid was substituted with plasmids expressing Dakahlia PB2-A84T or Giza PB2-D146G, respectively (note that the Dakahlia and Giza PB2 genes differ by two synonymous nucleotide replacements, but encode identical PB2 proteins). Cells were also transfected with pPol-I-NP(0)Luc2(0) (for A549 cells) or pPol-IGG-NP(0)Fluc(0) (for DF-1 cells), which express the firefly luciferase reporter protein from a virus-like RNA transcribed by the human or avian polymerase I promoter, respectively. Plasmid pRL-TK (Promega, Madison, WI) served as an internal control for the dual-luciferase assay. Transfected cells were incubated for 24 h at 33 °C and 37 °C (human A549 cells), or at 39 °C (avian DF-1 cells). Luciferase activity was measured by using the Dual-Glo luciferase assay system (Promega) on a Glomax microplate luminometer (Promega) according to the manufacturer’s instructions. The results of two experiments (both carried out in duplicate) were compared using one-way ANOVA, followed by Tukey’s Post-hoc test. We considered the results significant at *p* < 0.05.

### Solid-Phase Binding Assay

Viruses were amplified in MDCK cells. Virus-containing supernatant was centrifuged at low speed for 15 min to remove cell debris, and then laid over a cushion of 30% sucrose in PBS. After ultracentrifugation at 96,174 × *g* for 90 min at 4 °C, the virus pellet was resuspended in PBS containing glycerol and stored at −80 °C. The concentration of the viruses was determined by means of HA assays with 0.5% (vol/vol) chicken red blood cells.

For the binding assay, microtiter plates (Nunc) were incubated with the sodium salts of sialylglycopolymers [poly-L-glutamic acid backbones containing *N*-acetylneuraminic acid linked to galactose through either an α2,3 (Neu5Acα2,3Galβ1,4GlcNAcβ1-*p*AP) or an α2,6 (Neu5Acα2,6Galβ1,4GlcNAcβ1-*p*AP) bond]^36^ in PBS at 4 °C overnight. After removing the glycopolymers, we blocked the plates with 150 μl of PBS containing 4% BSA at room temperature for 1 h, and then washed them four times with cold PBS. Influenza virus equivalent to 32 HA units (in PBS) was then added to the plates and incubated overnight at 4 °C. After washing the plates as described above, we incubated them for 2 h at 4 °C with rabbit polyclonal anti-H5N1 (VN1203) or ferret polyclonal anti-H1N1 (K173) antibodies, respectively. After additional washes as described above, the plates were incubated with horseradish peroxidase (HRP)-conjugated goat anti-rabbit IgG (Zymed) antiserum or anti-ferret IgG (Bethyl Laboratories) for 2 h at 4 °C. After several additional washes, the plates were incubated with *O*-phenylenediamine (Sigma) in citrate-phosphate buffer containing 0.03% H_2_O_2_ for 10 min at room temperature. The reaction was stopped with 50 μl of 1 M HCl, and absorbance was determined at 490 nm by using an optical plate reader (Mark microplate reader model 680; BioRad).

### Tissue binding assay

Viruses were amplified in MDCK/AX4 cells. Virus-containing supernatants were collected from infected cells and centrifuged at 1,462 × *g* for 15 min to remove cell debris. Viruses were inactivated by incubating them with 0.1% β-propiolactone (final concentration) for at least 16 h at 4 °C. Virus supernatant was laid over a cushion of 30% sucrose in PBS and ultracentrifuged at 96,174 × *g* for 90 min at 4 °C; virus pellet was resuspended in PBS and stored at −80 °C. Virus concentrations were determined by using hemagglutination assays with 0.5% (vol/vol) TRBCs. To assess tissue binding, we deparaffinized and rehydrated paraffin-embedded normal human trachea and lung (US Biomax). The rehydrated sections were blocked with carbo-free blocking solution (Vector) and TNB blocking buffer (Perkin Elmer). Next, we added the equivalent of 8 HA units of fluorescein-5-isothiocyanate (FITC)-labeled virus to the tissue sections and incubated them at 4 °C overnight. The slides were washed five times with cold PBS, and subsequently incubated with an HRP-conjugated rabbit anti-FITC antibody (Dako) for 30 min at room temperature. After additional washes, the slides were incubated with AEC (3-amino-9-ethyl-carbozole) substrate-chromogen (Dako) for 15 min at room temperature. The slides were then rinsed with water, counterstained with Mayer’s hematoxylin (Sigma Aldrich) for 3 min at room temperature, and rinsed again with water. Finally, coverslips were mounted by using Shandon Immu-Mount (Thermo Scientific). We examined all tissue sections by using a high resolution camera (AxioCam HRc) mounted on a microscope (Zeiss, Axio Imager. A2).

### Experimental infection of ferrets

Six-month-old female ferrets (Triple F Farms), which were serologically negative by HI assay for currently circulating human influenza viruses, were used in this study. Under anaesthesia, six ferrets per group were intranasally inoculated with 10^6^ PFU (0.5 ml) of A/duck/Giza/15292 S/2015 (H5N1), A/duck/Dakahlia/1536CAG/2015 (H5N1), or A/duck/Cairo/1578CA/2015 (H5N1) virus. Three ferrets per group were euthanized on days 3 and 6 post-infection for virological and pathological examinations. The virus titres in various organs were determined by use of plaque assays in MDCK cells.

All experiments with ferrets were performed in accordance with the Science Council of Japan’s Guidelines for Proper Conduct of Animal Experiments and guidelines set by the Institutional Animal Care and Use Committee at the University of Wisconsin-Madison. The protocol was approved by the Animal Experiment Committee of the Institute of Medical Science, the University of Tokyo (approval number PA15-02) and the Animal Care and Use Committee of the University of Wisconsin-Madison (protocol number V00806). For more information, see ‘**Biosafety Statement**’.

### Ferret Transmission Study

Six-month-old female ferrets (Triple F Farms) were first serologically tested for exposure to currently circulating human influenza viruses by using the HI assay. Two-to-six ferrets per transmission group were anaesthetized intramuscularly with ketamine and xylazine (5–30 mg and 0.2–6 mg/kg of body weight, respectively) in the studies carried out at University of Tokyo (i.e., studies 1–3), or with ketamine and dexmedetomidine (4–5 mg/kg and 0.01–0.04 mg/kg, respectively) in the study carried out at the University of Madison (i.e., study 4); animals were then inoculated intranasally with 10^6^ PFU (500 μl) of virus. For ferrets anesthetized with ketamine and dexmedetomidine, atipamezole was used to shorten recovery time from anesthesia. The infected ferrets were housed in transmission cages that prevent direct and indirect contact between animals but allow spread of influenza virus through the air (Showa Science). Twenty-four hours later, one naïve ferret was placed in a cage adjacent to each inoculated ferret (exposed ferrets). All animals were assessed daily for clinical signs and symptoms and changes in body weight. Nasal washes were collected from infected and exposed animals on day 1 after infection or exposure, respectively, and then every other day. Virus titers in nasal washes were determined by means of plaque assays.

### Mouse Virulence Studies

To determine the dose required to kill 50% of infected mice (MLD_50_), six-week-old female BALB/c mice (Jackson Laboratory, Bar Harbor, ME) (3 mice/group) were anesthetized with isoflurane and inoculated intranasally with 10-fold serially diluted virus (from 10^−1^ to 10^5^ PFU) in a 50-μl volume. Changes in body weight and mortality were recorded daily for 14 days. Mice were euthanized if they lost more than 25% of their initial body weight The MLD_50_ was calculated by using the method of Reed and Muench^35^. To determine viral titers in the organs of infected animals, 6 mice/group were infected intranasally with 10^3^ PFU of virus. Three mice in each group were euthanized on days 3 and 6 post-infection, respectively. Lungs, nasal turbinates, kidneys, spleens, and brains were collected for virus titration by use of plaque assays in MDCK cells. The data shown are the mean virus titers ± standard deviation.

All experiments with mice were performed in accordance with the guidelines set by the Institutional Animal Care and Use Committee of the University of Wisconsin-Madison, which also approved the protocol (protocol number V00806).

### Pathological Examination

Excised tissues of animal organs were preserved in 10% phosphate-buffered formalin, processed for paraffin embedding, and cut into 3-μm-thick sections. One section from each tissue sample was stained using a standard hematoxylin-and-eosin procedure; another one was stained with a mouse monoclonal antibody for type A influenza virus nucleoprotein (NP) antigen (prepared in our laboratory) that reacts comparably with all of the viruses tested in this study. Specific antigen–antibody reactions were visualized with 3,3′-diaminobenzidine tetrahydrochloride staining by using the DAKO Envision detection system (DAKO Cytomation, Copenhagen, Denmark).

### Neuraminidase Inhibition Assay

Diluted viruses were mixed with different concentrations of oseltamivir carboxylate (the active form of oseltamivir), zanamivir, laninamivir, or peramivir. Samples were incubated for 30 min at 37 °C, followed by the addition of methylumbelliferyl-*N*-acetylneuraminic acid (Sigma, St Louis, MO) as a fluorescent substrate. After incubation for 1 h at 37 °C, the reaction was stopped with the addition of sodium hydroxide in 80% ethanol. The fluorescence of the solution was measured at an excitation wavelength of 360 nm and an emission wavelength of 465 nm, and the 50% inhibitory concentration (IC_50_) was calculated.

### Phylogenetic Analysis

HA gene sequences of highly pathogenic H5Nx influenza viruses of the goose/Guangdong lineage were downloaded from GISAID (Supplementary Table S19) and Genbank during the week of November 18, 2015; sequences from laboratory mutants were excluded. We required that the sequence included at least 75% of the HA coding region. This selection process resulted in a dataset of 5,848 sequences, including the HA sequence from A/chicken/Scotland/1959 for an out-group. Sequences were aligned using CLUSTALW^37^, then edited manually as needed. RAxML^38^ on CIPRES^39^ was used to infer a phylogeny for the final dataset, using the GTR model of evolution with a gamma model of rate heterogeneity; 100 bootstrap replicates under this model were used to assess topological certainty. The final tree was displayed in Archaeopteryx^40^.

### Biosafety Statement

Because this study involved the characterization of natural influenza viruses, it does not fall under the pause on gain-of-function research announced by the US Government on October 17, 2014.

All experiments were approved by the respective committees at the University of Tokyo and by the University of Wisconsin-Madison’s Institutional Biosafety Committee (IBC). When virus transmission was detected, the University of Wisconsin’s Alternate Responsible Official (ARO) and Institutional Contact for Dual Use Research (ICDUR) was contacted and a risk assessment was performed. All practices and procedures used for additional experiments followed the requirements of the *NIH Guidelines for Research Involving Recombinant or Synthetic Nucleic Acid Molecules* for working with mammalian-transmissible H5N1 viruses. The ARO/ICDUR was kept informed of the research results. This manuscript was reviewed by the University of Wisconsin-Madison Dual Use Research of Concern (DURC) Subcommittee in accordance with the United States Government September 2014 DURC Policy, which concluded that the studies described herein do not constitute DURC since the natural virus isolates were not modified or sequentially passed in our laboratory. In addition, the University of Wisconsin-Madison Biosecurity Task Force regularly reviews the research, policies, and practices of research conducted with pathogens of high consequence at the institution. This task force has a diverse skill set and provides support in the areas of biosafety, facilities, compliance, security, law, and health. Members of the Biosecurity Task Force are in frequent contact with the principal investigator and laboratory personnel to provide oversight and assure biosecurity.

All experiments with HPAI H5N1 viruses were performed in enhanced biosafety level 3 laboratories at the University of Tokyo (Tokyo, Japan), which are approved for such use by the Ministry of Agriculture, Forestry and Fisheries, Japan, or in biosafety level 3 agricultural (BSL-3Ag) laboratories at the University of Wisconsin-Madison approved for such use by the Centers for Disease Control and Prevention (CDC) and Animal and Plant Health Inspection Service (APHIS). Ferret transmission studies were conducted in enhanced BSL-3 containment at the University of Tokyo by PhD-level scientists who are highly experienced in such studies. Mouse virulence studies were conducted in BSL-3Ag at the University of Wisconsin-Madison, also by scientists who have several years of experience working with highly pathogenic influenza viruses and performing animal studies with such viruses. *In vitro* experiments were conducted in Class II biological safety cabinets and transmission experiments were conducted in HEPA-filtered ferret isolators. Staff working in enhanced BSL-3 and BSL-3Ag wear disposable overalls and powered air-purifying respirators. The enhanced BSL-3 facility at the University of Tokyo includes controlled access, exit through a shower change room, effluent decontamination, negative air-pressure, double-door autoclaves, HEPA-filtered supply and exhaust air, and airtight dampers on ductwork connected to the animal cage isolators and biosafety cabinets. The structure is pressure-decay tested regularly. All personnel complete biosafety and BSL-3 training before participating in BSL-3-level experiments. Refresher training is scheduled on a regular basis. Select Agent virus inventory, secured behind two physical barriers, is checked regularly. Virus inventory is submitted once a year to the Ministry of Agriculture, Forestry and Fisheries, Japan

The BSL-3Ag facility at University of Wisconsin-Madison was designed to exceed the standards outlined in *Biosafety in Microbiological and Biomedical Laboratories* (5th edition; http://www.cdc.gov/biosafety/publications/bmbl5/BMBL.pdf). Features include controlled access, entry/exit through a shower change room, effluent decontamination, negative air-pressure, double-door autoclaves, gas decontamination ports, HEPA-filtered supply and double-HEPA-filtered exhaust air, double-gasketed watertight and airtight seals, and airtight dampers on all ductwork. The structure is pressure-decay tested regularly. The University of Wisconsin-Madison facility has a dedicated alarm system that monitors all building controls (~500 possible alerts). Redundancies and emergency resources are built into the facility, including two air handlers, two compressors, two filters wherever filters are needed, two effluent sterilization tanks, two power feeds to the building, an emergency generator in case of a power failure, and other physical containment measures in the facility that operate without power. Biosecurity monitoring of the facility is ongoing. All personnel undergo Select Agent security risk assessment by the United States Criminal Justice Information Services Division and complete rigorous biosafety, BSL-3, and Select Agent training before participating in BSL-3-level experiments. Refresher training, including drills and review of emergency plans, is scheduled on a regular basis. The principal investigator participates in training sessions and emphasizes compliance to maintain safe operations and a responsible research environment. The laboratory occupational health plan is in compliance with the University of Wisconsin-Madison Occupational Health Program. Select Agent virus inventory, secured behind two physical barriers, is checked monthly and documentation is submitted to the University of Wisconsin-Madison Select Agent Program Manager. Virus inventory is submitted 1–2 times per year to the file holder in the Division of Select Agents and Toxins at the CDC. The research program, procedures, occupational health plan, documentation, security, and facilities are reviewed annually by the University of Wisconsin-Madison Responsible Official and at regular intervals by the CDC and the APHIS as part of the University of Wisconsin-Madison Select Agent Program.

## Additional Information

**How to cite this article**: Arafa, A.-S. *et al*. Risk assessment of recent Egyptian H5N1 influenza viruses. *Sci. Rep.* **6**, 38388; doi: 10.1038/srep38388 (2016).

**Publisher's note:** Springer Nature remains neutral with regard to jurisdictional claims in published maps and institutional affiliations.

## Supplementary Material

Supplementary Information
