# Supplementary material for: Risk assessment of recent Egyptian H5N1 influenza viruses
Source: Sci Rep. 2016 Dec 6;6:38388. doi: 10.1038/srep38388 (PMC5138598; doi:10.1038/srep38388)
Supplement: Supplementary Information [file srep38388-s1.pdf]

Supplementary Materials for

Risk assessment of recent Egyptian H5N1 influenza viruses

A.-S. Arafa[1†], S. Yamada[2†], M. Imai[2†], T. Watanabe[2†], S. Yamayoshi[2†], K. Iwatsuki-Horimoto[2†], M. Kiso[2†], Y. Sakai-Tagawa[2†], M. Ito[2†], T. Imamura[2], N. Nakajima[3], K. Takahashi[3], D. Zhao[2], K. Oishi[2], A. Yasuhara[2], C. A. Macken[4], G. Zhong[5], A. P. Hanson[5], S. Fan[5], J. Ping[5], M. Hatta[5], TJS. Lopes[5], Y. Suzuki[6], M. El-Husseiny[1], A. Selim[1], N. Hagag[1], M. Soliman[7] G. Neumann[5], H. Hasegawa[3], Y. Kawaoka[2,5,8]*

[1]National Laboratory for Veterinary Quality Control on Poultry Production, Animal Health Research Institute, Dokki, Giza, Egypt.

[2]Division of Virology, Department of Microbiology and Immunology, Institute of Medical Science, University of Tokyo, Tokyo 108-8639, Japan.

[3]Department of Pathology, National Institute of Infectious Diseases, Sinjuku-ku, Tokyo 162-8640, Japan.

[4]Bioinformatics Institute, The University of Auckland, Auckland 1142, New Zealand.

[5]Influenza Research Institute, Department of Pathobiological Sciences, School of Veterinary Medicine, University of Wisconsin-Madison, Madison, WI 53711, USA.

[6]College of Life and Health Sciences, Chubu University, Aichi 487-8501, Japan.

[7]General Organization for Veterinary Services, Dokki, Giza, Egypt.

[8]Department of Special Pathogens, International Research Center for Infectious Diseases, Institute of Medical Science, University of Tokyo, Minato-ku, Tokyo 108-8639, Japan.

†These authors contributed equally.

*Corresponding author: Yoshihiro Kawaoka, kawaokay@svm.vetmed.wisc.edu

Supplementary Figures

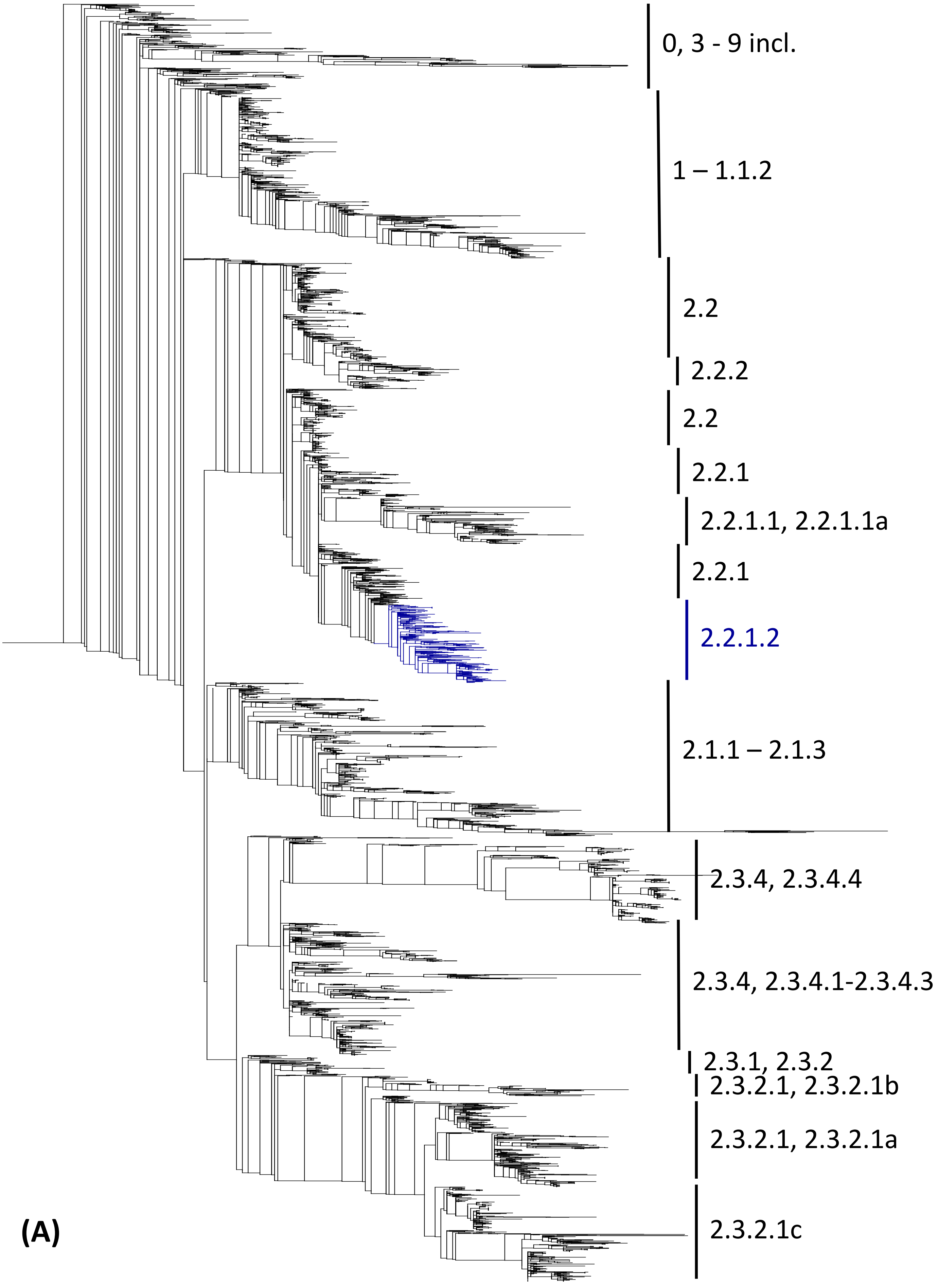
0, 3 - 9 incl.
1 – 1.1.2
2.2
2.2.2
2.2
2.2.1
2.2.1.1, 2.2.1.1a
2.2.1
2.2.1.2
2.1.1 – 2.1.3
2.3.4, 2.3.4.4
2.3.4, 2.3.4.1-2.3.4.3
2.3.1, 2.3.2
2.3.2.1, 2.3.2.1b
2.3.2.1, 2.3.2.1a
2.3.2.1c
(A)

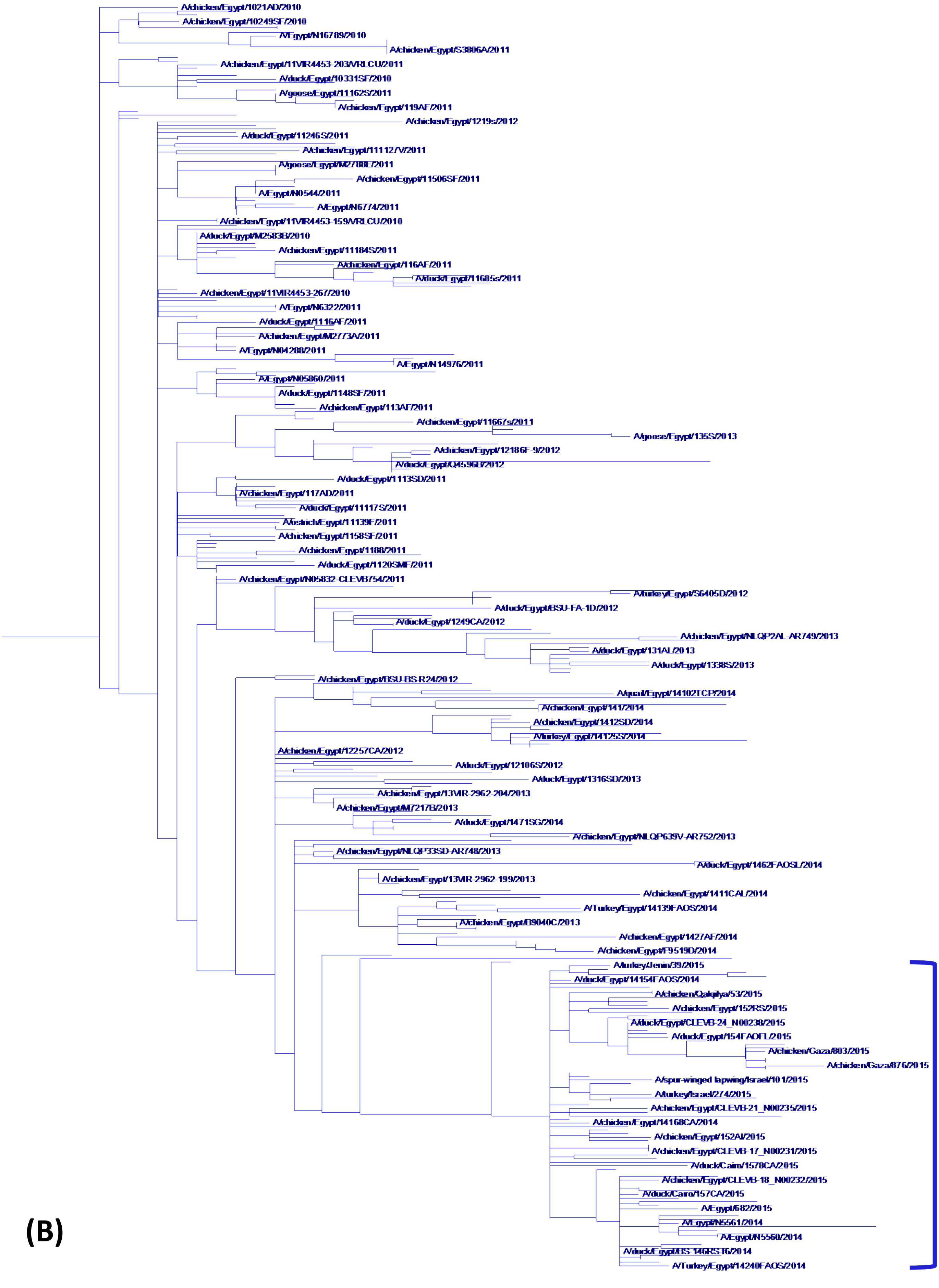

A/chicken/Egypt/1021AD/2010
A/chicken/Egypt/10249SF/2010
A/Egypt/N16789/2010
A/chicken/Egypt/S3806A/2011
A/chicken/Egypt/11VIR4453-203/VRLCU/2011
A/duck/Egypt/10331SF/2010
A/goose/Egypt/11162S/2011
A/chicken/Egypt/119AF/2011
A/chicken/Egypt/1219s/2012
A/duck/Egypt/11246S/2011
A/chicken/Egypt/111127V/2011
A/goose/Egypt/M2788E/2011
A/chicken/Egypt/11506SF/2011
A/Egypt/N0544/2011
A/Egypt/N6774/2011
A/chicken/Egypt/11VIR4453-159/VRLCU/2010
A/duck/Egypt/M2583B/2010
A/chicken/Egypt/11184S/2011
A/chicken/Egypt/116AF/2011
A/duck/Egypt/11685s/2011
A/chicken/Egypt/11VIR4453-267/2010
A/Egypt/N6322/2011
A/duck/Egypt/1116AF/2011
A/chicken/Egypt/M2773A/2011
A/Egypt/N04288/2011
A/Egypt/N14976/2011
A/Egypt/N05860/2011
A/duck/Egypt/1148SF/2011
A/chicken/Egypt/113AF/2011
A/chicken/Egypt/11667s/2011
A/goose/Egypt/135S/2013
A/chicken/Egypt/12186F-9/2012
A/duck/Egypt/Q4596B/2012
A/duck/Egypt/1113SD/2011
A/chicken/Egypt/117AD/2011
A/duck/Egypt/11117S/2011
A/ostrich/Egypt/11139F/2011
A/chicken/Egypt/1158SF/2011
A/chicken/Egypt/1188/2011
A/duck/Egypt/1120SMF/2011
A/chicken/Egypt/N05832-CLEVB754/2011
A/turkey/Egypt/S6405D/2012
A/duck/Egypt/BSU-FA-1D/2012
A/duck/Egypt/1249CA/2012
A/chicken/Egypt/NLQP2AL-AR749/2013
A/duck/Egypt/131AL/2013
A/duck/Egypt/1338S/2013
A/chicken/Egypt/BSU-BS-R24/2012
A/quail/Egypt/14102TCP/2014
A/chicken/Egypt/141/2014
A/chicken/Egypt/1412SD/2014
A/turkey/Egypt/14125S/2014
A/chicken/Egypt/12257CA/2012
A/duck/Egypt/12106S/2012
A/duck/Egypt/1316SD/2013
A/chicken/Egypt/13VIR-2962-204/2013
A/chicken/Egypt/M7217B/2013
A/duck/Egypt/1471SG/2014
A/chicken/Egypt/NLQP639V-AR752/2013
A/chicken/Egypt/NLQP33SD-AR748/2013
A/duck/Egypt/1462FAOSL/2014
A/chicken/Egypt/13VIR-2962-199/2013
A/chicken/Egypt/1411CAL/2014
A/Turkey/Egypt/14139FAOS/2014
A/chicken/Egypt/B9040C/2013
A/chicken/Egypt/1427AF/2014
A/chicken/Egypt/F9519D/2014
A/turkey/Jenin/39/2015
A/duck/Egypt/14154FAOS/2014
A/chicken/Qalqilya/53/2015
A/chicken/Egypt/152RS/2015
A/duck/Egypt/CLEVB-24_N00238/2015
A/duck/Egypt/154FAOFL/2015
A/chicken/Gaza/803/2015
A/chicken/Gaza/876/2015
A/spur-winged lapwing/Israel/101/2015
A/turkey/Israel/274/2015
A/chicken/Egypt/CLEVB-21_N00235/2015
A/chicken/Egypt/14168CA/2014
A/chicken/Egypt/152A/2015
A/chicken/Egypt/CLEVB-17_N00231/2015
A/duck/Cairo/1578CA/2015
A/chicken/Egypt/CLEVB-18_N00232/2015
A/duck/Cairo/157CA/2015
A/Egypt/682/2015
A/Egypt/N5561/2014
A/Egypt/N5560/2014
A/duck/Egypt/BS-146RS-T6/2014
A/Turkey/Egypt/14240FAOS/2014

(B)

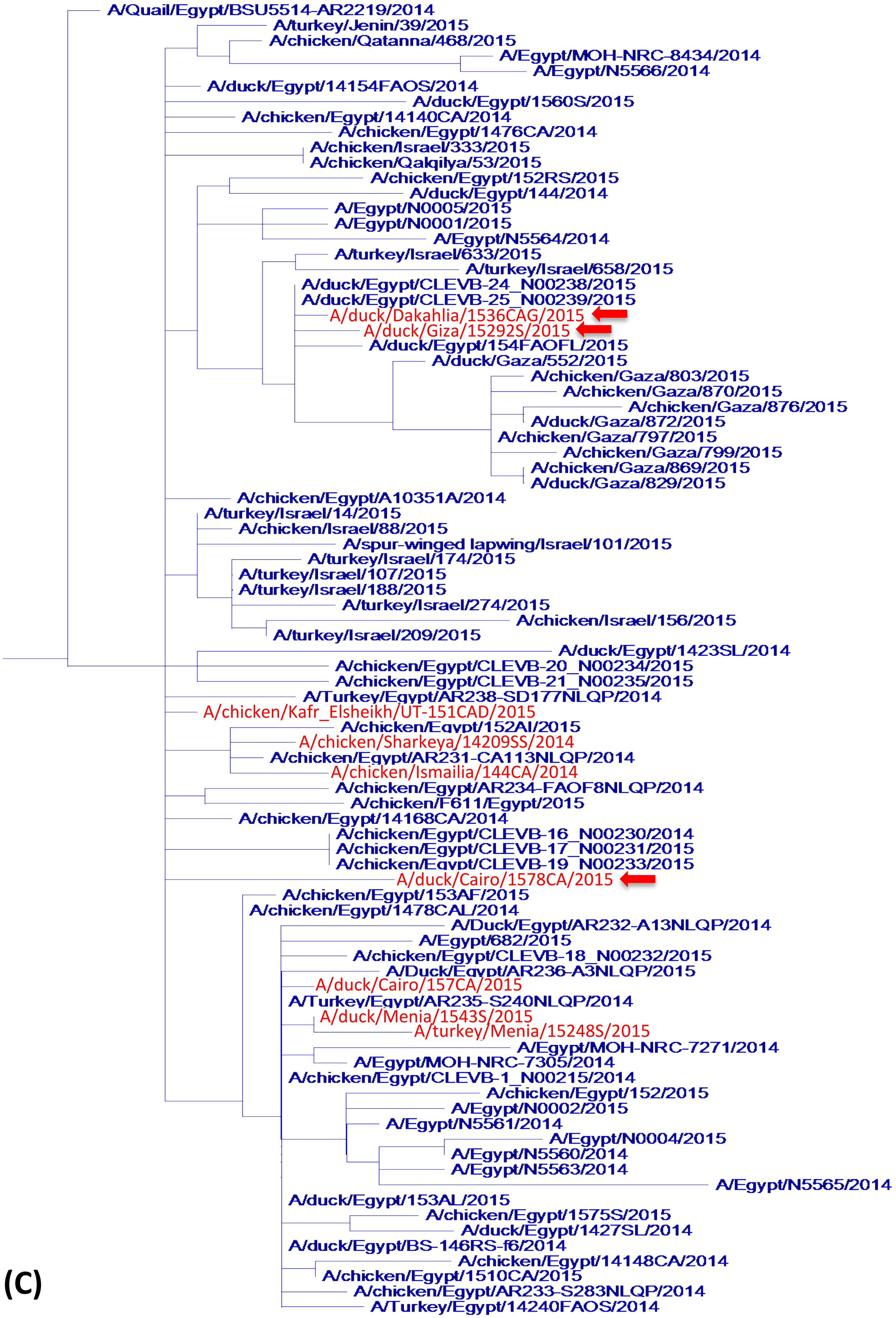
A/Quail/Egypt/BSU5514-AR2219/2014
A/turkey/Jenin/39/2015
A/chicken/Qatanna/468/2015
A/Egypt/MOH-NRC-8434/2014
A/Egypt/N5566/2014
A/duck/Egypt/14154FAOS/2014
A/duck/Egypt/1560S/2015
A/chicken/Egypt/14140CA/2014
A/chicken/Egypt/1476CA/2014
A/chicken/Israel/333/2015
A/chicken/Qalqilya/53/2015
A/chicken/Egypt/152RS/2015
A/duck/Egypt/144/2014
A/Egypt/N0005/2015
A/Egypt/N0001/2015
A/Egypt/N5564/2014
A/turkey/Israel/633/2015
A/turkey/Israel/658/2015
A/duck/Egypt/CLEVB-24_N00238/2015
A/duck/Egypt/CLEVB-25_N00239/2015
A/duck/Dakahlia/1536CAG/2015
A/duck/Giza/15292S/2015
A/duck/Egypt/154FAOFL/2015
A/duck/Gaza/552/2015
A/chicken/Gaza/803/2015
A/chicken/Gaza/870/2015
A/chicken/Gaza/876/2015
A/duck/Gaza/872/2015
A/chicken/Gaza/797/2015
A/chicken/Gaza/799/2015
A/chicken/Gaza/869/2015
A/duck/Gaza/829/2015
A/chicken/Egypt/A10351A/2014
A/turkey/Israel/14/2015
A/chicken/Israel/88/2015
A/spur-winged lapwing/Israel/101/2015
A/turkey/Israel/174/2015
A/turkey/Israel/107/2015
A/turkey/Israel/188/2015
A/turkey/Israel/274/2015
A/chicken/Israel/156/2015
A/turkey/Israel/209/2015
A/duck/Egypt/1423SL/2014
A/chicken/Egypt/CLEVB-20_N00234/2015
A/chicken/Egypt/CLEVB-21_N00235/2015
A/Turkey/Egypt/AR238-SD177NLQP/2014
A/chicken/Kafr_Elsheikh/UT-151CAD/2015
A/chicken/Egypt/152AI/2015
A/chicken/Sharkeya/14209SS/2014
A/chicken/Egypt/AR231-CA113NLQP/2014
A/chicken/Ismailia/144CA/2014
A/chicken/Egypt/AR234-FAOF8NLQP/2014
A/chicken/F611/Egypt/2015
A/chicken/Egypt/14168CA/2014
A/chicken/Egypt/CLEVB-16_N00230/2014
A/chicken/Egypt/CLEVB-17_N00231/2015
A/chicken/Egypt/CLEVB-19_N00233/2015
A/duck/Cairo/1578CA/2015
A/chicken/Egypt/153AF/2015
A/chicken/Egypt/1478CAL/2014
A/Duck/Egypt/AR232-A13NLQP/2014
A/Egypt/682/2015
A/chicken/Egypt/CLEVB-18_N00232/2015
A/Duck/Egypt/AR236-A3NLQP/2015
A/duck/Cairo/157CA/2015
A/Turkey/Egypt/AR235-S240NLQP/2014
A/duck/Menia/1543S/2015
A/turkey/Menia/15248S/2015
A/Egypt/MOH-NRC-7271/2014
A/Egypt/MOH-NRC-7305/2014
A/chicken/Egypt/CLEVB-1_N00215/2014
A/chicken/Egypt/152/2015
A/Egypt/N0002/2015
A/Egypt/N5561/2014
A/Egypt/N0004/2015
A/Egypt/N5560/2014
A/Egypt/N5563/2014
A/Egypt/N5565/2014
A/duck/Egypt/153AL/2015
A/chicken/Egypt/1575S/2015
A/duck/Egypt/1427SL/2014
A/duck/Egypt/BS-146RS-f6/2014
A/chicken/Egypt/14148CA/2014
A/chicken/Egypt/1510CA/2015
A/chicken/Egypt/AR233-S283NLQP/2014
A/Turkey/Egypt/14240FAOS/2014

(C)

Supplementary Fig. S1. Phylogenetic analysis of H5Nx HA sequences. Phylogenetic analysis of HA sequences of H5 viruses was carried out as described in the Supplementary Materials and Methods. Minor clades 2.3.3, 2.4, and 2.5 are present in the phylogeny, but are not indicated. (A) Overview of major H5 HA (sub)clades. Subclade 2.2.1.2 is shown in blue. (B) Enlarged view of subclade 2.2.1.2. The recently described novel cluster within subclade 2.2.1.2 is indicated by a bracket. (C) Enlarged view of the recently described novel cluster within subclade 2.2.1.2. The nine viruses characterized in this study are shown in red; those that transmitted among ferrets via respiratory droplets are indicated by arrows.

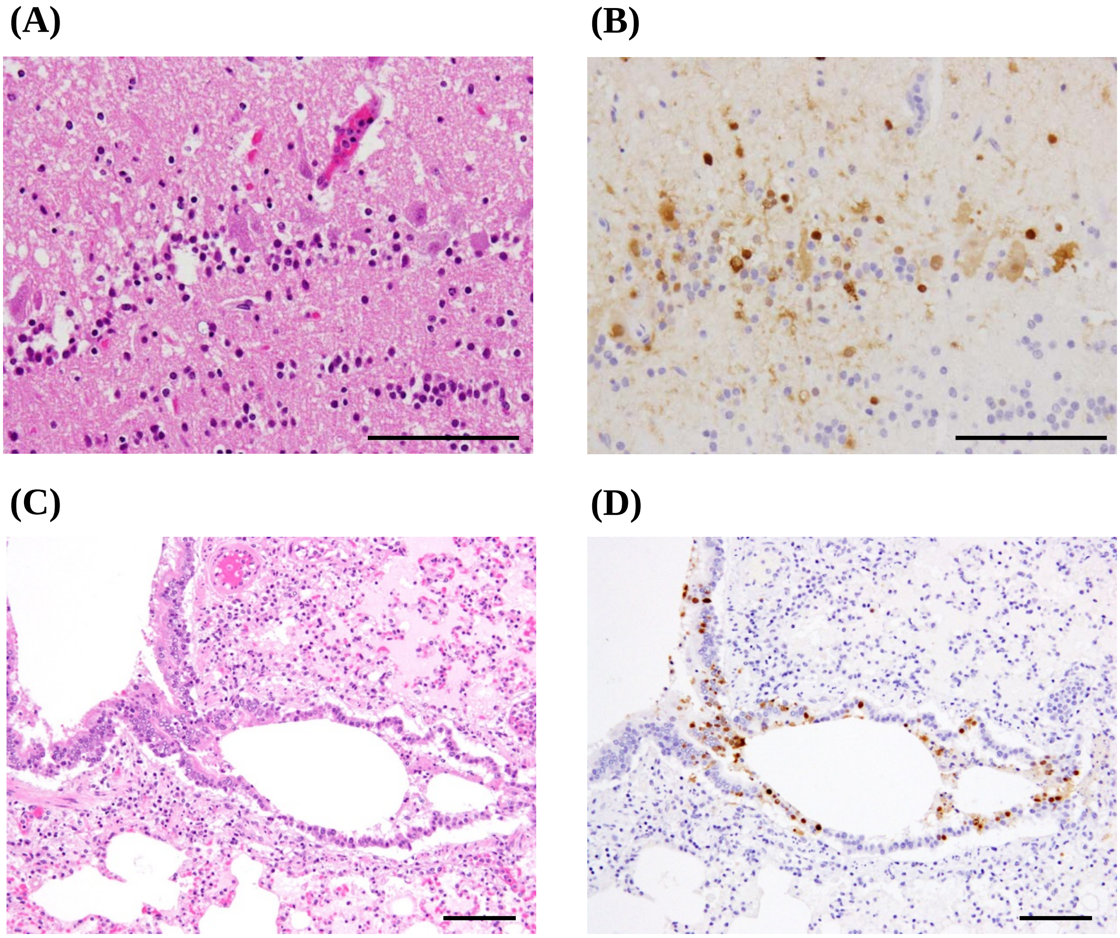

Supplementary Fig. S2. Histopathological findings in samples from the ferret that succumbed to inoculation with A/chicken/Sharkeya/14209SS/2014 on day 6 post-infection. Shown are hematoxylin-and-eosin (HE) stains (A, C) and immunohistochemistry (IHC) of anti-NP antigen (B, D). The cerebral tissue of rhinencephalon shows edematous change (A) and viral antigen was detected in neural cells (B). The lung shows bronchopneumonia with infiltration of inflammatory cells and edema (C) and viral antigen was detected mainly in the bronchial epithelial cells (D). Scale bars: 100 µm.

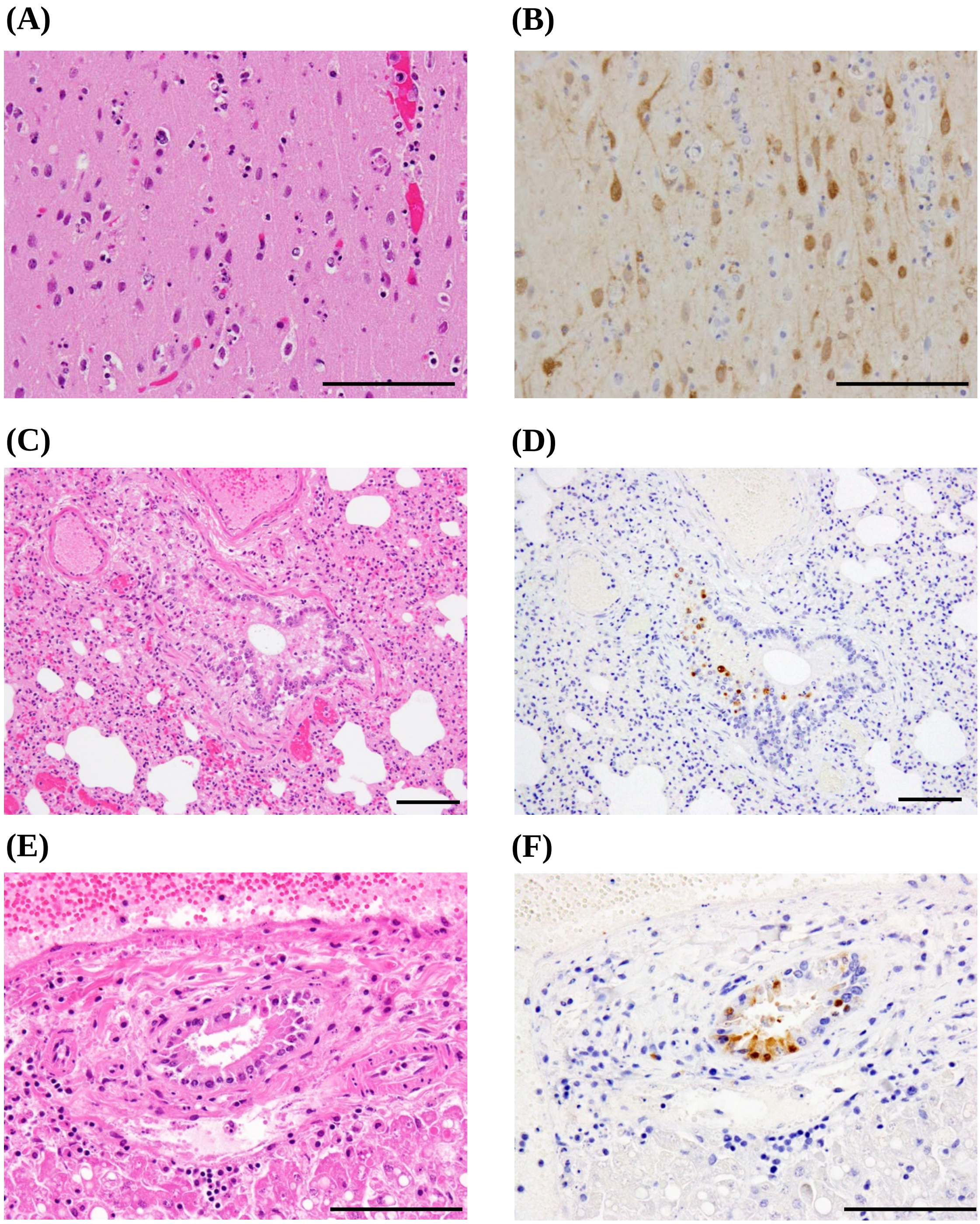

Supplementary Fig. S3. Histopathological findings in samples from the ferret that succumbed to virus infection after exposure to A/duck/Dakahlia/1536CAG/2015. Shown are HE stains (A, C, E) and IHC of anti-NP antigen (B, D, F). Encephalitis with neuronophagia was found in the cerebral cortex. Neural cells with viral antigen distributed multifocally in the whole brain (A). Multifocal regions of neural cells with viral antigen are distributed broadly in the whole brain (B). The lung shows severe bronchopneumonia with infiltration of inflammatory cells and edema, and hemorrhage (C). Viral antigen was detected in the bronchiolar epithelial cells (D). The liver shows infiltration of inflammatory cells in the portal region (E) and viral antigen was detected in biliary epithelial cells (F). Scale bars: 100 µm.

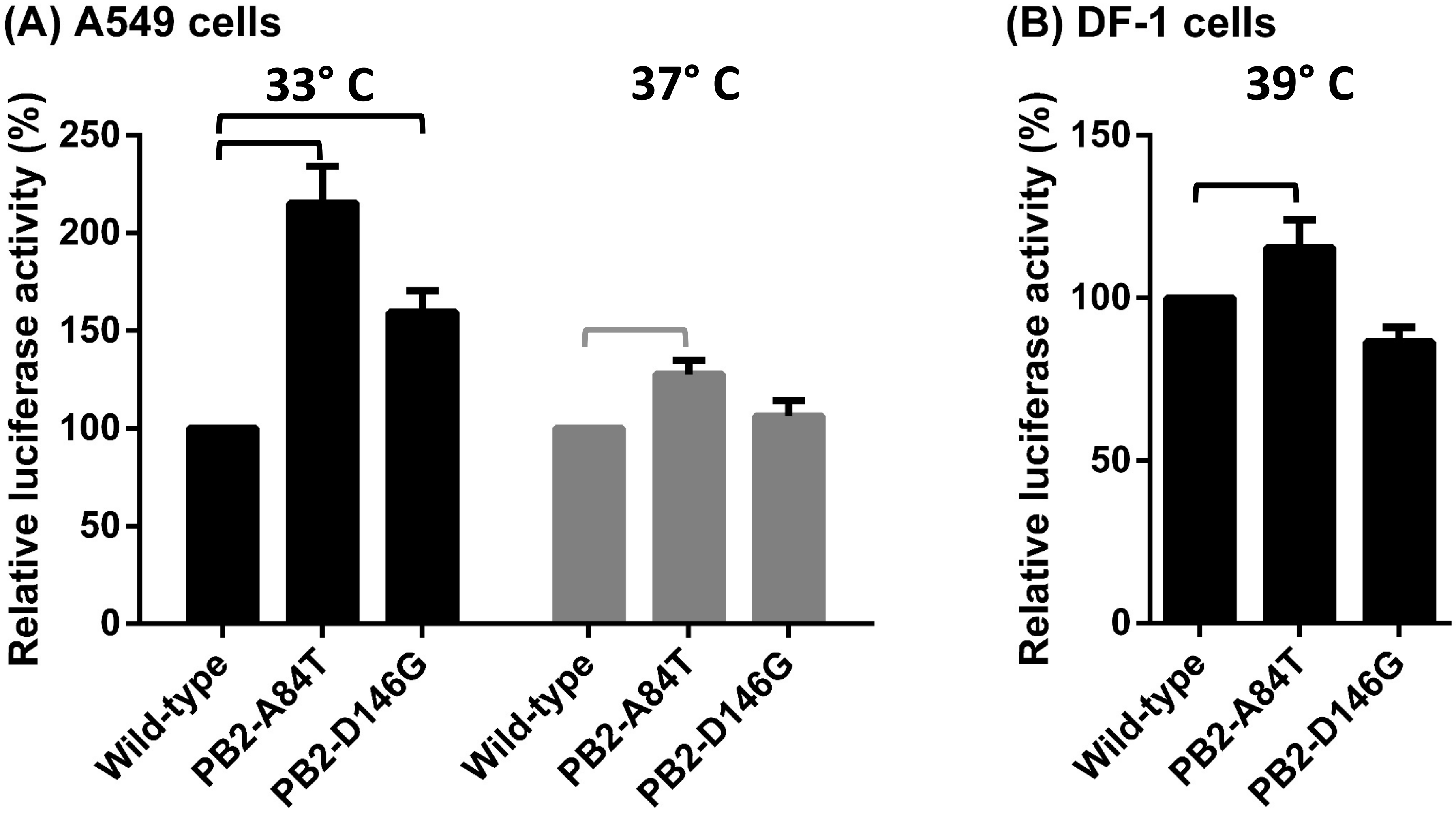

Supplementary Fig. S4. Mini-replicon assay to assess the effect of the PB2-A84T and PB2-D146G mutations on viral polymerase activity. Human A549 (A) or avian DF-1 (B) cells were transfected with plasmids for the expression of Giza PB2, PB1, PA, and NP proteins, with a plasmid for the expression of a virus-like RNA encoding firefly luciferase, and with a control plasmid expressing *Renilla* luciferase (used to normalize differences in transfection efficiencies). Alternatively, Dakahlia PB2-A84T or Giza-D146G protein was expressed instead of wild-type Giza PB2 (Giza and Dakahlia PB2, PB1, PA, and NP proteins are identical at the amino acid level). Transfected cells were incubated at the indicated temperatures. Firefly and *Renilla* luciferase activities were measured by means of a dual-luciferase assay at 24 h after transfection. Polymerase activity was calculated by normalization of the firefly luciferase activity to the *Renilla* luciferase activity. The data are shown as the relative polymerase activity ± standard deviation from two experiments (both performed in duplicate). The polymerase activity of the wild-type was set to 100%. Statistically significant differences (p <0.05) are shown by brackets.

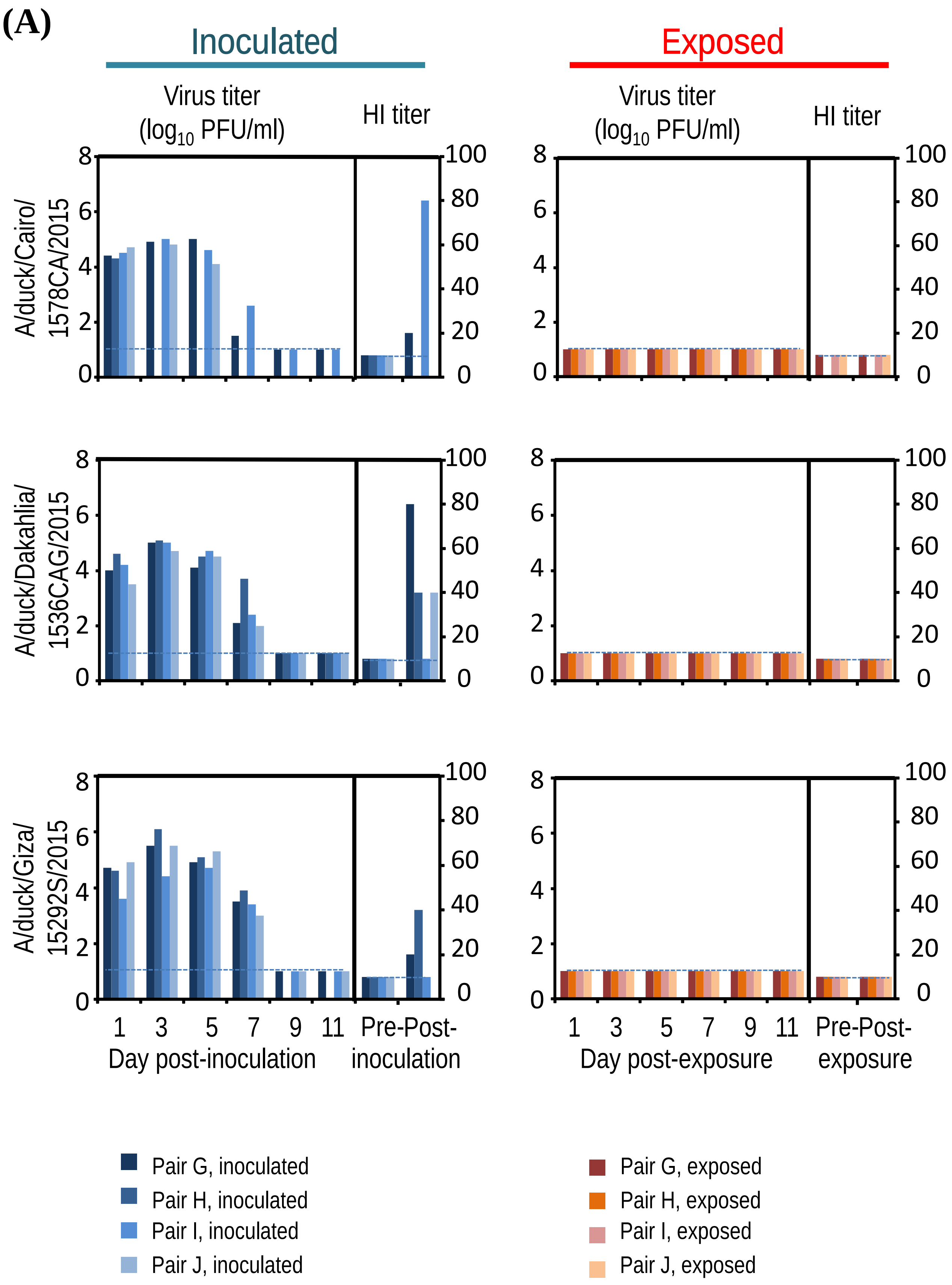
(A)
Inoculated
Exposed
Virus titer (log10 PFU/ml)
HI titer
A/duck/Cairo/ 1578CA/2015
A/duck/Dakahlia/ 1536CAG/2015
A/duck/Giza/ 15292S/2015
Day post-inoculation
Pre-Post-inoculation
Day post-exposure
Pre-Post-exposure
Pair G, inoculated
Pair H, inoculated
Pair I, inoculated
Pair J, inoculated
Pair G, exposed
Pair H, exposed
Pair I, exposed
Pair J, exposed

(B)

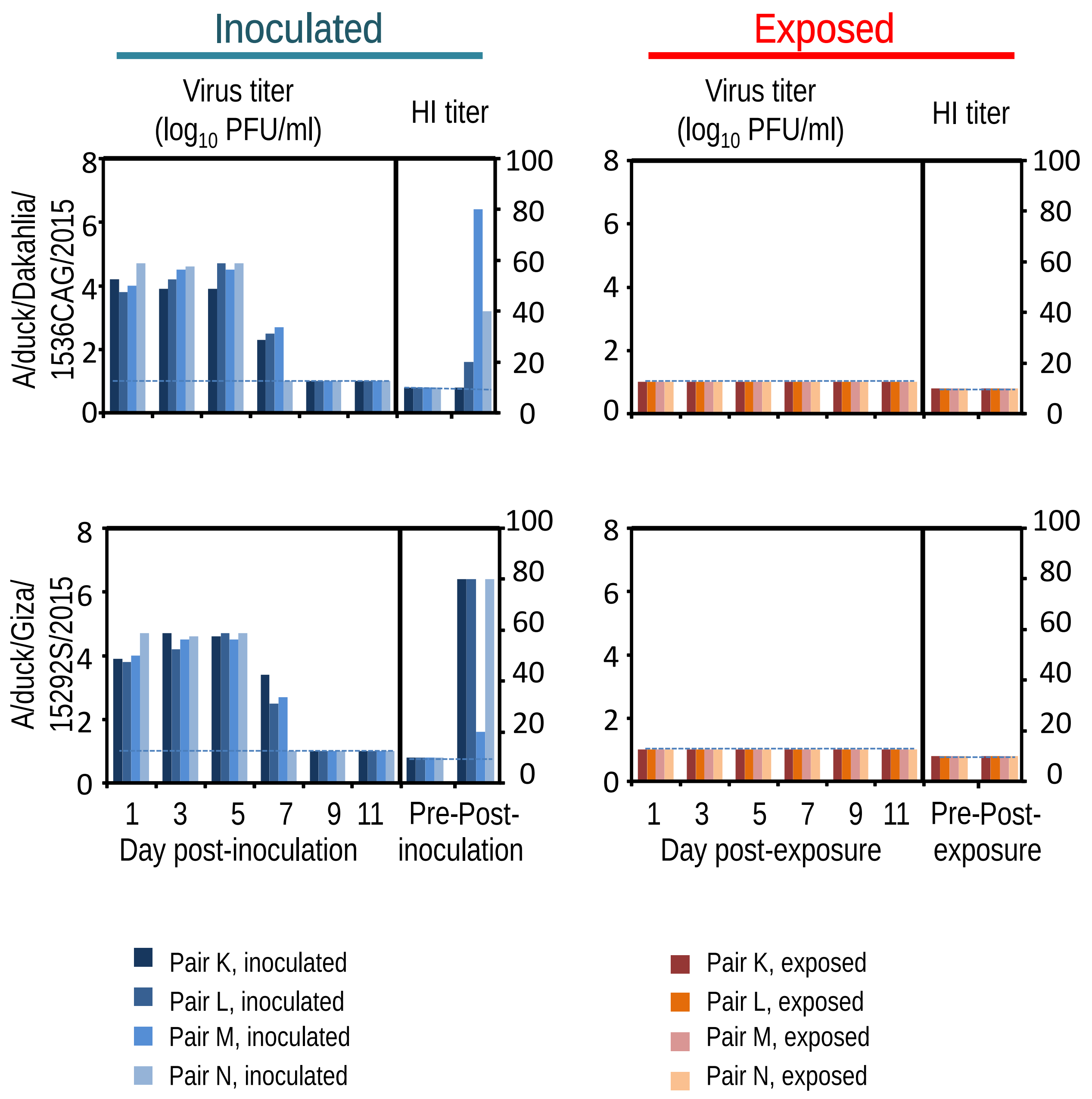
Inoculated
Exposed
Virus titer
(log10 PFU/ml)
HI titer
A/duck/Dakahlia/
1536CAG/2015
A/duck/Giza/
15292S/2015
0
2
4
6
8
0
20
40
60
80
100
1 3 5 7 9 11
Day post-inoculation
Pre-Post-
inoculation
Day post-exposure
Pre-Post-
exposure
Pair K, inoculated
Pair L, inoculated
Pair M, inoculated
Pair N, inoculated
Pair K, exposed
Pair L, exposed
Pair M, exposed
Pair N, exposed

(C)

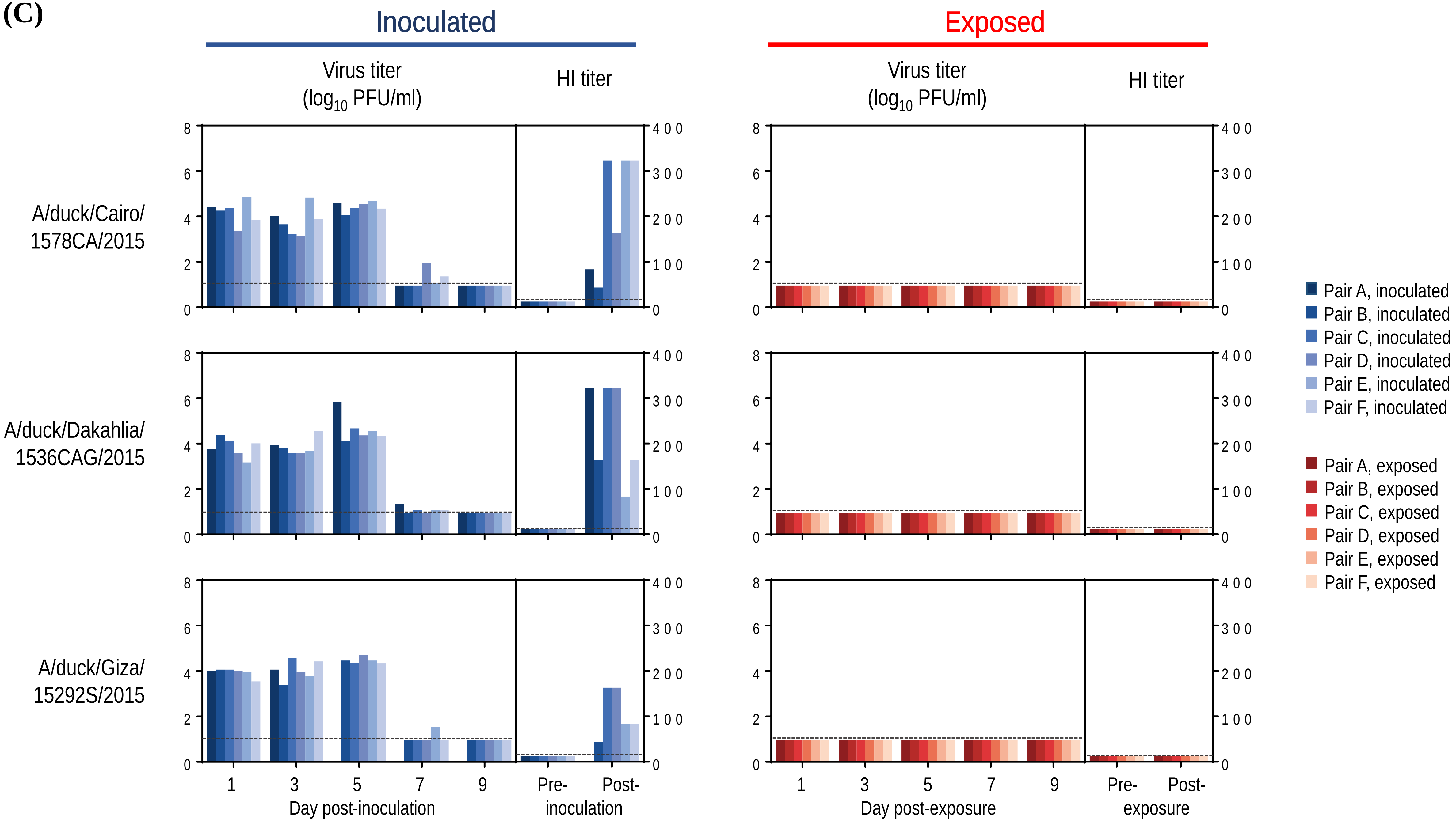
Inoculated
Exposed
Virus titer
(log10 PFU/ml)
HI titer
A/duck/Cairo/
1578CA/2015
A/duck/Dakahlia/
1536CAG/2015
A/duck/Giza/
15292S/2015
Day post-inoculation
Pre-
Post-
inoculation
Day post-exposure
exposure
Pair A, inoculated
Pair B, inoculated
Pair C, inoculated
Pair D, inoculated
Pair E, inoculated
Pair F, inoculated
Pair A, exposed
Pair B, exposed
Pair C, exposed
Pair D, exposed
Pair E, exposed
Pair F, exposed

Supplementary Figure S5. Additional respiratory droplet transmission studies in ferrets. Ferrets were inoculated with the indicated viruses ('Inoculated'). One day later, one naïve ferret was placed in a cage next to an inoculated ferret ('Exposed'). Virus titers were determined on the indicated days post-inoculation or -exposure, respectively. HI titers were measured pre-inoculation or -exposure, respectively, and on day 19, 22, or 26 post-inoculation or on day 19, 22, or 25 post-exposure. Horizontal lines indicate detection limits (i.e., a virus titer of 1.0 $\log_{10}$ PFU/ml or an HI titer of 10). (A) – (C) Shown are the results of three independent experiments.

Supplementary Table S1. Egyptian HPAI H5N1 viruses

Virus Isolate	Collection Date	Governorate	Source
A/chicken/Ismailia/144CAI/2014	09-Nov-2014	Ismailia	Household
A/chicken/Sharkeya/14209SS/2014	23-Dec-2014	Sharkeya	Household
A/duck/Cairo/157CA/2015	11-Jan-2015	Cairo	Household
A/duck/Cairo/1578CA/2015	02-Mar-2015	Cairo	Household
A/duck/Dakahlia/1536CAG/2015	10-Feb-2015	Dakahlia	Household
A/duck/Giza/15292S/2015	09-Mar-2015	Giza	Household
A/chicken/KafrElsheikh/UT-151CAD/2015	01-Jan-2015	KafrElsheikh	Household
A/duck/Menia/1543S/2015	12-Jan-2015	Menia	Household
A/turkey/Menia/15248S/2015	26-Feb-2015	Menia	Household

Supplementary Table S2. Virus titers in organs of inoculated ferrets[1]

Organ	Virus titers (mean log_{10} PFU/g ± SD/g) of animals infected with					
	A/duck/Cairo/1578CA/2015		A/duck/Dakahlia/1536CAG/2015		A/duck/Giza/15292S/2015	
	Day 3	Day 6	Day 3	Day 6	Day 3	Day 6
Nasal turbinate	4.8±1.5	5.4±0.4	6.3±1.2	4.7±0.3	7.4±0.4	4.7±1.1
Trachea	5.2±0.5	3.2±0.6	3.8	3.0, 3.1	2.4, 2.6	4.4±0.2
Lung	3.0±2.0	3.9	2.4	-	1.5	2.5
Liver	-[2]	-	-	-	-	-
Spleen	-	-	-	-	-	-
Kidney	-	-	-	-	-	-
Brain	2.2±0.3	2.4	4.0±0.4	2.3, 4.7[3]	2.6, 5.3	3.1±1.3
Rectum	-	-	3.3±1.4	-	1.8	-

[1]Ferrets were intranasally inoculated with 10^6 PFU (0.5 ml) of virus. Three ferrets from each group were euthanized at 3 and 6 days post-infection; [2]-; virus not detected (detection limit: 0.8 log_{10} PFU/g); [3]Individual titers are shown when virus was not recovered from all three ferrets.

Supplementary Table S3. Virus titers in the organs of a ferret that died after being inoculated with A/chicken/Sharkeya/14209SS/2014 virus

Organ[1]	Virus Titer (PFU/g)
Nasal turbinate	1.2×10^7
Trachea	2.5×10^2
Lung	6.0×10^6
Liver	n.d.[2]
Spleen	n.d.
Kidney	n.d.
Brain	1.0×10^4
Rectum	n.d.

[1]Organs were collected from the dead animal; virus titers were determined by means of plaque assays in MDCK cells; [2]n.d.: not detected (detection limit: 2.0 $\log_{10}$ PFU/g).

Supplementary Table S4. Virus titers in the organs of a ferret that died after exposure to A/duck/Dakahlia/ 1536CAG/2015 virus

Organ[1]	Virus Titer (PFU/g)
Nasal turbinate	2.0×10^6
Trachea	2.2×10^4
Lung	5.3×10^5
Liver	7.6×10^3
Spleen	6.0×10^5
Kidney	1.0×10^3
Brain	1.8×10^7
Rectum	4.5×10^3

[1]Organs were collected from the dead animal; virus titers were determined by means of plaque assays in MDCK cells.

Supplementary Table S5. Virus neutralization titers of sera obtained from ferrets inoculated with Egyptian H5N1 viruses

Virus	Pair	Viral neutralization titer[1]			
		Inoculated		Exposed	
		Pre[2]	Post[3]	Pre	Post
A/chicken/Ismailia/144CAI/2014	A	<4	256	<4	<4
	B	<4	256	<4	<4
A/chicken/Sharkeya/14209SS/2014	A	<4	n.d.[4]	<4	<4
	B	<4	256	<4	<4
A/duck/Cairo/157CA/2015	A	<4	256	<4	<4
	B	<4	128	<4	<4
A/duck/Cairo/1578CA/2015	A	<4	128	<4	128
	B	<4	128	<4	<4
A/duck/Dakahlia/1536CAG/2015	A	<4	128	<4	n.d.[4]
	B	<4	128	<4	<4
A/duck/Giza/15292S/2015	A	<4	64	<4	256
	B	<4	64	<4	64
A/chicken/KafrElsheikh/UT-151CAD/2015	A	<4	64	<4	<4
	B	<4	128	<4	n.d.
A/duck/Menia/1543S/2015	A	<4	128	<4	<4
	B	<4	128	<4	<4
A/turkey/Menia/15248S/2015	A	<4	>512	<4	<4
	B	<4	128	<4	<4

[1]Viral neutralization titers against homologous (inoculated) virus; [2]Sera were collected prior to virus inoculation; [3]Sera were collected on day 26 post-inoculation or on day 25 post-exposure; [4]Not done because the animal died before serum collection.

Supplementary Table S6. Virulence and replication properties of Egyptian H5N1 viruses in BALB/*c* mice

Virus	MLD_{50} (PFU)[1]	Virus titer (mean log_{10} PFU± SD/g)[2]					
		Days p.i.	Lung	Nasal turbinate	Spleen	Kidney	Brain
A/duck/Dakahlia/ 1536CAG/2015	100	3	6.6±0.3	4.4±0.9	4.1±0.3	n.d.[3]	1.5, 2.4, n.d.
		6	6.7±0.1	5.9±0.9	n.d., n.d., 2.4	4.4, 3.0, n.d.	3.6±1.5
A/duck/Giza/ 15292S/2015	5.6	3	7.1±0.1	5.1±0.1	5.2±0.5	2.2±0.2	n.d.
		6	6.9±0.6	6.8±0.8	2.0, n.d., n.d.	5.0±.08	5.3±0.5
A/duck/Cairo/ 1578CA/2015	17.8	3	6.9±0.1	5.0±0.4	n.d., 3.0, 3,7	n.d., n.d., 2.7	n.d.
		6	7.0±0.4	5.5±0.6	n.d.	3.8, 5.0, n.d.	4.1±0.5

[1]To determine the dose lethal to 50% of infected mice (MLD_{50}), three mice/group were intranasally inoculated with 10^1 to 10^5 PFU of virus in a 50-µl volume. The mice were then monitored for 14 days and examined daily for changes in body weight and mortality. The mice were euthanized if they lost more than 25% of their pre-infection body weight; [2]Six BALB/*c* mice per virus were inoculated intranasally with 10^3 PFU of virus in a 50-µl volume; three mice from each group were euthanized on days 3 and 6 post-infection (p.i.) for virus titration; [3] n.d.: not detected.

Supplementary Table S7. Amino acid changes detected in nasal wash samples from ferrets inoculated with Dakahlia or Giza virus[1]

Virus	Animal ID	Mutation		
		Gene	Nucleotide position	Amino acid change
Dakahlia	A	NS	A179A/C[2]	E60A (NS1)
	B	NS	A179A/C	E60A (NS1)
Giza	A	No amino acid changes		
	B	No amino acid changes		

[1]Nasal wash samples obtained on day 5 post-inoculation were analyzed by Sanger sequencing. Listed are non-synonymous amino acid changes compared with the virus stock used for infection; [2]Listed are the nucleotides changes: A179A/C indicates that the adenosine at nucleotide position 179 was replaced with a mixed population of adenosine (major sequencing signal) and cytosine (minor sequencing signal).

Supplementary Table S8. Amino acid changes detected in virus plaques obtained from nasal wash samples from ferrets inoculated with Dakahlia or Giza virus[1]

Virus	Animal ID	Plaque ID	Mutation		
			Gene	Nucleotide position	Amino acid change
Dakahlia	A	1	M	G919A	R77Q (M2)
			NS	A179C	E60A (NS1)
		2	M	G919A/G[2]	R77Q (M2)
		3	NA	G385A/G	V129I
			NS	A179C	E60A (NS1)
			NS	G284T	R95M (NS1)
		4	NS	A179C	E60A (NS1)
		5	No amino acid changes		
		6	NS	A179C	E60A (NS1)
		7	NP	G1295A/G	S432N
			NS	A179C	E60A (NS1)
		8	NS	T795C	I113T (NS2)
		9	No amino acid changes		
		10	PB2	G185A	R62K
			NS	A179C	E60A (NS1)
	B	1	PB2	A565G	K189E
			PB2	G2050A	A684T
			PB1	A1909G	I637V
			NS	A179C	E60A (NS1)
		2	PA	G1348A/G	V450I
		3	PB2	C904C/T	P302S
			PA	A424G/A	K142E
			NS	A179C	E60A (NS1)
		4	PB2	C904C/T	P302S
			NS	A179C/A	E60A (NS1)
		5	NA	C752G	A251G
			NS	A179C	E60A (NS1)
		6	PB2	C904C/T	P302S
		7	No amino acid changes		
		8	HA	A1109G/A	H354R
			NS	A179C	E60A (NS1)
		9	PB2	G748A	V250I
		10	NS	A179C	E60A (NS1)
Giza	A	1	PB2	G466A	A156T
		2	PB2	G466A	A156T
		3	PA	A1139G	D380G
		4	No amino acid changes		
		5	PB1	G377A	R126H
		6	NP	G778A	A260T
		7	PB1	A941G	N314S
		8	No amino acid changes		
		9	PB2	T554C/T	I185T
		10	No amino acid changes		

	B	1	PA<br>HA	A94G/A<br>G246A/G	T32A<br>M66I
		2	No amino acid changes		
		3	NA	C59T/C	A20V
		4	No amino acid changes		
		5	HA	G139A	D31N
		6	No amino acid changes		
		7	NP<br>NS	A1069C<br>C43A	Q357K<br>L15I (NS1)
		8	NP	A1069C	Q357K
		9	No amino acid changes		

[1]Nasal wash samples obtained on day 5 post-inoculation were subjected to plaque assays in MDCK cells. Individual viral plaques were analyzed by Sanger sequencing. Listed are non-synonymous amino acid changes compared with the virus stock used for infection; [2]Listed are the nucleotides changes: G919A/G indicates that the guanosine at nucleotide position 919 was replaced with a mixed population of adenosine (major sequencing signal) and cytosine (minor sequencing signal).

Supplementary Table S9. Amino acid changes detected in nasal wash samples from ferrets exposed to Dakahlia or Giza virus[1]

Virus	Animal ID	Day post-exposure	Mutation		
			Gene	Nucleotide position	Amino acid change
Dakahlia	A	7	PB2	G250G/A[2]	A84T
		9	PB2	G250G/A	A84T
			HA	G484G/A	V147I
Giza	A	3	PB2	A437G	D146G
		5	PB2	A437G	D146G
		7	PB2	A437G	D146G
		9	PB2	A437G	D146G
	B	7	PB2	A437G	D146G
		9	PB2	A437G	D146G

[1]Nasal wash samples obtained on the indicated day post-exposure were analyzed by Sanger sequencing. Listed are non-synonymous amino acid changes compared with the virus stock used for infection; [2]Listed are the nucleotides changes: G250G/A indicates that the guanosine at nucleotide position 250 was replaced with a mixed population of guanosine (major sequencing signal) and adenosine (minor sequencing signal).

Supplementary Table S10. Amino acid changes detected in virus plaques obtained from nasal wash samples from ferrets exposed to Dakahlia or Giza virus[1]

Virus	Animal ID	Plaque ID	Mutation		
			Gene	Nucleotide position	Amino acid change
Dakahlia	A	1	No mutations		
		2	HA<br>NP	G723A<br>A349G	M225I<br>R117G
		3	PB2	G250A	A84T
		4	No amino acid changes		
		5	PB2	G250A	A84T
Giza	A	1	PB2	A437G	D146G
		2	PB2	A437G	D146G
		3	PB2	A437G	D146G
		4	PB2	A437G	D146G
		5	PB2	A437G	D146G
		6	PB2	A437G	D146G
		7	PB2<br>HA	A437G<br>9 nucleotide insertion[2]	D146G<br>3 amino acid insertion[2]
		8	PB2	A437G	D146G
		9	PB2	A437G	D146G
		10	PB2	A437G	D146G
		11	PB2	A437G	D146G
	B	1	PB2	A437G	D146G
		2	PB2	A437G	D146G
		3	PB2<br>NS	A437G<br>T667C	D146G<br>S223P (NS1)
		4	PB2	A437G	D146G
		5	PB2	A437G	D146G
		6	PB2	A437G	D146G
		7	PB2	A437G	D146G
		8	PB2	A437G	D146G
		9	PB2	A437G	D146G
		10	PB2<br>PB2<br>HA	A437G<br>G1226A<br>C169A	D146G<br>C409Y<br>L41I

[1]Nasal wash samples obtained on day 9 post-exposure were subjected to plaque assays in MDCK cells. Individual viral plaques were analyzed by Sanger sequencing. Listed are non-synonymous amino acid changes compared with the virus stock used for infection; [2]Insertion of nine nucleotides (AGAAAAAAA) between HA nucleotides 1023 and 1024, resulting in the insertion of three amino acids (RKK) between amino acids 325 and 326, that is, at the HA cleavage site.

Supplementary Table S11. Viruses encoding PB2-84T or PB2-146G[1]

Virus	Subtype	Amino acid at PB2 position	
		84	146
A/equine/Guangxi/1/2008	H3N8	T	
A/Mexico_City/WRAIR3577T/2010	H3N2	T	
A/Queensland/33/2003	H3N2		G
A/chicken/British_Columbia/2004	H7N3		G
A/Wisconsin/629_S0410/2009	H1N1		G
A/swine/Thailand/CU_CT63/2011	H1N2		G
A/Nicaragua/5892_01_TR1/2013	H3N2		G

[1]We analyzed all publicly available influenza A virus PB2 sequences (>32,000).

Supplementary Table S12. Hemagglutination titers of Egyptian H5N1 viruses to chicken red blood cells[1]

Virus	Untreated CRBCs	CRBC treated with a Siaα2,3-linkage-specific sialidase
rgA/Kawasaki/173/2001 (H1N1)	16	32
rgA/Vietnam/1203/2004 (H5N1)	16	<2
A/chicken/Iswailia/144CAI/2014 (H5N1)	16	8
A/chicken/Sharkeya/14209SS/2014 (H5N1)	16	16
A/duck/Cairo/157CA/2015 (H5N1)	16	16
A/duck/Cairo/1578CA/2015 (H5N1)	16	16
A/duck/Dakahlia/1536CAG/2015 (H5N1)	16	16
A/duck/Giza/15292S/2015 (H5N1)	16	16
A/chicken/KafrElsheikh/UT-151CAD/2015 (H5N1)	16	<2
A/duck/Menia/1543S/2015 (H5N1)	16	32
A/turkey/Menia/15248S/2015 (H5N1)	16	8

[1]Viruses were tested for agglutination of untreated chicken red blood cells (CRBCs) possessing both Siaα2,3Gal and Siaα2,6Gal, or CRBC treated with a Siaα2,3-linkage-specific sialidase, leaving predominantly Siaα2,6Gal (i.e., human-type receptors).

Supplementary Table S13. Virus neutralization and hemagglutination inhibition antibody titers to Egyptian H5N1 viruses of sera obtained from individuals vaccinated with H5N1 candidate vaccine viruses

Virus	Human serum[1]									
	rgA/Vietnam/1203/2004 (H5N1) (clade 1)				A/Indonesia/05/2005 PR8-IBCDC-RG2 (H5N1) (clade 2.1.3.2)					
	Low-titer pool (NR-4110)		High-titer pool (NR-4109)		Low-titer pool (NR-33667)		Medium-titer pool (NR-33668)		High-titer pool (NR-33669)	
	Neutral.	HI	Neutral.	HI	Neutral.	HI	Neutral.	HI	Neutral.	HI
rgA/Vietnam/1203/2004 (H5N1)[2]	8	<10	16	10	ND					
A/Indonesia/UT3006/2005 (H5N1)	ND				16	<10	64	20	256	80
Dakahlia	<4	<10	4	<10	<4	<10	4	<10	16	10
Giza	<4	<10	4	<10	4	<10	8	<10	32	10
Cairo	<4	<10	4	<10	<4	<10	8	<10	16	<10

[1]NR-4110 and NR-4109 are low- and high-titer pools of polyclonal antisera, respectively, from individuals vaccinated with a monovalent influenza subvirion vaccine, rgA/Vietnam/1203/2004×A/PR/8/34 (H5N1). NR-33667, NR-33668, and NR-33669 are low-, medium-, and high-titer pools of polyclonal antisera, respectively, from individuals vaccinated with a monovalent influenza subvirion vaccine, A/Indonesia/05/2005 PR8-IBCDC-RG2 (H5N1). These reagents were obtained from BEI Resources. Virus neutralization and HI assays were carried out as described in the Materials and Methods section. ND, not determined. [2]This virus was generated by using reverse genetics.

Supplementary Table S14. Virus neutralization and hemagglutination inhibition antibody titers to Egyptian H5N1 viruses of sera obtained from individuals vaccinated with an H5N1 candidate vaccine virus

Virus	H5V-1[1]				H5V-2				H5V-3				H5V-4				H5V-9				H5V-13				H5V-16				H5V-19			
	Neutral.[2]		HI[3]		Neutral.		HI		Neutral.		HI		Neutral.		HI		Neutral.		HI		Neutral.		HI		Neutral.		HI		Neutral.		HI	
	Pre-vacc.	Post-vacc.	Pre-vacc.	Post-vacc.	Pre-vacc.	Post-vacc.	Pre-vacc.	Post-vacc.	Pre-vacc.	Post-vacc.	Pre-vacc.	Post-vacc.	Pre-vacc.	Post-vacc.	Pre-vacc.	Post-vacc.	Pre-vacc.	Post-vacc.	Pre-vacc.	Post-vacc.	Pre-vacc.	Post-vacc.	Pre-vacc.	Post-vacc.	Pre-vacc.	Post-vacc.	Pre-vacc.	Post-vacc.	Pre-vacc.	Post-vacc.	Pre-vacc.	Post-vacc.
rgEgypt-N03072 HA/PR8[4]	<4	8	<10	<10	<4	32	<10	40	<4	16	<10	80	<4	32	<10	80	<4	4	<10	10	<4	4	<10	<10	<4	16	<10	20	<4	4	<10	10
Dakahlia	<4	<4	<10	<10	<4	8	<10	10	<4	16	<10	40	<4	4	<10	40	<4	<4	<10	<10	<4	<4	<10	<10	<4	<4	<10	10	<4	<4	<10	<10
Giza	<4	4	<10	<10	<4	32	<10	10	<4	16	<10	40	<4	16	<10	40	<4	4	<10	<10	<4	4	<10	<10	<4	32	<10	10	<4	<4	<10	<10
Cairo	<4	<4	<10	<10	<4	32	<10	<10	<4	16	<10	20	<4	32	<10	20	<4	<4	<10	<10	<4	<4	<10	<10	<4	16	<10	<10	<4	<4	<10	<10

[1]Sera were obtained from individuals vaccinated twice with the clade 2.2.1 alum-adjuvanted, inactivated whole candidate vaccine virus A/Egypt/N03072/2010 (H5N1; IDCDC-RG 29). Blood samples were collected prior to vaccination and 31-38 days after the second vaccination (see Table S15). [2]Neutralization titer pre-vaccination (pre-vacc.) and post-vaccination (post-vacc.), respectively. [3]HI titer pre-vaccination (pre-vacc.) and post-vaccination (post-vacc.), respectively. [4]Reverse genetics virus possessing the A/Egypt/N03072/2010 (H5N1) HA gene in the genetic background of A/Puerto Rico/8/34 (H1N1) virus, which is commonly used as a genetic backbone for human influenza viruses.

Supplementary Table S15. Dates of vaccination with A/Egypt/N03072/2010 (H5N1; IDCDC-RG 29) and serum collection

Human serum ID	Date			
	Serum collection pre-vaccination	First vaccination	Second vaccination	Serum collection post-vaccination
H5V-1	Nov 06, 2013	Nov 13, 2013	Dec 04, 2013	Jan 06, 2014
H5V-2	Nov 06, 2013	Nov 13, 2013	Dec 03, 2013	Jan 06, 2014
H5V-3	Nov 06, 2013	Nov 13, 2013	Dec 03, 2013	Jan 06, 2014
H5V-4	Nov 06, 2013	Dec 04, 2013	Dec 24, 2013	Jan 24, 2014
H5V-9	Nov 07, 2013	Nov 13, 2013	Dec 04, 2013	Jan 06, 2014
H5V-13	Nov 08, 2013	Nov 13, 2013	Dec 04, 2013	Jan 07, 2014
H5V-16	Nov 08, 2013	Nov 13, 2013	Dec 03, 2013	Jan 10, 2014
H5V-19	Nov 11, 2013	Nov 12, 2013	Dec 04, 2013	Jan 07, 2014
A638[1]	Apr 24, 2015	-	-	

[1]Serum from unvaccinated individual (control).

Supplementary Table S16. Inhibition of the neuraminidase activity of Egyptian H5N1 viruses and control H1N1 viruses by neuraminidase inhibitors

	IC_{50} value (nM)[1]			
	Oseltamivir carboxylate	Zanamivir	Peramivir	Laninamivir
A/chicken/Ismailia/144CAI/2014	2.7	1.3	0.15	0.07
A/chicken/Sharkeya/14209SS/2014	5.2	1.0	0.16	0.14
A/duck/Cairo/157CA/2015	2.0	1.1	0.08	0.08
A/duck/Cairo/1578CA/2015	1.9	1.3	0.12	0.15
A/duck/Dakahlia/1536CAG/2015	2.2	1.0	0.13	0.07
A/duck/Giza/15292S/2015	5.3	1.2	0.2	0.07
A/chicken/KafrElsheikh/UT-151CAD/2015	4.7	1.6	0.22	0.12
A/duck/Menia/1543S/2015	1.8	1.2	0.25	0.07
A/turkey/Menia/15248S/2015	3.3	1.4	0.14	0.06
A/California/04/2009 (H1N1)	1.0	0.3	0.17	0.4
A/Kawasaki/UTK-4/2009 (H1N1)[2]	1313.0	0.8	74	0.3
A/Kawasaki/UTK-23/2008 (H1N1)	1.9	0.4	0.68	0.2

[1]Viruses were tested for their sensitivities to the indicated neuraminidase inhibitors. IC_{50} values represent the means of duplicate reactions.

[2]This virus encodes a mutation at NA position 275 (N1 numbering) and is known to be resistant to oseltamivir.

Supplementary Table S17. HI and neutralization titers of ferret sera derived from inoculated or exposed animals
(Transmission study 2)

Virus	Pair	HI titer[1]				Neutralization titer[1]			
		Inoculated		Exposed		Inoculated		Exposed	
		Pre[2]	Post[3]	Pre	Post	Pre	Post	Pre	Post
Cairo	C	<10	20	<10	<10	<4	64	<4	<4
	D	ND[4]	ND	ND	ND	ND	ND	ND	ND
	E	<10	80	<10	<10	<4	128	<4	<4
	F	ND	ND	<10	<10	ND	ND	<4	<4
Dakahlia	C	<10	80	<10	<10	<4	128	<4	<4
	D	<10	40	<10	<10	<4	64	<4	<4
	E	<10	<10	<10	<10	<4	32	<4	<4
	F	<10	40	<10	<10	<4	32	<4	<4
Giza	C	<10	20	<10	<10	<4	64	<4	<4
	D	<10	40	<10	<10	<4	64	<4	<4
	E	<10	10	<10	<10	<4	32	<4	<4
	F	<10	ND	<10	<10	<4	ND	<4	<4

[1]Viral HI and neutralization titers against homologous (inoculated) virus; [2]Sera were collected prior to virus inoculation; [3]Sera were collected on day 19 post-inoculation or on day 19 post-exposure; [4]Not done because the animal died before serum collection.

Supplementary Table S18. HI and neutralization titers of ferret sera derived from inoculated or exposed animals (Transmission study 3)

Virus	Pair	HI titer[1]				Neutralization titer[1]			
		Inoculated		Exposed		Inoculated		Exposed	
		Pre[2]	Post[3]	Pre	Post	Pre	Post	Pre	Post
Dakahlia	G	<10	10	<10	<10	<4	64	<4	<4
	H	<10	20	<10	<10	<4	64	<4	<4
	I	<10	80	<10	<10	<4	128	<4	<4
	J	<10	40	<10	<10	<4	64	<4	<4
Giza	G	<10	80	<10	<10	<4	256	<4	<4
	H	<10	80	<10	<10	<4	128	<4	<4
	I	<10	20	<10	<10	<4	128	<4	<4
	J	<10	80	<10	<10	<4	128	<4	<4

[1]Viral HI and neutralization titers against homologous (inoculated) virus; [2]Sera were collected prior to virus inoculation; [3]Sera were collected on day 22 post-inoculation or on day 22 post-exposure.

Supplementary Table S19. GISAID acknowledgement

***We acknowledge the authors, and the originating and submitting laboratories of the sequences from the GISAID's EpiFlu*™ Database on which this research is based. The list is detailed below.**

All submitters of data may be contacted directly via the GISAID website www.gisaid.org

Segment ID	Segment	Country	Collection date	Isolate name	Originating Lab	Submitting Lab	Authors
EPI623571	HA	United Arab Emirates	2014-Dec-07	A/Falcon/Dubai/2506/2014		Friedrich-Loeffler-Institut	Chen, H.; Chan, KH. ; Wong, PC. ; Woo, C.Y.P.
EPI603553	HA	United Arab Emirates	2014-Nov-17	A/Falcon/Dubai/AR3430-2293/2014		Friedrich-Loeffler-Institut	Naguib, M.M.; Wernery, U.; Harder, T.
EPI241681	HA	Denmark	2006-Mar-03	A/peregrine/Denmark/6632/2006	National Veterinary Institute	Statens Serum Institute	Bragstad,K.; Fomsgaard,A.; Jorgensen,P.H.; Hammer,A.S.; Kabell,S.; Handberg,K.
EPI588952	HA	Taiwan	2015-Jan-11	A/goose/Taiwan/a015/2015	ANIMAL HEALTH RESEARCH INSTITUTE	Animal Health Research Institute	Yu-Ju,Lin; Li-Hsuan,Chen; Wan-Chen,Li; Yu-Pin,Liu; Ming-Shiuh,Lee; Ming-Chu,Cheng; Hsiang-Jung,Tsai
EPI573220	HA	Korea, Republic of	2014-Jul-31	A/goose/Korea/H1698/2014		Animal and Plant Quarantine Agency	
EPI573219	HA	Korea, Republic of	2014-Jul-29	A/goose/Korea/H1689/2014		Animal and Plant Quarantine Agency	
EPI573211	HA	Korea, Republic of	2014-Jun-13	A/goose/Korea/H1545/2014		Animal and Plant Quarantine Agency	
EPI573204	HA	Korea, Republic of	2014-Apr-21	A/goose/Korea/H1296/2014		Animal and Plant Quarantine Agency	
EPI305543	HA	Turkey	2006-Jan-06	A/Goose/Turkey-Agri/09rs2841-17/2006	Istituto Zooprofilattico Sperimentale Delle Venezie	Istituto Zooprofilattico Sperimentale Delle Venezie	Schivo,A.; Valastro,V.; Monne,I.; Coven,F.; Fusaro,A.; Dakman,A.; Akcadag,B.; Salviato,A.; DeBattisti,C.; Capua,I.; Cattoli,G.
EPI244110	HA	Germany	2009-Dec-01	A/goose/Germany/R3160/09		Friedrich-Loeffler-Institut	
EPI595116	HA	Korea, Republic of	2014-Dec-24	A/greater white-fronted goose/Korea/K14-367-4/2014	Avian diaseases laboratory, College of Veterinary Medicine, Konkuk University	Konkuk University	Kwon,Jung-Hoon; Yuk,Seong-Su; Erdene-Ochir,TO; Noh,Jin-Yong; Hong,Woo-Tack; Jeong,Jei-Hyun; Jeong,Sol; Song,Chang-Seon
EPI241729	HA	Denmark	2006-Mar-21	A/grey lag goose/Denmark/6692/2006	National Veterinary Institute	Statens Serum Institute	Bragstad,K.; Fomsgaard,A.; Jorgensen,P.H.; Hammer,A.S.; Kabell,S.; Handberg,K.
EPI603561	HA	United Arab Emirates	2014-Dec-07	A/Sea Gull/Dubai/AR3443-25041/2014		Friedrich-Loeffler-Institut	Naguib, M.M.; Wernery, U.; Harder, T.
EPI345436	HA	Belgium	2010-Jan-27	A/Larus argentatus/Belgium/02936cls9/2010	Veterinary and Agrochemical Research Institute	Veterinary and Agrochemical Research Institute	Van Borm,S.; Rosseel,T.; Lambrecht,B.; Vangeluwe,D.; Vandenbussche,F.; van den Berg,T.
EPI371563	HA	South Africa	2011-Apr-19	A/ostrich/South Africa/1104333C/2011	Western Cape Provincial Veterinary Laboratory	Animal and Plant Health Agency (APHA)	Russell, C; Hanna, A; Shell, W; Focosi-Snyman, R; Manvell, R; Gers, S; Abolnik, C; Reid, S
EPI371562	HA	South Africa	2011-Apr-19	A/ostrich/South Africa/1104333A/2011	Western Cape Provincial Veterinary Laboratory	Animal and Plant Health Agency (APHA)	Russell, C; Hanna, A; Shell, W; Focosi-Snyman, R; Manvell, R; Gers, S; Abolnik, C; Reid, S
EPI174880	HA	Denmark	1996-Apr-09	A/ostrich/Denmark/96-72420/1996	Technical University of Denmark	National Veterinary Institute	
EPI169515	HA	Saudi Arabia	2007-Jan-01	A/ostrich/Saudi Arabia/3489-73VIR08/2007		Istituto Zooprofilattico Sperimentale Delle Venezie	
EPI557138	HA	Egypt	2014-Jun-15	A/Quail/Egypt/BSU5514-AR2219/2014		Friedrich-Loeffler-Institut	Naguib,M.M; El-Kady,M.F; Hassan, K.E; Abo-Zeid,H.H; Beer,M; Harder,TC
EPI375595	HA	Ireland	2012-Mar-24	A/pheasant/Ireland/PV12-010728/12	Central Veterinary Research Laboratory	Animal and Plant Health Agency (APHA)	Puranik, A; Thomas, S; Hanna, A; Essen, S; Focosi-Snyman, R; Manvell, R.J; Raleigh, P; Flynn, O; Reid, S
EPI559789	HA	China	2015-Jan-05	A/whooper swan/Henan/SMX4/2015(H5N1)	CAS Key Laboratory of Pathogenic Microbiology and Immunology, Institute of Microbiology, Chinese Academy of Sciences	Institute of Microbiology, Chinese Academy of Sciences	
EPI559770	HA	China	2015-Jan-04	A/whooper swan/Henan/SMX3/2015(H5N1)	CAS Key Laboratory of Pathogenic Microbiology and Immunology, Institute of Microbiology, Chinese Academy of Sciences	Institute of Microbiology, Chinese Academy of Sciences	
EPI559727	HA	China	2015-Jan-04	A/whooper swan/Henan/SMX1/2015(H5N1)	CAS Key Laboratory of Pathogenic Microbiology and Immunology, Institute of Microbiology, Chinese Academy of Sciences	Institute of Microbiology, Chinese Academy of Sciences	
EPI346524	HA	Germany	2009-Jan-01	A/whooper swan/Germany-HE/R535/2009		Friedrich-Loeffler-Institut	
EPI241751	HA	Denmark	2006-Apr-03	A/whooper swan/Denmark/7224/2006	National Veterinary Institute	Statens Serum Institute	Bragstad,K.; Fomsgaard,A.; Jorgensen,P.H.; Hammer,A.S.; Kabell,S.; Handberg,K.
EPI241656	HA	Denmark	2006-Apr-05	A/whooper swan/Denmark/7275/2006	National Veterinary Institute	Statens Serum Institute	Bragstad,K.; Fomsgaard,A.; Jorgensen,P.H.; Hammer,A.S.; Kabell,S.; Handberg,K.
EPI156837	HA	Poland	2006-Apr-03	A/swan/Poland/1242-139V08/2006		National Veterinary Research Institute	
EPI156789	HA	Poland	2006-Mar-04	A/swan/Poland/305-135V08/2006		National Veterinary Research Institute	
EPI584079	HA	Canada	2014-Dec-06	A/turkey/BC/FAV14/2014	Animal Health Centre, Ministry of Agriculture	Canadian Food Inspection Agency	
EPI574389	HA	Egypt	2014-Oct-31	A/Turkey/Egypt/14240FAOS/2014		Friedrich-Loeffler-Institut	Arafa,A.M; Hagag, N; Elhusseiny, M.H; Yehia, N; Selim, A.A; Abdelhalim, A; Kilany, W.H; Samy, A; Hassan, M.K; Abdelwhab,E.M; Beer,M; Naguib, M.M; Harder,TC
EPI574381	HA	Egypt	2014-Jun-18	A/Turkey/Egypt/14139FAOS/2014		Friedrich-Loeffler-Institut	Arafa,A.M; Hagag, N; Elhusseiny, M.H; Yehia, N; Selim, A.A; Abdelhalim, A; Kilany, W.H; Samy, A; Hassan, M.K; Abdelwhab,E.M; Beer,M; Naguib, M.M; Harder,TC
EPI573268	HA	Egypt	2014-Dec-06	A/Turkey/Egypt/AR238-SD177NLQP/2014		Friedrich-Loeffler-Institut	Naguib, M.M; Arafa,A.M; Luttermann,C; Selim,A.A; Hassan,M.K; Beer,M;Harder,TC
EPI573252	HA	Egypt	2014-Oct-31	A/Turkey/Egypt/AR235-S240NLQP/2014		Friedrich-Loeffler-Institut	Naguib, M.M; Arafa,A.M; Luttermann,C; Selim,A.A; Hassan,M.K; Beer,M;Harder,TC
EPI552746	HA	Germany	2014-Nov-04	A/turkey/Germany/AR2485-86-L00899/2014		Friedrich-Loeffler-Institut	
EPI544756	HA	Germany	2014-Nov-04	A/turkey/Germany-MV/R2472/2014		Friedrich-Loeffler-Institut	
EPI464956	HA	Italy	2012-Sep-03	A/turkey/Italy/12VIR-6651/2012	Istituto Zooprofilattico Sperimentale Delle Venezie	Istituto Zooprofilattico Sperimentale Delle Venezie	Monne, I.; Salviato, A.; Tassoni, L.; Cattoli, G.
EPI464954	HA	Italy	2012-Jun-28	A/turkey/Italy/12VIR-8036-2/2012	Istituto Zooprofilattico Sperimentale Delle Venezie	Istituto Zooprofilattico Sperimentale Delle Venezie	Monne, I.; Salviato, A.; Tassoni, L.; Cattoli, G.
EPI305602	HA	Turkey	2007-Feb-15	A/Turkey/Turkey/Batman/09rs2842-110/2007	Istituto Zooprofilattico Sperimentale Delle Venezie	Istituto Zooprofilattico Sperimentale Delle Venezie	Schivo,A.; Valastro,V.; Monne,I.; Coven,F.; Fusaro,A.; Dakman,A.; Akcadag,B.; Salviato,A.; DeBattisti,C.; Capua,I.; Cattoli,G.
EPI305596	HA	Turkey	2007-Feb-15	A/Turkey/Turkey/Diyarbakir/09rs2842-93/2007	Istituto Zooprofilattico Sperimentale Delle Venezie	Istituto Zooprofilattico Sperimentale Delle Venezie	Schivo,A.; Valastro,V.; Monne,I.; Coven,F.; Fusaro,A.; Dakman,A.; Akcadag,B.; Salviato,A.; DeBattisti,C.; Capua,I.; Cattoli,G.
EPI305554	HA	Turkey	2006-Jan-23	A/Turkey/Turkey/Kars/09rs2841-42/2006	Istituto Zooprofilattico Sperimentale Delle Venezie	Istituto Zooprofilattico Sperimentale Delle Venezie	Schivo,A.; Valastro,V.; Monne,I.; Coven,F.; Fusaro,A.; Dakman,A.; Akcadag,B.; Salviato,A.; DeBattisti,C.; Capua,I.; Cattoli,G.
EPI631928	HA	Russian Federation	2015-May-07	A/rook/Sartlan/42/2015	State Research Center of Virology and Biotechnology Vector	State Research Center of Virology and Biotechnology Vector	Ivan,Susloparov; Vasily,Marchenko; Natalya,Goncharova; Andrey,Shipovalov; Alexander,Durymanov; Tatyana,Ilyicheva; Alexander,Ryzhikov
EPI631912	HA	Russian Federation	2015-May-07	A/rook/Chany/32/2015	State Research Center of Virology and Biotechnology Vector	State Research Center of Virology and Biotechnology Vector	Ivan,Susloparov; Vasily,Marchenko; Natalya,Goncharova; Andrey,Shipovalov; Alexander,Durymanov; Tatyana,Ilyicheva; Alexander,Ryzhikov
EPI603569	HA	United Arab Emirates	2014-Dec-07	A/Stone curlew/Dubai/AR3444-25042/2014		Friedrich-Loeffler-Institut	Naguib, M.M.; Wernery, U.; Harder, T.
EPI592605	HA	Bulgaria	2015-Jan-23	A/dalmatian pelican/Bulgaria/4/2015	NDRVMI (National Diagnostic and Research Veterinary Medical Institute)	Animal and Plant Health Agency (APHA)	Thomas, S; Seekings, A; Essen, S; Manvell, R; Goujgoulova, G; Oreshkova, L; Banks, J; Brown, I
EPI592416	HA	Bulgaria	2015-Jan-23	A/dalmatian pelican/Bulgaria/3/2015	NDRVMI (National Diagnostic and Research Veterinary Medical Institute)	Animal and Plant Health Agency (APHA)	Thomas, S; Seekings, A; Essen, S; Manvell, R; Goujgoulova, G; Oreshkova, L; Banks, J; Brown, I
EPI573664	HA	Japan	2015-Jan-03	A/crane/Kagoshima/KU53/2015(H5N8)	Kagoshima University	Kagoshima University	
EPI573654	HA	Japan	2014-Dec-24	A/crane/Kagoshima/KU41/2014(H5N8)	Kagoshima University	Kagoshima University	
EPI573646	HA	Japan	2014-Dec-17	A/crane/Kagoshima/KU21/2014(H5N8)	Kagoshima University	Kagoshima University	
EPI553208	HA	Japan	2014-Nov-23	A/crane/Kagoshima/KU1/2014	Kagoshima University	Kagoshima University	
EPI356865	HA	Nepal	2012-Jan-18	A/crow/Nepal/CT1/12	Central Veterinary Laboratory	Animal and Plant Health Agency (APHA)	Puranik, A; Hanna, A; Essen, S; Focosi-Snyman, R; Manvell, R.J; Sedai, D; Chapagain, S; Manandhar, S; Koirala, P; Karki, K.B; Pandey, K.R; Air, T.B; Reid, S
EPI346566	HA	Germany	2006-Jan-01	A/Common Buzzard/Berlin/1/2006	Robert-Koch-Institute	Robert-Koch-Institute	Schweiger, Brunhilde; Zöhner, Andrea; Wolff, Thorsten
EPI293904	HA	Turkey	2005-Dec-10	A/Pigeon/Turkey-Agri/09rs2841-8/2005	Istituto Zooprofilattico Sperimentale Delle Venezie	Istituto Zooprofilattico Sperimentale Delle Venezie	Schivo,A.; Valastro,V.; Monne,I.; Coven,F.; Fusaro,A.; Dakman,A.; Akcadag,B.; Salviato,A.; DeBattisti,C.; Capua,I.; Cattoli,G.
EPI293896	HA	Turkey	2005-Dec-09	A/Pigeon/Turkey-Kars/09rs2841-7/2005	Istituto Zooprofilattico Sperimentale Delle Venezie	Istituto Zooprofilattico Sperimentale Delle Venezie	Schivo,A.; Valastro,V.; Monne,I.; Coven,F.; Fusaro,A.; Dakman,A.; Akcadag,B.; Salviato,A.; DeBattisti,C.; Capua,I.; Cattoli,G.

EPI241665	HA	Denmark	2006-May-16	A/peacock/Denmark/60295/2006	National Veterinary Institute	Statens Serum Institute	Bragstad,K.; Fomsgaard,A.; Jorgensen,P.H.; Hammer,A.S.; Kabell,S.; Handberg,K.
EPI241648	HA	Denmark	2006-Apr-28	A/great crested grebe/Denmark/7498/2006	Technical University of Denmark	Statens Serum Institute	Bragstad,K.; Fomsgaard,A.; Jorgensen,P.H.; Hammer,A.S.; Kabell,S.; Handberg,K.
EPI241640	HA	Denmark	2006-Mar-15	A/buzzard/Denmark/6370/2006	National Veterinary Institute	Statens Serum Institute	Bragstad,K.; Fomsgaard,A.; Jorgensen,P.H.; Hammer,A.S.; Kabell,S.; Handberg,K.
EPI162921	HA	Germany	2007-Jan-01	A/great crested grebe/Germany/R1406/07		Friedrich-Loeffler-Institut	
EPI284591	HA	Vietnam	2008-Jan-01	A/civet/Vietnam/NCVD-004/2008	National Centre of Veterinary Diagnostics	Centers for Disease Control and Prevention	Davis,Todd; Rivailler,Pierre; Nguyen,Tung
EPI530063	HA	China	2013-Dec-02	A/environment/Shenzhen/25-24/2013		BGI Shenzhen	Jinquan,Cheng;Renli,Zhang;Shisong,Fang
EPI464565	HA	China	2013-Apr-16	A/Environment/Huzhou/C291-7/2013	Huzhou Center for Disease Control and Prevention	Jiangsu University	Han, J. ; Wang, L. ; Liu, J. ; Jin, M. ; Zhang, C. ; Lan, K
EPI464564	HA	China	2013-Apr-16	A/Environment/Huzhou/C291-6/2013	Huzhou Center for Disease Control and Prevention	Jiangsu University	Han, J. ; Wang, L. ; Liu, J. ; Jin, M. ; Zhang, C. ; Lan, K
EPI464558	HA	China	2013-Apr-16	A/Environment/Huzhou/C291-10/2013	Huzhou Center for Disease Control and Prevention	Jiangsu University	Han, J. ; Wang, L. ; Liu, J. ; Jin, M. ; Zhang, C. ; Lan, K
EPI448263	HA	Bangladesh	2012-Feb-28	A/environment/Bangladesh/1019-G/2012	Institute of Epidemiology Disease Control and Research (IEDCR) & Bangladesh National Influenza Centre (NIC)	Centers for Disease Control and Prevention	Gerloff, Nancy; Simpson, Natosha; Poh, Mee; Davis, Todd
EPI448247	HA	Bangladesh	2011-Dec-28	A/environment/Bangladesh/1011/2011	Institute of Epidemiology Disease Control and Research (IEDCR) & Bangladesh National Influenza Centre (NIC)	Centers for Disease Control and Prevention	Gerloff, Nancy; Simpson, Natosha; Poh, Mee;Davis, Todd
EPI448239	HA	Bangladesh	2011-Sep-29	A/environment/Bangladesh/1018/2011	Institute of Epidemiology Disease Control and Research (IEDCR) & Bangladesh National Influenza Centre (NIC)	Centers for Disease Control and Prevention	Gerloff, Nancy; Simpson, Natosha; Poh, Mee;Davis, Todd
EPI280280	HA	China	2009-Jan-16	A/water/Hunan/7/2009	WHO Chinese National Influenza Center	WHO Chinese National Influenza Center	Lan,Yu,Dong,Libo,Li,Zi,Li,Xiyan,Zhao,Xiang,Cheng,Yanhui,Tan,minju,Yang,Lei,Zou,Shumei,Wen,Leying,Wang,Dayan,Li,Dexin,Shu,Yuelong
EPI280272	HA	China	2009-Jan-16	A/water/Hunan/3/2009	WHO Chinese National Influenza Center	WHO Chinese National Influenza Center	Lan,Yu,Dong,Libo,Li,Zi,Li,Xiyan,Zhao,Xiang,Cheng,Yanhui,Tan,minju,Yang,Lei,Zou,Shumei,Wen,Leying,Wang,Dayan,Li,Dexin,Shu,Yuelong
EPI280264	HA	China	2009-Jan-16	A/environment/Xinjiang/6/2009	WHO Chinese National Influenza Center	WHO Chinese National Influenza Center	Lan,Yu,Dong,Libo,Li,Zi,Li,Xiyan,Zhao,Xiang,Cheng,Yanhui,Tan,minju,Yang,Lei,Zou,Shumei,Wen,Leying,Wang,Dayan,Li,Dexin,Shu,Yuelong
EPI280248	HA	China	2009-Jan-16	A/environment/Guizhou/9/2009	WHO Chinese National Influenza Center	WHO Chinese National Influenza Center	Lan,Yu,Dong,Libo,Li,Zi,Li,Xiyan,Zhao,Xiang,Cheng,Yanhui,Tan,minju,Yang,Lei,Zou,Shumei,Wen,Leying,Wang,Dayan,Li,Dexin,Shu,Yuelong
EPI280240	HA	China	2009-Jan-16	A/environment/Guizhou/7/2009	WHO Chinese National Influenza Center	WHO Chinese National Influenza Center	Lan,Yu,Dong,Libo,Li,Zi,Li,Xiyan,Zhao,Xiang,Cheng,Yanhui,Tan,minju,Yang,Lei,Zou,Shumei,Wen,Leying,Wang,Dayan,Li,Dexin,Shu,Yuelong
EPI280224	HA	China	2009-Jan-16	A/environment/Guizhou/2/2009	WHO Chinese National Influenza Center	WHO Chinese National Influenza Center	Lan,Yu,Dong,Libo,Li,Zi,Li,Xiyan,Zhao,Xiang,Cheng,Yanhui,Tan,minju,Yang,Lei,Zou,Shumei,Wen,Leying,Wang,Dayan,Li,Dexin,Shu,Yuelong
EPI280216	HA	China	2009-Jan-06	A/duck feces/Hebei/5/2009	WHO Chinese National Influenza Center	WHO Chinese National Influenza Center	Lan,Yu,Dong,Libo,Li,Zi,Li,Xiyan,Zhao,Xiang,Cheng,Yanhui,Tan,minju,Yang,Lei,Zou,Shumei,Wen,Leying,Wang,Dayan,Li,Dexin,Shu,Yuelong
EPI280200	HA	China	2009-Jan-06	A/water/Hebei/2/2009	WHO Chinese National Influenza Center	WHO Chinese National Influenza Center	Lan,Yu,Dong,Libo,Li,Zi,Li,Xiyan,Zhao,Xiang,Cheng,Yanhui,Tan,minju,Yang,Lei,Zou,Shumei,Wen,Leying,Wang,Dayan,Li,Dexin,Shu,Yuelong
EPI280192	HA	China	2009-Jan-06	A/water/Hebei/1/2009	WHO Chinese National Influenza Center	WHO Chinese National Influenza Center	Lan,Yu,Dong,Libo,Li,Zi,Li,Xiyan,Zhao,Xiang,Cheng,Yanhui,Tan,minju,Yang,Lei,Zou,Shumei,Wen,Leying,Wang,Dayan,Li,Dexin,Shu,Yuelong
EPI553362	HA	Japan	2014-Dec-01	A/environment/Kagoshima/KU-ngr-H/2014	Kagoshima University	Kagoshima University	
EPI560202	HA	China	2015-Jan-05	A/enviroment/Henan/SMX1/2015(H5N1)	Institute of Pathogen Biology, Taishan Medical College	Institute of Microbiology, Chinese Academy of Sciences	Shi, Weifeng; Bi, Yuhai
EPI537680	HA	Indonesia	2012-Nov-08	A/Environment/East Java/LBM-LM13/2012	BBalitvet	BBalitvet	Dharmayanti,Ni Luh Putu Indi; Hartawan, Risza
EPI537677	HA	Indonesia	2013-Jun-25	A/Environment/West Java/Bksi34/2013	BBalitvet	BBalitvet	Dharmayanti, NiLuh Putu Indi; Hartawan, R; Hewajuli, D
EPI432533	HA	China	2010-Apr-27	r8	Harbin Veterinary Research Institute	Harbin Veterinary Research Institute	
EPI432525	HA	China	2010-Apr-27	r78	Harbin Veterinary Research Institute	Harbin Veterinary Research Institute	
EPI432485	HA	China	2010-Apr-21	r6	Harbin Veterinary Research Institute	Harbin Veterinary Research Institute	
EPI432469	HA	China	2010-Apr-21	r578	Harbin Veterinary Research Institute	Harbin Veterinary Research Institute	
EPI432461	HA	China	2010-Apr-21	r57	Harbin Veterinary Research Institute	Harbin Veterinary Research Institute	
EPI432453	HA	China	2010-Apr-21	r568	Harbin Veterinary Research Institute	Harbin Veterinary Research Institute	
EPI432445	HA	China	2010-Apr-21	r5678	Harbin Veterinary Research Institute	Harbin Veterinary Research Institute	
EPI432429	HA	China	2010-Apr-16	r56	Harbin Veterinary Research Institute	Harbin Veterinary Research Institute	
EPI432421	HA	China	2010-Apr-16	r5	Harbin Veterinary Research Institute	Harbin Veterinary Research Institute	
EPI432405	HA	China	2010-Apr-16	r378	Harbin Veterinary Research Institute	Harbin Veterinary Research Institute	
EPI432397	HA	China	2010-Apr-16	r37	Harbin Veterinary Research Institute	Harbin Veterinary Research Institute	
EPI432389	HA	China	2010-Apr-16	r368	Harbin Veterinary Research Institute	Harbin Veterinary Research Institute	
EPI432381	HA	China	2010-Apr-10	r3678	Harbin Veterinary Research Institute	Harbin Veterinary Research Institute	
EPI432373	HA	China	2010-Apr-10	r367	Harbin Veterinary Research Institute	Harbin Veterinary Research Institute	
EPI432349	HA	China	2010-Apr-10	r3578	Harbin Veterinary Research Institute	Harbin Veterinary Research Institute	
EPI432341	HA	China	2010-Apr-03	r357	Harbin Veterinary Research Institute	Harbin Veterinary Research Institute	
EPI432317	HA	China	2010-Apr-03	r3567	Harbin Veterinary Research Institute	Harbin Veterinary Research Institute	
EPI432309	HA	China	2010-Apr-03	r356	Harbin Veterinary Research Institute	Harbin Veterinary Research Institute	
EPI432301	HA	China	2010-Apr-03	r35	Harbin Veterinary Research Institute	Harbin Veterinary Research Institute	
EPI432293	HA	China	2010-Apr-03	r3	Harbin Veterinary Research Institute	Harbin Veterinary Research Institute	
EPI432285	HA	China	2010-Mar-28	r28	Harbin Veterinary Research Institute	Harbin Veterinary Research Institute	
EPI432269	HA	China	2010-Mar-28	r27	Harbin Veterinary Research Institute	Harbin Veterinary Research Institute	
EPI432261	HA	China	2010-Mar-28	r268	Harbin Veterinary Research Institute	Harbin Veterinary Research Institute	
EPI432253	HA	China	2010-Mar-28	r2678	Harbin Veterinary Research Institute	Harbin Veterinary Research Institute	
EPI432245	HA	China	2010-Mar-28	r267	Harbin Veterinary Research Institute	Harbin Veterinary Research Institute	
EPI432237	HA	China	2010-Mar-22	r26	Harbin Veterinary Research Institute	Harbin Veterinary Research Institute	
EPI432229	HA	China	2010-Mar-22	r258	Harbin Veterinary Research Institute	Harbin Veterinary Research Institute	
EPI432221	HA	China	2010-Mar-22	r2578	Harbin Veterinary Research Institute	Harbin Veterinary Research Institute	
EPI432189	HA	China	2010-Mar-17	r2567	Harbin Veterinary Research Institute	Harbin Veterinary Research Institute	
EPI432181	HA	China	2010-Mar-17	r256	Harbin Veterinary Research Institute	Harbin Veterinary Research Institute	
EPI432173	HA	China	2010-Mar-17	r25	Harbin Veterinary Research Institute	Harbin Veterinary Research Institute	
EPI432165	HA	China	2010-Mar-17	r238	Harbin Veterinary Research Institute	Harbin Veterinary Research Institute	
EPI432149	HA	China	2010-Mar-17	r237	Harbin Veterinary Research Institute	Harbin Veterinary Research Institute	
EPI432141	HA	China	2010-Mar-12	r2368	Harbin Veterinary Research Institute	Harbin Veterinary Research Institute	
EPI432133	HA	China	2010-Mar-12	r23678	Harbin Veterinary Research Institute	Harbin Veterinary Research Institute	
EPI432125	HA	China	2010-Mar-12	r2367	Harbin Veterinary Research Institute	Harbin Veterinary Research Institute	
EPI432117	HA	China	2010-Mar-12	r236	Harbin Veterinary Research Institute	Harbin Veterinary Research Institute	
EPI432085	HA	China	2010-Mar-07	r23568	Harbin Veterinary Research Institute	Harbin Veterinary Research Institute	
EPI432077	HA	China	2010-Mar-07	r235678	Harbin Veterinary Research Institute	Harbin Veterinary Research Institute	
EPI432069	HA	China	2010-Mar-07	r23567	Harbin Veterinary Research Institute	Harbin Veterinary Research Institute	
EPI432061	HA	China	2010-Mar-07	r2356	Harbin Veterinary Research Institute	Harbin Veterinary Research Institute	
EPI432037	HA	China	2010-Mar-02	r2	Harbin Veterinary Research Institute	Harbin Veterinary Research Institute	
EPI432029	HA	China	2010-Mar-02	r18	Harbin Veterinary Research Institute	Harbin Veterinary Research Institute	
EPI432005	HA	China	2010-Mar-02	r168	Harbin Veterinary Research Institute	Harbin Veterinary Research Institute	
EPI431989	HA	China	2012-Feb-25	r167	Harbin Veterinary Research Institute	Harbin Veterinary Research Institute	

EPI431981	HA	China	2012-Feb-25	r16	Harbin Veterinary Research Institute	Harbin Veterinary Research Institute	
EPI431973	HA	China	2012-Feb-25	r158	Harbin Veterinary Research Institute	Harbin Veterinary Research Institute	
EPI431949	HA	China	2010-Feb-18	r1568	Harbin Veterinary Research Institute	Harbin Veterinary Research Institute	
EPI431941	HA	China	2010-Feb-18	r15678	Harbin Veterinary Research Institute	Harbin Veterinary Research Institute	
EPI431933	HA	China	2010-Feb-18	r1567	Harbin Veterinary Research Institute	Harbin Veterinary Research Institute	
EPI431925	HA	China	2010-Feb-18	r156	Harbin Veterinary Research Institute	Harbin Veterinary Research Institute	
EPI431909	HA	China	2010-Feb-18	r138	Harbin Veterinary Research Institute	Harbin Veterinary Research Institute	
EPI431901	HA	China	2010-Feb-10	r1378	Harbin Veterinary Research Institute	Harbin Veterinary Research Institute	
EPI431885	HA	China	2010-Feb-10	r1368	Harbin Veterinary Research Institute	Harbin Veterinary Research Institute	
EPI431869	HA	China	2010-Feb-10	r1367	Harbin Veterinary Research Institute	Harbin Veterinary Research Institute	
EPI431845	HA	China	2010-Feb-05	r13578	Harbin Veterinary Research Institute	Harbin Veterinary Research Institute	
EPI431837	HA	China	2010-Feb-05	r1357	Harbin Veterinary Research Institute	Harbin Veterinary Research Institute	
EPI431829	HA	China	2010-Feb-05	r13568	Harbin Veterinary Research Institute	Harbin Veterinary Research Institute	
EPI431821	HA	China	2010-Feb-05	r135678	Harbin Veterinary Research Institute	Harbin Veterinary Research Institute	
EPI431805	HA	China	2010-Jan-31	r1356	Harbin Veterinary Research Institute	Harbin Veterinary Research Institute	
EPI431789	HA	China	2010-Jan-31	r13	Harbin Veterinary Research Institute	Harbin Veterinary Research Institute	
EPI431781	HA	China	2010-Jan-31	r128	Harbin Veterinary Research Institute	Harbin Veterinary Research Institute	
EPI431765	HA	China	2010-Jan-31	r127	Harbin Veterinary Research Institute	Harbin Veterinary Research Institute	
EPI431741	HA	China	2010-Jan-26	r1267	Harbin Veterinary Research Institute	Harbin Veterinary Research Institute	
EPI431733	HA	China	2010-Jan-26	r126	Harbin Veterinary Research Institute	Harbin Veterinary Research Institute	
EPI431725	HA	China	2010-Jan-26	r1258	Harbin Veterinary Research Institute	Harbin Veterinary Research Institute	
EPI431717	HA	China	2010-Jan-26	r12578	Harbin Veterinary Research Institute	Harbin Veterinary Research Institute	
EPI431709	HA	China	2010-Jan-20	r1257	Harbin Veterinary Research Institute	Harbin Veterinary Research Institute	
EPI431701	HA	China	2010-Jan-20	r12568	Harbin Veterinary Research Institute	Harbin Veterinary Research Institute	
EPI431685	HA	China	2010-Jan-20	r12567	Harbin Veterinary Research Institute	Harbin Veterinary Research Institute	
EPI431645	HA	China	2010-Jan-16	r1237	Harbin Veterinary Research Institute	Harbin Veterinary Research Institute	
EPI431621	HA	China	2010-Jan-16	r12367	Harbin Veterinary Research Institute	Harbin Veterinary Research Institute	
EPI431613	HA	China	2010-Jan-10	r1236	Harbin Veterinary Research Institute	Harbin Veterinary Research Institute	
EPI431597	HA	China	2010-Jan-10	r123578	Harbin Veterinary Research Institute	Harbin Veterinary Research Institute	
EPI431589	HA	China	2010-Jan-10	r12357	Harbin Veterinary Research Institute	Harbin Veterinary Research Institute	
EPI431581	HA	China	2010-Jan-10	r123568	Harbin Veterinary Research Institute	Harbin Veterinary Research Institute	
EPI431557	HA	China	2010-Jan-04	r12356	Harbin Veterinary Research Institute	Harbin Veterinary Research Institute	
EPI431541	HA	China	2010-Jan-04	r123	Harbin Veterinary Research Institute	Harbin Veterinary Research Institute	
EPI431533	HA	China	2010-Jan-04	r12	Harbin Veterinary Research Institute	Harbin Veterinary Research Institute	
EPI338347	HA	Germany	2009-Jan-01	A/hen's egg/Germany/[A/cygnus cygnus/Germany/R65/2006]-CoJ50-control/2009 (H5N1)	Friedrich-Loeffler-Institut	Friedrich-Loeffler-Institut	Kalthoff,Donata ;Röhrs,Susanne ;Höper,Dirk ;Hoffmann,Bernd ;Bogs,Jessica ;Stech,Jürgen ;Beer,Martin
EPI309750	HA	Germany	2008-Jan-01	A/cygnus cygnus/Germany/R65.1/2006 (H5N1)	Friedrich-Loeffler-Institut	Friedrich-Loeffler-Institut	Hoeper,Dirk; Kalthoff,Donata; Hoffmann,Bernd; Beer,Martin
EPI287340	HA	Germany	2009-Jan-01	A/MDCK/Germany/[A/cygnus cygnus/Germany/R65/2006]-CoQ100-control/2009 (H5N1)	Friedrich-Loeffler-Institut	Friedrich-Loeffler-Institut	Hoeper,Dirk; Kalthoff,Donata; Hoffmann,Bernd; Beer,Martin
EPI287332	HA	Germany	2009-Jan-01	A/MDCK/Germany/[A/cygnus cygnus/Germany/R65/2006]-Q100c-escape/2009 (H5N1)	Friedrich-Loeffler-Institut	Friedrich-Loeffler-Institut	Hoeper,Dirk; Kalthoff,Donata; Hoffmann,Bernd; Beer,Martin
EPI287324	HA	Germany	2009-Jan-01	A/MDCK/Germany/[A/cygnus cygnus/Germany/R65/2006]-QQ100a-escape/2009 (H5N1)	Friedrich-Loeffler-Institut	Friedrich-Loeffler-Institut	Hoeper,Dirk; Kalthoff,Donata; Hoffmann,Bernd; Beer,Martin
EPI287316	HA	Germany	2009-Jan-01	A/MDCK/Germany/[A/cygnus cygnus/Germany/R65/2006]-QQ100b-escape/2009 (H5N1)	Friedrich-Loeffler-Institut	Friedrich-Loeffler-Institut	Hoeper,Dirk; Kalthoff,Donata; Hoffmann,Bernd; Beer,Martin
EPI287308	HA	Germany	2009-Jan-01	A/MDCK/Germany/[A/cygnus cygnus/Germany/R65/2006]-Q100b-escape/2009 (H5N1)	Friedrich-Loeffler-Institut	Friedrich-Loeffler-Institut	Hoeper,Dirk; Kalthoff,Donata; Hoffmann,Bernd; Beer,Martin
EPI287276	HA	Germany	2008-Jan-01	A/MDCK/Germany/[A/cygnus cygnus/Germany/R65/2006]-CoP50-control/2008 (H5N1)	Friedrich-Loeffler-Institut	Friedrich-Loeffler-Institut	Hoeper,Dirk; Kalthoff,Donata; Hoffmann,Bernd; Beer,Martin
EPI287268	HA	Germany	2009-Jan-01	A/MDCK/Germany/[A/cygnus cygnus/Germany/R65/2006]-CoP100-control/2009 (H5N1)	Friedrich-Loeffler-Institut	Friedrich-Loeffler-Institut	Hoeper,Dirk; Kalthoff,Donata; Hoffmann,Bernd; Beer,Martin
EPI287260	HA	Germany	2009-Jan-01	A/MDCK/Germany/[A/cygnus cygnus/Germany/R65/2006]-P100c-escape/2009 (H5N1)	Friedrich-Loeffler-Institut	Friedrich-Loeffler-Institut	Hoeper,Dirk; Kalthoff,Donata; Hoffmann,Bernd; Beer,Martin
EPI287244	HA	Germany	2009-Jan-01	A/MDCK/Germany/[A/cygnus cygnus/Germany/R65/2006]-PP100b-escape/2009 (H5N1)	Friedrich-Loeffler-Institut	Friedrich-Loeffler-Institut	Hoeper,Dirk; Kalthoff,Donata; Hoffmann,Bernd; Beer,Martin
EPI287236	HA	Germany	2009-Jan-01	A/MDCK/Germany/[A/cygnus cygnus/Germany/R65/2006]-P100b-escape/2009 (H5N1)	Friedrich-Loeffler-Institut	Friedrich-Loeffler-Institut	Hoeper,Dirk; Kalthoff,Donata; Hoffmann,Bernd; Beer,Martin
EPI287228	HA	Germany	2008-Jan-01	A/MDCK/Germany/[A/cygnus cygnus/Germany/R65/2006]-P50-escape/2008 (H5N1)	Friedrich-Loeffler-Institut	Friedrich-Loeffler-Institut	Hoeper,Dirk; Kalthoff,Donata; Hoffmann,Bernd; Beer,Martin
EPI287212	HA	Germany	2008-Jan-01	A/MDCK/Germany/[A/cygnus cygnus/Germany/R65/2006]-P18-escape/2008 (H5N1)	Friedrich-Loeffler-Institut	Friedrich-Loeffler-Institut	Hoeper,Dirk; Kalthoff,Donata; Hoffmann,Bernd; Beer,Martin
EPI174805	HA	Egypt	2007-Jan-01	A/Egypt/321/2007(H5N1)-PR8-IDCDC-RG11		Centers for Disease Control and Prevention	
EPI643072	HA	Indonesia	2015-Mar-25	A/Indonesia/NIHRD15028/2015	National Institute of Health Research and Development	National Institute of Health Research and Development	HA,Pawestri;KD,Puspa;HD,Ikawati;AA,Nugraha;V,Setiawaty
EPI643069	HA	Indonesia	2015-Mar-24	A/Indonesia/NIHRD/15023/2015	National Institute of Health Research and Development	National Institute of Health Research and Development	HA,Pawestri;AA,Nugraha;KD,Puspa;HD,Ikawati;V,Setiawaty
EPI639614	HA	Vietnam	2013-Nov-15	A/Vietnam/VP39/2013	Pasteur Institute, Influenza Laboratory	Centers for Disease Control and Prevention	
EPI639606	HA	Vietnam	2012-Feb-23	A/Vietnam/CD12-76/2012	Pasteur Institute, Influenza Laboratory	Centers for Disease Control and Prevention	
EPI624930	HA	Vietnam	2013-Apr-04	A/Vietnam/VP13-28H/2013	Pasteur Institute of Ho Chi Minh City	National Institute of Infectious Diseases (NIID)	Ikuyo, Takayama; Nguyen Trung, Hieu; Masayuki, Shirakura; Mina, Nakauchi; Seiichiro, Fujisaki; Hitoshi, Takahashi; Shiho, Nagata; Nguyen Thanh, Long; Takato, Odagiri; Masato, Tashiro; Tsutomu, Kageyama

EPI624922	HA	Vietnam	2014-Jan-28	A/Vietnam/14012902/2014	Pasteur Institute of Ho Chi Minh City	National Institute of Infectious Diseases (NIID)	Ikuyo, Takayama; Nguyen Trung, Hieu; Masayuki, Shirakura; Mina, Nakauchi; Seiichiro, Fujisaki; Hitoshi, Takahashi; Shiho, Nagata; Nguyen Thanh, Long; Takato, Odagiri; Masato, Tashiro; Tsutomu, Kageyama
EPI624914	HA	Vietnam	2014-Jan-18	A/Vietnam/14011801/2014	Pasteur Institute of Ho Chi Minh City	National Institute of Infectious Diseases (NIID)	Ikuyo, Takayama; Nguyen Trung, Hieu; Masayuki, Shirakura; Mina, Nakauchi; Seiichiro, Fujisaki; Hitoshi, Takahashi; Shiho, Nagata; Nguyen Thanh, Long; Takato, Odagiri; Masato, Tashiro; Tsutomu, Kageyama
EPI547699	HA	Indonesia	2014-Jun-06	A/Indonesia/NIHRD14157/2014	National Institute of Health Research and Development	National Institute of Health Research and Development	HA,Pawestri;AA,Nugraha;V,Setiawaty
EPI537618	HA	Egypt	2014-Mar-07	A/Egypt/N01754/2014	Ministry of Health and Population	NAMRU-3	Younan, M.; Amir, E.; Naguib, A.; El Gohary, A.; Kandeel, A.; Defang, G.
EPI537617	HA	Egypt	2014-Mar-06	A/Egypt/N01753/2014	Ministry of Health and Population	NAMRU-3	Younan, M.; Amir, E.; Naguib, A.; El Gohary, A.; Kandeel, A.; Defang, G
EPI531959	HA	Indonesia	2014-Apr-19	A/Indonesia/NIHRD14122/2014	National Institute of Health Research and Development	National Institute of Health Research and Development	HA,Pawestri;AA,Nugraha;KD,Puspa;HD,Ikawati;V,Setiawaty
EPI509937	HA	Indonesia	2013-Nov-11	A/Indonesia/NIHRD13269/2013	National Institute of Health Research and Development	National Institute of Health Research and Development	HA,Pawestri;KD,Puspa;HD,Ikawati;AA,Nugraha;V,Setiawaty
EPI500771	HA	Canada	2014-Jan-03	A/Alberta/01/2014	Provincial Laboratory of Public Health for Southern Alberta	Public Health Agency of Canada (PHAC)	Li, Yan; Bastien, Nathalie; Fonseca, Kevin; Tipples, Graham; Pabbaraju, Kanti; Tellier, Raymond; Wong, Sallene; Tang, Julian W.; Drews, Steven J.
EPI492524	HA	Indonesia	2013-Sep-27	A/Indonesia/NIHRD13233/2013	National Institute of Health Research and Development	National Institute of Health Research and Development	HA,Pawestri;HD,Ikawati;KD,Puspa;AA,Nugraha;V,Setiawaty
EPI489378	HA	Indonesia	2011-Apr-08	A/Indonesia/NIHRD11454/2011	National Institute of Health Research and Development	National Institute of Health Research and Development	HA,Pawestri
EPI487461	HA	Indonesia	2010-Apr-25	A/Indonesia/NIHRD10364/2010	National Institute of Health Research and Development	National Institute of Health Research and Development	HA,Pawestri
EPI485587	HA	Cambodia	2013-Aug-09	A/Cambodia/X0810301/2013	Institut Pasteur in Cambodia	Institut Pasteur in Cambodia	Rith, S.; Horm, SV.; Buchy, P.
EPI460329	HA	Vietnam	2012-Jan-14	A/Vietnam/VP12-3/2012	Pasteur Institute, Influenza Laboratory	Centers for Disease Control and Prevention	
EPI442763	HA	Indonesia	2012-Dec-06	A/Indonesia/NIHRD12550/2012	National Institute of Health Research and Development	National Institute of Health Research and Development	Pawestri,H.A;Puspa,K.D;Setiawaty,V
EPI442759	HA	Indonesia	2012-Jun-29	A/Indonesia/NIHRD12377/2012	National Institute of Health Research and Development	National Institute of Health Research and Development	Pawestri,H.A;Ikawati,H.D;Setiawaty,V
EPI442757	HA	Indonesia	2012-Feb-29	A/Indonesia/NIHRD12162/2012	National Institute of Health Research and Development	National Institute of Health Research and Development	Pawestri,H.A;Nugraha,A.A;Setiawaty,V
EPI420391	HA	China	2013-Feb-09	A/Guizhou/2/2013	WHO Chinese National Influenza Center	China National Influenza Centre	
EPI420386	HA	China	2013-Feb-08	A/Guizhou/1/2013	WHO Chinese National Influenza Center	China National Influenza Centre	
EPI375432	HA	Hong Kong (SAR)	2012-May-28	A/Hong Kong/5923/2012	Public Health Laboratory Services Branch, Centre for Health Protection	Public Health Laboratory Services Branch, Centre for Health Protection	Mak,G.C.; Cheng,P.K.C.; Lo,J.Y.C.
EPI373023	HA	Egypt	2010-Jan-01	A/Egypt/17692/2010	U.S. Naval Medical Research Unit No.3	Centers for Disease Control and Prevention	Younan, M.; Elassal, E.; ElBadry, M.A.; Naguib, A.;Saied, I.; Kandeel, A.; Cornelius, C.
EPI373022	HA	Egypt	2010-Jan-01	A/Egypt/N09407/2010	U.S. Naval Medical Research Unit No.3	Centers for Disease Control and Prevention	Younan, M.; Elassal, E.; ElBadry, M.A.; Naguib, A.;Saied, I.; Kandeel, A.; Cornelius, C.
EPI373021	HA	Egypt	2010-Jan-01	A/Egypt/N16789/2010	U.S. Naval Medical Research Unit No.3	Centers for Disease Control and Prevention	Younan, M.; Elassal, E.; ElBadry, M.A.; Naguib, A.;Saied, I.; Kandeel, A.; Cornelius, C.
EPI373020	HA	Egypt	2010-Jan-01	A/Egypt/N08932/2010	U.S. Naval Medical Research Unit No.3	Centers for Disease Control and Prevention	Younan, M.; Elassal, E.; ElBadry, M.A.; Naguib, A.;Saied, I.; Kandeel, A.; Cornelius, C.
EPI373014	HA	Egypt	2011-Jan-01	A/Egypt/N04287/2011	U.S. Naval Medical Research Unit No.3	Centers for Disease Control and Prevention	Younan, M.; Elassal, E.; ElBadry, M.A.; Naguib, A.;Saied, I.; Kandeel, A.; Cornelius, C.
EPI373013	HA	Egypt	2011-Jan-01	A/Egypt/N00166/2011	U.S. Naval Medical Research Unit No.3	Centers for Disease Control and Prevention	Younan, M.; Elassal, E.; ElBadry, M.A.; Naguib, A.;Saied, I.; Kandeel, A.; Cornelius, C.
EPI373012	HA	Egypt	2011-Jan-01	A/Egypt/N04286/2011	U.S. Naval Medical Research Unit No.3	Centers for Disease Control and Prevention	Younan, M.; Elassal, E.; ElBadry, M.A.; Naguib, A.;Saied, I.; Kandeel, A.; Cornelius, C.
EPI373011	HA	Egypt	2011-Jan-01	A/Egypt/N04288/2011	U.S. Naval Medical Research Unit No.3	Centers for Disease Control and Prevention	Younan, M.; Elassal, E.; ElBadry, M.A.; Naguib, A.;Saied, I.; Kandeel, A.; Cornelius, C.
EPI372963	HA	Egypt	2011-Oct-30	A/Egypt/N10621/2011	U.S. Naval Medical Research Unit No.3	Centers for Disease Control and Prevention	Younan, M.; Poh, M.K.; Elassal, E.; Simpson, N.; Jones,J.; ElBadry,M.A.; Naguib, A.;Saied, I.; Kandeel,A.; Cornelius, C.
EPI372939	HA	Egypt	2011-Dec-01	A/Egypt/N11126/2011	U.S. Naval Medical Research Unit No.3	Centers for Disease Control and Prevention	Younan, M.; Poh, M.K.; Elassal, E.; Simpson, N.; Jones,J.; ElBadry,M.A.; Naguib, A.;Saied, I.; Kandeel,A.; Cornelius, C.
EPI372923	HA	Egypt	2011-Jan-01	A/Egypt/N7724/2011	U.S. Naval Medical Research Unit No.3	Centers for Disease Control and Prevention	Younan, M.; Poh, M.K.; Elassal, E.; Simpson, N.; Jones,J.; ElBadry,M.A.; Naguib, A.;Saied, I.; Kandeel,A.; Cornelius, C.
EPI372907	HA	Egypt	2011-Jan-01	A/Egypt/N7562/2011	U.S. Naval Medical Research Unit No.3	Centers for Disease Control and Prevention	Younan, M.; Poh, M.K.; Elassal, E.; Simpson, N.; Jones,J.; ElBadry,M.A.; Naguib, A.;Saied, I.; Kandeel,A.; Cornelius, C.
EPI372883	HA	Egypt	2011-Jan-01	A/Egypt/N6774/2011	U.S. Naval Medical Research Unit No.3	Centers for Disease Control and Prevention	Younan, M.; Poh, M.K.; Elassal, E.; Simpson, N.; Jones,J.; ElBadry,M.A.; Naguib, A.;Saied, I.; Kandeel,A.; Cornelius, C.
EPI372859	HA	Egypt	2011-Jan-01	A/Egypt/N6322/2011	U.S. Naval Medical Research Unit No.3	Centers for Disease Control and Prevention	Younan, M.; Poh, M.K.; Elassal, E.; Simpson, N.; Jones,J.; ElBadry,M.A.; Naguib, A.;Saied, I.; Kandeel,A.; Cornelius, C.
EPI372851	HA	Egypt	2011-Jan-01	A/Egypt/N0544/2011	U.S. Naval Medical Research Unit No.3	Centers for Disease Control and Prevention	Younan, M.; Poh, M.K.; Elassal, E.; Simpson, N.; Jones,J.; ElBadry,M.A.; Naguib, A.;Saied, I.; Kandeel,A.; Cornelius, C.
EPI372715	HA	Egypt	2009-May-18	A/Egypt/9538-NAMRU3/2009	U.S. Naval Medical Research Unit No.3	Centers for Disease Control and Prevention	Younan, M.; Poh, M.K.; Elassal, E.; Simpson, N.; Jones,J.; ElBadry,M.A.; Naguib, A.;Saied, I.; Kandeel,A.; Cornelius, C.
EPI397642	HA	Egypt	2007-Mar-13	A/Egypt/321-NAMRU3/2007	U.S. Naval Medical Research Unit No.3	Centers for Disease Control and Prevention	Younan, M.; Poh, M.K.; Elassal, E.; Simpson, N.; Jones,J.; ElBadry,M.A.; Naguib, A.;Saied, I.; Kandeel,A.; Cornelius, C.
EPI347615	HA	Vietnam	2004-Jan-01	A/Viet Nam/1194/2004	Queen Mary Hospital	National Institute for Biological Standards and Control (NIBSC)	Nicolson,Carolyn; Johnson,Rachel E; Harvey,Ruth; Robertson,James S; Engelhardt,Othmar G
EPI347304	HA	China	2011-Dec-28	A/Guangdong-Shenzhen/1/2011(H5N1)	WHO Chinese National Influenza Center	WHO Chinese National Influenza Center	Gao,Rongbao;Yang,Shuai;Wen,Leying; Zhou,Shumei; Yang,Lei; Li, Ming; Shu,Yuelong
EPI341632	HA	Indonesia	2011-Oct-07	A/Indonesia/NIHRD11767/2011(H5N1)	National Institute of Health Research and Development	National Institute of Health Research and Development	Pawestri,H.A;Setiawaty,V;Sampurno,O.D;Takamaya,I;Shirakura,M;Kagayama,T;Tashiro,M
EPI280176	HA	China	2009-Jan-23	A/Hunan/2/2009	WHO Chinese National Influenza Center	WHO Chinese National Influenza Center	Lan,Yu,Dong,Libo,Li,Zi,Li,Xiyan,Zhao,Xiang,Cheng,Yanhui,Tan,minju,Yang,Lei,Zou,Shumei,Wen,Leying,Wang,Dayan,Li,Dexin,Shu,Yuelong
EPI280136	HA	China	2007-Feb-18	A/Fujian/1/2007	WHO Chinese National Influenza Center	WHO Chinese National Influenza Center	Lan,Yu,Dong,Libo,Li,Zi,Li,Xiyan,Zhao,Xiang,Cheng,Yanhui,Tan,minju,Yang,Lei,Zou,Shumei,Wen,Leying,Wang,Dayan,Li,Dexin,Shu,Yuelong
EPI280128	HA	China	2006-Jun-19	A/Xinjiang/1/2006	WHO Chinese National Influenza Center	WHO Chinese National Influenza Center	Lan,Yu,Dong,Libo,Li,Zi,Li,Xiyan,Zhao,Xiang,Cheng,Yanhui,Tan,minju,Yang,Lei,Zou,Shumei,Wen,Leying,Wang,Dayan,Li,Dexin,Shu,Yuelong
EPI280096	HA	China	2006-Jan-27	A/Hunan/1/2006	WHO Chinese National Influenza Center	WHO Chinese National Influenza Center	Lan,Yu,Dong,Libo,Li,Zi,Li,Xiyan,Zhao,Xiang,Cheng,Yanhui,Tan,minju,Yang,Lei,Zou,Shumei,Wen,Leying,Wang,Dayan,Li,Dexin,Shu,Yuelong
EPI267032	HA	China	2010-Jun-01	A/Hubei/1/2010		WHO Chinese National Influenza Center	Yu Lan,Wei Wang,Shumei Zou,Zi Li,Leying Wen,Xiaodan Li,Libo Dong,Dexin Li,Yuelong Shu
EPI280032	HA	China	2006-Dec-10	A/Anhui/1/2006	WHO Chinese National Influenza Center	WHO Chinese National Influenza Center	Lan,Yu; Dong,Libo; Li,Zi; Li,Xiyan; Zhao,Xiang; Cheng,Yanhui; Tan,minju; Yang,Lei; Zou,Shumei; Wen,Leying; Wang,Dayan; Li,Dexin; Shu,Yuelong
EPI280074	HA	China	2009-Jan-10	A/Xinjiang/1/2009	WHO Chinese National Influenza Center	WHO Chinese National Influenza Center	Lan,Yu; Dong,Libo; Li,Zi; Li,Xiyan; Zhao,Xiang; Cheng,Yanhui; Tan,minju; Yang,Lei; Zou,Shumei; Wen,Leying; Wang,Dayan; Li,Dexin; Shu,Yuelong
EPI280067	HA	China	2009-Jan-05	A/Shandong/1/2009	WHO Chinese National Influenza Center	WHO Chinese National Influenza Center	Lan,Yu; Dong,Libo; Li,Zi; Li,Xiyan; Zhao,Xiang; Cheng,Yanhui; Tan,minju; Yang,Lei; Zou,Shumei; Wen,Leying; Wang,Dayan; Li,Dexin; Shu,Yuelong
EPI280081	HA	China	2009-Jan-15	A/Guizhou/1/2009	WHO Chinese National Influenza Center	WHO Chinese National Influenza Center	Lan,Yu; Dong,Libo; Li,Zi; Li,Xiyan; Zhao,Xiang; Cheng,Yanhui; Tan,minju; Yang,Lei; Zou,Shumei; Wen,Leying; Wang,Dayan; Li,Dexin; Shu,Yuelong
EPI280088	HA	China	2009-Jan-19	A/Guangxi/1/2009	WHO Chinese National Influenza Center	WHO Chinese National Influenza Center	Lan,Yu; Dong,Libo; Li,Zi; Li,Xiyan; Zhao,Xiang; Cheng,Yanhui; Tan,minju; Yang,Lei; Zou,Shumei; Wen,Leying; Wang,Dayan; Li,Dexin; Shu,Yuelong
EPI280060	HA	China	2008-Feb-16	A/Guangdong/1/2008	WHO Chinese National Influenza Center	WHO Chinese National Influenza Center	Lan,Yu; Dong,Libo; Li,Zi; Li,Xiyan; Zhao,Xiang; Cheng,Yanhui; Tan,minju; Yang,Lei; Zou,Shumei; Wen,Leying; Wang,Dayan; Li,Dexin; Shu,Yuelong

EPI280053	HA	China	2008-Feb-12	A/Guangxi/1/2008	WHO Chinese National Influenza Center	WHO Chinese National Influenza Center	Lan,Yu; Dong,Libo; Li,Zi; Li,Xiyan; Zhao,Xiang; Cheng,Yanhui; Tan,minju; Yang,Lei; Zou,Shumei; Wen,Leying; Wang,Dayan; Li,Dexin; Shu,Yuelong
EPI280046	HA	China	2008-Jan-16	A/Hunan/1/2008	WHO Chinese National Influenza Center	WHO Chinese National Influenza Center	Lan,Yu; Dong,Libo; Li,Zi; Li,Xiyan; Zhao,Xiang; Cheng,Yanhui; Tan,minju; Yang,Lei; Zou,Shumei; Wen,Leying; Wang,Dayan; Li,Dexin; Shu,Yuelong
EPI280039	HA	China	2007-Mar-17	A/Anhui/1/2007	WHO Chinese National Influenza Center	WHO Chinese National Influenza Center	Lan,Yu; Dong,Libo; Li,Zi; Li,Xiyan; Zhao,Xiang; Cheng,Yanhui; Tan,minju; Yang,Lei; Zou,Shumei; Wen,Leying; Wang,Dayan; Li,Dexin; Shu,Yuelong
EPI279949	HA	China	2006-Feb-10	A/Zhejiang/1/2006	WHO Chinese National Influenza Center	WHO Chinese National Influenza Center	Lan,Yu; Dong,Libo; Li,Zi; Li,Xiyan; Zhao,Xiang; Cheng,Yanhui; Tan,minju; Yang,Lei; Zou,Shumei; Wen,Leying; Wang,Dayan; Li,Dexin; Shu,Yuelong
EPI244098	HA	Vietnam	2005-Jun-27	A/duck/Vietnam/TG24/05		Friedrich-Loeffler-Institut	
EPI181297	HA	China	2006-Feb-21	A/Zhejiang/1/2006		WHO Chinese National Influenza Center	
EPI173707	HA	Egypt	2007-Jan-01	A/Egypt/321/2007		Centers for Disease Control and Prevention	
EPI164755	HA	Indonesia	2008-Jan-01	A/Indonesia/7379/2008	Ministry of Health, NIHRD	Eijkman Institute for Molecular Biology	
EPI164258	HA	China	2005-Nov-29	A/Guangxi/1/2005		WHO Chinese National Influenza Center	
EPI164232	HA	China	2006-Jan-17	A/Sichuan/2/2006		WHO Chinese National Influenza Center	
EPI164216	HA	China	2005-Nov-08	A/Anhui/1/2005		WHO Chinese National Influenza Center	
EPI164208	HA	Indonesia	2008-May-08	A/Indonesia/8228/2008	Ministry of Health, NIHRD	Eijkman Institute for Molecular Biology	
EPI586501	HA	Canada	2014-Dec-05	A/poultry/BC/FAV17/2014	Animal Health Centre, Ministry of Agriculture	Canadian Food Inspection Agency	
EPI553349	HA	Russian Federation	2014-Sep-25	A/wigeon/Sakha/1/2014	State Research Center of Virology and Biotechnology Vector	State Research Center of Virology and Biotechnology Vector	Susloparov, I; Kolosova, N; Goncharova, N; Marchenko, V; Ryzhikov, A
EPI515479	HA	United Kingdom	2014-Mar-03	A/razorbill/Scotland/7343/14	Animal and Plant Health Agency (APHA)	Animal and Plant Health Agency (APHA)	Hanna, A; Collins, S; Mynn, J; Ceeraz, V; Reid, S; Irvine, R
EPI448279	HA	Bangladesh	2012-Feb-20	A/duck/Bangladesh/32077/2012	Institute of Epidemiology Disease Control and Research (IEDCR) & Bangladesh National Influenza Centre (NIC)	Centers for Disease Control and Prevention	Gerloff, Nancy; Simpson, Natosha; Poh, Mee;Davis, Todd
EPI448271	HA	Bangladesh	2012-Jan-08	A/chicken/Bangladesh/42010/2012	Institute of Epidemiology Disease Control and Research (IEDCR) & Bangladesh National Influenza Centre (NIC)	Centers for Disease Control and Prevention	Gerloff, Nancy; Simpson, Natosha; Poh, Mee;Davis, Todd
EPI448223	HA	Bangladesh	2011-Jul-24	A/duck/Bangladesh/4124T/2011	Institute of Epidemiology Disease Control and Research (IEDCR) & Bangladesh National Influenza Centre (NIC)	Centers for Disease Control and Prevention	Gerloff, Nancy; Simpson, Natosha; Poh, Mee;Davis, Todd
EPI448215	HA	Bangladesh	2011-Jul-24	A/duck/Bangladesh/4120T/2011	Institute of Epidemiology Disease Control and Research (IEDCR) & Bangladesh National Influenza Centre (NIC)	Centers for Disease Control and Prevention	Gerloff, Nancy; Simpson, Natosha; Poh, Mee;Davis, Todd
EPI448207	HA	Bangladesh	2011-Jul-15	A/chicken/Bangladesh/4070T/2011	Institute of Epidemiology Disease Control and Research (IEDCR) & Bangladesh National Influenza Centre (NIC)	Centers for Disease Control and Prevention	Gerloff, Nancy; Simpson, Natosha; Poh, Mee;Davis, Todd
EPI448199	HA	Bangladesh	2011-Jul-14	A/chicken/Bangladesh/4058/2011	Institute of Epidemiology Disease Control and Research (IEDCR) & Bangladesh National Influenza Centre (NIC)	Centers for Disease Control and Prevention	Gerloff, Nancy; Simpson, Natosha; Poh, Mee;Davis, Todd
EPI448191	HA	Bangladesh	2011-Jul-01	A/goose/Bangladesh/4051T/2011	Institute of Epidemiology Disease Control and Research (IEDCR) & Bangladesh National Influenza Centre (NIC)	Centers for Disease Control and Prevention	Gerloff, Nancy; Simpson, Natosha; Poh, Mee;Davis, Todd
EPI448183	HA	Bangladesh	2011-May-24	A/chicken/Bangladesh/3075/2011	Institute of Epidemiology Disease Control and Research (IEDCR) & Bangladesh National Influenza Centre (NIC)	Centers for Disease Control and Prevention	Gerloff, Nancy; Simpson, Natosha; Poh, Mee; Davis, Todd
EPI448167	HA	Bangladesh	2011-Feb-07	A/crow/Bangladesh/316T/2011	Institute of Epidemiology Disease Control and Research (IEDCR) & Bangladesh National Influenza Centre (NIC)	Centers for Disease Control and Prevention	Gerloff, Nancy; Simpson, Natosha; Poh, Mee;Davis, Todd
EPI448159	HA	Bangladesh	2011-Feb-07	A/crow/Bangladesh/313T/2011	Institute of Epidemiology Disease Control and Research (IEDCR) & Bangladesh National Influenza Centre (NIC)	Centers for Disease Control and Prevention	Gerloff, Nancy; Simpson, Natosha; Poh, Mee;Davis, Todd
EPI448151	HA	Bangladesh	2011-Feb-13	A/crow/Bangladesh/1056/2011	Institute of Epidemiology Disease Control and Research (IEDCR) & Bangladesh National Influenza Centre (NIC)	Centers for Disease Control and Prevention	Gerloff, Nancy; Simpson, Natosha; Poh, Mee;Davis, Todd
EPI448135	HA	Bangladesh	2011-Feb-07	A/poultry/Bangladesh/11255-C/2011	Institute of Epidemiology Disease Control and Research (IEDCR) & Bangladesh National Influenza Centre (NIC)	Centers for Disease Control and Prevention	Gerloff, Nancy; Simpson, Natosha; Poh, Mee;Davis, Todd
EPI448111	HA	Bangladesh	2011-Jun-29	A/waterfowl/Bangladesh/33025/2011	Institute of Epidemiology Disease Control and Research (IEDCR) & Bangladesh National Influenza Centre (NIC)	Centers for Disease Control and Prevention	Gerloff, Nancy; Simpson, Natosha; Poh, Mee;Davis, Todd
EPI448063	HA	Bangladesh	2011-Feb-15	A/crow/Bangladesh/1061/2011	Institute of Epidemiology Disease Control and Research (IEDCR) & Bangladesh National Influenza Centre (NIC)	Centers for Disease Control and Prevention	Gerloff, Nancy; Simpson, Natosha; Poh, Mee;Davis, Todd
EPI448055	HA	Bangladesh	2011-Feb-13	A/crow/Bangladesh/1054/2011	Institute of Epidemiology Disease Control and Research (IEDCR) & Bangladesh National Influenza Centre (NIC)	Centers for Disease Control and Prevention	Gerloff, Nancy; Simpson, Natosha; Poh, Mee; Davis, Todd
EPI448047	HA	Bangladesh	2011-Jan-20	A/crow/Bangladesh/1020/2011	Institute of Epidemiology Disease Control and Research (IEDCR) & Bangladesh National Influenza Centre (NIC)	Centers for Disease Control and Prevention	Gerloff, Nancy; Simpson, Natosha; Poh, Mee; Davis, Todd
EPI448039	HA	Bangladesh	2011-Jan-20	A/crow/Bangladesh/1019/2011	Institute of Epidemiology Disease Control and Research (IEDCR) & Bangladesh National Influenza Centre (NIC)	Centers for Disease Control and Prevention	Gerloff, Nancy; Simpson, Natosha; Poh, Mee; Davis, Todd
EPI448031	HA	Bangladesh	2011-Jan-20	A/crow/Bangladesh/1008/2011	Institute of Epidemiology Disease Control and Research (IEDCR) & Bangladesh National Influenza Centre (NIC)	Centers for Disease Control and Prevention	Gerloff, Nancy; Simpson, Natosha; Poh, Mee; Davis, Todd
EPI425955	HA	Vietnam	2011-Sep-07	A/chicken/Vietnam/NCVD-1038/2011	National Centre of Veterinary Diagnostics	Centers for Disease Control and Prevention	
EPI425947	HA	Vietnam	2011-Feb-28	A/chicken/Vietnam/NCVD-715/2011	National Centre of Veterinary Diagnostics	Centers for Disease Control and Prevention	
EPI425939	HA	Vietnam	2012-Aug-22	A/duck/Vietnam/NCVD-1875/2012	National Centre of Veterinary Diagnostics	Centers for Disease Control and Prevention	
EPI425931	HA	Vietnam	2012-Jul-12	A/duck/Vietnam/NCVD-1544/2012	National Centre of Veterinary Diagnostics	Centers for Disease Control and Prevention	
EPI425915	HA	Vietnam	2012-Feb-24	A/duck/Vietnam/NCVD-1304/2012	National Centre of Veterinary Diagnostics	Centers for Disease Control and Prevention	
EPI425907	HA	Vietnam	2012-Feb-17	A/chicken/Vietnam/NCVD-1254/2011	National Centre of Veterinary Diagnostics	Centers for Disease Control and Prevention	
EPI425899	HA	Vietnam	2012-Feb-16	A/duck/Vietnam/NCVD-1251/2011	National Centre of Veterinary Diagnostics	Centers for Disease Control and Prevention	
EPI425891	HA	Vietnam	2012-Feb-08	A/duck/Vietnam/NCVD-1212/2012	National Centre of Veterinary Diagnostics	Centers for Disease Control and Prevention	
EPI425875	HA	Vietnam	2012-Jan-01	A/duck/Vietnam/NCVD-1163/2011	National Centre of Veterinary Diagnostics	Centers for Disease Control and Prevention	
EPI425867	HA	Vietnam	2011-Dec-30	A/duck/Vietnam/NCVD-1162/2011	National Centre of Veterinary Diagnostics	Centers for Disease Control and Prevention	
EPI425859	HA	Vietnam	2011-Dec-30	A/duck/Vietnam/NCVD-1161/2011	National Centre of Veterinary Diagnostics	Centers for Disease Control and Prevention	
EPI425835	HA	Vietnam	2011-Oct-31	A/chicken/Vietnam/NCVD-1148/2011	National Centre of Veterinary Diagnostics	Centers for Disease Control and Prevention	

EPI425819	HA	Vietnam	2011-Sep-08	A/muscovy duck/Vietnam/NCVD-1049/2011	National Centre of Veterinary Diagnostics	Centers for Disease Control and Prevention
EPI425795	HA	Vietnam	2011-Sep-07	A/duck/Vietnam/NCVD-1042/2011	National Centre of Veterinary Diagnostics	Centers for Disease Control and Prevention
EPI425771	HA	Vietnam	2011-Jan-01	A/duck/Vietnam/NCVD-671/2011	National Centre of Veterinary Diagnostics	Centers for Disease Control and Prevention
EPI425688	HA	Vietnam	2012-Jul-31	A/duck/Vietnam/NCVD-1602/2012	National Centre of Veterinary Diagnostics	Centers for Disease Control and Prevention
EPI425680	HA	Vietnam	2012-Jul-15	A/duck/Vietnam/NCVD-1593/2012	National Centre of Veterinary Diagnostics	Centers for Disease Control and Prevention
EPI425672	HA	Vietnam	2012-Aug-30	A/duck/Vietnam/NCVD-1905/2012	National Centre of Veterinary Diagnostics	Centers for Disease Control and Prevention
EPI425656	HA	Vietnam	2012-Sep-21	A/duck/Vietnam/NCVD-1968/2012	National Centre of Veterinary Diagnostics	Centers for Disease Control and Prevention
EPI425640	HA	Vietnam	2012-Aug-19	A/duck/Vietnam/NCVD-1931/2012	National Centre of Veterinary Diagnostics	Centers for Disease Control and Prevention
EPI425624	HA	Vietnam	2012-Aug-18	A/duck/Vietnam/NCVD-1930/2012	National Centre of Veterinary Diagnostics	Centers for Disease Control and Prevention
EPI425616	HA	Vietnam	2012-Mar-02	A/chicken/Vietnam/NCVD-1488/2012	National Centre of Veterinary Diagnostics	Centers for Disease Control and Prevention
EPI425608	HA	Vietnam	2012-Sep-21	A/chicken/Vietnam/NCVD-1963/2012	National Centre of Veterinary Diagnostics	Centers for Disease Control and Prevention
EPI425600	HA	Vietnam	2012-Sep-21	A/duck/Vietnam/NCVD-1463/2012	National Centre of Veterinary Diagnostics	Centers for Disease Control and Prevention
EPI425592	HA	Vietnam	2011-Jun-15	A/chicken/Vietnam/NCVD-883/2011	National Centre of Veterinary Diagnostics	Centers for Disease Control and Prevention
EPI425584	HA	Vietnam	2011-Jun-15	A/chicken/Vietnam/NCVD-882/2011	National Centre of Veterinary Diagnostics	Centers for Disease Control and Prevention
EPI425560	HA	Vietnam	2010-Nov-01	A/duck/Vietnam/NCVD-645/2010	National Centre of Veterinary Diagnostics	Centers for Disease Control and Prevention
EPI425552	HA	Vietnam	2011-Mar-12	A/duck/Vietnam/NCVD-850/2011	National Centre of Veterinary Diagnostics	Centers for Disease Control and Prevention
EPI425544	HA	Vietnam	2011-Mar-03	A/duck/Vietnam/NCVD-825/2011	National Centre of Veterinary Diagnostics	Centers for Disease Control and Prevention
EPI425536	HA	Vietnam	2010-Nov-06	A/duck/Vietnam/NCVD-815/2010	National Centre of Veterinary Diagnostics	Centers for Disease Control and Prevention
EPI425520	HA	Vietnam	2011-Jun-15	A/chicken/Vietnam/NCVD-780/2011	National Centre of Veterinary Diagnostics	Centers for Disease Control and Prevention
EPI425496	HA	Vietnam	2011-Aug-04	A/duck/Vietnam/NCVD-931/2011	National Centre of Veterinary Diagnostics	Centers for Disease Control and Prevention
EPI425480	HA	Vietnam	2011-Feb-25	A/chicken/Vietnam/NCVD-775/2011	National Centre of Veterinary Diagnostics	Centers for Disease Control and Prevention
EPI425472	HA	Vietnam	2012-Feb-16	A/chicken/Vietnam/NCVD-1247/2012	National Centre of Veterinary Diagnostics	Centers for Disease Control and Prevention
EPI425456	HA	Vietnam	2012-Feb-20	A/duck/Vietnam/NCVD-1276/2012	National Centre of Veterinary Diagnostics	Centers for Disease Control and Prevention
EPI425448	HA	Vietnam	2012-Feb-08	A/duck/Vietnam/NCVD-1219/2012	National Centre of Veterinary Diagnostics	Centers for Disease Control and Prevention
EPI425440	HA	Vietnam	2012-Feb-08	A/duck/Vietnam/NCVD-1210/2012	National Centre of Veterinary Diagnostics	Centers for Disease Control and Prevention
EPI425432	HA	Vietnam	2012-Feb-08	A/duck/Vietnam/NCVD-1207/2012	National Centre of Veterinary Diagnostics	Centers for Disease Control and Prevention
EPI425424	HA	Vietnam	2012-Feb-24	A/duck/Vietnam/NCVD-1312/2012	National Centre of Veterinary Diagnostics	Centers for Disease Control and Prevention
EPI425416	HA	Vietnam	2012-Feb-24	A/duck/Vietnam/NCVD-1306/2012	National Centre of Veterinary Diagnostics	Centers for Disease Control and Prevention
EPI425408	HA	Vietnam	2012-Feb-21	A/duck/Vietnam/NCVD-1269/2012	National Centre of Veterinary Diagnostics	Centers for Disease Control and Prevention
EPI425392	HA	Vietnam	2012-Feb-16	A/duck/Vietnam/NCVD-1253/2012	National Centre of Veterinary Diagnostics	Centers for Disease Control and Prevention
EPI425384	HA	Vietnam	2012-Feb-16	A/chicken/Vietnam/NCVD-1248/2012	National Centre of Veterinary Diagnostics	Centers for Disease Control and Prevention
EPI425376	HA	Vietnam	2012-Feb-17	A/duck/Vietnam/NCVD-1234/2012	National Centre of Veterinary Diagnostics	Centers for Disease Control and Prevention
EPI425368	HA	Vietnam	2012-Feb-17	A/duck/Vietnam/NCVD-1232/2012	National Centre of Veterinary Diagnostics	Centers for Disease Control and Prevention
EPI425360	HA	Vietnam	2012-Jan-06	A/chicken/Vietnam/NCVD-1225/2012	National Centre of Veterinary Diagnostics	Centers for Disease Control and Prevention
EPI425336	HA	Vietnam	2012-Feb-08	A/duck/Vietnam/NCVD-1221/2012	National Centre of Veterinary Diagnostics	Centers for Disease Control and Prevention
EPI425320	HA	Vietnam	2012-Feb-08	A/duck/Vietnam/NCVD-1217/2012	National Centre of Veterinary Diagnostics	Centers for Disease Control and Prevention
EPI425304	HA	Vietnam	2012-Aug-28	A/duck/Vietnam/NCVD-1902/2012	National Centre of Veterinary Diagnostics	Centers for Disease Control and Prevention
EPI425296	HA	Vietnam	2011-Jan-01	A/duck/Vietnam/NCVD-669/2011	National Centre of Veterinary Diagnostics	Centers for Disease Control and Prevention
EPI425248	HA	Vietnam	2012-Jan-30	A/chicken/Vietnam/NCVD-1177/2012	National Centre of Veterinary Diagnostics	Centers for Disease Control and Prevention
EPI425240	HA	Vietnam	2012-Jan-13	A/duck/Vietnam/NCVD-1171/2012	National Centre of Veterinary Diagnostics	Centers for Disease Control and Prevention
EPI425232	HA	Vietnam	2011-Dec-30	A/duck/Vietnam/NCVD-1160/2011	National Centre of Veterinary Diagnostics	Centers for Disease Control and Prevention
EPI425224	HA	Vietnam	2011-Nov-26	A/duck/Vietnam/NCVD1-48/2011	National Centre of Veterinary Diagnostics	Centers for Disease Control and Prevention
EPI425216	HA	Vietnam	2011-Nov-26	A/duck/Vietnam/NCVD1-37/2011	National Centre of Veterinary Diagnostics	Centers for Disease Control and Prevention
EPI425184	HA	Vietnam	2012-Sep-09	A/duck/Vietnam/NCVD-1944/2012	National Centre of Veterinary Diagnostics	Centers for Disease Control and Prevention
EPI425176	HA	Vietnam	2012-Sep-01	A/chicken/Vietnam/NCVD-1942/2012	National Centre of Veterinary Diagnostics	Centers for Disease Control and Prevention
EPI425160	HA	Vietnam	2012-Aug-25	A/duck/Vietnam/NCVD-1937/2012	National Centre of Veterinary Diagnostics	Centers for Disease Control and Prevention
EPI425152	HA	Vietnam	2012-Aug-23	A/duck/Vietnam/NCVD-1935/2012	National Centre of Veterinary Diagnostics	Centers for Disease Control and Prevention
EPI425144	HA	Vietnam	2012-Aug-21	A/duck/Vietnam/NCVD-1933/2012	National Centre of Veterinary Diagnostics	Centers for Disease Control and Prevention
EPI425136	HA	Vietnam	2012-Aug-28	A/duck/Vietnam/NCVD-1899/2012	National Centre of Veterinary Diagnostics	Centers for Disease Control and Prevention
EPI425128	HA	Vietnam	2012-Aug-21	A/duck/Vietnam/NCVD-1872/2012	National Centre of Veterinary Diagnostics	Centers for Disease Control and Prevention
EPI425104	HA	Vietnam	2012-Jun-12	A/duck/Vietnam/NCVD-1497/2012	National Centre of Veterinary Diagnostics	Centers for Disease Control and Prevention
EPI425096	HA	Vietnam	2011-Apr-03	A/duck/Vietnam/NCVD-827/2011	National Centre of Veterinary Diagnostics	Centers for Disease Control and Prevention
EPI425088	HA	Vietnam	2011-Jun-15	A/chicken/Vietnam/NCVD-779/2011	National Centre of Veterinary Diagnostics	Centers for Disease Control and Prevention
EPI425080	HA	Vietnam	2011-Jun-15	A/chicken/Vietnam/NCVD-778/2011	National Centre of Veterinary Diagnostics	Centers for Disease Control and Prevention
EPI425072	HA	Vietnam	2011-Feb-25	A/chicken/Vietnam/NCVD-776/2011	National Centre of Veterinary Diagnostics	Centers for Disease Control and Prevention
EPI425024	HA	Vietnam	2012-Aug-25	A/duck/Vietnam/NCVD-1884/2012	National Centre of Veterinary Diagnostics	Centers for Disease Control and Prevention
EPI425016	HA	Vietnam	2012-Aug-23	A/duck/Vietnam/NCVD-1878/2012	National Centre of Veterinary Diagnostics	Centers for Disease Control and Prevention
EPI425008	HA	Vietnam	2012-May-04	A/chicken/Vietnam/NCVD-1437/2012	National Centre of Veterinary Diagnostics	Centers for Disease Control and Prevention
EPI424992	HA	Vietnam	2012-Jul-31	A/duck/Vietnam/NCVD-1607/2012	National Centre of Veterinary Diagnostics	Centers for Disease Control and Prevention
EPI424984	HA	Vietnam	2012-Jul-27	A/duck/Vietnam/NCVD-1584/2012	National Centre of Veterinary Diagnostics	Centers for Disease Control and Prevention
EPI424976	HA	Vietnam	2011-Jun-15	A/chicken/Vietnam/NCVD-710/2011	National Centre of Veterinary Diagnostics	Centers for Disease Control and Prevention
EPI424968	HA	Vietnam	2011-Jun-15	A/chicken/Vietnam/NCVD-709/2011	National Centre of Veterinary Diagnostics	Centers for Disease Control and Prevention
EPI424960	HA	Vietnam	2010-Jan-01	A/duck/Vietnam/NCVD-891/2010	National Centre of Veterinary Diagnostics	Centers for Disease Control and Prevention
EPI424952	HA	Vietnam	2010-Jan-01	A/duck/Vietnam/NCVD-890/2010	National Centre of Veterinary Diagnostics	Centers for Disease Control and Prevention
EPI424944	HA	Vietnam	2010-Jan-01	A/duck/Vietnam/NCVD-885/2010	National Centre of Veterinary Diagnostics	Centers for Disease Control and Prevention
EPI424928	HA	Vietnam	2011-Apr-06	A/duck/Vietnam/NCVD-947/2011	National Centre of Veterinary Diagnostics	Centers for Disease Control and Prevention
EPI424920	HA	Vietnam	2011-Mar-18	A/duck/Vietnam/NCVD-945/2011	National Centre of Veterinary Diagnostics	Centers for Disease Control and Prevention
EPI424912	HA	Vietnam	2011-Feb-12	A/duck/Vietnam/NCVD-943/2011	National Centre of Veterinary Diagnostics	Centers for Disease Control and Prevention
EPI424904	HA	Vietnam	2011-Aug-04	A/duck/Vietnam/NCVD-935/2011	National Centre of Veterinary Diagnostics	Centers for Disease Control and Prevention
EPI424880	HA	Vietnam	2011-Sep-06	A/duck/Vietnam/NCVD-1029/2011	National Centre of Veterinary Diagnostics	Centers for Disease Control and Prevention
EPI424864	HA	Vietnam	2011-Sep-06	A/duck/Vietnam/NCVD-1025/2011	National Centre of Veterinary Diagnostics	Centers for Disease Control and Prevention
EPI424856	HA	Vietnam	2011-Sep-06	A/duck/Vietnam/NCVD-1022/2011	National Centre of Veterinary Diagnostics	Centers for Disease Control and Prevention
EPI424848	HA	Vietnam	2011-Sep-06	A/duck/Vietnam/NCVD-1018/2011	National Centre of Veterinary Diagnostics	Centers for Disease Control and Prevention
EPI424840	HA	Vietnam	2011-Jun-24	A/duck/Vietnam/NCVD-952/2011	National Centre of Veterinary Diagnostics	Centers for Disease Control and Prevention
EPI424832	HA	Vietnam	2011-Jun-15	A/duck/Vietnam/NCVD-711/2011	National Centre of Veterinary Diagnostics	Centers for Disease Control and Prevention
EPI424808	HA	Vietnam	2011-Jun-15	A/chicken/Vietnam/NCVD-881/2011	National Centre of Veterinary Diagnostics	Centers for Disease Control and Prevention
EPI424800	HA	Vietnam	2011-Jun-15	A/chicken/Vietnam/NCVD-880/2011	National Centre of Veterinary Diagnostics	Centers for Disease Control and Prevention
EPI424792	HA	Vietnam	2011-Jun-15	A/chicken/Vietnam/NCVD-876/2011	National Centre of Veterinary Diagnostics	Centers for Disease Control and Prevention
EPI424784	HA	Vietnam	2011-Apr-07	A/duck/Vietnam/NCVD-861/2011	National Centre of Veterinary Diagnostics	Centers for Disease Control and Prevention
EPI424776	HA	Vietnam	2011-Apr-07	A/duck/Vietnam/NCVD-859/2011	National Centre of Veterinary Diagnostics	Centers for Disease Control and Prevention
EPI424752	HA	Vietnam	2010-Oct-29	A/duck/Vietnam/NCVD-784/2010	National Centre of Veterinary Diagnostics	Centers for Disease Control and Prevention
EPI424744	HA	Vietnam	2012-Aug-20	A/duck/Vietnam/NCVD-1932/2012	National Centre of Veterinary Diagnostics	Centers for Disease Control and Prevention
EPI424712	HA	Vietnam	2012-Aug-23	A/duck/Vietnam/NCVD-1896/2012	National Centre of Veterinary Diagnostics	Centers for Disease Control and Prevention
EPI424704	HA	Vietnam	2012-Aug-20	A/duck/Vietnam/NCVD-1869/2012	National Centre of Veterinary Diagnostics	Centers for Disease Control and Prevention
EPI424696	HA	Vietnam	2011-Apr-06	A/duck/Vietnam/NCVD-948/2011	National Centre of Veterinary Diagnostics	Centers for Disease Control and Prevention
EPI424688	HA	Vietnam	2012-Sep-21	A/duck/Vietnam/NCVD-1974/2012	National Centre of Veterinary Diagnostics	Centers for Disease Control and Prevention

EPI424680	HA	Vietnam	2012-Jun-11	A/duck/Vietnam/NCVD-1494/2012	National Centre of Veterinary Diagnostics	Centers for Disease Control and Prevention	
EPI424434	HA	Vietnam	2011-Feb-01	A/chicken/Vietnam/NCVD-705/2011	National Centre of Veterinary Diagnostics	Centers for Disease Control and Prevention	
EPI424418	HA	Vietnam	2011-Feb-01	A/chicken/Vietnam/NCVD-703/2011	National Centre of Veterinary Diagnostics	Centers for Disease Control and Prevention	
EPI424410	HA	Vietnam	2011-Feb-01	A/chicken/Vietnam/NCVD-702/2011	National Centre of Veterinary Diagnostics	Centers for Disease Control and Prevention	
EPI424402	HA	Vietnam	2011-Feb-01	A/chicken/Vietnam/NCVD-699/2011	National Centre of Veterinary Diagnostics	Centers for Disease Control and Prevention	
EPI424394	HA	Vietnam	2012-Feb-26	A/duck/Vietnam/NCVD-1283/2012	National Centre of Veterinary Diagnostics	Centers for Disease Control and Prevention	
EPI424370	HA	Vietnam	2010-Dec-01	A/duck/Vietnam/NCVD-664/2010	National Centre of Veterinary Diagnostics	Centers for Disease Control and Prevention	
EPI424362	HA	Vietnam	2012-Feb-08	A/duck/Vietnam/NCVD-1206/2012	National Centre of Veterinary Diagnostics	Centers for Disease Control and Prevention	
EPI424354	HA	Vietnam	2011-Jun-15	A/chicken/Vietnam/NCVD-878/2011	National Centre of Veterinary Diagnostics	Centers for Disease Control and Prevention	
EPI373010	HA	Egypt	2007-Jan-01	A/chicken/Egypt/9403-NAMRU3/2007	U.S. Naval Medical Research Unit No.3	Centers for Disease Control and Prevention	Younan, M.; Poh, M.K.; Elassal, E.; Simpson, N.; Jones, J.; ElBadry, M.A.; Naguib, A.;Saied, I.; Kandeel, A.; Cornelius, C.
EPI353372	HA	Bangladesh	2011-Feb-19	A/chicken/Bangladesh/3012/2011	Institute of Epidemiology Disease Control and Research (IEDCR) & Bangladesh National Influenza Centre (NIC)	Centers for Disease Control and Prevention	Gerloff, Nancy; Simpson, Natosha; Poh, Mee;Davis, Todd
EPI353365	HA	Bangladesh	2011-Jul-14	A/duck/Bangladesh/4059T/2011	Institute of Epidemiology Disease Control and Research (IEDCR) & Bangladesh National Influenza Centre (NIC)	Centers for Disease Control and Prevention	Gerloff, Nancy; Simpson, Natosha; Poh, Mee;Davis, Todd
EPI347303	HA	Vietnam	2010-Jun-30	A/duck/Vietnam/NCVD-433/2010	National Institute of Hygiene and Epidemiology	Centers for Disease Control and Prevention	Davis,Todd; Rivailler,Pierre; Nguyen,Tung
EPI347302	HA	Vietnam	2010-Mar-29	A/duck/Vietnam/NCVD-472/2010	National Institute of Hygiene and Epidemiology	Centers for Disease Control and Prevention	Davis,Todd; Rivailler,Pierre; Nguyen,Tung
EPI347301	HA	Vietnam	2010-Mar-29	A/duck/Vietnam/NCVD-471/2010	National Institute of Hygiene and Epidemiology	Centers for Disease Control and Prevention	Davis,Todd; Rivailler,Pierre; Nguyen,Tung
EPI347300	HA	Vietnam	2010-Oct-15	A/duck/Vietnam/NCVD-462/2010	National Institute of Hygiene and Epidemiology	Centers for Disease Control and Prevention	Davis,Todd; Rivailler,Pierre; Nguyen,Tung
EPI347297	HA	Vietnam	2010-Nov-22	A/chicken/Vietnam/NCVD-459/2010	National Institute of Hygiene and Epidemiology	Centers for Disease Control and Prevention	Davis,Todd; Rivailler,Pierre; Nguyen,Tung
EPI347296	HA	Vietnam	2010-Nov-16	A/duck/Vietnam/NCVD-457/2010	National Institute of Hygiene and Epidemiology	Centers for Disease Control and Prevention	Davis,Todd; Rivailler,Pierre; Nguyen,Tung
EPI347295	HA	Vietnam	2010-Nov-16	A/duck/Vietnam/NCVD-456/2010	National Institute of Hygiene and Epidemiology	Centers for Disease Control and Prevention	Davis,Todd; Rivailler,Pierre; Nguyen,Tung
EPI347294	HA	Vietnam	2010-Oct-04	A/duck/Vietnam/NCVD-449/2010	National Institute of Hygiene and Epidemiology	Centers for Disease Control and Prevention	Davis,Todd; Rivailler,Pierre; Nguyen,Tung
EPI347293	HA	Vietnam	2010-Jul-14	A/duck/Vietnam/NCVD-432/2010	National Institute of Hygiene and Epidemiology	Centers for Disease Control and Prevention	Davis,Todd; Rivailler,Pierre; Nguyen,Tung
EPI347292	HA	Vietnam	2010-Mar-04	A/duck/Vietnam/NCVD-430/2010	National Institute of Hygiene and Epidemiology	Centers for Disease Control and Prevention	Davis,Todd; Rivailler,Pierre; Nguyen,Tung
EPI347291	HA	Vietnam	2010-Feb-25	A/chicken/Vietnam/NCVD-429/2010	National Institute of Hygiene and Epidemiology	Centers for Disease Control and Prevention	Davis,Todd; Rivailler,Pierre; Nguyen,Tung
EPI347287	HA	Vietnam	2009-Feb-20	A/chicken/Vietnam/NCVD-425/2009	National Institute of Hygiene and Epidemiology	Centers for Disease Control and Prevention	Davis,Todd; Rivailler,Pierre; Nguyen,Tung
EPI347286	HA	Vietnam	2009-Feb-17	A/chicken/Vietnam/NCVD-424/2009	National Institute of Hygiene and Epidemiology	Centers for Disease Control and Prevention	Davis,Todd; Rivailler,Pierre; Nguyen,Tung
EPI340808	HA	Indonesia	2010-Jan-01	A/quail/Klaten/BBVW-110-II/2010	Disease Investigation Centre Wates (BBVW)	CSIRO Australian Animal Health Laboratory	Dhamawan,Rama; Usman,Tri Bhakti; Junaidi,Akhmad; Suatmodjo,Musny; Bruce,Kerri; Davies,Kelly R; Stevens,Vittoria; Kim,Mia; Daniels,Peter; Wong,Frank YK
EPI340790	HA	Indonesia	2010-Jan-01	A/bird/Gunung Kidul/BBVW-52-I/2010	Disease Investigation Centre Wates (BBVW)	CSIRO Australian Animal Health Laboratory	Dhamawan,Rama; Usman,Tri Bhakti; Junaidi,Akhmad; Suatmodjo,Musny; Bruce,Kerri; Davies,Kelly R; Stevens,Vittoria; Kim,Mia; Daniels,Peter; Wong,Frank YK
EPI330999	HA	Vietnam	2010-Nov-16	A/duck/Vietnam/NCVD-458/2010	National Institute of Hygiene and Epidemiology	Centers for Disease Control and Prevention	Davis,Todd; Rivailler,Pierre; Nguyen,Tung
EPI330996	HA	Vietnam	2010-May-12	A/duck/Vietnam/NCVD-431/2010	National Institute of Hygiene and Epidemiology	Centers for Disease Control and Prevention	Davis,Todd; Rivailler,Pierre; Nguyen,Tung
EPI305616	HA	Turkey	2007-Feb-15	A/Sparrow/Turkey-Batman/09rs2842-104/2007	Istituto Zooprofilattico Sperimentale Delle Venezie	Istituto Zooprofilattico Sperimentale Delle Venezie	Schivo,A.; Valastro,V.; Monne,I.; Coven,F.; Fusaro,A.; Dakman,A.; Akcadag,B.; Salviato,A.; DeBattisti,C.; Capua,I.; Cattoli,G.
EPI305614	HA	Turkey	2006-Feb-14	A/Pigeon/Turkey-Mardin/09rs2841-95/2006	Istituto Zooprofilattico Sperimentale Delle Venezie	Istituto Zooprofilattico Sperimentale Delle Venezie	Schivo,A.; Valastro,V.; Monne,I.; Coven,F.; Fusaro,A.; Dakman,A.; Akcadag,B.; Salviato,A.; DeBattisti,C.; Capua,I.; Cattoli,G.
EPI305612	HA	Turkey	2006-Jan-23	A/Pigeon/Turkey-Karabuk/09rs2841-61/2006	Istituto Zooprofilattico Sperimentale Delle Venezie	Istituto Zooprofilattico Sperimentale Delle Venezie	Schivo,A.; Valastro,V.; Monne,I.; Coven,F.; Fusaro,A.; Dakman,A.; Akcadag,B.; Salviato,A.; DeBattisti,C.; Capua,I.; Cattoli,G.
EPI305611	HA	Turkey	2006-Jan-12	A/Pigeon/Turkey-Aydin/09rs2841-40/2006	Istituto Zooprofilattico Sperimentale Delle Venezie	Istituto Zooprofilattico Sperimentale Delle Venezie	Schivo,A.; Valastro,V.; Monne,I.; Coven,F.; Fusaro,A.; Dakman,A.; Akcadag,B.; Salviato,A.; DeBattisti,C.; Capua,I.; Cattoli,G.
EPI305610	HA	Turkey	2006-Jan-23	A/Pigeon/Turkey-Izmir/09rs2841-38/2006	Istituto Zooprofilattico Sperimentale Delle Venezie	Istituto Zooprofilattico Sperimentale Delle Venezie	Schivo,A.; Valastro,V.; Monne,I.; Coven,F.; Fusaro,A.; Dakman,A.; Akcadag,B.; Salviato,A.; DeBattisti,C.; Capua,I.; Cattoli,G.
EPI305555	HA	Turkey	2006-Jan-23	A/Avian/Turkey-Erzurum/09rs2841-46/2006	Istituto Zooprofilattico Sperimentale Delle Venezie	Istituto Zooprofilattico Sperimentale Delle Venezie	Schivo,A.; Valastro,V.; Monne,I.; Coven,F.; Fusaro,A.; Dakman,A.; Akcadag,B.; Salviato,A.; DeBattisti,C.; Capua,I.; Cattoli,G.
EPI287384	HA	Egypt	2010-Jan-01	A/poultry/Egypt/4794-18/2010	Istituto Zooprofilattico Sperimentale Delle Venezie	Istituto Zooprofilattico Sperimentale Delle Venezie	
EPI287383	HA	Egypt	2010-Jan-01	A/poultry/Egypt/4794-17/2010	Istituto Zooprofilattico Sperimentale Delle Venezie	Istituto Zooprofilattico Sperimentale Delle Venezie	
EPI287380	HA	Egypt	2010-Mar-15	A/poultry/Egypt/3982-55/2010	Istituto Zooprofilattico Sperimentale Delle Venezie	Istituto Zooprofilattico Sperimentale Delle Venezie	
EPI284593	HA	Vietnam	2008-Dec-01	A/duck/Vietnam/NCVD-187/2008	National Centre of Veterinary Diagnostics	Centers for Disease Control and Prevention	Davis,Todd; Rivailler,Pierre; Nguyen,Tung
EPI284590	HA	Vietnam	2008-Jan-01	A/quail/Vietnam/NCVD-037/2008	National Centre of Veterinary Diagnostics	Centers for Disease Control and Prevention	Davis,Todd; Rivailler,Pierre; Nguyen,Tung
EPI284589	HA	Vietnam	2010-Apr-01	A/poultry/Vietnam/NCVD-416/2010	National Centre of Veterinary Diagnostics	Centers for Disease Control and Prevention	Davis,Todd; Rivailler,Pierre; Nguyen,Tung
EPI284588	HA	Vietnam	2008-Dec-01	A/avian/Vietnam/NCVD-133/2008	National Centre of Veterinary Diagnostics	Centers for Disease Control and Prevention	Davis,Todd; Rivailler,Pierre; Nguyen,Tung
EPI284587	HA	Vietnam	2008-Dec-01	A/avian/Vietnam/NCVD-132/2008	National Centre of Veterinary Diagnostics	Centers for Disease Control and Prevention	Davis,Todd; Rivailler,Pierre; Nguyen,Tung
EPI284586	HA	Vietnam	2008-Dec-01	A/avian/Vietnam/NCVD-125/2008	National Centre of Veterinary Diagnostics	Centers for Disease Control and Prevention	Davis,Todd; Rivailler,Pierre; Nguyen,Tung
EPI284585	HA	Vietnam	2008-Dec-01	A/avian/Vietnam/NCVD-124/2008	National Centre of Veterinary Diagnostics	Centers for Disease Control and Prevention	Davis,Todd; Rivailler,Pierre; Nguyen,Tung
EPI284584	HA	Vietnam	2009-Jan-01	A/ostrich/Vietnam/NCVD-373/2009	National Centre of Veterinary Diagnostics	Centers for Disease Control and Prevention	Davis,Todd; Rivailler,Pierre; Nguyen,Tung
EPI284581	HA	Vietnam	2008-Jan-01	A/duck/Vietnam/NCVD-011/2008	National Centre of Veterinary Diagnostics	Centers for Disease Control and Prevention	Davis,Todd; Rivailler,Pierre; Nguyen,Tung
EPI284579	HA	Vietnam	2008-Jan-01	A/duck/Vietnam/NCVD-009/2008	National Centre of Veterinary Diagnostics	Centers for Disease Control and Prevention	Davis,Todd; Rivailler,Pierre; Nguyen,Tung
EPI284578	HA	Vietnam	2008-Jan-01	A/duck/Vietnam/NCVD-024/2008	National Centre of Veterinary Diagnostics	Centers for Disease Control and Prevention	Davis,Todd; Rivailler,Pierre; Nguyen,Tung
EPI284574	HA	Vietnam	2007-Jan-01	A/duck/Vietnam/NCVD-102/2007	National Centre of Veterinary Diagnostics	Centers for Disease Control and Prevention	Davis,Todd; Rivailler,Pierre; Nguyen,Tung
EPI284571	HA	Vietnam	2007-Jan-01	A/duck/Vietnam/NCVD-116/2007	National Centre of Veterinary Diagnostics	Centers for Disease Control and Prevention	Davis,Todd; Rivailler,Pierre; Nguyen,Tung
EPI284569	HA	Vietnam	2008-Jan-01	A/duck/Vietnam/NCVD-023/2008	National Centre of Veterinary Diagnostics	Centers for Disease Control and Prevention	Davis,Todd; Rivailler,Pierre; Nguyen,Tung
EPI284568	HA	Vietnam	2008-Jan-01	A/duck/Vietnam/NCVD-022/2008	National Centre of Veterinary Diagnostics	Centers for Disease Control and Prevention	Davis,Todd; Rivailler,Pierre; Nguyen,Tung
EPI284567	HA	Vietnam	2008-Jan-01	A/duck/Vietnam/NCVD-021/2008	National Centre of Veterinary Diagnostics	Centers for Disease Control and Prevention	Davis,Todd; Rivailler,Pierre; Nguyen,Tung
EPI284566	HA	Vietnam	2008-Jan-01	A/duck/Vietnam/NCVD-020/2008	National Centre of Veterinary Diagnostics	Centers for Disease Control and Prevention	Davis,Todd; Rivailler,Pierre; Nguyen,Tung
EPI284562	HA	Vietnam	2007-Jan-01	A/duck/Vietnam/NCVD-93/2007	National Centre of Veterinary Diagnostics	Centers for Disease Control and Prevention	Davis,Todd; Rivailler,Pierre; Nguyen,Tung
EPI284560	HA	Vietnam	2007-Jan-01	A/duck/Vietnam/NCVD-104/2007	National Centre of Veterinary Diagnostics	Centers for Disease Control and Prevention	Davis,Todd; Rivailler,Pierre; Nguyen,Tung
EPI284553	HA	Vietnam	2007-Jan-01	A/duck/Vietnam/NCVD-99/2007	National Centre of Veterinary Diagnostics	Centers for Disease Control and Prevention	Davis,Todd; Rivailler,Pierre; Nguyen,Tung
EPI284552	HA	Vietnam	2008-May-01	A/duck/Vietnam/NCVD-103/2008	National Centre of Veterinary Diagnostics	Centers for Disease Control and Prevention	Davis,Todd; Rivailler,Pierre; Nguyen,Tung
EPI284551	HA	Vietnam	2008-May-01	A/duck/Vietnam/NCVD-101/2008	National Centre of Veterinary Diagnostics	Centers for Disease Control and Prevention	Davis,Todd; Rivailler,Pierre; Nguyen,Tung
EPI284550	HA	Vietnam	2009-Mar-01	A/duck/Vietnam/NCVD-293/2009	National Centre of Veterinary Diagnostics	Centers for Disease Control and Prevention	Davis,Todd; Rivailler,Pierre; Nguyen,Tung
EPI284545	HA	Vietnam	2010-Feb-01	A/ngan/Vietnam/NCVD-420/2010	National Centre of Veterinary Diagnostics	Centers for Disease Control and Prevention	Davis,Todd; Rivailler,Pierre; Nguyen,Tung
EPI284544	HA	Vietnam	2008-Dec-01	A/duck/Vietnam/NCVD-186/2008	National Centre of Veterinary Diagnostics	Centers for Disease Control and Prevention	Davis,Todd; Rivailler,Pierre; Nguyen,Tung
EPI284543	HA	Vietnam	2008-Dec-01	A/duck/Vietnam/NCVD-160/2008	National Centre of Veterinary Diagnostics	Centers for Disease Control and Prevention	Davis,Todd; Rivailler,Pierre; Nguyen,Tung
EPI284542	HA	Vietnam	2008-Dec-01	A/duck/Vietnam/NCVD-159/2008	National Centre of Veterinary Diagnostics	Centers for Disease Control and Prevention	Davis,Todd; Rivailler,Pierre; Nguyen,Tung
EPI284541	HA	Vietnam	2008-Dec-01	A/duck/Vietnam/NCVD-146/2008	National Centre of Veterinary Diagnostics	Centers for Disease Control and Prevention	Davis,Todd; Rivailler,Pierre; Nguyen,Tung
EPI284537	HA	Vietnam	2008-Dec-01	A/duck/Vietnam/NCVD-140/2008	National Centre of Veterinary Diagnostics	Centers for Disease Control and Prevention	Davis,Todd; Rivailler,Pierre; Nguyen,Tung
EPI284535	HA	Vietnam	2009-Jan-01	A/duck/Vietnam/NCVD-372/2009	National Centre of Veterinary Diagnostics	Centers for Disease Control and Prevention	Davis,Todd; Rivailler,Pierre; Nguyen,Tung
EPI284534	HA	Vietnam	2009-Jan-01	A/duck/Vietnam/NCVD-379/2009	National Centre of Veterinary Diagnostics	Centers for Disease Control and Prevention	Davis,Todd; Rivailler,Pierre; Nguyen,Tung
EPI284532	HA	Vietnam	2009-Jan-01	A/duck/Vietnam/NCVD-377/2009	National Centre of Veterinary Diagnostics	Centers for Disease Control and Prevention	Davis,Todd; Rivailler,Pierre; Nguyen,Tung

EPI284527	HA	Vietnam	2009-Jan-01	A/duck/Vietnam/NCVD-362/2009	National Centre of Veterinary Diagnostics	Centers for Disease Control and Prevention	Davis,Todd; Rivailler,Pierre; Nguyen,Tung
EPI284526	HA	Vietnam	2009-Jan-01	A/duck/Vietnam/NCVD-374/2009	National Centre of Veterinary Diagnostics	Centers for Disease Control and Prevention	Davis,Todd; Rivailler,Pierre; Nguyen,Tung
EPI284524	HA	Vietnam	2009-Jan-01	A/duck/Vietnam/NCVD-375/2009	National Centre of Veterinary Diagnostics	Centers for Disease Control and Prevention	Davis,Todd; Rivailler,Pierre; Nguyen,Tung
EPI284523	HA	Vietnam	2009-Jan-01	A/duck/Vietnam/NCVD-368/2009	National Centre of Veterinary Diagnostics	Centers for Disease Control and Prevention	Davis,Todd; Rivailler,Pierre; Nguyen,Tung
EPI284522	HA	Vietnam	2009-Jan-01	A/duck/Vietnam/NCVD-367/2009	National Centre of Veterinary Diagnostics	Centers for Disease Control and Prevention	Davis,Todd; Rivailler,Pierre; Nguyen,Tung
EPI284518	HA	Vietnam	2009-Jan-01	A/duck/Vietnam/NCVD-371/2009	National Centre of Veterinary Diagnostics	Centers for Disease Control and Prevention	Davis,Todd; Rivailler,Pierre; Nguyen,Tung
EPI284517	HA	Vietnam	2009-Jan-01	A/duck/Vietnam/NCVD-370/2009	National Centre of Veterinary Diagnostics	Centers for Disease Control and Prevention	Davis,Todd; Rivailler,Pierre; Nguyen,Tung
EPI284516	HA	Vietnam	2008-Jan-01	A/muscovy duck/Vietnam/NCVD-041/2008	National Centre of Veterinary Diagnostics	Centers for Disease Control and Prevention	Davis,Todd; Rivailler,Pierre; Nguyen,Tung
EPI284514	HA	Vietnam	2007-Jan-01	A/muscovy duck/Vietnam/NCVD-119/2007	National Centre of Veterinary Diagnostics	Centers for Disease Control and Prevention	Davis,Todd; Rivailler,Pierre; Nguyen,Tung
EPI284512	HA	Vietnam	2007-Jan-01	A/muscovy duck/Vietnam/NCVD-112/2007	National Centre of Veterinary Diagnostics	Centers for Disease Control and Prevention	Davis,Todd; Rivailler,Pierre; Nguyen,Tung
EPI284511	HA	Vietnam	2007-Jan-01	A/muscovy duck/Vietnam/NCVD-101/2007	National Centre of Veterinary Diagnostics	Centers for Disease Control and Prevention	Davis,Todd; Rivailler,Pierre; Nguyen,Tung
EPI284510	HA	Vietnam	2008-Jan-01	A/muscovy duck/Vietnam/NCVD-038/2008	National Centre of Veterinary Diagnostics	Centers for Disease Control and Prevention	Davis,Todd; Rivailler,Pierre; Nguyen,Tung
EPI284508	HA	Vietnam	2008-May-01	A/muscovy duck/Vietnam/NCVD-082/2008	National Centre of Veterinary Diagnostics	Centers for Disease Control and Prevention	Davis,Todd; Rivailler,Pierre; Nguyen,Tung
EPI284505	HA	Vietnam	2009-Jan-01	A/muscovy duck/Vietnam/NCVD-384/2009	National Centre of Veterinary Diagnostics	Centers for Disease Control and Prevention	Davis,Todd; Rivailler,Pierre; Nguyen,Tung
EPI284502	HA	Vietnam	2008-Dec-01	A/muscovy duck/Vietnam/NCVD-154/2008	National Centre of Veterinary Diagnostics	Centers for Disease Control and Prevention	Davis,Todd; Rivailler,Pierre; Nguyen,Tung
EPI284501	HA	Vietnam	2010-Apr-01	A/muscovy duck/Vietnam/NCVD-403/2010	National Centre of Veterinary Diagnostics	Centers for Disease Control and Prevention	Davis,Todd; Rivailler,Pierre; Nguyen,Tung
EPI284499	HA	Vietnam	2008-Jan-01	A/chicken/Vietnam/NCVD-026/2008	National Centre of Veterinary Diagnostics	Centers for Disease Control and Prevention	Davis,Todd; Rivailler,Pierre; Nguyen,Tung
EPI284498	HA	Vietnam	2008-Jan-01	A/chicken/Vietnam/NCVD-018/2008	National Centre of Veterinary Diagnostics	Centers for Disease Control and Prevention	Davis,Todd; Rivailler,Pierre; Nguyen,Tung
EPI284496	HA	Vietnam	2008-Jan-01	A/chicken/Vietnam/NCVD-008/2008	National Centre of Veterinary Diagnostics	Centers for Disease Control and Prevention	Davis,Todd; Rivailler,Pierre; Nguyen,Tung
EPI284495	HA	Vietnam	2008-Jan-01	A/chicken/Vietnam/NCVD-029/2008	National Centre of Veterinary Diagnostics	Centers for Disease Control and Prevention	Davis,Todd; Rivailler,Pierre; Nguyen,Tung
EPI284492	HA	Vietnam	2008-Jan-01	A/chicken/Vietnam/NCVD-033/2008	National Centre of Veterinary Diagnostics	Centers for Disease Control and Prevention	Davis,Todd; Rivailler,Pierre; Nguyen,Tung
EPI284489	HA	Vietnam	2008-Jan-01	A/chicken/Vietnam/NCVD-035/2008	National Centre of Veterinary Diagnostics	Centers for Disease Control and Prevention	Davis,Todd; Rivailler,Pierre; Nguyen,Tung
EPI284487	HA	Vietnam	2008-Jan-01	A/chicken/Vietnam/NCVD-006/2008	National Centre of Veterinary Diagnostics	Centers for Disease Control and Prevention	Davis,Todd; Rivailler,Pierre; Nguyen,Tung
EPI284486	HA	Vietnam	2008-Jan-01	A/chicken/Vietnam/NCVD-005/2008	National Centre of Veterinary Diagnostics	Centers for Disease Control and Prevention	Davis,Todd; Rivailler,Pierre; Nguyen,Tung
EPI284484	HA	Vietnam	2008-Jan-01	A/chicken/Vietnam/NCVD-027/2008	National Centre of Veterinary Diagnostics	Centers for Disease Control and Prevention	Davis,Todd; Rivailler,Pierre; Nguyen,Tung
EPI284483	HA	Vietnam	2007-Jan-01	A/chicken/Vietnam/NCVD-103/2007	National Centre of Veterinary Diagnostics	Centers for Disease Control and Prevention	Davis,Todd; Rivailler,Pierre; Nguyen,Tung
EPI284481	HA	Vietnam	2008-May-01	A/chicken/Vietnam/NCVD-085/2008	National Centre of Veterinary Diagnostics	Centers for Disease Control and Prevention	Davis,Todd; Rivailler,Pierre; Nguyen,Tung
EPI284479	HA	Vietnam	2008-May-01	A/chicken/Vietnam/NCVD-083/2008	National Centre of Veterinary Diagnostics	Centers for Disease Control and Prevention	Davis,Todd; Rivailler,Pierre; Nguyen,Tung
EPI284478	HA	Vietnam	2008-May-01	A/chicken/Vietnam/NCVD-081/2008	National Centre of Veterinary Diagnostics	Centers for Disease Control and Prevention	Davis,Todd; Rivailler,Pierre; Nguyen,Tung
EPI284473	HA	Vietnam	2008-May-01	A/chicken/Vietnam/NCVD-03/2008	National Centre of Veterinary Diagnostics	Centers for Disease Control and Prevention	Davis,Todd; Rivailler,Pierre; Nguyen,Tung
EPI284471	HA	Vietnam	2009-Mar-01	A/chicken/Vietnam/NCVD-295/2009	National Centre of Veterinary Diagnostics	Centers for Disease Control and Prevention	Davis,Todd; Rivailler,Pierre; Nguyen,Tung
EPI284470	HA	Vietnam	2010-Mar-01	A/chicken/Vietnam/NCVD-421/2010	National Centre of Veterinary Diagnostics	Centers for Disease Control and Prevention	Davis,Todd; Rivailler,Pierre; Nguyen,Tung
EPI284467	HA	Vietnam	2010-Mar-01	A/chicken/Vietnam/NCVD-400/2010	National Centre of Veterinary Diagnostics	Centers for Disease Control and Prevention	Davis,Todd; Rivailler,Pierre; Nguyen,Tung
EPI284465	HA	Vietnam	2010-Feb-01	A/chicken/Vietnam/NCVD-397/2010	National Centre of Veterinary Diagnostics	Centers for Disease Control and Prevention	Davis,Todd; Rivailler,Pierre; Nguyen,Tung
EPI284464	HA	Vietnam	2010-Feb-01	A/chicken/Vietnam/NCVD-396/2010	National Centre of Veterinary Diagnostics	Centers for Disease Control and Prevention	Davis,Todd; Rivailler,Pierre; Nguyen,Tung
EPI284463	HA	Vietnam	2010-Feb-01	A/chicken/Vietnam/NCVD-395/2010	National Centre of Veterinary Diagnostics	Centers for Disease Control and Prevention	Davis,Todd; Rivailler,Pierre; Nguyen,Tung
EPI284462	HA	Vietnam	2010-Feb-01	A/chicken/Vietnam/NCVD-394/2010	National Centre of Veterinary Diagnostics	Centers for Disease Control and Prevention	Davis,Todd; Rivailler,Pierre; Nguyen,Tung
EPI284461	HA	Vietnam	2009-Feb-01	A/chicken/Vietnam/NCVD-292/2009	National Centre of Veterinary Diagnostics	Centers for Disease Control and Prevention	Davis,Todd; Rivailler,Pierre; Nguyen,Tung
EPI284460	HA	Vietnam	2009-Feb-01	A/chicken/Vietnam/NCVD-291/2009	National Centre of Veterinary Diagnostics	Centers for Disease Control and Prevention	Davis,Todd; Rivailler,Pierre; Nguyen,Tung
EPI284459	HA	Vietnam	2009-Feb-01	A/chicken/Vietnam/NCVD-290/2009	National Centre of Veterinary Diagnostics	Centers for Disease Control and Prevention	Davis,Todd; Rivailler,Pierre; Nguyen,Tung
EPI284458	HA	Vietnam	2009-Feb-01	A/chicken/Vietnam/NCVD-289/2009	National Centre of Veterinary Diagnostics	Centers for Disease Control and Prevention	Davis,Todd; Rivailler,Pierre; Nguyen,Tung
EPI284455	HA	Vietnam	2009-Feb-01	A/chicken/Vietnam/NCVD-282/2009	National Centre of Veterinary Diagnostics	Centers for Disease Control and Prevention	Davis,Todd; Rivailler,Pierre; Nguyen,Tung
EPI284454	HA	Vietnam	2009-Feb-01	A/chicken/Vietnam/NCVD-281/2009	National Centre of Veterinary Diagnostics	Centers for Disease Control and Prevention	Davis,Todd; Rivailler,Pierre; Nguyen,Tung
EPI284452	HA	Vietnam	2008-Dec-01	A/chicken/Vietnam/NCVD-188/2008	National Centre of Veterinary Diagnostics	Centers for Disease Control and Prevention	Davis,Todd; Rivailler,Pierre; Nguyen,Tung
EPI284451	HA	Vietnam	2008-Dec-01	A/chicken/Vietnam/NCVD-185/2008	National Centre of Veterinary Diagnostics	Centers for Disease Control and Prevention	Davis,Todd; Rivailler,Pierre; Nguyen,Tung
EPI284450	HA	Vietnam	2008-Dec-01	A/chicken/Vietnam/NCVD-138/2008	National Centre of Veterinary Diagnostics	Centers for Disease Control and Prevention	Davis,Todd; Rivailler,Pierre; Nguyen,Tung
EPI284449	HA	Vietnam	2008-Dec-01	A/chicken/Vietnam/NCVD-117/2008	National Centre of Veterinary Diagnostics	Centers for Disease Control and Prevention	Davis,Todd; Rivailler,Pierre; Nguyen,Tung
EPI284448	HA	Vietnam	2010-Apr-01	A/chicken/Vietnam/NCVD-412/2010	National Centre of Veterinary Diagnostics	Centers for Disease Control and Prevention	Davis,Todd; Rivailler,Pierre; Nguyen,Tung
EPI284446	HA	Vietnam	2010-Apr-01	A/chicken/Vietnam/NCVD-409/2010	National Centre of Veterinary Diagnostics	Centers for Disease Control and Prevention	Davis,Todd; Rivailler,Pierre; Nguyen,Tung
EPI284445	HA	Vietnam	2010-Apr-01	A/chicken/Vietnam/NCVD-407/2010	National Centre of Veterinary Diagnostics	Centers for Disease Control and Prevention	Davis,Todd; Rivailler,Pierre; Nguyen,Tung
EPI284442	HA	Vietnam	2010-Apr-01	A/chicken/Vietnam/NCVD-404/2010	National Centre of Veterinary Diagnostics	Centers for Disease Control and Prevention	Davis,Todd; Rivailler,Pierre; Nguyen,Tung
EPI284440	HA	Vietnam	2009-Jan-01	A/chicken/Vietnam/NCVD-390/2009	National Centre of Veterinary Diagnostics	Centers for Disease Control and Prevention	Davis,Todd; Rivailler,Pierre; Nguyen,Tung
EPI284439	HA	Vietnam	2009-Jan-01	A/chicken/Vietnam/NCVD-389/2009	National Centre of Veterinary Diagnostics	Centers for Disease Control and Prevention	Davis,Todd; Rivailler,Pierre; Nguyen,Tung
EPI284438	HA	Vietnam	2009-Jan-01	A/chicken/Vietnam/NCVD-392/2009	National Centre of Veterinary Diagnostics	Centers for Disease Control and Prevention	Davis,Todd; Rivailler,Pierre; Nguyen,Tung
EPI284437	HA	Vietnam	2009-Jan-01	A/chicken/Vietnam/NCVD-383/2009	National Centre of Veterinary Diagnostics	Centers for Disease Control and Prevention	Davis,Todd; Rivailler,Pierre; Nguyen,Tung
EPI284436	HA	Vietnam	2009-Jan-01	A/chicken/Vietnam/NCVD-381/2009	National Centre of Veterinary Diagnostics	Centers for Disease Control and Prevention	Davis,Todd; Rivailler,Pierre; Nguyen,Tung
EPI284435	HA	Vietnam	2009-Jan-01	A/chicken/Vietnam/NCVD-387/2009	National Centre of Veterinary Diagnostics	Centers for Disease Control and Prevention	Davis,Todd; Rivailler,Pierre; Nguyen,Tung
EPI284434	HA	Vietnam	2009-Jan-01	A/chicken/Vietnam/NCVD-385/2009	National Centre of Veterinary Diagnostics	Centers for Disease Control and Prevention	Davis,Todd; Rivailler,Pierre; Nguyen,Tung
EPI284432	HA	Vietnam	2009-Jan-01	A/chicken/Vietnam/NCVD-382/2009	National Centre of Veterinary Diagnostics	Centers for Disease Control and Prevention	Davis,Todd; Rivailler,Pierre; Nguyen,Tung
EPI284431	HA	Vietnam	2009-Jan-01	A/chicken/Vietnam/NCVD-380/2009	National Centre of Veterinary Diagnostics	Centers for Disease Control and Prevention	Davis,Todd; Rivailler,Pierre; Nguyen,Tung
EPI594492	HA	Bulgaria	2015-Jan-30	A/chicken/Bulgaria/5409/15	NDRVMI (National Diagnostic and Research Veterinary Medical Institute)	Animal and Plant Health Agency (APHA)	Thomas, S; Seekings, A; Essen, S; Manvell, R; Goujgoulova, G; Oreshkova, L; Banks, J; Brown, I
EPI594491	HA	Bulgaria	2015-Jan-30	A/chicken/Bulgaria/5408/15	NDRVMI (National Diagnostic and Research Veterinary Medical Institute)	Animal and Plant Health Agency (APHA)	Thomas, S; Seekings, A; Essen, S; Manvell, R; Goujgoulova, G; Oreshkova, L; Banks, J; Brown, I
EPI573337	HA	Egypt	2015-Jan-11	A/chicken/Egypt/1540S/2015		Friedrich-Loeffler-Institut	Arafa,A.M; Hagag, N; Elhusseiny, M.H; Yehia, N; Selim, A.A; Abdelhalim, A; Kilany, W.H; Samy, A; Hassan, M.K; Abdelwhab,E.M; Beer,M; Naguib, M.M; Harder,TC
EPI573333	HA	Egypt	2014-Sep-03	A/chicken/Egypt/1427AF/2014		Friedrich-Loeffler-Institut	Arafa,A.M; Hagag, N; Elhusseiny, M.H; Yehia, N; Selim, A.A; Abdelhalim, A; Kilany, W.H; Samy, A; Hassan, M.K; Abdelwhab,E.M; Beer,M; Naguib, M.M; Harder,TC
EPI573330	HA	Egypt	2014-Oct-14	A/chicken/Egypt/1476CA/2014		Friedrich-Loeffler-Institut	Arafa,A.M; Hagag, N; Elhusseiny, M.H; Yehia, N; Selim, A.A; Abdelhalim, A; Kilany, W.H; Samy, A; Hassan, M.K; Abdelwhab,E.M; Beer,M; Naguib, M.M; Harder,TC
EPI573326	HA	Egypt	2015-Jan-11	A/chicken/Egypt/152AI/2015		Friedrich-Loeffler-Institut	Arafa,A.M; Hagag, N; Elhusseiny, M.H; Yehia, N; Selim, A.A; Abdelhalim, A; Kilany, W.H; Samy, A; Hassan, M.K; Abdelwhab,E.M; Beer,M; Naguib, M.M; Harder,TC
EPI573322	HA	Egypt	2014-Dec-29	A/chicken/Egypt/152RS/2015		Friedrich-Loeffler-Institut	Arafa,A.M; Hagag, N; Elhusseiny, M.H; Yehia, N; Selim, A.A; Abdelhalim, A; Kilany, W.H; Samy, A; Hassan, M.K; Abdelwhab,E.M; Beer,M; Naguib, M.M; Harder,TC
EPI573321	HA	Egypt	2015-Jan-04	A/chicken/Egypt/153AF/2015		Friedrich-Loeffler-Institut	Arafa,A.M; Hagag, N; Elhusseiny, M.H; Yehia, N; Selim, A.A; Abdelhalim, A; Kilany, W.H; Samy, A; Hassan, M.K; Abdelwhab,E.M; Beer,M; Naguib, M.M; Harder,TC
EPI573318	HA	Egypt	2014-Nov-23	A/chicken/Egypt/1478CAL/2014		Friedrich-Loeffler-Institut	Arafa,A.M; Hagag, N; Elhusseiny, M.H; Yehia, N; Selim, A.A; Abdelhalim, A; Kilany, W.H; Samy, A; Hassan, M.K; Abdelwhab,E.M; Beer,M; Naguib, M.M; Harder,TC
EPI573314	HA	Egypt	2014-Dec-09	A/chicken/Egypt/14148CA/2014		Friedrich-Loeffler-Institut	Arafa,A.M; Hagag, N; Elhusseiny, M.H; Yehia, N; Selim, A.A; Abdelhalim, A; Kilany, W.H; Samy, A; Hassan, M.K; Abdelwhab,E.M; Beer,M; Naguib, M.M; Harder,TC
EPI573250	HA	Egypt	2014-Nov-12	A/chicken/Egypt/AR234-FAOF8NLQP/2014		Friedrich-Loeffler-Institut	Naguib, M.M; Arafa,A.M; Luttermann,C; Selim,A.A; Hassan,M.K; Beer,M;Harder,TC
EPI573248	HA	Egypt	2014-Dec-15	A/chicken/Egypt/AR233-S283NLQP/2014		Friedrich-Loeffler-Institut	Naguib, M.M; Arafa,A.M; Luttermann,C; Selim,A.A; Hassan,M.K; Beer,M;Harder,TC
EPI573244	HA	Egypt	2014-Nov-19	A/chicken/Egypt/AR231-CA113NLQP/2014		Friedrich-Loeffler-Institut	Naguib, M.M; Arafa,A.M; Luttermann,C; Selim,A.A; Hassan,M.K; Beer,M;Harder,TC
EPI573235	HA	Korea, Republic of	2014-Nov-24	A/Korean native chicken/Korea/H1903/2014		Animal and Plant Quarantine Agency	
EPI573233	HA	Korea, Republic of	2014-Nov-12	A/Korean native chicken/Korea/H1847/2014		Animal and Plant Quarantine Agency	
EPI573226	HA	Korea, Republic of	2014-Oct-01	A/Korean native chicken/Korea/H1747/2014		Animal and Plant Quarantine Agency	

EPI573218	HA	Korea, Republic of	2014-Jul-28	A/Korean native chicken/Korea/H1687/2014		Animal and Plant Quarantine Agency	
EPI573212	HA	Korea, Republic of	2014-Jun-16	A/Korean native chicken/Korea/H1554/2014		Animal and Plant Quarantine Agency	
EPI573207	HA	Korea, Republic of	2014-May-08	A/chicken/Korea/H1350/2014		Animal and Plant Quarantine Agency	
EPI573205	HA	Korea, Republic of	2014-Apr-23	A/Korean native chicken/Korea/H1299/2014		Animal and Plant Quarantine Agency	
EPI573202	HA	Korea, Republic of	2014-Apr-12	A/chicken/Korea/H1268/2014	Animal and Plant Quarantine Agency	Animal and Plant Quarantine Agency	
EPI573201	HA	Korea, Republic of	2014-Apr-07	A/chicken/Korea/H1236/2014		Animal and Plant Quarantine Agency	
EPI573199	HA	Korea, Republic of	2014-Mar-17	A/breeder chicken/Korea/H1068/2014		Animal and Plant Quarantine Agency	
EPI573194	HA	Korea, Republic of	2014-Mar-02	A/breeder chicken/Korea/H818/2014		Animal and Plant Quarantine Agency	
EPI573195	HA	Korea, Republic of	2014-Mar-02	A/breeder chicken/Korea/H818/2014		Animal and Plant Quarantine Agency	
EPI573171	HA	Netherlands	2014-Nov-20	A/Chicken/Netherlands/14015824/2014	Central Veterinary Institute	Central Veterinary Institute	Heutink, Rene; Harders, Frank; Verschuren-Pritz, Sylvia; Bossers, Alex; Koch, Guus; Bouwstra, Ruth
EPI557202	HA	Egypt	2013-May-09	A/chicken/Egypt/NLQP20SL-AR751/2013	National Laboratory for Veterinary Quality Control on Poultry production- Animal Health Research Inistitute	Friedrich-Loeffler-Institut	Naguib, M.M; Arafa, A.M; Selim,A.A; Hassan,M.K; Beer,M; Harder,TC
EPI557186	HA	Egypt	2013-Feb-20	A/chicken/Egypt/NLQP2AL-AR749/2013	National Laboratory for Veterinary Quality Control on Poultry production- Animal Health Research Inistitute	Friedrich-Loeffler-Institut	Naguib, M.M; Arafa, A.M; Selim,A.A; Hassan,M.K; Beer,M; Harder,TC
EPI557178	HA	Egypt	2013-Feb-06	A/chicken/Egypt/NLQP33SD-AR748/2013	National Laboratory for Veterinary Quality Control on Poultry production- Animal Health Research Inistitute	Friedrich-Loeffler-Institut	Naguib, M.M; Arafa, A.M; Selim,A.A; Hassan,M.K; Beer,M; Harder,TC
EPI557170	HA	Egypt	2013-Jan-14	A/chicken/Egypt/NLQP7FL-AR747/2013	National Laboratory for Veterinary Quality Control on Poultry production- Animal Health Research Inistitute	Friedrich-Loeffler-Institut	Naguib, M.M; Arafa, A.M; Selim,A.A; Hassan,M.K; Beer,M; Harder,TC
EPI556504	HA	Nigeria	2015-Jan-01	A/chicken/Nigeria/15VIR339-2/2015	National Veterinary Research Institute	Istituto Zooprofilattico Sperimentale Delle Venezie	Joannis, T.; Ahmed, M.; Meseko, C.; Shittu, I.; Solomon, P.; Luka, P.; Olorunshola, B.; Tassoni, L.; Schivo, A.; Ormelli, S.; Monne, I.
EPI553300	HA	China	2009-Sep-01	A/Chicken/Shandong/J06/2009	Qingdao Agricultural University	Beijing Institute of Microbiology and Epidemiology	yanbo,yin;dongdong,wang;linlin,liu;xiliang,wang;penghui,yang;xin,liu;keyu,wang;chengcai,lai
EPI553292	HA	China	2009-Sep-01	A/Chicken/Shandong/J05/2009	Qingdao Agricultural University	Beijing Institute of Microbiology and Epidemiology	yanbo,yin;dongdong,wang;linlin,liu;xiliang,wang;penghui,yang;xin,liu;keyu,wang;chengcai,lai
EPI553284	HA	China	2009-Sep-01	A/Chicken/Shandong/J04/2009	Qingdao Agricultural University	Beijing Institute of Microbiology and Epidemiology	yanbo,yin;dongdong,wang;linlin,liu;xiliang,wang;penghui,yang;xin,liu;keyu,wang;chengcai,lai
EPI553276	HA	China	2009-Sep-01	A/Chicken/Shandong/J03/2009	Qingdao Agricultural University	Beijing Institute of Microbiology and Epidemiology	yanbo,yin;dongdong,wang;linlin,liu;xiliang,wang;penghui,yang;xin,liu;keyu,wang;chengcai,lai
EPI538823	HA	Egypt	2013-May-01	A/chicken/Egypt/13VIR-2962-204/2013	Istituto Zooprofilattico Sperimentale Delle Venezie	Istituto Zooprofilattico Sperimentale Delle Venezie	Hussein, H.A.; El Hady, M.M.; Abd Hamid, H.S.; Sultan, H.A.; Abdel Hafez, A.; Fusaro, A.; Schivo, A.; Ormelli, S.; Monne, I.; Cattoli, G.
EPI538807	HA	Egypt	2013-May-01	A/chicken/Egypt/13VIR-2962-200/2013	Istituto Zooprofilattico Sperimentale Delle Venezie	Istituto Zooprofilattico Sperimentale Delle Venezie	Hussein, H.A.; El Hady, M.M.; Abd Hamid, H.S.; Sultan, H.A.; Abdel Hafez, A.; Fusaro, A.; Schivo, A.; Ormelli, S.; Monne, I.; Cattoli, G.
EPI538799	HA	Egypt	2013-May-01	A/chicken/Egypt/13VIR-2962-199/2013	Istituto Zooprofilattico Sperimentale Delle Venezie	Istituto Zooprofilattico Sperimentale Delle Venezie	Hussein, H.A.; El Hady, M.M.; Abd Hamid, H.S.; Sultan, H.A.; Abdel Hafez, A.; Fusaro, A.; Schivo, A.; Ormelli, S.; Monne, I.; Cattoli, G.
EPI538791	HA	Egypt	2013-May-01	A/chicken/Egypt/13VIR-2962-198/2013	Istituto Zooprofilattico Sperimentale Delle Venezie	Istituto Zooprofilattico Sperimentale Delle Venezie	Hussein, H.A.; El Hady, M.M.; Abd Hamid, H.S.; Sultan, H.A.; Abdel Hafez, A.; Fusaro, A.; Schivo, A.; Ormelli, S.; Monne, I.; Cattoli, G.
EPI538759	HA	Egypt	2013-Apr-01	A/chicken/Egypt/13VIR-2962-24/2013	Istituto Zooprofilattico Sperimentale Delle Venezie	Istituto Zooprofilattico Sperimentale Delle Venezie	Hussein, H.A.; El Hady, M.M.; Abd Hamid, H.S.; Sultan, H.A.; Abdel Hafez, A.; Fusaro, A.; Schivo, A.; Ormelli, S.; Monne, I.; Cattoli, G.
EPI475760	HA	Nepal	2013-Apr-16	A/chicken/Nepal/T-262/13	Central Veterinary Laboratory	Animal and Plant Health Agency (APHA)	Puranik, A; Hanna, A; Essen, S; Focosi-Snyman, R; Manvell, R; Jha, VC; Chapagain, S; Koirala, P; Air, TB; Reid, S
EPI475759	HA	Nepal	2013-Apr-16	A/chicken/Nepal/T-260/13	Central Veterinary Laboratory	Animal and Plant Health Agency (APHA)	Puranik, A; Hanna, A; Essen, S; Focosi-Snyman, R; Manvell, R; Jha, VC; Chapagain, S; Koirala, P; Air, TB; Reid, S
EPI475757	HA	Nepal	2013-Mar-30	A/chicken/Nepal/T-234/13	Central Veterinary Laboratory	Animal and Plant Health Agency (APHA)	Puranik, A; Hanna, A; Essen, S; Focosi-Snyman, R; Manvell, R; Jha, VC; Chapagain, S; Koirala, P; Air, TB; Reid, S
EPI475755	HA	Nepal	2013-Mar-30	A/chicken/Nepal/T-233/13	Central Veterinary Laboratory	Animal and Plant Health Agency (APHA)	Puranik, A; Hanna, A; Essen, S; Focosi-Snyman, R; Manvell, R; Jha, VC; Chapagain, S; Koirala, P; Air, TB; Reid, S
EPI464930	HA	Italy	2011-Dec-28	A/chicken/Italy/11VIR-7548/2011	Istituto Zooprofilattico Sperimentale Delle Venezie	Istituto Zooprofilattico Sperimentale Delle Venezie	Monne, I.; Salviato, A.; Tassoni, L.; Cattoli, G.
EPI446006	HA	Nepal	2013-Mar-31	A/chicken/Nepal/232/13	Central Veterinary Laboratory	Animal and Plant Health Agency (APHA)	Puranik, A; Hanna, A; Essen, S; Focosi-Snyman, R; Manvell, R; Manandhar, P; Reid, S
EPI446004	HA	Nepal	2013-Mar-31	A/chicken/Nepal/231/13	Central Veterinary Laboratory	Animal and Plant Health Agency (APHA)	Puranik, A; Hanna, A; Essen, S; Focosi-Snyman, R; Manvell, R; Manandhar, P; Reid, S
EPI446002	HA	Nepal	2013-Mar-28	A/chicken/Nepal/223/13	Central Veterinary Laboratory	Animal and Plant Health Agency (APHA)	Puranik, A; Hanna, A; Essen, S; Focosi-Snyman, R; Manvell, R; Manandhar, P; Reid, S
EPI446000	HA	Nepal	2013-Feb-28	A/chicken/Nepal/PT-30/13	Central Veterinary Laboratory	Animal and Plant Health Agency (APHA)	Puranik, A; Hanna, A; Essen, S; Focosi-Snyman, R; Manvell, R; Manandhar, P; Reid, S
EPI445998	HA	Nepal	2013-Feb-28	A/chicken/Nepal/PT-29/13	Central Veterinary Laboratory	Animal and Plant Health Agency (APHA)	Puranik, A; Hanna, A; Essen, S; Focosi-Snyman, R; Manvell, R; Manandhar, P; Reid, S
EPI432653	HA	Nepal	2012-Dec-18	A/chicken/Nepal/PT-17/12	Central Veterinary Laboratory	Animal and Plant Health Agency (APHA)	Puranik, A; Hanna, A; Essen, S; Focosi-Snyman, R; Manvell, R; Manandhar, P; Reid, S
EPI407283	HA	Nepal	2012-Oct-16	A/chicken/Nepal/PT-16/12	Central Veterinary Laboratory	Animal and Plant Health Agency (APHA)	Puranik, A; Hanna, A; Essen, S; Focosi-Snyman, R; Manvell, R; Bahadur Singh, D; Chapagain, S; Manandhar, S; Bahadur Air, T; Bahadur Kunwar, B; Reid, S
EPI356867	HA	Nepal	2011-Dec-21	A/chicken/Nepal/PT-1/12	Central Veterinary Laboratory	Animal and Plant Health Agency (APHA)	Collins, S; Hanna, A; Essen, S; Focosi-Snyman, R; Manvell, R.J; Sedai, D; Chapagain, S; Manandhar, S; Koirala, P; Karki, K.B; Pandey, K.R; Air, T.B; Reid, S
EPI354072	HA	Indonesia	2004-Jan-01	A/chicken/Indonesia/R132/2004	Friedrich-Loeffler-Institut	Robert-Koch-Institute	Zoehner, Andrea; Matthaei, Markus; Schweiger, Brunhilde; Wolff, Thorsten
EPI348166	HA	Egypt	2011-Jan-01	A/chicken/Egypt/11VIR4453-40/VRLCU/2011	Istituto Zooprofilattico Sperimentale Delle Venezie	Istituto Zooprofilattico Sperimentale Delle Venezie	Valastro, V.; Fusaro, A.; Monne, I.; Hussein, H.A.; Rohiam, M.; El Sanousi, A.A.; Abdel hamid, H.S.; Sultan, H.A.; Adel hafez, A.; Sedik, M.; Cattoli, G.
EPI348165	HA	Egypt	2011-Jan-01	A/chicken/Egypt/11VIR4453-203/VRLCU/2011	Istituto Zooprofilattico Sperimentale Delle Venezie	Istituto Zooprofilattico Sperimentale Delle Venezie	Valastro, V.; Fusaro, A.; Monne, I.; Hussein, H.A.; Rohiam, M.; El Sanousi, A.A.; Abdel hamid, H.S.; Sultan, H.A.; Adel hafez, A.; Sedik, M.; Cattoli, G.
EPI348163	HA	Egypt	2010-Jan-01	A/chicken/Egypt/11VIR4453-175/VRLCU/2010	Istituto Zooprofilattico Sperimentale Delle Venezie	Istituto Zooprofilattico Sperimentale Delle Venezie	Valastro, V.; Fusaro, A.; Monne, I.; Hussein, H.A.; Rohiam, M.; El Sanousi, A.A.; Abdel hamid, H.S.; Sultan, H.A.; Adel hafez, A.; Sedik, M.; Cattoli, G.
EPI348161	HA	Egypt	2010-Jan-01	A/chicken/Egypt/11VIR4453-9/VRLCU/2010	Istituto Zooprofilattico Sperimentale Delle Venezie	Istituto Zooprofilattico Sperimentale Delle Venezie	Valastro, V.; Fusaro, A.; Monne, I.; Hussein, H.A.; Rohiam, M.; El Sanousi, A.A.; Abdel hamid, H.S.; Sultan, H.A.; Adel hafez, A.; Sedik, M.; Cattoli, G.
EPI348160	HA	Egypt	2010-Jan-01	A/chicken/Egypt/11VIR4453-267/2010	Istituto Zooprofilattico Sperimentale Delle Venezie	Istituto Zooprofilattico Sperimentale Delle Venezie	Valastro, V.; Fusaro, A.; Monne, I.; Hussein, H.A.; Rohiam, M.; El Sanousi, A.A.; Abdel hamid, H.S.; Sultan, H.A.; Adel hafez, A.; Sedik, M.; Cattoli, G.
EPI348158	HA	Egypt	2011-Jan-01	A/chicken/Egypt/11VIR4453-109/VRLCU/2011	Istituto Zooprofilattico Sperimentale Delle Venezie	Istituto Zooprofilattico Sperimentale Delle Venezie	Valastro, V.; Fusaro, A.; Monne, I.; Hussein, H.A.; Rohiam, M.; El Sanousi, A.A.; Abdel hamid, H.S.; Sultan, H.A.; Adel hafez, A.; Sedik, M.; Cattoli, G.
EPI348157	HA	Egypt	2010-Jan-01	A/chicken/Egypt/11VIR4453-15/VRLCU/2010	Istituto Zooprofilattico Sperimentale Delle Venezie	Istituto Zooprofilattico Sperimentale Delle Venezie	Valastro, V.; Fusaro, A.; Monne, I.; Hussein, H.A.; Rohiam, M.; El Sanousi, A.A.; Abdel hamid, H.S.; Sultan, H.A.; Adel hafez, A.; Sedik, M.; Cattoli, G.
EPI348155	HA	Egypt	2011-Jan-01	A/chicken/Egypt/11VIR4453-68/VRLCU/2011	Istituto Zooprofilattico Sperimentale Delle Venezie	Istituto Zooprofilattico Sperimentale Delle Venezie	Valastro, V.; Fusaro, A.; Monne, I.; Hussein, H.A.; Rohiam, M.; El Sanousi, A.A.; Abdel hamid, H.S.; Sultan, H.A.; Adel hafez, A.; Sedik, M.; Cattoli, G.
EPI348152	HA	Egypt	2010-Jan-01	A/chicken/Egypt/11VIR4453-268/2010	Istituto Zooprofilattico Sperimentale Delle Venezie	Istituto Zooprofilattico Sperimentale Delle Venezie	Valastro, V.; Fusaro, A.; Monne, I.; Hussein, H.A.; Rohiam, M.; El Sanousi, A.A.; Abdel hamid, H.S.; Sultan, H.A.; Adel hafez, A.; Sedik, M.; Cattoli, G.
EPI348151	HA	Egypt	2010-Jan-01	A/chicken/Egypt/11VIR4453-270/2010	Istituto Zooprofilattico Sperimentale Delle Venezie	Istituto Zooprofilattico Sperimentale Delle Venezie	Valastro, V.; Fusaro, A.; Monne, I.; Hussein, H.A.; Rohiam, M.; El Sanousi, A.A.; Abdel hamid, H.S.; Sultan, H.A.; Adel hafez, A.; Sedik, M.; Cattoli, G.
EPI348150	HA	Egypt	2011-Jan-01	A/chicken/Egypt/11VIR4453-138/VRLCU/2011	Istituto Zooprofilattico Sperimentale Delle Venezie	Istituto Zooprofilattico Sperimentale Delle Venezie	Valastro, V.; Fusaro, A.; Monne, I.; Hussein, H.A.; Rohiam, M.; El Sanousi, A.A.; Abdel hamid, H.S.; Sultan, H.A.; Adel hafez, A.; Sedik, M.; Cattoli, G.

EPI348149	HA	Egypt	2010-Jan-01	A/chicken/Egypt/11VIR4453-159/VRLCU/2010	Istituto Zooprofilattico Sperimentale Delle Venezie	Istituto Zooprofilattico Sperimentale Delle Venezie	Valastro, V.; Fusaro, A.; Monne, I.; Hussein, H.A.; Rohiam, M.; El Sanousi, A.A.; Abdel hamid, H.S.; Sultan, H.A.; Adel hafez, A.; Sedik, M.; Cattoli, G.
EPI340804	HA	Indonesia	2009-Jan-01	A/chicken/Agam/BPPVRII-682/2009	Disease Investigation Centre Regional II Bukittinggi (BPPVRII)	CSIRO Australian Animal Health Laboratory	Miswati,Yuli; Yulfitria,.; Azfirman,.; Suatmodjo,Musny; Bruce,Kerri; Davies,Kelly R; Stevens,Vittoria; Kim,Mia; Daniels,Peter; Wong,Frank YK
EPI340802	HA	Indonesia	2009-Jan-01	A/chicken/Payakumbuh/BPPVRII-236/2009	Disease Investigation Centre Regional II Bukittinggi (BPPVRII)	CSIRO Australian Animal Health Laboratory	Miswati,Yuli; Yulfitria,.; Azfirman,.; Suatmodjo,Musny; Bruce,Kerri; Davies,Kelly R; Stevens,Vittoria; Kim,Mia; Daniels,Peter; Wong,Frank YK
EPI340800	HA	Indonesia	2009-Jan-01	A/chicken/Agam/BPPVRII-142/2009	Disease Investigation Centre Regional II Bukittinggi (BPPVRII)	CSIRO Australian Animal Health Laboratory	Miswati,Yuli; Yulfitria,.; Azfirman,.; Suatmodjo,Musny; Bruce,Kerri; Davies,Kelly R; Stevens,Vittoria; Kim,Mia; Daniels,Peter; Wong,Frank YK
EPI340798	HA	Indonesia	2009-Jan-01	A/chicken/Agam/BPPVRII-110/2009	Disease Investigation Centre Regional II Bukittinggi (BPPVRII)	CSIRO Australian Animal Health Laboratory	Miswati,Yuli; Yulfitria,.; Azfirman,.; Suatmodjo,Musny; Bruce,Kerri; Davies,Kelly R; Stevens,Vittoria; Kim,Mia; Daniels,Peter; Wong,Frank YK
EPI340796	HA	Indonesia	2010-Jan-01	A/chicken/Palu/BBVM-67/2010	Disease Investigation Centre Maros (BBVM)	CSIRO Australian Animal Health Laboratory	Hendrawati,Ferra; Poermadjaja,Bagoes; Suatmodjo,Musny; Bruce,Kerri; Davies,Kelly R; Stevens,Vittoria; Kim,Mia; Daniels,Peter; Wong,Frank YK
EPI340788	HA	Indonesia	2010-Jan-01	A/chicken/Klaten/BBVW-109-II/2010	Disease Investigation Centre Wates (BBVW)	CSIRO Australian Animal Health Laboratory	Dhamawan,Rama; Usman,Tri Bhakti; Junaidi,Akhmad; Suatmodjo,Musny; Bruce,Kerri; Davies,Kelly R; Stevens,Vittoria; Kim,Mia; Daniels,Peter; Wong,Frank YK
EPI340786	HA	Indonesia	2010-Jan-01	A/chicken/Jombang/BBVW-166-III/2010	Disease Investigation Centre Wates (BBVW)	CSIRO Australian Animal Health Laboratory	Dhamawan,Rama; Usman,Tri Bhakti; Junaidi,Akhmad; Suatmodjo,Musny; Bruce,Kerri; Davies,Kelly R; Stevens,Vittoria; Kim,Mia; Daniels,Peter; Wong,Frank YK
EPI340784	HA	Indonesia	2010-Jan-01	A/chicken/Sleman/BBVW-150-III/2010	Disease Investigation Centre Wates (BBVW)	CSIRO Australian Animal Health Laboratory	Dhamawan,Rama; Usman,Tri Bhakti; Junaidi,Akhmad; Suatmodjo,Musny; Bruce,Kerri; Davies,Kelly R; Stevens,Vittoria; Kim,Mia; Daniels,Peter; Wong,Frank YK
EPI340782	HA	Indonesia	2010-Jan-01	A/chicken/Sleman/BBVW-180-III/2010	Disease Investigation Centre Wates (BBVW)	CSIRO Australian Animal Health Laboratory	Dhamawan,Rama; Usman,Tri Bhakti; Junaidi,Akhmad; Suatmodjo,Musny; Bruce,Kerri; Davies,Kelly R; Stevens,Vittoria; Kim,Mia; Daniels,Peter; Wong,Frank YK
EPI340780	HA	Indonesia	2010-Jan-01	A/chicken/Lamongan/BBVW-170-III/2010	Disease Investigation Centre Wates (BBVW)	CSIRO Australian Animal Health Laboratory	Dhamawan,Rama; Usman,Tri Bhakti; Junaidi,Akhmad; Suatmodjo,Musny; Bruce,Kerri; Davies,Kelly R; Stevens,Vittoria; Kim,Mia; Daniels,Peter; Wong,Frank YK
EPI340774	HA	Indonesia	2009-Jan-01	A/chicken/Tasikmalaya/BBVW-991-VI/2009	Disease Investigation Centre Wates (BBVW)	CSIRO Australian Animal Health Laboratory	Dhamawan,Rama; Usman,Tri Bhakti; Junaidi,Akhmad; Suatmodjo,Musny; Bruce,Kerri; Davies,Kelly R; Stevens,Vittoria; Kim,Mia; Daniels,Peter; Wong,Frank YK
EPI340772	HA	Indonesia	2008-Jan-01	A/chicken/Wonogiri/BBVW-1563-XII/2008	Disease Investigation Centre Wates (BBVW)	CSIRO Australian Animal Health Laboratory	Dhamawan,Rama; Usman,Tri Bhakti; Junaidi,Akhmad; Suatmodjo,Musny; Bruce,Kerri; Davies,Kelly R; Stevens,Vittoria; Kim,Mia; Daniels,Peter; Wong,Frank YK
EPI340770	HA	Indonesia	2008-Jan-01	A/chicken/Banyumas/BBVW-1555b-XII/2008	Disease Investigation Centre Wates (BBVW)	CSIRO Australian Animal Health Laboratory	Dhamawan,Rama; Usman,Tri Bhakti; Junaidi,Akhmad; Suatmodjo,Musny; Bruce,Kerri; Davies,Kelly R; Stevens,Vittoria; Kim,Mia; Daniels,Peter; Wong,Frank YK
EPI340768	HA	Indonesia	2009-Jan-01	A/chicken/Kuningan/BBVW-1004-VI/2009	Disease Investigation Centre Wates (BBVW)	CSIRO Australian Animal Health Laboratory	Dhamawan,Rama; Usman,Tri Bhakti; Junaidi,Akhmad; Suatmodjo,Musny; Bruce,Kerri; Davies,Kelly R; Stevens,Vittoria; Kim,Mia; Daniels,Peter; Wong,Frank YK
EPI340766	HA	Indonesia	2009-Jan-01	A/chicken/Kudus/BBVW-1186-IX/2009	Disease Investigation Centre Wates (BBVW)	CSIRO Australian Animal Health Laboratory	Dhamawan,Rama; Usman,Tri Bhakti; Junaidi,Akhmad; Suatmodjo,Musny; Bruce,Kerri; Davies,Kelly R; Stevens,Vittoria; Kim,Mia; Daniels,Peter; Wong,Frank YK
EPI305607	HA	Turkey	2008-Feb-04	A/Chicken/Turkey-Sakarya/09rs2843-3/2008	Istituto Zooprofilattico Sperimentale Delle Venezie	Istituto Zooprofilattico Sperimentale Delle Venezie	Schivo,A.; Valastro,V.; Monne,I.; Coven,F.; Fusaro,A.; Dakman,A.; Akcadag,B.; Salviato,A.; DeBattisti,C.; Capua,I.; Cattoli,G.
EPI305604	HA	Turkey	2008-Jan-15	A/Chicken/Turkey-Zonguldak/09rs2842-112/2008	Istituto Zooprofilattico Sperimentale Delle Venezie	Istituto Zooprofilattico Sperimentale Delle Venezie	Schivo,A.; Valastro,V.; Monne,I.; Coven,F.; Fusaro,A.; Dakman,A.; Akcadag,B.; Salviato,A.; DeBattisti,C.; Capua,I.; Cattoli,G.
EPI305599	HA	Turkey	2007-Feb-15	A/Chicken/Turkey-Diyarbakir/09rs2842-100/2006	Istituto Zooprofilattico Sperimentale Delle Venezie	Istituto Zooprofilattico Sperimentale Delle Venezie	Schivo,A.; Valastro,V.; Monne,I.; Coven,F.; Fusaro,A.; Dakman,A.; Akcadag,B.; Salviato,A.; DeBattisti,C.; Capua,I.; Cattoli,G.
EPI305598	HA	Turkey	2007-Feb-15	A/Chicken/Turkey-Batman/09rs2842-96/2007	Istituto Zooprofilattico Sperimentale Delle Venezie	Istituto Zooprofilattico Sperimentale Delle Venezie	Schivo,A.; Valastro,V.; Monne,I.; Coven,F.; Fusaro,A.; Dakman,A.; Akcadag,B.; Salviato,A.; DeBattisti,C.; Capua,I.; Cattoli,G.
EPI305597	HA	Turkey	2007-Feb-15	A/Chicken/Turkey-Diyarbakir/09rs2842-94/2007	Istituto Zooprofilattico Sperimentale Delle Venezie	Istituto Zooprofilattico Sperimentale Delle Venezie	Schivo,A.; Valastro,V.; Monne,I.; Coven,F.; Fusaro,A.; Dakman,A.; Akcadag,B.; Salviato,A.; DeBattisti,C.; Capua,I.; Cattoli,G.
EPI305594	HA	Turkey	2007-Feb-15	A/Chicken/Turkey-Batman/09rs2842-90/2007	Istituto Zooprofilattico Sperimentale Delle Venezie	Istituto Zooprofilattico Sperimentale Delle Venezie	Schivo,A.; Valastro,V.; Monne,I.; Coven,F.; Fusaro,A.; Dakman,A.; Akcadag,B.; Salviato,A.; DeBattisti,C.; Capua,I.; Cattoli,G.
EPI305591	HA	Turkey	2006-Feb-20	A/Chicken/Turkey-Manisa/09rs2841-118/2006	Istituto Zooprofilattico Sperimentale Delle Venezie	Istituto Zooprofilattico Sperimentale Delle Venezie	Schivo,A.; Valastro,V.; Monne,I.; Coven,F.; Fusaro,A.; Dakman,A.; Akcadag,B.; Salviato,A.; DeBattisti,C.; Capua,I.; Cattoli,G.
EPI305590	HA	Turkey	2006-Mar-16	A/Chicken/Turkey-Elazig/09rs2841-117/2006	Istituto Zooprofilattico Sperimentale Delle Venezie	Istituto Zooprofilattico Sperimentale Delle Venezie	Schivo,A.; Valastro,V.; Monne,I.; Coven,F.; Fusaro,A.; Dakman,A.; Akcadag,B.; Salviato,A.; DeBattisti,C.; Capua,I.; Cattoli,G.
EPI305589	HA	Turkey	2006-Feb-08	A/Chicken/Turkey-Adiyaman/09rs2841-115/2006	Istituto Zooprofilattico Sperimentale Delle Venezie	Istituto Zooprofilattico Sperimentale Delle Venezie	Schivo,A.; Valastro,V.; Monne,I.; Coven,F.; Fusaro,A.; Dakman,A.; Akcadag,B.; Salviato,A.; DeBattisti,C.; Capua,I.; Cattoli,G.
EPI305588	HA	Turkey	2006-Feb-08	A/Chicken/Turkey-Diyarbakir/09rs2841-110/2006	Istituto Zooprofilattico Sperimentale Delle Venezie	Istituto Zooprofilattico Sperimentale Delle Venezie	Schivo,A.; Valastro,V.; Monne,I.; Coven,F.; Fusaro,A.; Dakman,A.; Akcadag,B.; Salviato,A.; DeBattisti,C.; Capua,I.; Cattoli,G.
EPI305586	HA	Turkey	2006-Feb-08	A/Chicken/Turkey-Mardin/09rs2841-107/2006	Istituto Zooprofilattico Sperimentale Delle Venezie	Istituto Zooprofilattico Sperimentale Delle Venezie	Schivo,A.; Valastro,V.; Monne,I.; Coven,F.; Fusaro,A.; Dakman,A.; Akcadag,B.; Salviato,A.; DeBattisti,C.; Capua,I.; Cattoli,G.
EPI305585	HA	Turkey	2006-Feb-14	A/Chicken/Turkey-Siirt/09rs2841-105/2006	Istituto Zooprofilattico Sperimentale Delle Venezie	Istituto Zooprofilattico Sperimentale Delle Venezie	Schivo,A.; Valastro,V.; Monne,I.; Coven,F.; Fusaro,A.; Dakman,A.; Akcadag,B.; Salviato,A.; DeBattisti,C.; Capua,I.; Cattoli,G.
EPI305583	HA	Turkey	2006-Feb-14	A/Chicken/Turkey-Tunceli/09rs2841-103/2006	Istituto Zooprofilattico Sperimentale Delle Venezie	Istituto Zooprofilattico Sperimentale Delle Venezie	Schivo,A.; Valastro,V.; Monne,I.; Coven,F.; Fusaro,A.; Dakman,A.; Akcadag,B.; Salviato,A.; DeBattisti,C.; Capua,I.; Cattoli,G.
EPI305582	HA	Turkey	2006-Feb-14	A/Chicken/Turkey-Tunceli/09rs2841-101/2006	Istituto Zooprofilattico Sperimentale Delle Venezie	Istituto Zooprofilattico Sperimentale Delle Venezie	Schivo,A.; Valastro,V.; Monne,I.; Coven,F.; Fusaro,A.; Dakman,A.; Akcadag,B.; Salviato,A.; DeBattisti,C.; Capua,I.; Cattoli,G.
EPI305581	HA	Turkey	2006-Feb-08	A/Chicken/Turkey-Malatya/09rs2841-97/2006	Istituto Zooprofilattico Sperimentale Delle Venezie	Istituto Zooprofilattico Sperimentale Delle Venezie	Schivo,A.; Valastro,V.; Monne,I.; Coven,F.; Fusaro,A.; Dakman,A.; Akcadag,B.; Salviato,A.; DeBattisti,C.; Capua,I.; Cattoli,G.
EPI305580	HA	Turkey	2006-Feb-14	A/Chicken/Turkey-Mus/09rs2841-94/2006	Istituto Zooprofilattico Sperimentale Delle Venezie	Istituto Zooprofilattico Sperimentale Delle Venezie	Schivo,A.; Valastro,V.; Monne,I.; Coven,F.; Fusaro,A.; Dakman,A.; Akcadag,B.; Salviato,A.; DeBattisti,C.; Capua,I.; Cattoli,G.
EPI305576	HA	Turkey	2006-Feb-14	A/Chicken/Turkey-Elazig/09rs2841-87/2006	Istituto Zooprofilattico Sperimentale Delle Venezie	Istituto Zooprofilattico Sperimentale Delle Venezie	Schivo,A.; Valastro,V.; Monne,I.; Coven,F.; Fusaro,A.; Dakman,A.; Akcadag,B.; Salviato,A.; DeBattisti,C.; Capua,I.; Cattoli,G.
EPI305575	HA	Turkey	2006-Feb-14	A/Chicken/Turkey-Elazig/09rs2841-86/2006	Istituto Zooprofilattico Sperimentale Delle Venezie	Istituto Zooprofilattico Sperimentale Delle Venezie	Schivo,A.; Valastro,V.; Monne,I.; Coven,F.; Fusaro,A.; Dakman,A.; Akcadag,B.; Salviato,A.; DeBattisti,C.; Capua,I.; Cattoli,G.
EPI305574	HA	Turkey	2006-Feb-08	A/Chicken/Turkey-Konya/09rs2841-85/2006	Istituto Zooprofilattico Sperimentale Delle Venezie	Istituto Zooprofilattico Sperimentale Delle Venezie	Schivo,A.; Valastro,V.; Monne,I.; Coven,F.; Fusaro,A.; Dakman,A.; Akcadag,B.; Salviato,A.; DeBattisti,C.; Capua,I.; Cattoli,G.
EPI305569	HA	Turkey	2006-Feb-08	A/Chicken/Turkey-Sanliurfa/09rs2841-80/2006	Istituto Zooprofilattico Sperimentale Delle Venezie	Istituto Zooprofilattico Sperimentale Delle Venezie	Schivo,A.; Valastro,V.; Monne,I.; Coven,F.; Fusaro,A.; Dakman,A.; Akcadag,B.; Salviato,A.; DeBattisti,C.; Capua,I.; Cattoli,G.
EPI305567	HA	Turkey	2006-Jan-26	A/Chicken/Turkey-Malatya/09rs2841-78/2006	Istituto Zooprofilattico Sperimentale Delle Venezie	Istituto Zooprofilattico Sperimentale Delle Venezie	Schivo,A.; Valastro,V.; Monne,I.; Coven,F.; Fusaro,A.; Dakman,A.; Akcadag,B.; Salviato,A.; DeBattisti,C.; Capua,I.; Cattoli,G.
EPI305566	HA	Turkey	2006-Jan-26	A/Chicken/Turkey-Diyarbakir/09rs2841-77/2006	Istituto Zooprofilattico Sperimentale Delle Venezie	Istituto Zooprofilattico Sperimentale Delle Venezie	Schivo,A.; Valastro,V.; Monne,I.; Coven,F.; Fusaro,A.; Dakman,A.; Akcadag,B.; Salviato,A.; DeBattisti,C.; Capua,I.; Cattoli,G.
EPI305565	HA	Turkey	2006-Jan-26	A/Chicken/Turkey-Diyarbakir/09rs2841-74/2006	Istituto Zooprofilattico Sperimentale Delle Venezie	Istituto Zooprofilattico Sperimentale Delle Venezie	Schivo,A.; Valastro,V.; Monne,I.; Coven,F.; Fusaro,A.; Dakman,A.; Akcadag,B.; Salviato,A.; DeBattisti,C.; Capua,I.; Cattoli,G.
EPI305563	HA	Turkey	2006-Jan-26	A/Chicken/Turkey-Batman/09rs2841-69/2006	Istituto Zooprofilattico Sperimentale Delle Venezie	Istituto Zooprofilattico Sperimentale Delle Venezie	Schivo,A.; Valastro,V.; Monne,I.; Coven,F.; Fusaro,A.; Dakman,A.; Akcadag,B.; Salviato,A.; DeBattisti,C.; Capua,I.; Cattoli,G.
EPI305562	HA	Turkey	2006-Jan-26	A/Chicken/Aydin/09rs2841-68/2006	Istituto Zooprofilattico Sperimentale Delle Venezie	Istituto Zooprofilattico Sperimentale Delle Venezie	Schivo,A.; Valastro,V.; Monne,I.; Coven,F.; Fusaro,A.; Dakman,A.; Akcadag,B.; Salviato,A.; DeBattisti,C.; Capua,I.; Cattoli,G.
EPI305560	HA	Turkey	2006-Jan-27	A/Chicken/Turkey-Burdur/09rs2841-64/2006	Istituto Zooprofilattico Sperimentale Delle Venezie	Istituto Zooprofilattico Sperimentale Delle Venezie	Schivo,A.; Valastro,V.; Monne,I.; Coven,F.; Fusaro,A.; Dakman,A.; Akcadag,B.; Salviato,A.; DeBattisti,C.; Capua,I.; Cattoli,G.
EPI305557	HA	Turkey	2006-Jan-23	A/Chicken/Turkey-Mugla/09rs2841-54/2006	Istituto Zooprofilattico Sperimentale Delle Venezie	Istituto Zooprofilattico Sperimentale Delle Venezie	Schivo,A.; Valastro,V.; Monne,I.; Coven,F.; Fusaro,A.; Dakman,A.; Akcadag,B.; Salviato,A.; DeBattisti,C.; Capua,I.; Cattoli,G.

EPI305556	HA	Turkey	2006-Jan-23	A/Chicken/Turkey-Elazi/09rs2841-52/2006	Istituto Zooprofilattico Sperimentale Delle Venezie	Istituto Zooprofilattico Sperimentale Delle Venezie	Schivo,A.; Valastro,V.; Monne,I.; Coven,F.; Fusaro,A.; Dakman,A.; Akcadag,B.; Salviato,A.; DeBattisti,C.; Capua,I.; Cattoli,G.
EPI305553	HA	Turkey	2006-Jan-23	A/Chicken/Turkey-Gunushane/09rs2841-41/2006	Istituto Zooprofilattico Sperimentale Delle Venezie	Istituto Zooprofilattico Sperimentale Delle Venezie	Schivo,A.; Valastro,V.; Monne,I.; Coven,F.; Fusaro,A.; Dakman,A.; Akcadag,B.; Salviato,A.; DeBattisti,C.; Capua,I.; Cattoli,G.
EPI305552	HA	Turkey	2006-Jan-12	A/Chicken/Turkey-Yozgat/09rs2841-35/2006	Istituto Zooprofilattico Sperimentale Delle Venezie	Istituto Zooprofilattico Sperimentale Delle Venezie	Schivo,A.; Valastro,V.; Monne,I.; Coven,F.; Fusaro,A.; Dakman,A.; Akcadag,B.; Salviato,A.; DeBattisti,C.; Capua,I.; Cattoli,G.
EPI305549	HA	Turkey	2006-Jan-07	A/Chicken/Turkey-Yozgat/09rs2841-26/2006	Istituto Zooprofilattico Sperimentale Delle Venezie	Istituto Zooprofilattico Sperimentale Delle Venezie	Schivo,A.; Valastro,V.; Monne,I.; Coven,F.; Fusaro,A.; Dakman,A.; Akcadag,B.; Salviato,A.; DeBattisti,C.; Capua,I.; Cattoli,G.
EPI305546	HA	Turkey	2006-Jan-07	A/Chicken/Turkey-Agri/09rs2841-22/2006	Istituto Zooprofilattico Sperimentale Delle Venezie	Istituto Zooprofilattico Sperimentale Delle Venezie	Schivo,A.; Valastro,V.; Monne,I.; Coven,F.; Fusaro,A.; Dakman,A.; Akcadag,B.; Salviato,A.; DeBattisti,C.; Capua,I.; Cattoli,G.
EPI305541	HA	Turkey	2006-Jan-06	A/Chicken/Turkey-Agri/09rs2841-12/2006	Istituto Zooprofilattico Sperimentale Delle Venezie	Istituto Zooprofilattico Sperimentale Delle Venezie	Schivo,A.; Valastro,V.; Monne,I.; Coven,F.; Fusaro,A.; Dakman,A.; Akcadag,B.; Salviato,A.; DeBattisti,C.; Capua,I.; Cattoli,G.
EPI305540	HA	Turkey	2005-Dec-11	A/Chicken/Turkey-Agri/09rs2841-10/2005	Istituto Zooprofilattico Sperimentale Delle Venezie	Istituto Zooprofilattico Sperimentale Delle Venezie	Schivo,A.; Valastro,V.; Monne,I.; Coven,F.; Fusaro,A.; Dakman,A.; Akcadag,B.; Salviato,A.; DeBattisti,C.; Capua,I.; Cattoli,G.
EPI293960	HA	Turkey	2008-Mar-14	A/Chicken/Turkey-Edirne/09rs2843-5/2008	Istituto Zooprofilattico Sperimentale Delle Venezie	Istituto Zooprofilattico Sperimentale Delle Venezie	Schivo,A.; Valastro,V.; Monne,I.; Coven,F.; Fusaro,A.; Dakman,A.; Akcadag,B.; Salviato,A.; DeBattisti,C.; Capua,I.; Cattoli,G.
EPI293952	HA	Turkey	2007-Feb-01	A/Chicken/Turkey-Batman/09rs2842-92/2007	Istituto Zooprofilattico Sperimentale Delle Venezie	Istituto Zooprofilattico Sperimentale Delle Venezie	Schivo,A.; Valastro,V.; Monne,I.; Coven,F.; Fusaro,A.; Dakman,A.; Akcadag,B.; Salviato,A.; DeBattisti,C.; Capua,I.; Cattoli,G.
EPI293944	HA	Turkey	2006-Feb-14	A/Chicken/Turkey-Bitlis/09rs2841-93/2006	Istituto Zooprofilattico Sperimentale Delle Venezie	Istituto Zooprofilattico Sperimentale Delle Venezie	Schivo,A.; Valastro,V.; Monne,I.; Coven,F.; Fusaro,A.; Dakman,A.; Akcadag,B.; Salviato,A.; DeBattisti,C.; Capua,I.; Cattoli,G.
EPI293936	HA	Turkey	2006-Jan-26	A/Chicken/Turkey-Batman/09rs2841-71/2006	Istituto Zooprofilattico Sperimentale Delle Venezie	Istituto Zooprofilattico Sperimentale Delle Venezie	Schivo,A.; Valastro,V.; Monne,I.; Coven,F.; Fusaro,A.; Dakman,A.; Akcadag,B.; Salviato,A.; DeBattisti,C.; Capua,I.; Cattoli,G.
EPI293928	HA	Turkey	2006-Jan-23	A/Chicken/Turkey-Mus/09rs2841-51/2006	Istituto Zooprofilattico Sperimentale Delle Venezie	Istituto Zooprofilattico Sperimentale Delle Venezie	Schivo,A.; Valastro,V.; Monne,I.; Coven,F.; Fusaro,A.; Dakman,A.; Akcadag,B.; Salviato,A.; DeBattisti,C.; Capua,I.; Cattoli,G.
EPI293920	HA	Turkey	2006-Jan-23	A/Chicken/Turkey-Mus/09rs2841-50/2006	Istituto Zooprofilattico Sperimentale Delle Venezie	Istituto Zooprofilattico Sperimentale Delle Venezie	Schivo,A.; Valastro,V.; Monne,I.; Coven,F.; Fusaro,A.; Dakman,A.; Akcadag,B.; Salviato,A.; DeBattisti,C.; Capua,I.; Cattoli,G.
EPI287382	HA	Egypt	2010-Feb-02	A/chicken/Egypt/3982-78/2010	Istituto Zooprofilattico Sperimentale Delle Venezie	Istituto Zooprofilattico Sperimentale Delle Venezie	
EPI287381	HA	Egypt	2010-May-10	A/poultry/Egypt/3982-56/2010	Istituto Zooprofilattico Sperimentale Delle Venezie	Istituto Zooprofilattico Sperimentale Delle Venezie	
EPI287374	HA	Egypt	2010-Jul-02	A/chicken/Egypt/3982-20/2010	Istituto Zooprofilattico Sperimentale Delle Venezie	Istituto Zooprofilattico Sperimentale Delle Venezie	
EPI287373	HA	Egypt	2010-Jul-02	A/chicken/Egypt/3982-19/2010	Istituto Zooprofilattico Sperimentale Delle Venezie	Istituto Zooprofilattico Sperimentale Delle Venezie	
EPI287372	HA	Egypt	2010-Apr-21	A/chicken/Egypt/3982-14/2010	Istituto Zooprofilattico Sperimentale Delle Venezie	Istituto Zooprofilattico Sperimentale Delle Venezie	
EPI287367	HA	Egypt	2010-Apr-11	A/chicken/Egypt/3982-5/2010	Istituto Zooprofilattico Sperimentale Delle Venezie	Istituto Zooprofilattico Sperimentale Delle Venezie	
EPI287365	HA	Egypt	2010-Feb-01	A/chicken/Egypt/3982-3/2010	Istituto Zooprofilattico Sperimentale Delle Venezie	Istituto Zooprofilattico Sperimentale Delle Venezie	
EPI287363	HA	Egypt	2010-Mar-27	A/chicken/Egypt/2095-75/2010	Istituto Zooprofilattico Sperimentale Delle Venezie	Istituto Zooprofilattico Sperimentale Delle Venezie	
EPI287362	HA	Egypt	2010-Mar-25	A/chicken/Egypt/2095-73/2010	Istituto Zooprofilattico Sperimentale Delle Venezie	Istituto Zooprofilattico Sperimentale Delle Venezie	
EPI287361	HA	Egypt	2010-Mar-23	A/chicken/Egypt/2095-70/2010	Istituto Zooprofilattico Sperimentale Delle Venezie	Istituto Zooprofilattico Sperimentale Delle Venezie	
EPI287360	HA	Egypt	2010-Jan-11	A/chicken/Egypt/2095-68/2010	Istituto Zooprofilattico Sperimentale Delle Venezie	Istituto Zooprofilattico Sperimentale Delle Venezie	
EPI287359	HA	Egypt	2010-Jan-31	A/chicken/Egypt/2095-65/2010	Istituto Zooprofilattico Sperimentale Delle Venezie	Istituto Zooprofilattico Sperimentale Delle Venezie	
EPI287354	HA	Egypt	2010-Feb-01	A/chicken/Egypt/1553-28/2010	Istituto Zooprofilattico Sperimentale Delle Venezie	Istituto Zooprofilattico Sperimentale Delle Venezie	
EPI287352	HA	Egypt	2010-Feb-01	A/chicken/Egypt/1553-15/2010	Istituto Zooprofilattico Sperimentale Delle Venezie	Istituto Zooprofilattico Sperimentale Delle Venezie	
EPI287351	HA	Egypt	2010-Feb-01	A/chicken/Egypt/1553-13/2010	Istituto Zooprofilattico Sperimentale Delle Venezie	Istituto Zooprofilattico Sperimentale Delle Venezie	
EPI287349	HA	Egypt	2010-Feb-01	A/chicken/Egypt/1553-1/2010	Istituto Zooprofilattico Sperimentale Delle Venezie	Istituto Zooprofilattico Sperimentale Delle Venezie	
EPI210057	HA	Russian Federation	2006-Mar-01	A/chicken/Adygea/203/06	Federal Centre for Animal Health (ARRIAH)	Istituto Zooprofilattico Sperimentale Delle Venezie	
EPI210035	HA	Russian Federation	2007-Dec-01	A/chicken/Rostov/864/07	Federal Centre for Animal Health (ARRIAH)	Istituto Zooprofilattico Sperimentale Delle Venezie	
EPI210009	HA	Russian Federation	2006-Mar-01	A/chicken/Volgograd/236/06	Federal Centre for Animal Health (ARRIAH)	Istituto Zooprofilattico Sperimentale Delle Venezie	
EPI210001	HA	Russian Federation	2007-Sep-01	A/chicken/Krasnodar/776/2007	Federal Centre for Animal Health (ARRIAH)	Istituto Zooprofilattico Sperimentale Delle Venezie	
EPI169518	HA	Saudi Arabia	2008-Jan-01	A/chicken/Saudi Arabia/3489-79/2008		Istituto Zooprofilattico Sperimentale Delle Venezie	
EPI169517	HA	Saudi Arabia	2007-Jan-01	A/chicken/Saudi Arabia/3489-75VIR08/2007		Istituto Zooprofilattico Sperimentale Delle Venezie	
EPI169512	HA	Saudi Arabia	2007-Jan-01	A/chicken/Saudi Arabia/3489-60VIR08/2007		Istituto Zooprofilattico Sperimentale Delle Venezie	
EPI169424	HA	Belgium	1999-Jun-29	A/chicken/Belgium/150VB/1999		Veterinary and Agrochemical Research Institute	
EPI164747	HA	China	2004-Jan-01	A/Chicken/Anhui/39/2004	Harbin Veterinary Research Institute	Harbin Veterinary Research Institute	
EPI164731	HA	China	2004-Jan-01	A/Chicken/Jiangxi/25/2004	Harbin Veterinary Research Institute	Harbin Veterinary Research Institute	
EPI159887	HA	Egypt	2007-Dec-18	A/chicken/Egypt/1709-8VIR08/2007		Istituto Zooprofilattico Sperimentale Delle Venezie	
EPI156788	HA	Benin	2007-Dec-01	A/chicken/Benin/6693-1/2007		Istituto Zooprofilattico Sperimentale Delle Venezie	
EPI156784	HA	Egypt	2007-Jun-01	A/chicken/Egypt/5169-5/2007		Istituto Zooprofilattico Sperimentale Delle Venezie	
EPI156768	HA	Egypt	2007-Jun-01	A/chicken/Egypt/5169-3/2007		Istituto Zooprofilattico Sperimentale Delle Venezie	
EPI156760	HA	Togo	2007-Jan-01	A/chicken/Togo/4106-4/2007		Istituto Zooprofilattico Sperimentale Delle Venezie	

EPI156750	HA	Egypt	2007-Apr-01	A/chicken/Egypt/2628-4/2007		Istituto Zooprofilattico Sperimentale Delle Venezie	
EPI154914	HA	Egypt	2007-Jan-01	A/chicken/Egypt/1709-2/2008		Istituto Zooprofilattico Sperimentale Delle Venezie	
EPI154525	HA	Saudi Arabia	2007-Jan-01	A/chicken/Saudi Arabia/6732-18/2007		Istituto Zooprofilattico Sperimentale Delle Venezie	
EPI154517	HA	Saudi Arabia	2007-Jan-01	A/chicken/Saudi Arabia/6732-13/2007		Istituto Zooprofilattico Sperimentale Delle Venezie	
EPI154501	HA	Saudi Arabia	2007-Jan-01	A/chicken/Saudi Arabia/6732-4/2007		Istituto Zooprofilattico Sperimentale Delle Venezie	
EPI152044	HA	Egypt	2007-Jan-01	A/chicken/Egypt/5169-2/2007		Istituto Zooprofilattico Sperimentale Delle Venezie	
EPI628911	HA	Egypt	2015-Jan-07	A/chicken/F611/Egypt/2015(H5N1)	National Laboratory for Veterinary Quality Control on Poultry production- Animal Health Research Iinstitute	Faculty of Veterinary - Zagazig University	
EPI586550	HA	Canada	2014-Dec-18	A/chicken/BC/FAV24/2014	Animal Health Centre, Ministry of Agriculture	Canadian Food Inspection Agency	
EPI586542	HA	Canada	2014-Dec-15	A/chicken/BC/FAV23/2014	Animal Health Centre, Ministry of Agriculture	Canadian Food Inspection Agency	
EPI586526	HA	Canada	2014-Dec-11	A/chicken/BC/FAV21/2014	Animal Health Centre, Ministry of Agriculture	Canadian Food Inspection Agency	
EPI586518	HA	Canada	2014-Dec-08	A/chicken/BC/FAV20/2014	Animal Health Centre, Ministry of Agriculture	Canadian Food Inspection Agency	
EPI579783	HA	Egypt	2015-Jan-04	A/chicken/Egypt/CLEVB-21_N00235/2015	The Central Laboratory for Evaluation of Veterinary Biologics (CLEVB)	NAMRU-3	Younan, M.; Defang, G.; Mohareb, E.; Ali, A.M.; Nassif, S.A.; Mourad,A.A.; Fouad, E.M.; Ragab, A.S.; Khelfa, D.G.
EPI579782	HA	Egypt	2015-Jan-11	A/chicken/Egypt/CLEVB-20_N00234/2015	The Central Laboratory for Evaluation of Veterinary Biologics (CLEVB)	NAMRU-3	Younan, M.; Defang, G.; Mohareb, E.; Ali, A.M.; Nassif, S.A.; Mourad,A.A.; Fouad, E.M.; Ragab, A.S.; Khelfa, D.G.
EPI579520	HA	Egypt	2015-Jan-20	A/chicken/Egypt/CLEVB-17_N00231/2015	The Central Laboratory for Evaluation of Veterinary Biologics (CLEVB)	NAMRU-3	Younan, M.; Defang, G.; Mohareb, E.; Ali, A.M.; Nassif, S.A.; Mourad,A.A.; Fouad, E.M.; Ragab, A.S.; Khelfa, D.G.
EPI579006	HA	Egypt	2014-Dec-29	A/chicken/Egypt/CLEVB-1_N00215/2014	The Central Laboratory for Evaluation of Veterinary Biologics (CLEVB)	NAMRU-3	Younan, M.; Defang, G.; Mohareb, E.; Ali, A.M.; Nassif, S.A.; Mourad,A.A.; Fouad, E.M.; Ragab, A.S.; Khelfa, D.G.
EPI573187	HA	Netherlands	2014-Nov-29	A/chicken/Netherlands/14016437/2014	Central Veterinary Institute	Central Veterinary Institute	Heutink, Rene; Harders, Frank; Verschuren-Pritz, Sylvia; Bossers, Alex; Koch, Guus; Bouwstra, Ruth
EPI553343	HA	Japan	2014-Dec-16	A/chicken/Miyazaki/7/2014		National Institute of Animal Health	
EPI533459	HA	Indonesia	2010-Jan-01	A/chicken/North Sumatra/072/2010	Dr. Teguh Y. Prajitno	Erasmus Medical Center	Koel,B.F.; van der Vliet,S.; Burke,D.F.; Bestebroer,T.M.; Bharoto,E.E.; Yasa,I.W.; Herliana,I.; Laksono,B.M.; Xu,K.; Skepner,E.; Russell,C.A.; Rimmelzwaan,G.F.; Perez,D.R.; Osterhaus,A.D.; Smith,D.J.; Prajitno,T.Y.; Fouchier,R.A.; Yasa,I.W.W.; Osterhaus,A.D.M.E.; Fouchier,R.A.M.
EPI533454	HA	Indonesia	2008-Jan-01	A/chicken/Lampung/7/2008	Dr. Teguh Y. Prajitno	Erasmus Medical Center	Koel,B.F.; van der Vliet,S.; Burke,D.F.; Bestebroer,T.M.; Bharoto,E.E.; Yasa,I.W.; Herliana,I.; Laksono,B.M.; Xu,K.; Skepner,E.; Russell,C.A.; Rimmelzwaan,G.F.; Perez,D.R.; Osterhaus,A.D.; Smith,D.J.; Prajitno,T.Y.; Fouchier,R.A.; Yasa,I.W.W.; Osterhaus,A.D.M.E.; Fouchier,R.A.M.
EPI533453	HA	Indonesia	2010-Jan-01	A/chicken/Lampung/092/2010	Dr. Teguh Y. Prajitno	Erasmus Medical Center	Koel,B.F.; van der Vliet,S.; Burke,D.F.; Bestebroer,T.M.; Bharoto,E.E.; Yasa,I.W.; Herliana,I.; Laksono,B.M.; Xu,K.; Skepner,E.; Russell,C.A.; Rimmelzwaan,G.F.; Perez,D.R.; Osterhaus,A.D.; Smith,D.J.; Prajitno,T.Y.; Fouchier,R.A.; Yasa,I.W.W.; Osterhaus,A.D.M.E.; Fouchier,R.A.M.
EPI533452	HA	Indonesia	2011-Jan-01	A/chicken/Lampung/153/2011	Dr. Teguh Y. Prajitno	Erasmus Medical Center	Koel,B.F.; van der Vliet,S.; Burke,D.F.; Bestebroer,T.M.; Bharoto,E.E.; Yasa,I.W.; Herliana,I.; Laksono,B.M.; Xu,K.; Skepner,E.; Russell,C.A.; Rimmelzwaan,G.F.; Perez,D.R.; Osterhaus,A.D.; Smith,D.J.; Prajitno,T.Y.; Fouchier,R.A.; Yasa,I.W.W.; Osterhaus,A.D.M.E.; Fouchier,R.A.M.
EPI533451	HA	Indonesia	2006-Jan-01	A/chicken/West Java/CSLK-EC/2006	Dr. Teguh Y. Prajitno	Erasmus Medical Center	Koel,B.F.; van der Vliet,S.; Burke,D.F.; Bestebroer,T.M.; Bharoto,E.E.; Yasa,I.W.; Herliana,I.; Laksono,B.M.; Xu,K.; Skepner,E.; Russell,C.A.; Rimmelzwaan,G.F.; Perez,D.R.; Osterhaus,A.D.; Smith,D.J.; Prajitno,T.Y.; Fouchier,R.A.; Yasa,I.W.W.; Osterhaus,A.D.M.E.; Fouchier,R.A.M.
EPI533449	HA	Indonesia	2009-Jan-01	A/chicken/West Java/6-2/2009	Dr. Teguh Y. Prajitno	Erasmus Medical Center	Koel,B.F.; van der Vliet,S.; Burke,D.F.; Bestebroer,T.M.; Bharoto,E.E.; Yasa,I.W.; Herliana,I.; Laksono,B.M.; Xu,K.; Skepner,E.; Russell,C.A.; Rimmelzwaan,G.F.; Perez,D.R.; Osterhaus,A.D.; Smith,D.J.; Prajitno,T.Y.; Fouchier,R.A.; Yasa,I.W.W.; Osterhaus,A.D.M.E.; Fouchier,R.A.M.
EPI533446	HA	Indonesia	2010-Jan-01	A/chicken/West Java/126/2010	Dr. Teguh Y. Prajitno	Erasmus Medical Center	Koel,B.F.; van der Vliet,S.; Burke,D.F.; Bestebroer,T.M.; Bharoto,E.E.; Yasa,I.W.; Herliana,I.; Laksono,B.M.; Xu,K.; Skepner,E.; Russell,C.A.; Rimmelzwaan,G.F.; Perez,D.R.; Osterhaus,A.D.; Smith,D.J.; Prajitno,T.Y.; Fouchier,R.A.; Yasa,I.W.W.; Osterhaus,A.D.M.E.; Fouchier,R.A.M.
EPI533445	HA	Indonesia	2011-Jan-01	A/chicken/West Java/148/2011	Dr. Teguh Y. Prajitno	Erasmus Medical Center	Koel,B.F.; van der Vliet,S.; Burke,D.F.; Bestebroer,T.M.; Bharoto,E.E.; Yasa,I.W.; Herliana,I.; Laksono,B.M.; Xu,K.; Skepner,E.; Russell,C.A.; Rimmelzwaan,G.F.; Perez,D.R.; Osterhaus,A.D.; Smith,D.J.; Prajitno,T.Y.; Fouchier,R.A.; Yasa,I.W.W.; Osterhaus,A.D.M.E.; Fouchier,R.A.M.
EPI533444	HA	Indonesia	2009-Jan-01	A/chicken/West Java/59A/2009	Dr. Teguh Y. Prajitno	Erasmus Medical Center	Koel,B.F.; van der Vliet,S.; Burke,D.F.; Bestebroer,T.M.; Bharoto,E.E.; Yasa,I.W.; Herliana,I.; Laksono,B.M.; Xu,K.; Skepner,E.; Russell,C.A.; Rimmelzwaan,G.F.; Perez,D.R.; Osterhaus,A.D.; Smith,D.J.; Prajitno,T.Y.; Fouchier,R.A.; Yasa,I.W.W.; Osterhaus,A.D.M.E.; Fouchier,R.A.M.
EPI533443	HA	Indonesia	2006-Jan-01	A/chicken/West Java/8/2006	Dr. Teguh Y. Prajitno	Erasmus Medical Center	Koel,B.F.; van der Vliet,S.; Burke,D.F.; Bestebroer,T.M.; Bharoto,E.E.; Yasa,I.W.; Herliana,I.; Laksono,B.M.; Xu,K.; Skepner,E.; Russell,C.A.; Rimmelzwaan,G.F.; Perez,D.R.; Osterhaus,A.D.; Smith,D.J.; Prajitno,T.Y.; Fouchier,R.A.; Yasa,I.W.W.; Osterhaus,A.D.M.E.; Fouchier,R.A.M.
EPI533442	HA	Indonesia	2006-Jan-01	A/chicken/West Java/12/2006	Dr. Teguh Y. Prajitno	Erasmus Medical Center	Koel,B.F.; van der Vliet,S.; Burke,D.F.; Bestebroer,T.M.; Bharoto,E.E.; Yasa,I.W.; Herliana,I.; Laksono,B.M.; Xu,K.; Skepner,E.; Russell,C.A.; Rimmelzwaan,G.F.; Perez,D.R.; Osterhaus,A.D.; Smith,D.J.; Prajitno,T.Y.; Fouchier,R.A.; Yasa,I.W.W.; Osterhaus,A.D.M.E.; Fouchier,R.A.M.
EPI533441	HA	Indonesia	2007-Jan-01	A/chicken/West Java/29/002/2007	Dr. Teguh Y. Prajitno	Erasmus Medical Center	Koel,B.F.; van der Vliet,S.; Burke,D.F.; Bestebroer,T.M.; Bharoto,E.E.; Yasa,I.W.; Herliana,I.; Laksono,B.M.; Xu,K.; Skepner,E.; Russell,C.A.; Rimmelzwaan,G.F.; Perez,D.R.; Osterhaus,A.D.; Smith,D.J.; Prajitno,T.Y.; Fouchier,R.A.; Yasa,I.W.W.; Osterhaus,A.D.M.E.; Fouchier,R.A.M.
EPI533438	HA	Indonesia	2008-Jan-01	A/chicken/West Java/2008	Dr. Teguh Y. Prajitno	Erasmus Medical Center	Koel,B.F.; van der Vliet,S.; Burke,D.F.; Bestebroer,T.M.; Bharoto,E.E.; Yasa,I.W.; Herliana,I.; Laksono,B.M.; Xu,K.; Skepner,E.; Russell,C.A.; Rimmelzwaan,G.F.; Perez,D.R.; Osterhaus,A.D.; Smith,D.J.; Prajitno,T.Y.; Fouchier,R.A.; Yasa,I.W.W.; Osterhaus,A.D.M.E.; Fouchier,R.A.M.
EPI533437	HA	Indonesia	2009-Jan-01	A/chicken/West Java/068/2009	Dr. Teguh Y. Prajitno	Erasmus Medical Center	Koel,B.F.; van der Vliet,S.; Burke,D.F.; Bestebroer,T.M.; Bharoto,E.E.; Yasa,I.W.; Herliana,I.; Laksono,B.M.; Xu,K.; Skepner,E.; Russell,C.A.; Rimmelzwaan,G.F.; Perez,D.R.; Osterhaus,A.D.; Smith,D.J.; Prajitno,T.Y.; Fouchier,R.A.; Yasa,I.W.W.; Osterhaus,A.D.M.E.; Fouchier,R.A.M.
EPI533436	HA	Indonesia	2009-Jan-01	A/chicken/West Java/X3/2009	Dr. Teguh Y. Prajitno	Erasmus Medical Center	Koel,B.F.; van der Vliet,S.; Burke,D.F.; Bestebroer,T.M.; Bharoto,E.E.; Yasa,I.W.; Herliana,I.; Laksono,B.M.; Xu,K.; Skepner,E.; Russell,C.A.; Rimmelzwaan,G.F.; Perez,D.R.; Osterhaus,A.D.; Smith,D.J.; Prajitno,T.Y.; Fouchier,R.A.; Yasa,I.W.W.; Osterhaus,A.D.M.E.; Fouchier,R.A.M.
EPI533435	HA	Indonesia	2009-Jan-01	A/chicken/West Java/2/2009	Dr. Teguh Y. Prajitno	Erasmus Medical Center	Koel,B.F.; van der Vliet,S.; Burke,D.F.; Bestebroer,T.M.; Bharoto,E.E.; Yasa,I.W.; Herliana,I.; Laksono,B.M.; Xu,K.; Skepner,E.; Russell,C.A.; Rimmelzwaan,G.F.; Perez,D.R.; Osterhaus,A.D.; Smith,D.J.; Prajitno,T.Y.; Fouchier,R.A.; Yasa,I.W.W.; Osterhaus,A.D.M.E.; Fouchier,R.A.M.
EPI533430	HA	Indonesia	2010-Jan-01	A/chicken/West Java/145/2010	Dr. Teguh Y. Prajitno	Erasmus Medical Center	Koel,B.F.; van der Vliet,S.; Burke,D.F.; Bestebroer,T.M.; Bharoto,E.E.; Yasa,I.W.; Herliana,I.; Laksono,B.M.; Xu,K.; Skepner,E.; Russell,C.A.; Rimmelzwaan,G.F.; Perez,D.R.; Osterhaus,A.D.; Smith,D.J.; Prajitno,T.Y.; Fouchier,R.A.; Yasa,I.W.W.; Osterhaus,A.D.M.E.; Fouchier,R.A.M.
EPI533429	HA	Indonesia	2011-Jan-01	A/chicken/West Java/145/2011	Dr. Teguh Y. Prajitno	Erasmus Medical Center	Koel,B.F.; van der Vliet,S.; Burke,D.F.; Bestebroer,T.M.; Bharoto,E.E.; Yasa,I.W.; Herliana,I.; Laksono,B.M.; Xu,K.; Skepner,E.; Russell,C.A.; Rimmelzwaan,G.F.; Perez,D.R.; Osterhaus,A.D.; Smith,D.J.; Prajitno,T.Y.; Fouchier,R.A.; Yasa,I.W.W.; Osterhaus,A.D.M.E.; Fouchier,R.A.M.
EPI533428	HA	Indonesia	2010-Jan-01	A/chicken/West Java/X4/2010	Dr. Teguh Y. Prajitno	Erasmus Medical Center	Koel,B.F.; van der Vliet,S.; Burke,D.F.; Bestebroer,T.M.; Bharoto,E.E.; Yasa,I.W.; Herliana,I.; Laksono,B.M.; Xu,K.; Skepner,E.; Russell,C.A.; Rimmelzwaan,G.F.; Perez,D.R.; Osterhaus,A.D.; Smith,D.J.; Prajitno,T.Y.; Fouchier,R.A.; Yasa,I.W.W.; Osterhaus,A.D.M.E.; Fouchier,R.A.M.

EPI533424	HA	Indonesia	2009-Jan-01	A/chicken/West Java/47/2009	Dr. Teguh Y. Prajitno	Erasmus Medical Center	Koel,B.F.; van der Vliet,S.; Burke,D.F.; Bestebroer,T.M.; Bharoto,E.E.; Yasa,I.W.; Herliana,I.; Laksono,B.M.; Xu,K.; Skepner,E.; Russell,C.A.; Rimmelzwaan,G.F.; Perez,D.R.; Osterhaus,A.D.; Smith,D.J.; Prajitno,T.Y.; Fouchier,R.A.; Yasa,I.W.W.; Osterhaus,A.D.M.E.; Fouchier,R.A.M.
EPI533423	HA	Indonesia	2009-Jan-01	A/chicken/West Java/049/2009	Dr. Teguh Y. Prajitno	Erasmus Medical Center	Koel,B.F.; van der Vliet,S.; Burke,D.F.; Bestebroer,T.M.; Bharoto,E.E.; Yasa,I.W.; Herliana,I.; Laksono,B.M.; Xu,K.; Skepner,E.; Russell,C.A.; Rimmelzwaan,G.F.; Perez,D.R.; Osterhaus,A.D.; Smith,D.J.; Prajitno,T.Y.; Fouchier,R.A.; Yasa,I.W.W.; Osterhaus,A.D.M.E.; Fouchier,R.A.M.
EPI533417	HA	Indonesia	2010-Jan-01	A/chicken/West Java/125A/2010	Dr. Teguh Y. Prajitno	Erasmus Medical Center	Koel,B.F.; van der Vliet,S.; Burke,D.F.; Bestebroer,T.M.; Bharoto,E.E.; Yasa,I.W.; Herliana,I.; Laksono,B.M.; Xu,K.; Skepner,E.; Russell,C.A.; Rimmelzwaan,G.F.; Perez,D.R.; Osterhaus,A.D.; Smith,D.J.; Prajitno,T.Y.; Fouchier,R.A.; Yasa,I.W.W.; Osterhaus,A.D.M.E.; Fouchier,R.A.M.
EPI533416	HA	Indonesia	2010-Jan-01	A/chicken/West Java/131/2010	Dr. Teguh Y. Prajitno	Erasmus Medical Center	Koel,B.F.; van der Vliet,S.; Burke,D.F.; Bestebroer,T.M.; Bharoto,E.E.; Yasa,I.W.; Herliana,I.; Laksono,B.M.; Xu,K.; Skepner,E.; Russell,C.A.; Rimmelzwaan,G.F.; Perez,D.R.; Osterhaus,A.D.; Smith,D.J.; Prajitno,T.Y.; Fouchier,R.A.; Yasa,I.W.W.; Osterhaus,A.D.M.E.; Fouchier,R.A.M.
EPI533415	HA	Indonesia	2010-Jan-01	A/chicken/West Java/138/2010	Dr. Teguh Y. Prajitno	Erasmus Medical Center	Koel,B.F.; van der Vliet,S.; Burke,D.F.; Bestebroer,T.M.; Bharoto,E.E.; Yasa,I.W.; Herliana,I.; Laksono,B.M.; Xu,K.; Skepner,E.; Russell,C.A.; Rimmelzwaan,G.F.; Perez,D.R.; Osterhaus,A.D.; Smith,D.J.; Prajitno,T.Y.; Fouchier,R.A.; Yasa,I.W.W.; Osterhaus,A.D.M.E.; Fouchier,R.A.M.
EPI533413	HA	Indonesia	2009-Jan-01	A/chicken/West Java/20/2009	Dr. Teguh Y. Prajitno	Erasmus Medical Center	Koel,B.F.; van der Vliet,S.; Burke,D.F.; Bestebroer,T.M.; Bharoto,E.E.; Yasa,I.W.; Herliana,I.; Laksono,B.M.; Xu,K.; Skepner,E.; Russell,C.A.; Rimmelzwaan,G.F.; Perez,D.R.; Osterhaus,A.D.; Smith,D.J.; Prajitno,T.Y.; Fouchier,R.A.; Yasa,I.W.W.; Osterhaus,A.D.M.E.; Fouchier,R.A.M.
EPI533410	HA	Indonesia	2009-Jan-01	A/chicken/West Java/034/2009	Dr. Teguh Y. Prajitno	Erasmus Medical Center	Koel,B.F.; van der Vliet,S.; Burke,D.F.; Bestebroer,T.M.; Bharoto,E.E.; Yasa,I.W.; Herliana,I.; Laksono,B.M.; Xu,K.; Skepner,E.; Russell,C.A.; Rimmelzwaan,G.F.; Perez,D.R.; Osterhaus,A.D.; Smith,D.J.; Prajitno,T.Y.; Fouchier,R.A.; Yasa,I.W.W.; Osterhaus,A.D.M.E.; Fouchier,R.A.M.
EPI533409	HA	Indonesia	2010-Jan-01	A/chicken/West Java/084/2010	Dr. Teguh Y. Prajitno	Erasmus Medical Center	Koel,B.F.; van der Vliet,S.; Burke,D.F.; Bestebroer,T.M.; Bharoto,E.E.; Yasa,I.W.; Herliana,I.; Laksono,B.M.; Xu,K.; Skepner,E.; Russell,C.A.; Rimmelzwaan,G.F.; Perez,D.R.; Osterhaus,A.D.; Smith,D.J.; Prajitno,T.Y.; Fouchier,R.A.; Yasa,I.W.W.; Osterhaus,A.D.M.E.; Fouchier,R.A.M.
EPI533408	HA	Indonesia	2010-Jan-01	A/chicken/West Java/84/2010	Dr. Teguh Y. Prajitno	Erasmus Medical Center	Koel,B.F.; van der Vliet,S.; Burke,D.F.; Bestebroer,T.M.; Bharoto,E.E.; Yasa,I.W.; Herliana,I.; Laksono,B.M.; Xu,K.; Skepner,E.; Russell,C.A.; Rimmelzwaan,G.F.; Perez,D.R.; Osterhaus,A.D.; Smith,D.J.; Prajitno,T.Y.; Fouchier,R.A.; Yasa,I.W.W.; Osterhaus,A.D.M.E.; Fouchier,R.A.M.
EPI533407	HA	Indonesia	2009-Jan-01	A/chicken/West Java/015/2009	Dr. Teguh Y. Prajitno	Erasmus Medical Center	Koel,B.F.; van der Vliet,S.; Burke,D.F.; Bestebroer,T.M.; Bharoto,E.E.; Yasa,I.W.; Herliana,I.; Laksono,B.M.; Xu,K.; Skepner,E.; Russell,C.A.; Rimmelzwaan,G.F.; Perez,D.R.; Osterhaus,A.D.; Smith,D.J.; Prajitno,T.Y.; Fouchier,R.A.; Yasa,I.W.W.; Osterhaus,A.D.M.E.; Fouchier,R.A.M.
EPI533405	HA	Indonesia	2010-Jan-01	A/chicken/West Java/081/2010	Dr. Teguh Y. Prajitno	Erasmus Medical Center	Koel,B.F.; van der Vliet,S.; Burke,D.F.; Bestebroer,T.M.; Bharoto,E.E.; Yasa,I.W.; Herliana,I.; Laksono,B.M.; Xu,K.; Skepner,E.; Russell,C.A.; Rimmelzwaan,G.F.; Perez,D.R.; Osterhaus,A.D.; Smith,D.J.; Prajitno,T.Y.; Fouchier,R.A.; Yasa,I.W.W.; Osterhaus,A.D.M.E.; Fouchier,R.A.M.
EPI533404	HA	Indonesia	2010-Jan-01	A/chicken/West Java/090/2010	Dr. Teguh Y. Prajitno	Erasmus Medical Center	Koel,B.F.; van der Vliet,S.; Burke,D.F.; Bestebroer,T.M.; Bharoto,E.E.; Yasa,I.W.; Herliana,I.; Laksono,B.M.; Xu,K.; Skepner,E.; Russell,C.A.; Rimmelzwaan,G.F.; Perez,D.R.; Osterhaus,A.D.; Smith,D.J.; Prajitno,T.Y.; Fouchier,R.A.; Yasa,I.W.W.; Osterhaus,A.D.M.E.; Fouchier,R.A.M.
EPI533403	HA	Indonesia	2010-Jan-01	A/chicken/West Java/90/2010	Dr. Teguh Y. Prajitno	Erasmus Medical Center	Koel,B.F.; van der Vliet,S.; Burke,D.F.; Bestebroer,T.M.; Bharoto,E.E.; Yasa,I.W.; Herliana,I.; Laksono,B.M.; Xu,K.; Skepner,E.; Russell,C.A.; Rimmelzwaan,G.F.; Perez,D.R.; Osterhaus,A.D.; Smith,D.J.; Prajitno,T.Y.; Fouchier,R.A.; Yasa,I.W.W.; Osterhaus,A.D.M.E.; Fouchier,R.A.M.
EPI533402	HA	Indonesia	2010-Jan-01	A/chicken/West Java/097/2010	Dr. Teguh Y. Prajitno	Erasmus Medical Center	Koel,B.F.; van der Vliet,S.; Burke,D.F.; Bestebroer,T.M.; Bharoto,E.E.; Yasa,I.W.; Herliana,I.; Laksono,B.M.; Xu,K.; Skepner,E.; Russell,C.A.; Rimmelzwaan,G.F.; Perez,D.R.; Osterhaus,A.D.; Smith,D.J.; Prajitno,T.Y.; Fouchier,R.A.; Yasa,I.W.W.; Osterhaus,A.D.M.E.; Fouchier,R.A.M.
EPI533401	HA	Indonesia	2010-Jan-01	A/chicken/West Java/97/2010	Dr. Teguh Y. Prajitno	Erasmus Medical Center	Koel,B.F.; van der Vliet,S.; Burke,D.F.; Bestebroer,T.M.; Bharoto,E.E.; Yasa,I.W.; Herliana,I.; Laksono,B.M.; Xu,K.; Skepner,E.; Russell,C.A.; Rimmelzwaan,G.F.; Perez,D.R.; Osterhaus,A.D.; Smith,D.J.; Prajitno,T.Y.; Fouchier,R.A.; Yasa,I.W.W.; Osterhaus,A.D.M.E.; Fouchier,R.A.M.
EPI533400	HA	Indonesia	2003-Jan-01	A/chicken/West Java/A/2003	Dr. Teguh Y. Prajitno	Erasmus Medical Center	Koel,B.F.; van der Vliet,S.; Burke,D.F.; Bestebroer,T.M.; Bharoto,E.E.; Yasa,I.W.; Herliana,I.; Laksono,B.M.; Xu,K.; Skepner,E.; Russell,C.A.; Rimmelzwaan,G.F.; Perez,D.R.; Osterhaus,A.D.; Smith,D.J.; Prajitno,T.Y.; Fouchier,R.A.; Yasa,I.W.W.; Osterhaus,A.D.M.E.; Fouchier,R.A.M.
EPI533399	HA	Indonesia	2009-Jan-01	A/chicken/West Java/127/2009	Dr. Teguh Y. Prajitno	Erasmus Medical Center	Koel,B.F.; van der Vliet,S.; Burke,D.F.; Bestebroer,T.M.; Bharoto,E.E.; Yasa,I.W.; Herliana,I.; Laksono,B.M.; Xu,K.; Skepner,E.; Russell,C.A.; Rimmelzwaan,G.F.; Perez,D.R.; Osterhaus,A.D.; Smith,D.J.; Prajitno,T.Y.; Fouchier,R.A.; Yasa,I.W.W.; Osterhaus,A.D.M.E.; Fouchier,R.A.M.
EPI533398	HA	Indonesia	2010-Jan-01	A/chicken/West Java/142/2010	Dr. Teguh Y. Prajitno	Erasmus Medical Center	Koel,B.F.; van der Vliet,S.; Burke,D.F.; Bestebroer,T.M.; Bharoto,E.E.; Yasa,I.W.; Herliana,I.; Laksono,B.M.; Xu,K.; Skepner,E.; Russell,C.A.; Rimmelzwaan,G.F.; Perez,D.R.; Osterhaus,A.D.; Smith,D.J.; Prajitno,T.Y.; Fouchier,R.A.; Yasa,I.W.W.; Osterhaus,A.D.M.E.; Fouchier,R.A.M.
EPI533396	HA	Indonesia	2009-Jan-01	A/chicken/Central Java/59/2009	Dr. Teguh Y. Prajitno	Erasmus Medical Center	Koel,B.F.; van der Vliet,S.; Burke,D.F.; Bestebroer,T.M.; Bharoto,E.E.; Yasa,I.W.; Herliana,I.; Laksono,B.M.; Xu,K.; Skepner,E.; Russell,C.A.; Rimmelzwaan,G.F.; Perez,D.R.; Osterhaus,A.D.; Smith,D.J.; Prajitno,T.Y.; Fouchier,R.A.; Yasa,I.W.W.; Osterhaus,A.D.M.E.; Fouchier,R.A.M.
EPI533395	HA	Indonesia	2009-Jan-01	A/chicken/Central Java/52/2009	Dr. Teguh Y. Prajitno	Erasmus Medical Center	Koel,B.F.; van der Vliet,S.; Burke,D.F.; Bestebroer,T.M.; Bharoto,E.E.; Yasa,I.W.; Herliana,I.; Laksono,B.M.; Xu,K.; Skepner,E.; Russell,C.A.; Rimmelzwaan,G.F.; Perez,D.R.; Osterhaus,A.D.; Smith,D.J.; Prajitno,T.Y.; Fouchier,R.A.; Yasa,I.W.W.; Osterhaus,A.D.M.E.; Fouchier,R.A.M.
EPI533394	HA	Indonesia	2009-Jan-01	A/chicken/Central Java/053/2009	Dr. Teguh Y. Prajitno	Erasmus Medical Center	Koel,B.F.; van der Vliet,S.; Burke,D.F.; Bestebroer,T.M.; Bharoto,E.E.; Yasa,I.W.; Herliana,I.; Laksono,B.M.; Xu,K.; Skepner,E.; Russell,C.A.; Rimmelzwaan,G.F.; Perez,D.R.; Osterhaus,A.D.; Smith,D.J.; Prajitno,T.Y.; Fouchier,R.A.; Yasa,I.W.W.; Osterhaus,A.D.M.E.; Fouchier,R.A.M.
EPI533393	HA	Indonesia	2010-Jan-01	A/chicken/Central Java/123/2010	Dr. Teguh Y. Prajitno	Erasmus Medical Center	Koel,B.F.; van der Vliet,S.; Burke,D.F.; Bestebroer,T.M.; Bharoto,E.E.; Yasa,I.W.; Herliana,I.; Laksono,B.M.; Xu,K.; Skepner,E.; Russell,C.A.; Rimmelzwaan,G.F.; Perez,D.R.; Osterhaus,A.D.; Smith,D.J.; Prajitno,T.Y.; Fouchier,R.A.; Yasa,I.W.W.; Osterhaus,A.D.M.E.; Fouchier,R.A.M.
EPI533392	HA	Indonesia	2010-Jan-01	A/chicken/Central Java/139/2010	Dr. Teguh Y. Prajitno	Erasmus Medical Center	Koel,B.F.; van der Vliet,S.; Burke,D.F.; Bestebroer,T.M.; Bharoto,E.E.; Yasa,I.W.; Herliana,I.; Laksono,B.M.; Xu,K.; Skepner,E.; Russell,C.A.; Rimmelzwaan,G.F.; Perez,D.R.; Osterhaus,A.D.; Smith,D.J.; Prajitno,T.Y.; Fouchier,R.A.; Yasa,I.W.W.; Osterhaus,A.D.M.E.; Fouchier,R.A.M.
EPI533388	HA	Indonesia	2010-Jan-01	A/chicken/Central Java/099/2010	Dr. Teguh Y. Prajitno	Erasmus Medical Center	Koel,B.F.; van der Vliet,S.; Burke,D.F.; Bestebroer,T.M.; Bharoto,E.E.; Yasa,I.W.; Herliana,I.; Laksono,B.M.; Xu,K.; Skepner,E.; Russell,C.A.; Rimmelzwaan,G.F.; Perez,D.R.; Osterhaus,A.D.; Smith,D.J.; Prajitno,T.Y.; Fouchier,R.A.; Yasa,I.W.W.; Osterhaus,A.D.M.E.; Fouchier,R.A.M.
EPI533387	HA	Indonesia	2009-Jan-01	A/chicken/Central Java/056/2009	Dr. Teguh Y. Prajitno	Erasmus Medical Center	Koel,B.F.; van der Vliet,S.; Burke,D.F.; Bestebroer,T.M.; Bharoto,E.E.; Yasa,I.W.; Herliana,I.; Laksono,B.M.; Xu,K.; Skepner,E.; Russell,C.A.; Rimmelzwaan,G.F.; Perez,D.R.; Osterhaus,A.D.; Smith,D.J.; Prajitno,T.Y.; Fouchier,R.A.; Yasa,I.W.W.; Osterhaus,A.D.M.E.; Fouchier,R.A.M.
EPI533386	HA	Indonesia	2011-Jan-01	A/chicken/Central Java/146/2011	Dr. Teguh Y. Prajitno	Erasmus Medical Center	Koel,B.F.; van der Vliet,S.; Burke,D.F.; Bestebroer,T.M.; Bharoto,E.E.; Yasa,I.W.; Herliana,I.; Laksono,B.M.; Xu,K.; Skepner,E.; Russell,C.A.; Rimmelzwaan,G.F.; Perez,D.R.; Osterhaus,A.D.; Smith,D.J.; Prajitno,T.Y.; Fouchier,R.A.; Yasa,I.W.W.; Osterhaus,A.D.M.E.; Fouchier,R.A.M.
EPI533381	HA	Indonesia	2009-Jan-01	A/chicken/East Java/013/2009	Dr. Teguh Y. Prajitno	Erasmus Medical Center	Koel,B.F.; van der Vliet,S.; Burke,D.F.; Bestebroer,T.M.; Bharoto,E.E.; Yasa,I.W.; Herliana,I.; Laksono,B.M.; Xu,K.; Skepner,E.; Russell,C.A.; Rimmelzwaan,G.F.; Perez,D.R.; Osterhaus,A.D.; Smith,D.J.; Prajitno,T.Y.; Fouchier,R.A.; Yasa,I.W.W.; Osterhaus,A.D.M.E.; Fouchier,R.A.M.
EPI533380	HA	Indonesia	2009-Jan-01	A/chicken/East Java/6/2009	Dr. Teguh Y. Prajitno	Erasmus Medical Center	Koel,B.F.; van der Vliet,S.; Burke,D.F.; Bestebroer,T.M.; Bharoto,E.E.; Yasa,I.W.; Herliana,I.; Laksono,B.M.; Xu,K.; Skepner,E.; Russell,C.A.; Rimmelzwaan,G.F.; Perez,D.R.; Osterhaus,A.D.; Smith,D.J.; Prajitno,T.Y.; Fouchier,R.A.; Yasa,I.W.W.; Osterhaus,A.D.M.E.; Fouchier,R.A.M.

EPI533379	HA	Indonesia	2009-Jan-01	A/chicken/East Java/035/2009	Dr. Teguh Y. Prajitno	Erasmus Medical Center	Koel,B.F.; van der Vliet,S.; Burke,D.F.; Bestebroer,T.M.; Bharoto,E.E.; Yasa,I.W.; Herliana,I.; Laksono,B.M.; Xu,K.; Skepner,E.; Russell,C.A.; Rimmelzwaan,G.F.; Perez,D.R.; Osterhaus,A.D.; Smith,D.J.; Prajitno,T.Y.; Fouchier,R.A.; Yasa,I.W.W.; Osterhaus,A.D.M.E.; Fouchier,R.A.M.
EPI533378	HA	Indonesia	2009-Jan-01	A/chicken/South Kalimantan/070/2009	Dr. Teguh Y. Prajitno	Erasmus Medical Center	Koel,B.F.; van der Vliet,S.; Burke,D.F.; Bestebroer,T.M.; Bharoto,E.E.; Yasa,I.W.; Herliana,I.; Laksono,B.M.; Xu,K.; Skepner,E.; Russell,C.A.; Rimmelzwaan,G.F.; Perez,D.R.; Osterhaus,A.D.; Smith,D.J.; Prajitno,T.Y.; Fouchier,R.A.; Yasa,I.W.W.; Osterhaus,A.D.M.E.; Fouchier,R.A.M.
EPI533376	HA	Indonesia	2011-Jan-01	A/chicken/South Sulawesi/156/2011	Dr. Teguh Y. Prajitno	Erasmus Medical Center	Koel,B.F.; van der Vliet,S.; Burke,D.F.; Bestebroer,T.M.; Bharoto,E.E.; Yasa,I.W.; Herliana,I.; Laksono,B.M.; Xu,K.; Skepner,E.; Russell,C.A.; Rimmelzwaan,G.F.; Perez,D.R.; Osterhaus,A.D.; Smith,D.J.; Prajitno,T.Y.; Fouchier,R.A.; Yasa,I.W.W.; Osterhaus,A.D.M.E.; Fouchier,R.A.M.
EPI533373	HA	Indonesia	2011-Jan-01	A/chicken/South Sulawesi/195/2011	Dr. Teguh Y. Prajitno	Erasmus Medical Center	Koel,B.F.; van der Vliet,S.; Burke,D.F.; Bestebroer,T.M.; Bharoto,E.E.; Yasa,I.W.; Herliana,I.; Laksono,B.M.; Xu,K.; Skepner,E.; Russell,C.A.; Rimmelzwaan,G.F.; Perez,D.R.; Osterhaus,A.D.; Smith,D.J.; Prajitno,T.Y.; Fouchier,R.A.; Yasa,I.W.W.; Osterhaus,A.D.M.E.; Fouchier,R.A.M.
EPI533372	HA	Indonesia	2011-Jan-01	A/chicken/South Sulawesi/196/2011	Dr. Teguh Y. Prajitno	Erasmus Medical Center	Koel,B.F.; van der Vliet,S.; Burke,D.F.; Bestebroer,T.M.; Bharoto,E.E.; Yasa,I.W.; Herliana,I.; Laksono,B.M.; Xu,K.; Skepner,E.; Russell,C.A.; Rimmelzwaan,G.F.; Perez,D.R.; Osterhaus,A.D.; Smith,D.J.; Prajitno,T.Y.; Fouchier,R.A.; Yasa,I.W.W.; Osterhaus,A.D.M.E.; Fouchier,R.A.M.
EPI533370	HA	Indonesia	2003-Jan-01	A/chicken/Indonesia/BL/2003	Dr. Teguh Y. Prajitno	Erasmus Medical Center	Koel,B.F.; van der Vliet,S.; Burke,D.F.; Bestebroer,T.M.; Bharoto,E.E.; Yasa,I.W.; Herliana,I.; Laksono,B.M.; Xu,K.; Skepner,E.; Russell,C.A.; Rimmelzwaan,G.F.; Perez,D.R.; Osterhaus,A.D.; Smith,D.J.; Prajitno,T.Y.; Fouchier,R.A.; Yasa,I.W.W.; Osterhaus,A.D.M.E.; Fouchier,R.A.M.
EPI533369	HA	Indonesia	2003-Jan-01	A/chicken/Indonesia/PA/2003	Dr. Teguh Y. Prajitno	Erasmus Medical Center	Koel,B.F.; van der Vliet,S.; Burke,D.F.; Bestebroer,T.M.; Bharoto,E.E.; Yasa,I.W.; Herliana,I.; Laksono,B.M.; Xu,K.; Skepner,E.; Russell,C.A.; Rimmelzwaan,G.F.; Perez,D.R.; Osterhaus,A.D.; Smith,D.J.; Prajitno,T.Y.; Fouchier,R.A.; Yasa,I.W.W.; Osterhaus,A.D.M.E.; Fouchier,R.A.M.
EPI517161	HA	Japan	2014-Apr-13	A/Chicken/Kumamoto/1-7/2014	National Institute of Animal Health	National Agriculture and Food Research Organization	Kanehira,K.; Takemae,N.; Uchida,Y.; Tunekuni,R.; Hikono,H.; Saito,T.
EPI498342	HA	Myanmar	2010-Mar-04	A/chicken/Yinmarbin/A295/2010	National Veterinary Diagnostic Laboratory, Ministry of Livestock and Fisheries	CSIRO Australian Animal Health Laboratory	MaungMaung,Kyin; Vicky,Stevens; Kelly,Davies; Songhua,Shan; Daglas,Susie; Kerri,Bruce; Paul,Selleck; Frank,Wong; Chris,Morrissy
EPI632943	HA	Cote d'Ivoire	2015-Apr-13	A/duck/Ivory Coast/15VIR-2742-1/2015		Istituto Zooprofilattico Sperimentale Delle Venezie	Diarra,Cisse-Aman; Emmanuel,Coucy-Hymann; Alice,Fusaro; Luca,Tassoni; Alessia,Schivo; Silvia,Ormelli; Giovanni,Cattoli; Isabella,Monne
EPI588976	HA	Taiwan	2015-Jan-13	A/duck/Taiwan/a068/2015	ANIMAL HEALTH RESEARCH INSTITUTE	Animal Health Research Institute	Yu-Ju,Lin; Li-Hsuan,Chen; Wan-Chen,Li; Yu-Pin,Liu; Ming-Shiuh,Lee; Ming-Chu,Cheng; Hsiang-Jung,Tsai
EPI588960	HA	Taiwan	2015-Jan-13	A/duck/Taiwan/a043/2015	ANIMAL HEALTH RESEARCH INSTITUTE	Animal Health Research Institute	Yu-Ju,Lin; Li-Hsuan,Chen; Wan-Chen,Li; Yu-Pin,Liu; Ming-Shiuh,Lee; Ming-Chu,Cheng; Hsiang-Jung,Tsai
EPI579781	HA	Egypt	2015-Jan-18	A/duck/Egypt/CLEVB-25_N00239/2015	The Central Laboratory for Evaluation of Veterinary Biologics (CLEVB)	NAMRU-3	Younan, M.; Defang, G.; Mohareb, E.; Ali, A.M.; Nassif, S.A.; Mourad,A.A.; Fouad, E.M.; Ragab, A.S.; Khelfa, D.G.
EPI579780	HA	Egypt	2015-Jan-18	A/duck/Egypt/CLEVB-24_N00238/2015	The Central Laboratory for Evaluation of Veterinary Biologics (CLEVB)	NAMRU-3	Younan, M.; Defang, G.; Mohareb, E.; Ali, A.M.; Nassif, S.A.; Mourad,A.A.; Fouad, E.M.; Ragab, A.S.; Khelfa, D.G.
EPI573332	HA	Egypt	2014-Jun-25	A/duck/Egypt/14154FAOS/2014		Friedrich-Loeffler-Institut	Arafa,A.M; Hagag, N; Elhusseiny, M.H; Yehia, N; Selim, A.A; Abdelhalim, A; Kilany, W.H; Samy, A; Hassan, M.K; Abdelwhab,E.M; Beer,M; Naguib, M.M; Harder,TC
EPI573324	HA	Egypt	2015-Jan-18	A/duck/Egypt/1560S/2015		Friedrich-Loeffler-Institut	Arafa,A.M; Hagag, N; Elhusseiny, M.H; Yehia, N; Selim, A.A; Abdelhalim, A; Kilany, W.H; Samy, A; Hassan, M.K; Abdelwhab,E.M; Beer,M; Naguib, M.M; Harder,TC
EPI573323	HA	Egypt	2014-Dec-04	A/duck/Egypt/144/2014		Friedrich-Loeffler-Institut	Arafa,A.M; Hagag, N; Elhusseiny, M.H; Yehia, N; Selim, A.A; Abdelhalim, A; Kilany, W.H; Samy, A; Hassan, M.K; Abdelwhab,E.M; Beer,M; Naguib, M.M; Harder,TC
EPI573315	HA	Egypt	2014-Dec-14	A/duck/Egypt/BS-146RS-f6/2014		Friedrich-Loeffler-Institut	Arafa,A.M; Hagag, N; Elhusseiny, M.H; Yehia, N; Selim, A.A; Abdelhalim, A; Kilany, W.H; Samy, A; Hassan, M.K; Abdelwhab,E.M; Beer,M; Naguib, M.M; Harder,TC
EPI573260	HA	Egypt	2015-Jan-15	A/Duck/Egypt/AR236-A3NLQP/2015		Friedrich-Loeffler-Institut	Naguib, M.M; Arafa,A.M; Luttermann,C; Selim,A.A; Hassan,M.K; Beer,M;Harder,TC
EPI573246	HA	Egypt	2014-Dec-22	A/Duck/Egypt/AR232-A13NLQP/2014		Friedrich-Loeffler-Institut	Naguib, M.M; Arafa,A.M; Luttermann,C; Selim,A.A; Hassan,M.K; Beer,M;Harder,TC
EPI573242	HA	Korea, Republic of	2014-Mar-04	A/Common Teal/Korea/H844/2014		Animal and Plant Quarantine Agency	
EPI573234	HA	Korea, Republic of	2014-Nov-15	A/broiler duck/Korea/H1864/2014		Animal and Plant Quarantine Agency	
EPI573232	HA	Korea, Republic of	2014-Nov-07	A/broiler duck/Korea/H1840/2014		Animal and Plant Quarantine Agency	
EPI573231	HA	Korea, Republic of	2014-Nov-07	A/broiler duck/Korea/H1839/2014		Animal and Plant Quarantine Agency	
EPI573228	HA	Korea, Republic of	2014-Oct-05	A/broiler duck/Korea/H1755/2014		Animal and Plant Quarantine Agency	
EPI573227	HA	Korea, Republic of	2014-Oct-02	A/breeder duck/Korea/H1752/2014		Animal and Plant Quarantine Agency	
EPI573225	HA	Korea, Republic of	2014-Sep-30	A/broiler duck/Korea/H1745/2014		Animal and Plant Quarantine Agency	
EPI573223	HA	Korea, Republic of	2014-Sep-25	A/broiler duck/Korea/H1734/2014		Animal and Plant Quarantine Agency	
EPI573221	HA	Korea, Republic of	2014-Sep-24	A/broiler duck/Korea/H1731/2014		Animal and Plant Quarantine Agency	
EPI573215	HA	Korea, Republic of	2014-Jun-26	A/breeder duck/Korea/H1596/2014		Animal and Plant Quarantine Agency	
EPI573214	HA	Korea, Republic of	2014-Jun-25	A/broiler duck/Korea/H1582/2014		Animal and Plant Quarantine Agency	
EPI573209	HA	Korea, Republic of	2014-May-20	A/broiler duck/Korea/H1413/2014		Animal and Plant Quarantine Agency	
EPI573206	HA	Korea, Republic of	2014-May-04	A/breeder duck/Korea/H1343/2014		Animal and Plant Quarantine Agency	
EPI573198	HA	Korea, Republic of	2014-Mar-10	A/broiler duck/Korea/H959/2014		Animal and Plant Quarantine Agency	
EPI557194	HA	Egypt	2013-Feb-21	A/Duck/Egypt/NLQP27SG-AR750/2013	National Laboratory for Veterinary Quality Control on Poultry production- Animal Health Research Institute	Friedrich-Loeffler-Institut	Naguib, M.M; Arafa, A.M; Selim,A.A; Hassan,M.K; Beer,M; Harder,TC
EPI553316	HA	China	2009-Sep-01	A/Duck/Shandong/Y02/2009	Qingdao Agricultural University	Beijing Institute of Microbiology and Epidemiology	yanbo,yin;dongdong,wang;linlin,liu;xiliang,wang;penghui,yang;xin,liu;keyu,wang;chengcai,lai
EPI550849	HA	United Kingdom	2014-Nov-14	A/duck/England/36226/14	Animal and Plant Health Agency (APHA)	Animal and Plant Health Agency (APHA)	Hanna, Amanda; Ellis, Richard; Ceeraz, Vanessa; Seekings, James; Londt, Brandon; Brookes, Sharon; Banks, Jill; Essen, Stephen; Brown, Ian
EPI550848	HA	United Kingdom	2014-Nov-14	A/duck/England/36038/14	Animal and Plant Health Agency (APHA)	Animal and Plant Health Agency (APHA)	Hanna, Amanda; Ellis, Richard; Ceeraz, Vanessa; Seekings, James; Londt, Brandon; Brookes, Sharon; Banks, Jill; Essen, Stephen; Brown, Ian
EPI543010	HA	China	2014-Jan-22	A/duck/Beijing/CT01/2014	Institute of Microbiology, Chinese Academy of Sciences	Institute of Microbiology	
EPI542617	HA	China	2013-Nov-10	A/duck/Beijing/FS01/2013	Institute of Microbiology, Chinese Academy of Sciences	Institute of Microbiology	
EPI538783	HA	Egypt	2013-May-01	A/duck/Egypt/13VIR-2962-196/2013	Istituto Zooprofilattico Sperimentale Delle Venezie	Istituto Zooprofilattico Sperimentale Delle Venezie	Hussein, H.A.; El Hady, M.M.; Abd Hamid, H.S.; Sultan, H.A.; Abdel Hafez, A.; Fusaro, A.; Schivo, A.; Ormelli, S.; Monne, I.; Cattoli, G.
EPI538775	HA	Egypt	2013-May-01	A/duck/Egypt/13VIR-2962-195/2013	Istituto Zooprofilattico Sperimentale Delle Venezie	Istituto Zooprofilattico Sperimentale Delle Venezie	Hussein, H.A.; El Hady, M.M.; Abd Hamid, H.S.; Sultan, H.A.; Abdel Hafez, A.; Fusaro, A.; Schivo, A.; Ormelli, S.; Monne, I.; Cattoli, G.
EPI485596	HA	Cambodia	2013-Feb-12	A/duck/Cambodia/261W7M2/2013	Institut Pasteur in Cambodia	Institut Pasteur in Cambodia	Rith, S.; Horm, SV.; Buchy, P.
EPI485592	HA	Cambodia	2013-Jan-09	A/duck/Cambodia/46W2M4/2013	Institut Pasteur in Cambodia	Institut Pasteur in Cambodia	Rith, S.; Horm, SV.; Buchy, P.
EPI431456	HA	China	2011-Dec-07	A/duck/Hebei/3/2011	Institute of Microbiology, Chinese Academy of Sciences	Institute of Microbiology	Di,Liu; Haigang,Sun; Jinghua,Yan; George F,Gao; Juncai,Ma
EPI431448	HA	China	2011-Dec-01	A/duck/Hebei/2/2011	Institute of Microbiology, Chinese Academy of Sciences	Institute of Microbiology	Di,Liu; Haigang,Sun; Jinghua,Yan; George F,Gao; Juncai,Ma
EPI376533	HA		2008-Jan-01	A/duck/Egypt/1709-10/2008		Istituto Zooprofilattico Sperimentale Delle Venezie	
EPI356863	HA	Nepal	2011-Nov-07	A/duck/Nepal/5.TZoo/11	Central Veterinary Laboratory	Animal and Plant Health Agency (APHA)	Puranik, A; Hanna, A; Essen, S; Focosi-Snyman, R; Manvell, R.J; Sedai, D; Chapagain, S; Manandhar, S; Koirala, P; Karki, K.B; Pandey, K.R; Air, T.B; Reid, S

EPI356413	HA	Germany	2009-Jan-01	A/wild duck/Germany-BY/R2892/2009		Friedrich-Loeffler-Institut	
EPI354764	HA	Bulgaria	2011-Oct-22	A/duck/Bulgaria/Shishmanzi-26/11	NDRVMI (National Diagnostic and Research Veterinary Medical Institute)	Animal and Plant Health Agency (APHA)	Puranik, A; Hanna, A; Essen, S; Focosi-Snyman, R; Manvell, R; Marinova-Petkova, A; Georgiev, G; Webby, R.J; Webster, R.G; Reid, S
EPI354763	HA	Bulgaria	2011-Oct-22	A/duck/Bulgaria/Shishmanzi-25/11	NDRVMI (National Diagnostic and Research Veterinary Medical Institute)	Animal and Plant Health Agency (APHA)	Puranik, A; Hanna, A; Essen, S; Focosi-Snyman, R; Manvell, R; Marinova-Petkova, A; Georgiev, G; Webby, R.J; Webster, R.G; Reid, S
EPI354762	HA	Bulgaria	2011-Oct-22	A/duck/Bulgaria/Shishmanzi-23/11	NDRVMI (National Diagnostic and Research Veterinary Medical Institute)	Animal and Plant Health Agency (APHA)	Puranik, A; Hanna, A; Essen, S; Focosi-Snyman, R; Manvell, R; Marinova-Petkova, A; Georgiev, G; Webby, R.J; Webster, R.G; Reid, S
EPI287375	HA	Egypt	2010-Jul-02	A/duck/Egypt/3982-21/2010	Istituto Zooprofilattico Sperimentale Delle Venezie	Istituto Zooprofilattico Sperimentale Delle Venezie	
EPI246379	HA	Germany	2009-Jan-01	A/wild duck/Germany/R2892/09		Friedrich-Loeffler-Institut	
EPI241721	HA	Denmark	2006-Mar-15	A/tufted duck/Denmark/6431/2006	National Veterinary Institute	Statens Serum Institute	Bragstad,K.; Fomsgaard,A.; Jorgensen,P.H.; Hammer,A.S.; Kabell,S.; Handberg,K.
EPI241673	HA	Denmark	2006-Mar-19	A/tufted duck/Denmark/6540/2006	National Veterinary Institute	Statens Serum Institute	Bragstad,K.; Fomsgaard,A.; Jorgensen,P.H.; Hammer,A.S.; Kabell,S.; Handberg,K.
EPI210109	HA	Italy	2007-Jan-01	A/duck/Italy/4445/2007	Istituto Zooprofilattico Sperimentale Delle Venezie	Istituto Zooprofilattico Sperimentale Delle Venezie	
EPI186118	HA	Germany	2008-Jan-01	A/duck/Germany/R854/2008		Friedrich-Loeffler-Institut	
EPI173273	HA	France	2009-Jan-30	A/duck/France/090043/2009		AFSSA	
EPI166709	HA	Germany	2008-Oct-01	A/domestic duck/Germany/R874/2008		Friedrich-Loeffler-Institut	
EPI164739	HA	China	2004-Jan-01	A/Duck/Hunnan/70/2004	Harbin Veterinary Research Institute	Harbin Veterinary Research Institute	
EPI164205	HA	Vietnam	2007-Jul-01	A/muscovy duck/Vietnam/NCVD-69/2007		Centers for Disease Control and Prevention	
EPI164165	HA	Vietnam	2007-Jul-01	A/muscovy duck/Vietnam/NCVD-47/2007		Centers for Disease Control and Prevention	
EPI164207	HA	Vietnam	2007-Jul-01	A/duck/Vietnam/NCVD-82/07		Centers for Disease Control and Prevention	
EPI160516	HA	Vietnam	2007-Jul-01	A/duck/Vietnam/NCVD-70/07		Centers for Disease Control and Prevention	
EPI160508	HA	Vietnam	2007-Jul-01	A/duck/Vietnam/NCVD-68/07		Centers for Disease Control and Prevention	
EPI160500	HA	Vietnam	2007-Jul-01	A/duck/Vietnam/NCVD-65/07		Centers for Disease Control and Prevention	
EPI160492	HA	Vietnam	2007-Jul-01	A/duck/Vietnam/NCVD-64/07		Centers for Disease Control and Prevention	
EPI160476	HA	Vietnam	2007-Jul-01	A/duck/Vietnam/NCVD-62/07		Centers for Disease Control and Prevention	
EPI160460	HA	Vietnam	2007-Jul-01	A/duck/Vietnam/NCVD-60/07		Centers for Disease Control and Prevention	
EPI160436	HA	Vietnam	2007-Jul-01	A/duck/Vietnam/NCVD-57/07		Centers for Disease Control and Prevention	
EPI160428	HA	Vietnam	2007-Jul-01	A/duck/Vietnam/NCVD-56/07		Centers for Disease Control and Prevention	
EPI160420	HA	Vietnam	2007-Jul-01	A/duck/Vietnam/NCVD-55/07		Centers for Disease Control and Prevention	
EPI160412	HA	Vietnam	2007-Jul-01	A/duck/Vietnam/NCVD-54/07		Centers for Disease Control and Prevention	
EPI160404	HA	Vietnam	2007-Jul-01	A/duck/Vietnam/NCVD-53/07		Centers for Disease Control and Prevention	
EPI156786	HA	Egypt	2007-Jun-01	A/duck/Egypt/5169-6/2007		Istituto Zooprofilattico Sperimentale Delle Venezie	
EPI156776	HA	Egypt	2007-Feb-01	A/duck/Egypt/5169-4/2007		Istituto Zooprofilattico Sperimentale Delle Venezie	
EPI156731	HA	Egypt	2007-Nov-01	A/duck/Egypt/452-1VIR07/2006		Istituto Zooprofilattico Sperimentale Delle Venezie	
EPI587521	HA	Canada	2014-Jan-01	A/American wigeon/BC/050-31/2015	Animal Health Centre, Ministry of Agriculture	Canadian Food Inspection Agency	
EPI595055	HA	Korea, Republic of	2015-Jan-22	A/common teal/Korea/KU-12/2015	Avian diaseases laboratory, College of Veterinary Medicine, Konkuk University	Konkuk University	Kwon,Jung-Hoon; Yuk,Seong-Su; Erdene-Ochir,TO; Noh,Jin-Yong; Hong,Woo-Tack; Jeong,Jei-Hyun; Jeong,Sol; Song,Chang-Seon
EPI552768	HA	Netherlands	2014-Nov-24	A/eurasian wigeon/Netherlands/emc-2/2014	Erasmus Medical Center	Erasmus Medical Center	Fouchier, Ron A.M.; Verhagen, Josanne H.; Vuong, Oanh; Bestebroer, Theo; Van Vliet, Stefan; Van der Jeugd, Henk
EPI551149	HA	Netherlands	2014-Nov-24	A/eurasian wigeon/Netherlands/emc-2/2014	Erasmus Medical Center	Erasmus Medical Center	Fouchier, Ron A.M.; Verhagen, Josanne H.; Vuong, Oanh; Bestebroer, Theo; Van Vliet, Stefan; Van der Jeugd, Henk
EPI551143	HA	Netherlands	2014-Nov-24	A/eurasian wigeon/Netherlands/emc-1/2014	Erasmus Medical Center	Erasmus Medical Center	Fouchier, Ron A.M.; Verhagen, Josanne H.; Vuong, Oanh; Bestebroer, Theo; Van Vliet, Stefan; Van der Jeugd, Henk
EPI573680	HA	Japan	2015-Feb-13	A/mallard duck/Kagoshima/KU116/2015(H5N8)	Kagoshima University	Kagoshima University	
EPI573672	HA	Japan	2015-Jan-14	A/mallard duck/Kagoshima/KU70/2015(H5N8)	Kagoshima University	Kagoshima University	
EPI573239	HA	Korea, Republic of	2014-Dec-01	A/mallard/Korea/H1924-6/2014		Animal and Plant Quarantine Agency	
EPI573237	HA	Korea, Republic of	2014-Dec-18	A/mallard/Korea/H1991/2014		Animal and Plant Quarantine Agency	
EPI507673	HA	China	2013-Nov-18	A/mallard duck/Shanghai/SH-9/2013	Institute of Military Veterinary, Academy of Military Medical Sciences	Institute of Laboratory Animal Sciences, Chinese Academy	Fan,S.; Gao,X.;Ying,Y.; Guo,J.; Sun,W.; Wang,T.;Ren,Z.; Yu,Z.; Li,Y.; Zhao,Y.; Yang,S.;Gao,Y. ; Xia,X.
EPI257228	HA	Belgium	2008-Dec-01	A/Anas platyrhynchos/09-884/2008	Veterinary and Agrochemical Research Institute	Veterinary and Agrochemical Research Institute	Van Borm,Steven
EPI257220	HA	Belgium	2008-Nov-01	A/Anas platyrhynchos/Belgium/09-762-P1/2008	Veterinary and Agrochemical Research Institute	Veterinary and Agrochemical Research Institute	Van Borm,Steven
EPI210107	HA	Italy	2007-Jan-01	A/mallard/Italy/5582-21/2007	Istituto Zooprofilattico Sperimentale Delle Venezie	Istituto Zooprofilattico Sperimentale Delle Venezie	
EPI169267	HA	France	2006-Nov-08	A/duck/France/06964/2006		AFSSA	
EPI573179	HA	Netherlands	2014-Nov-21	A/duck/Netherlands/14015898/2014	Central Veterinary Institute	Central Veterinary Institute	Heutink, Rene; Harders, Frank; Verschuren-Pritz, Sylvia; Bossers, Alex; Koch, Guus; Bouwstra, Ruth
EPI462795	HA	Austria	2013-Jun-12	A/mynah/Austria-quarantine/13064792-010/13	Institute for Veterinary Disease Control Moedling, Austrian Agency for Health and Food Safety	Animal and Plant Health Agency (APHA)	Collins, S; Hanna, A; Essen, S; Focosi-Snyman, R; Manvell, R; Wodak, E; Revilla-Fernandez, S; Bago, Z; Schmoll, F; Reid, S
EPI462791	HA	Austria	2013-Jun-07	A/mynah/Austria-quarantine/13063485-025/13	Institute for Veterinary Disease Control Moedling, Austrian Agency for Health and Food Safety	Animal and Plant Health Agency (APHA)	Collins, S; Hanna, A; Essen, S; Focosi-Snyman, R; Manvell, R; Wodak, E; Revilla-Fern?ndez, S; Bag?, Z; Schmoll, F; Reid, S
EPI595107	HA	Korea, Republic of	2014-Dec-24	A/mandarin duck/Korea/K14-367-1/2014	Avian diaseases laboratory, College of Veterinary Medicine, Konkuk University	Konkuk University	Kwon,Jung-Hoon; Yuk,Seong-Su; Erdene-Ochir,TO; Noh,Jin-Yong; Hong,Woo-Tack; Jeong,Jei-Hyun; Jeong,Sol; Song,Chang-Seon
EPI595094	HA	Korea, Republic of	2014-Dec-24	A/mandarin duck/Korea/K14-366-1/2014	Avian diaseases laboratory, College of Veterinary Medicine, Konkuk University	Konkuk University	Kwon,Jung-Hoon; Yuk,Seong-Su; Erdene-Ochir,TO; Noh,Jin-Yong; Hong,Woo-Tack; Jeong,Jei-Hyun; Jeong,Sol; Song,Chang-Seon
EPI305547	HA	Turkey	2006-Jan-07	A/Goose/Turkey-Erzurum/09rs2841-24/2006	Istituto Zooprofilattico Sperimentale Delle Venezie	Istituto Zooprofilattico Sperimentale Delle Venezie	Schivo,A.; Valastro,V.; Monne,I.; Coven,F.; Fusaro,A.; Dakman,A.; Akcadag,B.; Salviato,A.; DeBattisti,C.; Capua,I.; Cattoli,G.
EPI595146	HA	Korea, Republic of	2014-Dec-24	A/greater white-fronted goose/Korea/K14-374-1/2014	Avian diaseases laboratory, College of Veterinary Medicine, Konkuk University	Konkuk University	Kwon,Jung-Hoon; Yuk,Seong-Su; Erdene-Ochir,TO; Noh,Jin-Yong; Hong,Woo-Tack; Jeong,Jei-Hyun; Jeong,Sol; Song,Chang-Seon
EPI595138	HA	Korea, Republic of	2014-Dec-24	A/greater white-fronted goose/Korea/K14-372-2/2014	Avian diaseases laboratory, College of Veterinary Medicine, Konkuk University	Konkuk University	Kwon,Jung-Hoon; Yuk,Seong-Su; Erdene-Ochir,TO; Noh,Jin-Yong; Hong,Woo-Tack; Jeong,Jei-Hyun; Jeong,Sol; Song,Chang-Seon
EPI595133	HA	Korea, Republic of	2014-Dec-24	A/greater white-fronted goose/Korea/K14-371-4/2014	Avian diaseases laboratory, College of Veterinary Medicine, Konkuk University	Konkuk University	Kwon,Jung-Hoon; Yuk,Seong-Su; Erdene-Ochir,TO; Noh,Jin-Yong; Hong,Woo-Tack; Jeong,Jei-Hyun; Jeong,Sol; Song,Chang-Seon
EPI595124	HA	Korea, Republic of	2014-Dec-24	A/greater white-fronted goose/Korea/K14-369-3/2014	Avian diaseases laboratory, College of Veterinary Medicine, Konkuk University	Konkuk University	Kwon,Jung-Hoon; Yuk,Seong-Su; Erdene-Ochir,TO; Noh,Jin-Yong; Hong,Woo-Tack; Jeong,Jei-Hyun; Jeong,Sol; Song,Chang-Seon
EPI154493	HA	Saudi Arabia	2007-Jan-01	A/ostrich/Saudi Arabia/6732-3/2007		Istituto Zooprofilattico Sperimentale Delle Venezie	
EPI169516	HA	Saudi Arabia	2007-Jan-01	A/peacock/Saudi Arabia/3489-74VIR08/2007		Istituto Zooprofilattico Sperimentale Delle Venezie	
EPI559910	HA	China	2015-Jan-05	A/whooper swan/Henan/SMX9/2015(H5N1)	CAS Key Laboratory of Pathogenic Microbiology and Immunology, Institute of Microbiology, Chinese Academy of Sciences	Institute of Microbiology, Chinese Academy of Sciences	

EPI576391	HA	Sweden	2015-Feb-18	A/MuteSwan/Sweden/SVA-1503130141-SZ543/2015	National Veterinary Institute	National Veterinary Institute	'Zohari,Siamak';'Karin,Ullman';Olofsson,Ann-Sophie*
EPI576384	HA	Sweden	2015-Feb-18	A/MuteSwan/Sweden/SVA-U1503110277-SZ502/2015	National Veterinary Institute	National Veterinary Institute	'Zohari,Siamak';'Ullman,Karin'; 'Olofson,Ann-Sophie'
EPI156821	HA	Poland	2006-Mar-10	A/swan/Poland/467-136V08/2006		National Veterinary Research Institute	
EPI586509	HA	Canada	2014-Dec-08	A/poultry/BC/FAV19/2014	Animal Health Centre, Ministry of Agriculture	Canadian Food Inspection Agency	
EPI557136	HA	Egypt	2014-May-15	A/Turkey/Egypt/BSU5114-AR2218/2014		Friedrich-Loeffler-Institut	Naguib,M.M; El-Kady,M.F; Hassan, K.E; Abo-Zeid,H.H; Beer,M; Harder,TC
EPI553144	HA	Italy	2014-Dec-15	A/turkey/Italy/14VIR7898-10/2014	Istituto Zooprofilattico Sperimentale Delle Venezie	Istituto Zooprofilattico Sperimentale Delle Venezie	Luca,Tassoni; Silvia,Ormelli; Alessia,Schivo; Alice,Fusaro; Isabella,Monne; Giovanni,Cattoli
EPI464938	HA	Italy	2012-Aug-31	A/turkey/Italy/12VIR-6607-5/2012	Istituto Zooprofilattico Sperimentale Delle Venezie	Istituto Zooprofilattico Sperimentale Delle Venezie	Monne, I.; Salviato, A.; Tassoni, L.; Cattoli, G.
EPI305600	HA	Turkey	2007-Feb-15	A/Turkey/Turkey/Batman/09rs2842-107/2007	Istituto Zooprofilattico Sperimentale Delle Venezie	Istituto Zooprofilattico Sperimentale Delle Venezie	Schivo,A.; Valastro,V.; Monne,I.; Coven,F.; Fusaro,A.; Dakman,A.; Akcadag,B.; Salviato,A.; DeBattisti,C.; Capua,I.; Cattoli,G.
EPI305593	HA	Turkey	2006-Mar-09	A/Turkey/Turkey/Batman/09rs2841-120/2006	Istituto Zooprofilattico Sperimentale Delle Venezie	Istituto Zooprofilattico Sperimentale Delle Venezie	Schivo,A.; Valastro,V.; Monne,I.; Coven,F.; Fusaro,A.; Dakman,A.; Akcadag,B.; Salviato,A.; DeBattisti,C.; Capua,I.; Cattoli,G.
EPI305573	HA	Turkey	2006-Feb-08	A/Turkey/Turkey/Konya/09rs2841-84/2006	Istituto Zooprofilattico Sperimentale Delle Venezie	Istituto Zooprofilattico Sperimentale Delle Venezie	Schivo,A.; Valastro,V.; Monne,I.; Coven,F.; Fusaro,A.; Dakman,A.; Akcadag,B.; Salviato,A.; DeBattisti,C.; Capua,I.; Cattoli,G.
EPI169514	HA	Saudi Arabia	2007-Jan-01	A/turkey/Saudi Arabia/3489-70VIR08/2007		Istituto Zooprofilattico Sperimentale Delle Venezie	
EPI631920	HA	Russian Federation	2015-May-07	A/rook/Dovolnoe/50/2015	State Research Center of Virology and Biotechnology Vector	State Research Center of Virology and Biotechnology Vector	Ivan,Susloparov; Vasily,Marchenko; Natalya,Goncharova; Andrey,Shipovalov; Alexander,Durymanov; Tatyana,Ilyicheva; Alexander,Ryzhikov
EPI623555	HA	United Arab Emirates	2014-Nov-30	A/Hoabara/Dubai/2455.5/2014		Friedrich-Loeffler-Institut	Chen, H.; Chan, KH. ; Wong, PC. ; Woo, C.Y.P.
EPI603577	HA	United Arab Emirates	2014-Dec-07	A/Quail/Dubai/AR3445-25043/2014		Friedrich-Loeffler-Institut	Naguib, M.M.; Wernery, U.; Harder, T.
EPI594560	HA	Romania	2015-Mar-26	A/pelican/Romania/12449/2015	Institute for Diagnosis & Animal Health (IDAH)	Animal and Plant Health Agency (APHA)	Thomas, S; Puranik, A; Londt, B; Essen, S; Manvell, R; Onita, I; Neicut, A; Cioranu, R; Motiu, R; Banks, J; Brown, I
EPI156829	HA	Poland	2006-Mar-24	A/hawk/Poland/937-138V08/2006		National Veterinary Research Institute	
EPI156813	HA	Poland	2006-Mar-11	A/goosander/Poland/502-137V08/2006		National Veterinary Research Institute	
EPI156805	HA	Poland	2007-Dec-11	A/buzzard/Poland/MB266B-141V08/2007		National Veterinary Research Institute	
EPI156752	HA	Togo	2007-Jan-01	A/avian/Togo/3618-10/2007		Istituto Zooprofilattico Sperimentale Delle Venezie	
EPI448255	HA	Bangladesh	2011-Dec-30	A/environment/Bangladesh/1017-1/2011	Institute of Epidemiology Disease Control and Research (IEDCR) & Bangladesh National Influenza Centre (NIC)	Centers for Disease Control and Prevention	Gerloff, Nancy; Simpson, Natosha; Poh, Mee; Davis, Todd
EPI448119	HA	Bangladesh	2011-May-29	A/environment/Bangladesh/1017/2011	Institute of Epidemiology Disease Control and Research (IEDCR) & Bangladesh National Influenza Centre (NIC)	Centers for Disease Control and Prevention	Gerloff, Nancy; Simpson, Natosha; Poh, Mee;Davis, Todd
EPI280256	HA	China	2009-Jan-16	A/water/Xinjiang/3/2009	WHO Chinese National Influenza Center	WHO Chinese National Influenza Center	Lan,Yu,Dong,Libo,Li,Zi,Li,Xiyan,Zhao,Xiang,Cheng,Yanhui,Tan,minju,Yang,Lei,Zou,Shumei,Wen,Leying,Wang,Dayan,Li,Dexin,Shu,Yuelong
EPI280232	HA	China	2009-Jan-16	A/environment/Guizhou/4/2009	WHO Chinese National Influenza Center	WHO Chinese National Influenza Center	Lan,Yu,Dong,Libo,Li,Zi,Li,Xiyan,Zhao,Xiang,Cheng,Yanhui,Tan,minju,Yang,Lei,Zou,Shumei,Wen,Leying,Wang,Dayan,Li,Dexin,Shu,Yuelong
EPI280208	HA	China	2009-Jan-06	A/water/Hebei/3/2009	WHO Chinese National Influenza Center	WHO Chinese National Influenza Center	Lan,Yu,Dong,Libo,Li,Zi,Li,Xiyan,Zhao,Xiang,Cheng,Yanhui,Tan,minju,Yang,Lei,Zou,Shumei,Wen,Leying,Wang,Dayan,Li,Dexin,Shu,Yuelong
EPI594523	HA	China	2013-Feb-26	A/environment/Hubei/950/2013	Xiangfan Center for Disease Control and Prevention	WHO Chinese National Influenza Center	Li, Xiaodan; Gao, Rongbao; Bo, Hong; Zhang, Ye; Wang, Dayan; Shu, Yuelong
EPI454493	HA	China	2013-Apr-12	A/environment/Hangzhou/109-2/2013(H5N1)	Hangzhou Center for Disease Control and Prevention	Hangzhou Center for Disease Control and Prevention	Li,J;Jin,T;Yu,XF;Pu,XY;Pan,JC
EPI643147	HA	China	2013-Sep-10	A/Environment/Guangdong/GZ55/2013(H5N8)	Guangdong Provincial Center for Disease Control and Prevention	Guangdong Center of Disease Control and Prevention	
EPI432517	HA	China	2010-Apr-27	r7	Harbin Veterinary Research Institute	Harbin Veterinary Research Institute	
EPI432509	HA	China	2010-Apr-27	r68	Harbin Veterinary Research Institute	Harbin Veterinary Research Institute	
EPI432501	HA	China	2010-Apr-27	r678	Harbin Veterinary Research Institute	Harbin Veterinary Research Institute	
EPI432493	HA	China	2010-Apr-27	r67	Harbin Veterinary Research Institute	Harbin Veterinary Research Institute	
EPI432477	HA	China	2010-Apr-21	r58	Harbin Veterinary Research Institute	Harbin Veterinary Research Institute	
EPI432437	HA	China	2010-Apr-21	r567	Harbin Veterinary Research Institute	Harbin Veterinary Research Institute	
EPI432413	HA	China	2010-Apr-16	r38	Harbin Veterinary Research Institute	Harbin Veterinary Research Institute	
EPI432365	HA	China	2010-Apr-10	r36	Harbin Veterinary Research Institute	Harbin Veterinary Research Institute	
EPI432357	HA	China	2010-Apr-10	r358	Harbin Veterinary Research Institute	Harbin Veterinary Research Institute	
EPI432333	HA	China	2010-Apr-03	r3568	Harbin Veterinary Research Institute	Harbin Veterinary Research Institute	
EPI432325	HA	China	2010-Apr-03	r35678	Harbin Veterinary Research Institute	Harbin Veterinary Research Institute	
EPI432277	HA	China	2010-Mar-28	r278	Harbin Veterinary Research Institute	Harbin Veterinary Research Institute	
EPI432213	HA	China	2010-Mar-22	r257	Harbin Veterinary Research Institute	Harbin Veterinary Research Institute	
EPI432205	HA	China	2010-Mar-22	r2568	Harbin Veterinary Research Institute	Harbin Veterinary Research Institute	
EPI432197	HA	China	2010-Mar-22	r25678	Harbin Veterinary Research Institute	Harbin Veterinary Research Institute	
EPI432157	HA	China	2010-Mar-17	r2378	Harbin Veterinary Research Institute	Harbin Veterinary Research Institute	
EPI432109	HA	China	2010-Mar-12	r2358	Harbin Veterinary Research Institute	Harbin Veterinary Research Institute	
EPI432101	HA	China	2010-Mar-12	r23578	Harbin Veterinary Research Institute	Harbin Veterinary Research Institute	
EPI432093	HA	China	2010-Mar-07	r2357	Harbin Veterinary Research Institute	Harbin Veterinary Research Institute	
EPI432053	HA	China	2010-Mar-07	r235	Harbin Veterinary Research Institute	Harbin Veterinary Research Institute	
EPI432045	HA	China	2010-Mar-02	r23	Harbin Veterinary Research Institute	Harbin Veterinary Research Institute	
EPI432021	HA	China	2010-Mar-02	r178	Harbin Veterinary Research Institute	Harbin Veterinary Research Institute	
EPI432013	HA	China	2010-Mar-02	r17	Harbin Veterinary Research Institute	Harbin Veterinary Research Institute	
EPI431997	HA	China	2012-Feb-25	r1678	Harbin Veterinary Research Institute	Harbin Veterinary Research Institute	
EPI431965	HA	China	2012-Feb-25	r1578	Harbin Veterinary Research Institute	Harbin Veterinary Research Institute	
EPI431957	HA	China	2012-Feb-25	r157	Harbin Veterinary Research Institute	Harbin Veterinary Research Institute	
EPI431917	HA	China	2010-Feb-18	r15	Harbin Veterinary Research Institute	Harbin Veterinary Research Institute	
EPI431893	HA	China	2010-Feb-10	r137	Harbin Veterinary Research Institute	Harbin Veterinary Research Institute	
EPI431877	HA	China	2010-Feb-10	r13678	Harbin Veterinary Research Institute	Harbin Veterinary Research Institute	
EPI431861	HA	China	2010-Feb-10	r136	Harbin Veterinary Research Institute	Harbin Veterinary Research Institute	
EPI431853	HA	China	2010-Feb-05	r1358	Harbin Veterinary Research Institute	Harbin Veterinary Research Institute	
EPI431813	HA	China	2010-Feb-05	r13567	Harbin Veterinary Research Institute	Harbin Veterinary Research Institute	
EPI431797	HA	China	2010-Jan-31	r135	Harbin Veterinary Research Institute	Harbin Veterinary Research Institute	
EPI431773	HA	China	2010-Jan-31	r1278	Harbin Veterinary Research Institute	Harbin Veterinary Research Institute	
EPI431757	HA	China	2010-Jan-26	r1268	Harbin Veterinary Research Institute	Harbin Veterinary Research Institute	
EPI431749	HA	China	2010-Jan-26	r12678	Harbin Veterinary Research Institute	Harbin Veterinary Research Institute	
EPI431693	HA	China	2010-Jan-20	r125678	Harbin Veterinary Research Institute	Harbin Veterinary Research Institute	
EPI431677	HA	China	2010-Jan-20	r1256	Harbin Veterinary Research Institute	Harbin Veterinary Research Institute	
EPI431669	HA	China	2010-Jan-20	r125	Harbin Veterinary Research Institute	Harbin Veterinary Research Institute	

EPI431661	HA	China	2010-Jan-16	r1238	Harbin Veterinary Research Institute	Harbin Veterinary Research Institute	
EPI431653	HA	China	2010-Jan-16	r12378	Harbin Veterinary Research Institute	Harbin Veterinary Research Institute	
EPI431637	HA	China	2010-Jan-16	r12368	Harbin Veterinary Research Institute	Harbin Veterinary Research Institute	
EPI431629	HA	China	2010-Jan-16	r123678	Harbin Veterinary Research Institute	Harbin Veterinary Research Institute	
EPI431605	HA	China	2010-Jan-10	r12358	Harbin Veterinary Research Institute	Harbin Veterinary Research Institute	
EPI431573	HA	China	2010-Jan-10	r1235678	Harbin Veterinary Research Institute	Harbin Veterinary Research Institute	
EPI431565	HA	China	2010-Jan-04	r123567	Harbin Veterinary Research Institute	Harbin Veterinary Research Institute	
EPI431549	HA	China	2010-Jan-04	r1235	Harbin Veterinary Research Institute	Harbin Veterinary Research Institute	
EPI431525	HA	China	2010-Jan-04	r1	Harbin Veterinary Research Institute	Harbin Veterinary Research Institute	
EPI338339	HA	Germany	2009-Jan-01	A/hen's egg/Germany/[A/cygnus cygnus/Germany/R65/2006]-EscEgg50A-escape/2009 (H5N1)	Friedrich-Loeffler-Institut	Friedrich-Loeffler-Institut	Kalthoff,Donata ;Röhrs,Susanne ;Höper,Dirk ;Hoffmann,Bernd ;Bogs,Jessica ;Stech,Jürgen ;Beer,Martin
EPI287348	HA	Germany	2009-Jan-01	A/MDCK/Germany/[A/cygnus cygnus/Germany/R65/2006]-CoQ50-control/2009 (H5N1)	Friedrich-Loeffler-Institut	Friedrich-Loeffler-Institut	Hoeper,Dirk; Kalthoff,Donata; Hoffmann,Bernd; Beer,Martin
EPI287300	HA	Germany	2009-Jan-01	A/MDCK/Germany/[A/cygnus cygnus/Germany/R65/2006]-Q50-escape/2009 (H5N1)	Friedrich-Loeffler-Institut	Friedrich-Loeffler-Institut	Hoeper,Dirk; Kalthoff,Donata; Hoffmann,Bernd; Beer,Martin
EPI287292	HA	Germany	2009-Jan-01	A/MDCK/Germany/[A/cygnus cygnus/Germany/R65/2006]-Q30-escape/2009 (H5N1)	Friedrich-Loeffler-Institut	Friedrich-Loeffler-Institut	Hoeper,Dirk; Kalthoff,Donata; Hoffmann,Bernd; Beer,Martin
EPI287284	HA	Germany	2009-Jan-01	A/MDCK/Germany/[A/cygnus cygnus/Germany/R65/2006]-Q18-escape/2009 (H5N1)	Friedrich-Loeffler-Institut	Friedrich-Loeffler-Institut	Hoeper,Dirk; Kalthoff,Donata; Hoffmann,Bernd; Beer,Martin
EPI287252	HA	Germany	2009-Jan-01	A/MDCK/Germany/[A/cygnus cygnus/Germany/R65/2006]-PP100c-escape/2009 (H5N1)	Friedrich-Loeffler-Institut	Friedrich-Loeffler-Institut	Hoeper,Dirk; Kalthoff,Donata; Hoffmann,Bernd; Beer,Martin
EPI287220	HA	Germany	2008-Jan-01	A/MDCK/Germany/[A/cygnus cygnus/Germany/R65/2006]-P30-escape/2008 (H5N1)	Friedrich-Loeffler-Institut	Friedrich-Loeffler-Institut	Hoeper,Dirk; Kalthoff,Donata; Hoffmann,Bernd; Beer,Martin
EPI174803	HA	Vietnam	2008-Jan-01	A/chicken/Vietnam/NCVD-016/2008(H5N1)-PR8-IDCDC-RG12		Centers for Disease Control and Prevention	
EPI642537	HA	Egypt	2015-Jan-15	A/Egypt/682/2015	Ministry of Health and Population	Crick Worldwide Influenza Centre	
EPI533583	HA	China	2014-Apr-21	A/Sichuan/26221/2014		WHO Chinese National Influenza Center	
EPI497961	HA	Cambodia	2013-Oct-25	A/Cambodia/X1030304/2013	Institut Pasteur in Cambodia	Institut Pasteur in Cambodia	Horm, S-V; Rith, S; Buchy, P.
EPI487549	HA	Indonesia	2011-Feb-03	A/Indonesia/NIHRD11046/2011	National Institute of Health Research and Development	National Institute of Health Research and Development	HA,Pawestri
EPI487525	HA	Indonesia	2010-Jun-01	A/Indonesia/NIHRD10459/2010	National Institute of Health Research and Development	National Institute of Health Research and Development	HA,Pawestri
EPI487467	HA	Indonesia	2010-Sep-16	A/Indonesia/NIHRD10623/2010	National Institute of Health Research and Development	National Institute of Health Research and Development	HA,Pawestri
EPI487454	HA	Indonesia	2010-Nov-21	A/Indonesia/NIHRD10728/2010	National Institute of Health Research and Development	National Institute of Health Research and Development	HA,Pawestri
EPI486487	HA	Indonesia	2009-Feb-27	A/Indonesia/NIHRD9340/2009	National Institute of Health Research and Development	National Institute of Health Research and Development	HA,Pawestri
EPI485818	HA	Indonesia	2008-Apr-23	A/Indonesia/NIHRD7988/2008	National Institute of Health Research and Development	National Institute of Health Research and Development	HA,Pawestri
EPI485581	HA	Cambodia	2013-Jan-25	A/Cambodia/X0125302/2013	Institut Pasteur in Cambodia	Institut Pasteur in Cambodia	Rith, S.; Horm, SV.; Buchy, P.
EPI463648	HA	Indonesia	2013-Jun-18	A/Indonesia/NIHRD13157/2013	National Institute of Health Research and Development	National Institute of Health Research and Development	HA,Pawestri;AA,Nugraha;V,Setiawaty
EPI448095	HA	Bangladesh	2011-Mar-07	A/Bangladesh/5487/2011	Institute of Epidemiology Disease Control and Research (IEDCR) & Bangladesh National Influenza Centre (NIC)	Centers for Disease Control and Prevention	Gerloff, Nancy; Simpson, Natosha; Poh, Mee;Davis, Todd
EPI373019	HA	Egypt	2011-Jan-01	A/Egypt/N04285/2011	U.S. Naval Medical Research Unit No.3	Centers for Disease Control and Prevention	Younan, M.; Elassal, E.; ElBadry, M.A.; Naguib, A.;Saied, I.; Kandeel, A.; Cornelius, C.
EPI373018	HA	Egypt	2011-Jan-01	A/Egypt/N00002/2011	U.S. Naval Medical Research Unit No.3	Centers for Disease Control and Prevention	Younan, M.; Elassal, E.; ElBadry, M.A.; Naguib, A.;Saied, I.; Kandeel, A.; Cornelius, C.
EPI373017	HA	Egypt	2011-Jan-01	A/Egypt/N05860/2011	U.S. Naval Medical Research Unit No.3	Centers for Disease Control and Prevention	Younan, M.; Elassal, E.; ElBadry, M.A.; Naguib, A.;Saied, I.; Kandeel, A.; Cornelius, C.
EPI373016	HA	Egypt	2010-Jan-01	A/Egypt/N10954/2010	U.S. Naval Medical Research Unit No.3	Centers for Disease Control and Prevention	Younan, M.; Elassal, E.; ElBadry, M.A.; Naguib, A.;Saied, I.; Kandeel, A.; Cornelius, C.
EPI373015	HA	Egypt	2011-Jan-01	A/Egypt/N04284/2011	U.S. Naval Medical Research Unit No.3	Centers for Disease Control and Prevention	Younan, M.; Elassal, E.; ElBadry, M.A.; Naguib, A.;Saied, I.; Kandeel, A.; Cornelius, C.
EPI372955	HA	Egypt	2011-Dec-21	A/Egypt/N14976/2011	U.S. Naval Medical Research Unit No.3	Centers for Disease Control and Prevention	Younan, M.; Poh, M.K.; Elassal, E.; Simpson, N.; Jones,J.; ElBadry,M.A.; Naguib, A.;Saied, I.; Kandeel,A.; Cornelius, C.
EPI372947	HA	Egypt	2011-Dec-15	A/Egypt/N11470/2011	U.S. Naval Medical Research Unit No.3	Centers for Disease Control and Prevention	Younan, M.; Poh, M.K.; Elassal, E.; Simpson, N.; Jones,J.; ElBadry,M.A.; Naguib, A.;Saied, I.; Kandeel,A.; Cornelius, C.
EPI372931	HA	Egypt	2011-Sep-21	A/Egypt/N09966/2011	U.S. Naval Medical Research Unit No.3	Centers for Disease Control and Prevention	Younan, M.; Poh, M.K.; Elassal, E.; Simpson, N.; Jones,J.; ElBadry,M.A.; Naguib, A.;Saied, I.; Kandeel,A.; Cornelius, C.
EPI372915	HA	Egypt	2011-Jan-01	A/Egypt/N7592/2011	U.S. Naval Medical Research Unit No.3	Centers for Disease Control and Prevention	Younan, M.; Poh, M.K.; Elassal, E.; Simpson, N.; Jones,J.; ElBadry,M.A.; Naguib, A.;Saied, I.; Kandeel,A.; Cornelius, C.
EPI372899	HA	Egypt	2011-Jan-01	A/Egypt/N0423/2011	U.S. Naval Medical Research Unit No.3	Centers for Disease Control and Prevention	Younan, M.; Poh, M.K.; Elassal, E.; Simpson, N.; Jones,J.; ElBadry,M.A.; Naguib, A.;Saied, I.; Kandeel,A.; Cornelius, C.
EPI372891	HA	Egypt	2011-Jan-01	A/Egypt/N6828/2011	U.S. Naval Medical Research Unit No.3	Centers for Disease Control and Prevention	Younan, M.; Poh, M.K.; Elassal, E.; Simpson, N.; Jones,J.; ElBadry,M.A.; Naguib, A.;Saied, I.; Kandeel,A.; Cornelius, C.
EPI372875	HA	Egypt	2011-Jan-01	A/Egypt/N0677/2011	U.S. Naval Medical Research Unit No.3	Centers for Disease Control and Prevention	Younan, M.; Poh, M.K.; Elassal, E.; Simpson, N.; Jones,J.; ElBadry,M.A.; Naguib, A.;Saied, I.; Kandeel,A.; Cornelius, C.
EPI372867	HA	Egypt	2011-Jan-01	A/Egypt/N6658/2011	U.S. Naval Medical Research Unit No.3	Centers for Disease Control and Prevention	Younan, M.; Poh, M.K.; Elassal, E.; Simpson, N.; Jones,J.; ElBadry,M.A.; Naguib, A.;Saied, I.; Kandeel,A.; Cornelius, C.
EPI372753	HA	Egypt	2009-May-13	A/Egypt/4935-NAMRU3/2009	U.S. Naval Medical Research Unit No.3	Centers for Disease Control and Prevention	Younan, M.; Poh, M.K.; Elassal, E.; Simpson, N.; Jones,J.; ElBadry,M.A.; Naguib, A.;Saied, I.; Kandeel,A.; Cornelius, C.
EPI372659	HA	Egypt	2008-Jan-01	A/Egypt/2472-NAMRU3/2008	U.S. Naval Medical Research Unit No.3	Centers for Disease Control and Prevention	Younan, M.; Poh, M.K.; Elassal, E.; Simpson, N.; Jones,J.; ElBadry,M.A.; Naguib, A.;Saied, I.; Kandeel,A.; Cornelius, C.
EPI356474	HA	Indonesia	2012-Jan-13	A/Indonesia/NIHRD11949/2012	National Institute of Health Research and Development	National Institute of Health Research and Development	Pawestri,H.A;Puspa,K.D;Ikawati,H.D;Setiawaty,V;Sampurno,O.D
EPI356472	HA	Indonesia	2012-Jan-07	A/Indonesia/NIHRD11931/2012	National Institute of Health Research and Development	National Institute of Health Research and Development	Pawestri,H.A;Puspa,K.D;Ikawati,H.D;Setiawaty,V;Sampurno,O.D
EPI352222	HA	China	2012-Jan-22	A/Guizhou/1/2012		WHO Chinese National Influenza Center	
EPI341634	HA	Indonesia	2011-Oct-16	A/Indonesia/NIHRD11797/2011(H5N1)	National Institute of Health Research and Development	National Institute of Health Research and Development	Pawestri,H.A;Setiawaty,V;Sampurno,O.D

EPI341633	HA	Indonesia	2011-Oct-07	A/Indonesia/NIHRD11771/2011(H5N1)	National Institute of Health Research and Development	National Institute of Health Research and Development	Pawestri,H.A;Setiawaty,V;Sampurno,O.D;Takayama,I;Shirakura,M;Kageyama,T;Tashiro,M
EPI341332	HA	Indonesia	2011-Feb-06	A/Indonesia/NIHRD11073/2011(H5N1)	National Institute of Health Research and Development	National Institute of Health Research and Development	'Vivi,Setiawaty; Hana,Pawestri; Eka,Pratiwi; Fera,Ibrahim'
EPI280168	HA	China	2009-Jan-08	A/Hunan/1/2009	WHO Chinese National Influenza Center	WHO Chinese National Influenza Center	Lan,Yu,Dong,Libo,Li,Zi,Li,Xiyan,Zhao,Xiang,Cheng,Yanhui,Tan,minju,Yang,Lei,Zou,Shumei,Wen,Leying,Wang,Dayan,Li,Dexin,Shu,Yuelong
EPI280160	HA	China	2008-Dec-04	A/Beijing/1/2009	WHO Chinese National Influenza Center	WHO Chinese National Influenza Center	Lan,Yu,Dong,Libo,Li,Zi,Li,Xiyan,Zhao,Xiang,Cheng,Yanhui,Tan,minju,Yang,Lei,Zou,Shumei,Wen,Leying,Wang,Dayan,Li,Dexin,Shu,Yuelong
EPI280152	HA	China	2007-Dec-03	A/Jiangsu/2/2007	WHO Chinese National Influenza Center	WHO Chinese National Influenza Center	Lan,Yu,Dong,Libo,Li,Zi,Li,Xiyan,Zhao,Xiang,Cheng,Yanhui,Tan,minju,Yang,Lei,Zou,Shumei,Wen,Leying,Wang,Dayan,Li,Dexin,Shu,Yuelong
EPI280144	HA	China	2007-Nov-24	A/Jiangsu/1/2007	WHO Chinese National Influenza Center	WHO Chinese National Influenza Center	Lan,Yu,Dong,Libo,Li,Zi,Li,Xiyan,Zhao,Xiang,Cheng,Yanhui,Tan,minju,Yang,Lei,Zou,Shumei,Wen,Leying,Wang,Dayan,Li,Dexin,Shu,Yuelong
EPI280120	HA	China	2006-Apr-01	A/Hubei/1/2006	WHO Chinese National Influenza Center	WHO Chinese National Influenza Center	Lan,Yu,Dong,Libo,Li,Zi,Li,Xiyan,Zhao,Xiang,Cheng,Yanhui,Tan,minju,Yang,Lei,Zou,Shumei,Wen,Leying,Wang,Dayan,Li,Dexin,Shu,Yuelong
EPI280112	HA	China	2006-Mar-13	A/Shanghai/1/2006	WHO Chinese National Influenza Center	WHO Chinese National Influenza Center	Lan,Yu,Dong,Libo,Li,Zi,Li,Xiyan,Zhao,Xiang,Cheng,Yanhui,Tan,minju,Yang,Lei,Zou,Shumei,Wen,Leying,Wang,Dayan,Li,Dexin,Shu,Yuelong
EPI280104	HA	China	2006-Feb-22	A/Guangdong/1/2006	WHO Chinese National Influenza Center	WHO Chinese National Influenza Center	Lan,Yu,Dong,Libo,Li,Zi,Li,Xiyan,Zhao,Xiang,Cheng,Yanhui,Tan,minju,Yang,Lei,Zou,Shumei,Wen,Leying,Wang,Dayan,Li,Dexin,Shu,Yuelong
EPI280025	HA	China	2006-Jun-03	A/Guangdong/2/2006	WHO Chinese National Influenza Center	WHO Chinese National Influenza Center	Lan,Yu; Dong,Libo; Li,Zi; Li,Xiyan; Zhao,Xiang; Cheng,Yanhui; Tan,minju; Yang,Lei; Zou,Shumei; Wen,Leying; Wang,Dayan; Li,Dexin; Shu,Yuelong
EPI280018	HA	China	2006-Apr-16	A/Sichuan/3/2006	WHO Chinese National Influenza Center	WHO Chinese National Influenza Center	Lan,Yu; Dong,Libo; Li,Zi; Li,Xiyan; Zhao,Xiang; Cheng,Yanhui; Tan,minju; Yang,Lei; Zou,Shumei; Wen,Leying; Wang,Dayan; Li,Dexin; Shu,Yuelong
EPI176348	HA	China	2009-Jan-24	A/Guangxi/1/2009		WHO Chinese National Influenza Center	
EPI164763	HA	Indonesia	2008-Jan-01	A/Indonesia/7272/2008	Ministry of Health, NIHRD	Eijkman Institute for Molecular Biology	
EPI164266	HA	China	2005-Dec-10	A/Jiangxi/1/2005		WHO Chinese National Influenza Center	
EPI164250	HA	China	2005-Dec-12	A/Fujian/1/2005		WHO Chinese National Influenza Center	
EPI164240	HA	China	2005-Nov-17	A/Anhui/2/2005		WHO Chinese National Influenza Center	
EPI164224	HA	China	2006-Jan-11	A/Sichuan/1/2006		WHO Chinese National Influenza Center	
EPI162940	HA	Indonesia	2008-Jan-16	A/Indonesia/7261/2008	Ministry of Health, NIHRD	Eijkman Institute for Molecular Biology	
EPI586493	HA	Canada	2014-Dec-05	A/poultry/BC/FAV15/2014	Animal Health Centre, Ministry of Agriculture	Canadian Food Inspection Agency	
EPI573638	HA	Japan	2014-Dec-07	A/crane/Kagoshima/KU13/2014(H5N8)	Kagoshima University	Kagoshima University	
EPI464922	HA	Italy	2010-Jan-12	A/guinea fowl/Italy/10VIR-218-134/2010	Istituto Zooprofilattico Sperimentale Delle Venezie	Istituto Zooprofilattico Sperimentale Delle Venezie	Monne, I.; Salviato, A.; Tassoni, L.; Cattoli, G.
EPI448231	HA	Bangladesh	2011-Jun-15	A/chicken/Bangladesh/11RS-1984-30/2011		Centers for Disease Control and Prevention	Gerloff, Nancy; Simpson, Natosha; Poh, Mee;Davis, Todd
EPI448175	HA	Bangladesh	2011-May-23	A/chicken/Bangladesh/3072/2011	Institute of Epidemiology Disease Control and Research (IEDCR) & Bangladesh National Influenza Centre (NIC)	Centers for Disease Control and Prevention	Gerloff, Nancy; Simpson, Natosha; Poh, Mee;Davis, Todd
EPI448143	HA	Bangladesh	2011-Feb-04	A/chicken/Bangladesh/11303/2011	Institute of Epidemiology Disease Control and Research (IEDCR) & Bangladesh National Influenza Centre (NIC)	Centers for Disease Control and Prevention	Gerloff, Nancy; Simpson, Natosha; Poh, Mee;Davis, Todd
EPI448127	HA	Bangladesh	2011-Feb-20	A/chicken/Bangladesh/31289-1/2011	Institute of Epidemiology Disease Control and Research (IEDCR) & Bangladesh National Influenza Centre (NIC)	Centers for Disease Control and Prevention	Gerloff, Nancy; Simpson, Natosha; Poh, Mee;Davis, Todd
EPI448103	HA	Bangladesh	2011-Mar-20	A/duck/Bangladesh/1849/2011	Institute of Epidemiology Disease Control and Research (IEDCR) & Bangladesh National Influenza Centre (NIC)	Centers for Disease Control and Prevention	Gerloff, Nancy; Simpson, Natosha; Poh, Mee;Davis, Todd
EPI448087	HA	Bangladesh	2010-Jan-12	A/chicken/Bangladesh/0411/2010	Institute of Epidemiology Disease Control and Research (IEDCR) & Bangladesh National Influenza Centre (NIC)	Centers for Disease Control and Prevention	Gerloff, Nancy; Simpson, Natosha; Poh, Mee; Davis, Todd
EPI448079	HA	Bangladesh	2010-Jan-04	A/chicken/Bangladesh/1012/2010	Institute of Epidemiology Disease Control and Research (IEDCR) & Bangladesh National Influenza Centre (NIC)	Centers for Disease Control and Prevention	Gerloff, Nancy; Simpson, Natosha; Poh, Mee;Davis, Todd
EPI448071	HA	Bangladesh	2010-Jan-04	A/chicken/Bangladesh/0912/2010	Institute of Epidemiology Disease Control and Research (IEDCR) & Bangladesh National Influenza Centre (NIC)	Centers for Disease Control and Prevention	Gerloff, Nancy; Simpson, Natosha; Poh, Mee;Davis, Todd
EPI425923	HA	Vietnam	2012-Jul-16	A/duck/Vietnam/NCVD-1547/2012	National Centre of Veterinary Diagnostics	Centers for Disease Control and Prevention	
EPI425883	HA	Vietnam	2012-Feb-01	A/duck/Vietnam/NCVD-1182/2012	National Centre of Veterinary Diagnostics	Centers for Disease Control and Prevention	
EPI425851	HA	Vietnam	2011-Nov-26	A/duck/Vietnam/NCVD3-31/2011	National Centre of Veterinary Diagnostics	Centers for Disease Control and Prevention	
EPI425843	HA	Vietnam	2011-Nov-26	A/duck/Vietnam/NCVD3-8/2011	National Centre of Veterinary Diagnostics	Centers for Disease Control and Prevention	
EPI425827	HA	Vietnam	2011-Sep-08	A/chicken/Vietnam/NCVD-1059/2011	National Centre of Veterinary Diagnostics	Centers for Disease Control and Prevention	
EPI425811	HA	Vietnam	2011-Sep-07	A/duck/Vietnam/NCVD-1048/2011	National Centre of Veterinary Diagnostics	Centers for Disease Control and Prevention	
EPI425803	HA	Vietnam	2011-Sep-07	A/duck/Vietnam/NCVD-1044/2011	National Centre of Veterinary Diagnostics	Centers for Disease Control and Prevention	
EPI425787	HA	Vietnam	2011-Sep-07	A/chicken/Vietnam/NCVD-1040/2011	National Centre of Veterinary Diagnostics	Centers for Disease Control and Prevention	
EPI425779	HA	Vietnam	2011-Sep-07	A/chicken/Vietnam/NCVD-1034/2011	National Centre of Veterinary Diagnostics	Centers for Disease Control and Prevention	
EPI425664	HA	Vietnam	2012-Feb-04	A/chicken/Vietnam/NCVD-1188/2012	National Centre of Veterinary Diagnostics	Centers for Disease Control and Prevention	
EPI425648	HA	Vietnam	2012-Aug-24	A/duck/Vietnam/NCVD-1936/2012	National Centre of Veterinary Diagnostics	Centers for Disease Control and Prevention	
EPI425632	HA	Vietnam	2012-Aug-16	A/duck/Vietnam/NCVD-1928/2012	National Centre of Veterinary Diagnostics	Centers for Disease Control and Prevention	
EPI425576	HA	Vietnam	2011-Jun-15	A/chicken/Vietnam/NCVD-879/2011	National Centre of Veterinary Diagnostics	Centers for Disease Control and Prevention	
EPI425568	HA	Vietnam	2011-Mar-17	A/chicken/Vietnam/NCVD-857/2011	National Centre of Veterinary Diagnostics	Centers for Disease Control and Prevention	
EPI425528	HA	Vietnam	2011-Mar-19	A/chicken/Vietnam/NCVD-802/2011	National Centre of Veterinary Diagnostics	Centers for Disease Control and Prevention	
EPI425512	HA	Vietnam	2011-Feb-25	A/chicken/Vietnam/NCVD-777/2011	National Centre of Veterinary Diagnostics	Centers for Disease Control and Prevention	
EPI425504	HA	Vietnam	2011-Mar-18	A/duck/Vietnam/NCVD-946/2011	National Centre of Veterinary Diagnostics	Centers for Disease Control and Prevention	
EPI425488	HA	Vietnam	2011-Jul-19	A/duck/Vietnam/NCVD-927/2011	National Centre of Veterinary Diagnostics	Centers for Disease Control and Prevention	
EPI425464	HA	Vietnam	2012-Feb-20	A/duck/Vietnam/NCVD-1280/2012	National Centre of Veterinary Diagnostics	Centers for Disease Control and Prevention	
EPI425400	HA	Vietnam	2012-Feb-18	A/muscovy duck/Vietnam/NCVD-1256/2012	National Centre of Veterinary Diagnostics	Centers for Disease Control and Prevention	
EPI425352	HA	Vietnam	2012-Feb-10	A/chicken/Vietnam/NCVD-1223/2012	National Centre of Veterinary Diagnostics	Centers for Disease Control and Prevention	
EPI425344	HA	Vietnam	2011-Apr-04	A/chicken/Vietnam/NCVD-753/2011	National Centre of Veterinary Diagnostics	Centers for Disease Control and Prevention	
EPI425328	HA	Vietnam	2012-Feb-08	A/muscovy duck/Vietnam/NCVD-1220/2012	National Centre of Veterinary Diagnostics	Centers for Disease Control and Prevention	
EPI425312	HA	Vietnam	2012-Aug-30	A/duck/Vietnam/NCVD-1904/2012	National Centre of Veterinary Diagnostics	Centers for Disease Control and Prevention	
EPI425288	HA	Vietnam	2011-Jan-01	A/duck/Vietnam/NCVD-674/2011	National Centre of Veterinary Diagnostics	Centers for Disease Control and Prevention	
EPI425280	HA	Vietnam	2011-Jan-01	A/duck/Vietnam/NCVD-673/2011	National Centre of Veterinary Diagnostics	Centers for Disease Control and Prevention	
EPI425272	HA	Vietnam	2012-Aug-27	A/duck/Vietnam/NCVD-1901/2012	National Centre of Veterinary Diagnostics	Centers for Disease Control and Prevention	
EPI425264	HA	Vietnam	2012-Jan-06	A/chicken/Vietnam/NCVD-1224/2012	National Centre of Veterinary Diagnostics	Centers for Disease Control and Prevention	
EPI425256	HA	Vietnam	2012-Jan-30	A/dove/Vietnam/NCVD-1178/2012	National Centre of Veterinary Diagnostics	Centers for Disease Control and Prevention	
EPI425208	HA	Vietnam	2012-Feb-04	A/chicken/Vietnam/NCVD-1192/2012	National Centre of Veterinary Diagnostics	Centers for Disease Control and Prevention	
EPI425200	HA	Vietnam	2011-Nov-22	A/duck/Vietnam/NCVD129-7/2011	National Centre of Veterinary Diagnostics	Centers for Disease Control and Prevention	

EPI425192	HA	Vietnam	2012-Feb-04	A/chicken/Vietnam/NCVD-1189/2012	National Centre of Veterinary Diagnostics	Centers for Disease Control and Prevention	
EPI425168	HA	Vietnam	2011-Mar-17	A/chicken/Vietnam/NCVD-854/2011	National Centre of Veterinary Diagnostics	Centers for Disease Control and Prevention	
EPI425120	HA	Vietnam	2012-Feb-20	A/duck/Vietnam/NCVD-1279/2012	National Centre of Veterinary Diagnostics	Centers for Disease Control and Prevention	
EPI425112	HA	Vietnam	2012-Sep-06	A/duck/Vietnam/NCVD-1943/2012	National Centre of Veterinary Diagnostics	Centers for Disease Control and Prevention	
EPI425064	HA	Vietnam	2012-Feb-16	A/duck/Vietnam/NCVD-1252/2011	National Centre of Veterinary Diagnostics	Centers for Disease Control and Prevention	
EPI425056	HA	Vietnam	2011-Mar-25	A/duck/Vietnam/NCVD-760/2011	National Centre of Veterinary Diagnostics	Centers for Disease Control and Prevention	
EPI425048	HA	Vietnam	2011-Mar-25	A/duck/Vietnam/NCVD-759/2011	National Centre of Veterinary Diagnostics	Centers for Disease Control and Prevention	
EPI425040	HA	Vietnam	2011-Mar-25	A/duck/Vietnam/NCVD-758/2011	National Centre of Veterinary Diagnostics	Centers for Disease Control and Prevention	
EPI425032	HA	Vietnam	2011-Apr-04	A/duck/Vietnam/NCVD-754/2011	National Centre of Veterinary Diagnostics	Centers for Disease Control and Prevention	
EPI425000	HA	Vietnam	2011-Feb-26	A/duck/Vietnam/NCVD-714/2011	National Centre of Veterinary Diagnostics	Centers for Disease Control and Prevention	
EPI424936	HA	Vietnam	2011-Apr-04	A/chicken/Vietnam/NCVD-752/2011	National Centre of Veterinary Diagnostics	Centers for Disease Control and Prevention	
EPI424896	HA	Vietnam	2011-Jul-15	A/chicken/Vietnam/NCVD-921/2011	National Centre of Veterinary Diagnostics	Centers for Disease Control and Prevention	
EPI424888	HA	Vietnam	2011-Jul-15	A/duck/Vietnam/NCVD-920/2011	National Centre of Veterinary Diagnostics	Centers for Disease Control and Prevention	
EPI424872	HA	Vietnam	2011-Sep-06	A/duck/Vietnam/NCVD-1026/2011	National Centre of Veterinary Diagnostics	Centers for Disease Control and Prevention	
EPI424824	HA	Vietnam	2011-Mar-28	A/duck/Vietnam/NCVD-806/2011	National Centre of Veterinary Diagnostics	Centers for Disease Control and Prevention	
EPI424816	HA	Vietnam	2011-Feb-26	A/duck/Vietnam/NCVD-712/2011	National Centre of Veterinary Diagnostics	Centers for Disease Control and Prevention	
EPI424768	HA	Vietnam	2011-Mar-12	A/duck/Vietnam/NCVD-851/2011	National Centre of Veterinary Diagnostics	Centers for Disease Control and Prevention	
EPI424760	HA	Vietnam	2012-Feb-20	A/chicken/Vietnam/NCVD-1277/2012	National Centre of Veterinary Diagnostics	Centers for Disease Control and Prevention	
EPI424736	HA	Vietnam	2012-Aug-15	A/chicken/Vietnam/NCVD-1927/2012	National Centre of Veterinary Diagnostics	Centers for Disease Control and Prevention	
EPI424728	HA	Vietnam	2012-Aug-27	A/duck/Vietnam/NCVD-1898/2012	National Centre of Veterinary Diagnostics	Centers for Disease Control and Prevention	
EPI424720	HA	Vietnam	2012-Aug-23	A/duck/Vietnam/NCVD-1897/2012	National Centre of Veterinary Diagnostics	Centers for Disease Control and Prevention	
EPI424672	HA	Vietnam	2012-Aug-29	A/duck/Vietnam/NCVD-1940/2012	National Centre of Veterinary Diagnostics	Centers for Disease Control and Prevention	
EPI424664	HA	Vietnam	2012-Feb-18	A/chicken/Vietnam/NCVD-1255/2012	National Centre of Veterinary Diagnostics	Centers for Disease Control and Prevention	
EPI424426	HA	Vietnam	2011-Feb-01	A/chicken/Vietnam/NCVD-704/2011	National Centre of Veterinary Diagnostics	Centers for Disease Control and Prevention	
EPI424386	HA	Vietnam	2011-Feb-01	A/chicken/Vietnam/NCVD-685/2011	National Centre of Veterinary Diagnostics	Centers for Disease Control and Prevention	
EPI424378	HA	Vietnam	2011-Feb-01	A/duck/Vietnam/NCVD-680/2011	National Centre of Veterinary Diagnostics	Centers for Disease Control and Prevention	
EPI405128	HA	Vietnam	2011-Jan-01	A/chicken/Vietnam/NCVD-675/2011	National Centre of Veterinary Diagnostics	Centers for Disease Control and Prevention	
EPI372267	HA	Egypt	2003-Dec-15	A/shoveler/Egypt/20313-NAMRU3/2003	U.S. Naval Medical Research Unit No.3	Centers for Disease Control and Prevention	Gerloff, Nancy; Simpson, Natosha; Jones, Joyce; Kis, Zoltan; Bahgat, Verina; Soliman,Atef; Elassal, Emad; Ahmed, Lu'ay; Gaynor, Anne; Cornelius, Claire; Davis,Todd
EPI353381	HA	Bangladesh	2011-Feb-07	A/crow/Bangladesh/315T/2011	Institute of Epidemiology Disease Control and Research (IEDCR) & Bangladesh National Influenza Centre (NIC)	Centers for Disease Control and Prevention	Gerloff, Nancy; Simpson, Natosha; Poh, Mee;Davis, Todd
EPI353379	HA	Bangladesh	2011-Jul-17	A/waterfowl/Bangladesh/31935/2011	Institute of Epidemiology Disease Control and Research (IEDCR) & Bangladesh National Influenza Centre (NIC)	Centers for Disease Control and Prevention	Gerloff, Nancy; Simpson, Natosha; Poh, Mee;Davis, Todd
EPI353370	HA	Bangladesh	2011-Jul-24	A/duck/Bangladesh/4117T/2011	Institute of Epidemiology Disease Control and Research (IEDCR) & Bangladesh National Influenza Centre (NIC)	Centers for Disease Control and Prevention	Gerloff, Nancy; Simpson, Natosha; Poh, Mee;Davis, Todd
EPI353364	HA	Bangladesh	2011-Feb-13	A/crow/Bangladesh/1058/2011	Institute of Epidemiology Disease Control and Research (IEDCR) & Bangladesh National Influenza Centre (NIC)	Centers for Disease Control and Prevention	Gerloff, Nancy; Simpson, Natosha; Poh, Mee;Davis, Todd
EPI347299	HA	Vietnam	2010-Nov-22	A/duck/Vietnam/NCVD-461/2010	National Institute of Hygiene and Epidemiology	Centers for Disease Control and Prevention	Davis,Todd; Rivailler,Pierre; Nguyen,Tung
EPI347298	HA	Vietnam	2010-Nov-22	A/duck/Vietnam/NCVD-460/2010	National Institute of Hygiene and Epidemiology	Centers for Disease Control and Prevention	Davis,Todd; Rivailler,Pierre; Nguyen,Tung
EPI347290	HA	Vietnam	2010-Feb-25	A/chicken/Vietnam/NCVD-428/2010	National Institute of Hygiene and Epidemiology	Centers for Disease Control and Prevention	Davis,Todd; Rivailler,Pierre; Nguyen,Tung
EPI347289	HA	Vietnam	2010-Feb-23	A/chicken/Vietnam/NCVD-427/2010	National Institute of Hygiene and Epidemiology	Centers for Disease Control and Prevention	Davis,Todd; Rivailler,Pierre; Nguyen,Tung
EPI347288	HA	Vietnam	2009-Oct-11	A/muscovy duck/Vietnam/NCVD-426/2009	National Institute of Hygiene and Epidemiology	Centers for Disease Control and Prevention	Davis,Todd; Rivailler,Pierre; Nguyen,Tung
EPI330998	HA	Vietnam	2010-Dec-01	A/chicken/Vietnam/NCVD-668/2010	National Centre of Veterinary Diagnostics	Centers for Disease Control and Prevention	Davis,Todd; Rivailler,Pierre; Nguyen,Tung
EPI305615	HA	Turkey	2006-Jan-31	A/Owl/Turkey-Tunceli/09rs2841-102/2006	Istituto Zooprofilattico Sperimentale Delle Venezie	Istituto Zooprofilattico Sperimentale Delle Venezie	Schivo,A.; Valastro,V.; Monne,I.; Coven,F.; Fusaro,A.; Dakman,A.; Akcadag,B.; Salviato,A.; DeBattisti,C.; Capua,I.; Cattoli,G.
EPI305613	HA	Turkey	2006-Jan-27	A/Quail/Turkey-Batman/09rs2841-73/2006	Istituto Zooprofilattico Sperimentale Delle Venezie	Istituto Zooprofilattico Sperimentale Delle Venezie	Schivo,A.; Valastro,V.; Monne,I.; Coven,F.; Fusaro,A.; Dakman,A.; Akcadag,B.; Salviato,A.; DeBattisti,C.; Capua,I.; Cattoli,G.
EPI305609	HA	Turkey	2006-Jan-06	A/Pigeon/Turkey-Bitlis/09rs2841-11/2006	Istituto Zooprofilattico Sperimentale Delle Venezie	Istituto Zooprofilattico Sperimentale Delle Venezie	Schivo,A.; Valastro,V.; Monne,I.; Coven,F.; Fusaro,A.; Dakman,A.; Akcadag,B.; Salviato,A.; DeBattisti,C.; Capua,I.; Cattoli,G.
EPI284592	HA	Vietnam	2008-Dec-01	A/muscovy duck/Vietnam/NCVD-156/2008	National Centre of Veterinary Diagnostics	Centers for Disease Control and Prevention	Davis,Todd; Rivailler,Pierre; Nguyen,Tung
EPI284583	HA	Vietnam	2010-Apr-01	A/muscovy duck/Vietnam/NCVD-401/2010	National Centre of Veterinary Diagnostics	Centers for Disease Control and Prevention	Davis,Todd; Rivailler,Pierre; Nguyen,Tung
EPI284582	HA	Vietnam	2007-Jan-01	A/duck/Vietnam/NCVD-110/2007	National Centre of Veterinary Diagnostics	Centers for Disease Control and Prevention	Davis,Todd; Rivailler,Pierre; Nguyen,Tung
EPI284580	HA	Vietnam	2008-Jan-01	A/duck/Vietnam/NCVD-010/2008	National Centre of Veterinary Diagnostics	Centers for Disease Control and Prevention	Davis,Todd; Rivailler,Pierre; Nguyen,Tung
EPI284577	HA	Vietnam	2008-Jan-01	A/duck/Vietnam/NCVD-001/2008	National Centre of Veterinary Diagnostics	Centers for Disease Control and Prevention	Davis,Todd; Rivailler,Pierre; Nguyen,Tung
EPI284576	HA	Vietnam	2007-Jan-01	A/duck/Vietnam/NCVD-107/2007	National Centre of Veterinary Diagnostics	Centers for Disease Control and Prevention	Davis,Todd; Rivailler,Pierre; Nguyen,Tung
EPI284575	HA	Vietnam	2007-Jan-01	A/duck/Vietnam/NCVD-105/2007	National Centre of Veterinary Diagnostics	Centers for Disease Control and Prevention	Davis,Todd; Rivailler,Pierre; Nguyen,Tung
EPI284573	HA	Vietnam	2007-Jan-01	A/duck/Vietnam/NCVD-100/2007	National Centre of Veterinary Diagnostics	Centers for Disease Control and Prevention	Davis,Todd; Rivailler,Pierre; Nguyen,Tung
EPI284572	HA	Vietnam	2007-Jan-01	A/duck/Vietnam/NCVD-94/2007	National Centre of Veterinary Diagnostics	Centers for Disease Control and Prevention	Davis,Todd; Rivailler,Pierre; Nguyen,Tung
EPI284570	HA	Vietnam	2007-Jan-01	A/duck/Vietnam/NCVD-113/2007	National Centre of Veterinary Diagnostics	Centers for Disease Control and Prevention	Davis,Todd; Rivailler,Pierre; Nguyen,Tung
EPI284565	HA	Vietnam	2008-Jan-01	A/duck/Vietnam/NCVD-019/2008	National Centre of Veterinary Diagnostics	Centers for Disease Control and Prevention	Davis,Todd; Rivailler,Pierre; Nguyen,Tung
EPI284564	HA	Vietnam	2008-Jan-01	A/duck/Vietnam/NCVD-003/2008	National Centre of Veterinary Diagnostics	Centers for Disease Control and Prevention	Davis,Todd; Rivailler,Pierre; Nguyen,Tung
EPI284563	HA	Vietnam	2007-Jan-01	A/duck/Vietnam/NCVD-111/2007	National Centre of Veterinary Diagnostics	Centers for Disease Control and Prevention	Davis,Todd; Rivailler,Pierre; Nguyen,Tung
EPI284561	HA	Vietnam	2008-Jan-01	A/duck/Vietnam/NCVD-030/2008	National Centre of Veterinary Diagnostics	Centers for Disease Control and Prevention	Davis,Todd; Rivailler,Pierre; Nguyen,Tung
EPI284559	HA	Vietnam	2007-Jan-01	A/duck/Vietnam/NCVD-98/2007	National Centre of Veterinary Diagnostics	Centers for Disease Control and Prevention	Davis,Todd; Rivailler,Pierre; Nguyen,Tung
EPI284558	HA	Vietnam	2008-Jan-01	A/duck/Vietnam/NCVD-014/2008	National Centre of Veterinary Diagnostics	Centers for Disease Control and Prevention	Davis,Todd; Rivailler,Pierre; Nguyen,Tung
EPI284557	HA	Vietnam	2008-Jan-01	A/duck/Vietnam/NCVD-013/2008	National Centre of Veterinary Diagnostics	Centers for Disease Control and Prevention	Davis,Todd; Rivailler,Pierre; Nguyen,Tung
EPI284556	HA	Vietnam	2007-Jan-01	A/duck/Vietnam/NCVD-118/2007	National Centre of Veterinary Diagnostics	Centers for Disease Control and Prevention	Davis,Todd; Rivailler,Pierre; Nguyen,Tung
EPI284555	HA	Vietnam	2008-Jan-01	A/duck/Vietnam/NCVD-028/2008	National Centre of Veterinary Diagnostics	Centers for Disease Control and Prevention	Davis,Todd; Rivailler,Pierre; Nguyen,Tung
EPI284554	HA	Vietnam	2008-Jan-01	A/duck/Vietnam/NCVD-007/2008	National Centre of Veterinary Diagnostics	Centers for Disease Control and Prevention	Davis,Todd; Rivailler,Pierre; Nguyen,Tung
EPI284549	HA	Vietnam	2010-Mar-01	A/duck/Vietnam/NCVD-422/2010	National Centre of Veterinary Diagnostics	Centers for Disease Control and Prevention	Davis,Todd; Rivailler,Pierre; Nguyen,Tung
EPI284548	HA	Vietnam	2010-Mar-01	A/duck/Vietnam/NCVD-423/2010	National Centre of Veterinary Diagnostics	Centers for Disease Control and Prevention	Davis,Todd; Rivailler,Pierre; Nguyen,Tung
EPI284547	HA	Vietnam	2009-Jun-01	A/duck/Vietnam/NCVD-287/2009	National Centre of Veterinary Diagnostics	Centers for Disease Control and Prevention	Davis,Todd; Rivailler,Pierre; Nguyen,Tung
EPI284546	HA	Vietnam	2009-Jul-01	A/duck/Vietnam/NCVD-343/2009	National Centre of Veterinary Diagnostics	Centers for Disease Control and Prevention	Davis,Todd; Rivailler,Pierre; Nguyen,Tung
EPI284540	HA	Vietnam	2008-Dec-01	A/duck/Vietnam/NCVD-145/2008	National Centre of Veterinary Diagnostics	Centers for Disease Control and Prevention	Davis,Todd; Rivailler,Pierre; Nguyen,Tung
EPI284539	HA	Vietnam	2008-Dec-01	A/duck/Vietnam/NCVD-142/2008	National Centre of Veterinary Diagnostics	Centers for Disease Control and Prevention	Davis,Todd; Rivailler,Pierre; Nguyen,Tung
EPI284538	HA	Vietnam	2008-Dec-01	A/duck/Vietnam/NCVD-141/2008	National Centre of Veterinary Diagnostics	Centers for Disease Control and Prevention	Davis,Todd; Rivailler,Pierre; Nguyen,Tung
EPI284536	HA	Vietnam	2008-Dec-01	A/duck/Vietnam/NCVD-118/2008	National Centre of Veterinary Diagnostics	Centers for Disease Control and Prevention	Davis,Todd; Rivailler,Pierre; Nguyen,Tung
EPI284533	HA	Vietnam	2009-Jan-01	A/duck/Vietnam/NCVD-378/2009	National Centre of Veterinary Diagnostics	Centers for Disease Control and Prevention	Davis,Todd; Rivailler,Pierre; Nguyen,Tung
EPI284531	HA	Vietnam	2009-Jan-01	A/duck/Vietnam/NCVD-391/2009	National Centre of Veterinary Diagnostics	Centers for Disease Control and Prevention	Davis,Todd; Rivailler,Pierre; Nguyen,Tung
EPI284530	HA	Vietnam	2009-Jan-01	A/duck/Vietnam/NCVD-365/2009	National Centre of Veterinary Diagnostics	Centers for Disease Control and Prevention	Davis,Todd; Rivailler,Pierre; Nguyen,Tung
EPI284529	HA	Vietnam	2009-Jan-01	A/duck/Vietnam/NCVD-364/2009	National Centre of Veterinary Diagnostics	Centers for Disease Control and Prevention	Davis,Todd; Rivailler,Pierre; Nguyen,Tung
EPI284528	HA	Vietnam	2009-Jan-01	A/duck/Vietnam/NCVD-363/2009	National Centre of Veterinary Diagnostics	Centers for Disease Control and Prevention	Davis,Todd; Rivailler,Pierre; Nguyen,Tung

EPI284525	HA	Vietnam	2009-Jan-01	A/duck/Vietnam/NCVD-376/2009	National Centre of Veterinary Diagnostics	Centers for Disease Control and Prevention	Davis,Todd; Rivailler,Pierre; Nguyen,Tung
EPI284521	HA	Vietnam	2009-Jan-01	A/duck/Vietnam/NCVD-366/2009	National Centre of Veterinary Diagnostics	Centers for Disease Control and Prevention	Davis,Todd; Rivailler,Pierre; Nguyen,Tung
EPI284520	HA	Vietnam	2009-Jan-01	A/duck/Vietnam/NCVD-361/2009	National Centre of Veterinary Diagnostics	Centers for Disease Control and Prevention	Davis,Todd; Rivailler,Pierre; Nguyen,Tung
EPI284519	HA	Vietnam	2009-Jan-01	A/duck/Vietnam/NCVD-360/2009	National Centre of Veterinary Diagnostics	Centers for Disease Control and Prevention	Davis,Todd; Rivailler,Pierre; Nguyen,Tung
EPI284515	HA	Vietnam	2007-Jan-01	A/muscovy duck/Vietnam/NCVD-120/2007	National Centre of Veterinary Diagnostics	Centers for Disease Control and Prevention	Davis,Todd; Rivailler,Pierre; Nguyen,Tung
EPI284513	HA	Vietnam	2007-Jan-01	A/muscovy duck/Vietnam/NCVD-114/2007	National Centre of Veterinary Diagnostics	Centers for Disease Control and Prevention	Davis,Todd; Rivailler,Pierre; Nguyen,Tung
EPI284509	HA	Vietnam	2008-Jan-01	A/muscovy duck/Vietnam/NCVD-034/2008	National Centre of Veterinary Diagnostics	Centers for Disease Control and Prevention	Davis,Todd; Rivailler,Pierre; Nguyen,Tung
EPI284507	HA	Vietnam	2008-May-01	A/muscovy duck/Vietnam/NCVD-079/2008	National Centre of Veterinary Diagnostics	Centers for Disease Control and Prevention	Davis,Todd; Rivailler,Pierre; Nguyen,Tung
EPI284506	HA	Vietnam	2009-Jan-01	A/muscovy duck/Vietnam/NCVD-388/2009	National Centre of Veterinary Diagnostics	Centers for Disease Control and Prevention	Davis,Todd; Rivailler,Pierre; Nguyen,Tung
EPI284504	HA	Vietnam	2009-Jan-01	A/muscovy duck/Vietnam/NCVD-393/2009	National Centre of Veterinary Diagnostics	Centers for Disease Control and Prevention	Davis,Todd; Rivailler,Pierre; Nguyen,Tung
EPI284503	HA	Vietnam	2008-Dec-01	A/muscovy duck/Vietnam/NCVD-155/2008	National Centre of Veterinary Diagnostics	Centers for Disease Control and Prevention	Davis,Todd; Rivailler,Pierre; Nguyen,Tung
EPI284500	HA	Vietnam	2010-Apr-01	A/muscovy duck/Vietnam/NCVD-402/2010	National Centre of Veterinary Diagnostics	Centers for Disease Control and Prevention	Davis,Todd; Rivailler,Pierre; Nguyen,Tung
EPI284497	HA	Vietnam	2008-Jan-01	A/chicken/Vietnam/NCVD-039/2008	National Centre of Veterinary Diagnostics	Centers for Disease Control and Prevention	Davis,Todd; Rivailler,Pierre; Nguyen,Tung
EPI284494	HA	Vietnam	2007-Jan-01	A/chicken/Vietnam/NCVD-108/2007	National Centre of Veterinary Diagnostics	Centers for Disease Control and Prevention	Davis,Todd; Rivailler,Pierre; Nguyen,Tung
EPI284493	HA	Vietnam	2007-Jan-01	A/chicken/Vietnam/NCVD-106/2007	National Centre of Veterinary Diagnostics	Centers for Disease Control and Prevention	Davis,Todd; Rivailler,Pierre; Nguyen,Tung
EPI284491	HA	Vietnam	2007-Jan-01	A/duck/Vietnam/NCVD-126/2007	National Centre of Veterinary Diagnostics	Centers for Disease Control and Prevention	Davis,Todd; Rivailler,Pierre; Nguyen,Tung
EPI284490	HA	Vietnam	2008-Jan-01	A/chicken/Vietnam/NCVD-002/2008	National Centre of Veterinary Diagnostics	Centers for Disease Control and Prevention	Davis,Todd; Rivailler,Pierre; Nguyen,Tung
EPI284488	HA	Vietnam	2008-Jan-01	A/chicken/Vietnam/NCVD-025/2008	National Centre of Veterinary Diagnostics	Centers for Disease Control and Prevention	Davis,Todd; Rivailler,Pierre; Nguyen,Tung
EPI284485	HA	Vietnam	2007-Jan-01	A/chicken/Vietnam/NCVD-109/2007	National Centre of Veterinary Diagnostics	Centers for Disease Control and Prevention	Davis,Todd; Rivailler,Pierre; Nguyen,Tung
EPI284482	HA	Vietnam	2008-May-01	A/chicken/Vietnam/NCVD-086/2008	National Centre of Veterinary Diagnostics	Centers for Disease Control and Prevention	Davis,Todd; Rivailler,Pierre; Nguyen,Tung
EPI284480	HA	Vietnam	2008-May-01	A/chicken/Vietnam/NCVD-084/2008	National Centre of Veterinary Diagnostics	Centers for Disease Control and Prevention	Davis,Todd; Rivailler,Pierre; Nguyen,Tung
EPI284477	HA	Vietnam	2008-May-01	A/chicken/Vietnam/NCVD-080/2008	National Centre of Veterinary Diagnostics	Centers for Disease Control and Prevention	Davis,Todd; Rivailler,Pierre; Nguyen,Tung
EPI284476	HA	Vietnam	2008-May-01	A/chicken/Vietnam/NCVD-093/2008	National Centre of Veterinary Diagnostics	Centers for Disease Control and Prevention	Davis,Todd; Rivailler,Pierre; Nguyen,Tung
EPI284475	HA	Vietnam	2008-May-01	A/chicken/Vietnam/NCVD-05/2008	National Centre of Veterinary Diagnostics	Centers for Disease Control and Prevention	Davis,Todd; Rivailler,Pierre; Nguyen,Tung
EPI284474	HA	Vietnam	2008-May-01	A/chicken/Vietnam/NCVD-04/2008	National Centre of Veterinary Diagnostics	Centers for Disease Control and Prevention	Davis,Todd; Rivailler,Pierre; Nguyen,Tung
EPI284472	HA	Vietnam	2009-Mar-01	A/chicken/Vietnam/NCVD-296/2009	National Centre of Veterinary Diagnostics	Centers for Disease Control and Prevention	Davis,Todd; Rivailler,Pierre; Nguyen,Tung
EPI284469	HA	Vietnam	2010-Mar-01	A/chicken/Vietnam/NCVD-399/2010	National Centre of Veterinary Diagnostics	Centers for Disease Control and Prevention	Davis,Todd; Rivailler,Pierre; Nguyen,Tung
EPI284468	HA	Vietnam	2010-Mar-01	A/chicken/Vietnam/NCVD-398/2010	National Centre of Veterinary Diagnostics	Centers for Disease Control and Prevention	Davis,Todd; Rivailler,Pierre; Nguyen,Tung
EPI284466	HA	Vietnam	2009-Jan-01	A/chicken/Vietnam/NCVD-279/2009	National Centre of Veterinary Diagnostics	Centers for Disease Control and Prevention	Davis,Todd; Rivailler,Pierre; Nguyen,Tung
EPI284457	HA	Vietnam	2009-Feb-01	A/chicken/Vietnam/NCVD-288/2009	National Centre of Veterinary Diagnostics	Centers for Disease Control and Prevention	Davis,Todd; Rivailler,Pierre; Nguyen,Tung
EPI284456	HA	Vietnam	2009-Feb-01	A/chicken/Vietnam/NCVD-283/2009	National Centre of Veterinary Diagnostics	Centers for Disease Control and Prevention	Davis,Todd; Rivailler,Pierre; Nguyen,Tung
EPI284453	HA	Vietnam	2008-Dec-01	A/chicken/Vietnam/NCVD-swab276/2008	National Centre of Veterinary Diagnostics	Centers for Disease Control and Prevention	Davis,Todd; Rivailler,Pierre; Nguyen,Tung
EPI284447	HA	Vietnam	2010-Apr-01	A/chicken/Vietnam/NCVD-410/2010	National Centre of Veterinary Diagnostics	Centers for Disease Control and Prevention	Davis,Todd; Rivailler,Pierre; Nguyen,Tung
EPI284444	HA	Vietnam	2010-Apr-01	A/chicken/Vietnam/NCVD-406/2010	National Centre of Veterinary Diagnostics	Centers for Disease Control and Prevention	Davis,Todd; Rivailler,Pierre; Nguyen,Tung
EPI284443	HA	Vietnam	2010-Apr-01	A/chicken/Vietnam/NCVD-405/2010	National Centre of Veterinary Diagnostics	Centers for Disease Control and Prevention	Davis,Todd; Rivailler,Pierre; Nguyen,Tung
EPI284441	HA	Vietnam	2009-Jan-01	A/chicken/Vietnam/NCVD-359/2009	National Centre of Veterinary Diagnostics	Centers for Disease Control and Prevention	Davis,Todd; Rivailler,Pierre; Nguyen,Tung
EPI284433	HA	Vietnam	2009-Jan-01	A/chicken/Vietnam/NCVD-369/2009	National Centre of Veterinary Diagnostics	Centers for Disease Control and Prevention	Davis,Todd; Rivailler,Pierre; Nguyen,Tung
EPI594490	HA	Bulgaria	2015-Jan-30	A/chicken/Bulgaria/5407/15	NDRVMI (National Diagnostic and Research Veterinary Medical Institute)	Animal and Plant Health Agency (APHA)	Thomas, S; Seekings, A; Essen, S; Manvell, R; Goujgoulova, G; Oreshkova, L; Banks, J; Brown, I
EPI588968	HA	Taiwan	2015-Jan-16	A/chicken/Taiwan/a174/2015	ANIMAL HEALTH RESEARCH INSTITUTE	Animal Health Research Institute	Yu-Ju,Lin; Li-Hsuan,Chen; Wan-Chen,Li; Yu-Pin,Liu; Ming-Shiuh,Lee; Ming-Chu,Cheng; Hsiang-Jung,Tsai
EPI584232	HA	Burkina Faso	2015-Mar-23	A/chicken/Burkina Faso/15VIR1774-35/2015	Services Vétérinaires	Istituto Zooprofilattico Sperimentale Delle Venezie	Ouattara, L.; Minoungou, G.L.; Ormelli, S.; Schivo, A.; Fusaro, A.; Monne, I.; Cattoli, G.
EPI573334	HA	Egypt	2014-Nov-03	A/chicken/Egypt/141/2014		Friedrich-Loeffler-Institut	Arafa,A.M; Hagag, N; Elhusseiny, M.H; Yehia, N; Selim, A.A; Abdelhalim, A; Kilany, W.H; Samy, A; Hassan, M.K; Abdelwhab,E.M; Beer,M; Naguib, M.M; Harder,TC
EPI573329	HA	Egypt	2014-Dec-05	A/chicken/Egypt/14140CA/2014		Friedrich-Loeffler-Institut	Arafa,A.M; Hagag, N; Elhusseiny, M.H; Yehia, N; Selim, A.A; Abdelhalim, A; Kilany, W.H; Samy, A; Hassan, M.K; Abdelwhab,E.M; Beer,M; Naguib, M.M; Harder,TC
EPI573328	HA	Egypt	2014-Dec-16	A/chicken/Egypt/14168CA/2014		Friedrich-Loeffler-Institut	Arafa,A.M; Hagag, N; Elhusseiny, M.H; Yehia, N; Selim, A.A; Abdelhalim, A; Kilany, W.H; Samy, A; Hassan, M.K; Abdelwhab,E.M; Beer,M; Naguib, M.M; Harder,TC
EPI573319	HA	Egypt	2015-Jan-01	A/chicken/Egypt/152/2015		Friedrich-Loeffler-Institut	Arafa,A.M; Hagag, N; Elhusseiny, M.H; Yehia, N; Selim, A.A; Abdelhalim, A; Kilany, W.H; Samy, A; Hassan, M.K; Abdelwhab,E.M; Beer,M; Naguib, M.M; Harder,TC
EPI573317	HA	Egypt	2015-Jan-21	A/chicken/Egypt/1575S/2015		Friedrich-Loeffler-Institut	Arafa,A.M; Hagag, N; Elhusseiny, M.H; Yehia, N; Selim, A.A; Abdelhalim, A; Kilany, W.H; Samy, A; Hassan, M.K; Abdelwhab,E.M; Beer,M; Naguib, M.M; Harder,TC
EPI573313	HA	Egypt	2015-Jan-14	A/chicken/Egypt/1510CA/2015		Friedrich-Loeffler-Institut	Arafa,A.M; Hagag, N; Elhusseiny, M.H; Yehia, N; Selim, A.A; Abdelhalim, A; Kilany, W.H; Samy, A; Hassan, M.K; Abdelwhab,E.M; Beer,M; Naguib, M.M; Harder,TC
EPI573203	HA	Korea, Republic of	2014-Apr-20	A/chicken/Korea/H1292/2014		Animal and Plant Quarantine Agency	
EPI573200	HA	Korea, Republic of	2014-Mar-21	A/Korean native chicken/Korea/H1139/2014		Animal and Plant Quarantine Agency	
EPI573197	HA	Korea, Republic of	2014-Mar-06	A/chicken/Korea/H881/2014		Animal and Plant Quarantine Agency	
EPI557218	HA	Egypt	2013-Mar-25	A/chicken/Egypt/NLQP139V-AR753/2013	National Laboratory for Veterinary Quality Control on Poultry production- Animal Health Research Inistitute	Friedrich-Loeffler-Institut	Naguib, M.M; Arafa, A.M; Selim,A.A; Hassan,M.K; Beer,M; Harder,TC
EPI557210	HA	Egypt	2013-Apr-14	A/chicken/Egypt/NLQP639V-AR752/2013	National Laboratory for Veterinary Quality Control on Poultry production- Animal Health Research Inistitute	Friedrich-Loeffler-Institut	Naguib, M.M; Arafa, A.M; Selim,A.A; Hassan,M.K; Beer,M; Harder,TC
EPI553223	HA	China	2009-Sep-01	A/Chicken/Shandong/J02/2009	Qingdao Agricultural University	Beijing Institute of Microbiology and Epidemiology	yanbo,yin;dongdong,wang;linlin,liu;xiliang,wang;penghui,yang;xin,liu;keyu,wang;chengcai,lai
EPI553219	HA	China	2009-Sep-01	A/Chicken/Shandong/J01/2009	Qingdao Agricultural University	Beijing Institute of Microbiology and Epidemiology	yanbo,yin;dongdong,wang;linlin,liu;xiliang,wang;penghui,yang;xin,liu;keyu,wang;chengcai,lai
EPI538815	HA	Egypt	2013-May-01	A/chicken/Egypt/13VIR-2962-203/2013	Istituto Zooprofilattico Sperimentale Delle Venezie	Istituto Zooprofilattico Sperimentale Delle Venezie	Hussein, H.A.; El Hady, M.M.; Abd Hamid, H.S.; Sultan, H.A.; Abdel Hafez, A.; Fusaro, A.; Schivo, A.; Ormelli, S.; Monne, I.; Cattoli, G.
EPI538767	HA	Egypt	2013-Mar-01	A/chicken/Egypt/13VIR-2962-109/2013	Istituto Zooprofilattico Sperimentale Delle Venezie	Istituto Zooprofilattico Sperimentale Delle Venezie	Hussein, H.A.; El Hady, M.M.; Abd Hamid, H.S.; Sultan, H.A.; Abdel Hafez, A.; Fusaro, A.; Schivo, A.; Ormelli, S.; Monne, I.; Cattoli, G.
EPI526016	HA	Nepal	2014-Feb-17	A/chicken/Nepal/T-359/2014	Central Veterinary Laboratory	Animal and Plant Health Agency (APHA)	Puranik, A; Collins, S; Thomas, S; Hanna, A; Essen, S; Focosi-Snyman, R; Manvell, R; Jha, V; Chapagai, S; Koirala, P; Air, T; Banks, J
EPI475775	HA	Nepal	2013-Apr-30	A/chicken/Nepal/T-301/13	Central Veterinary Laboratory	Animal and Plant Health Agency (APHA)	Collins, S; Hanna, A; Essen, S; Focosi-Snyman, R; Manvell, R; Jha, VC; Chapagain, S; Koirala, P; Air, TB; Reid, S
EPI475773	HA	Nepal	2013-Apr-30	A/chicken/Nepal/T-299/13	Central Veterinary Laboratory	Animal and Plant Health Agency (APHA)	Puranik, A; Hanna, A; Essen, S; Focosi-Snyman, R; Manvell, R; Jha, VC; Chapagain, S; Koirala, P; Air, TB; Reid, S
EPI475772	HA	Nepal	2013-Apr-23	A/chicken/Nepal/T-279/13	Central Veterinary Laboratory	Animal and Plant Health Agency (APHA)	Collins, S; Hanna, A; Essen, S; Focosi-Snyman, R; Manvell, R; Jha, VC; Chapagain, S; Koirala, P; Air, TB; Reid, S
EPI475770	HA	Nepal	2013-Apr-23	A/chicken/Nepal/T-278/13	Central Veterinary Laboratory	Animal and Plant Health Agency (APHA)	Puranik, A; Hanna, A; Essen, S; Focosi-Snyman, R; Manvell, R; Jha, VC; Chapagain, S; Koirala, P; Air, TB; Reid, S
EPI475768	HA	Nepal	2013-Apr-21	A/chicken/Nepal/T-274/13	Central Veterinary Laboratory	Animal and Plant Health Agency (APHA)	Puranik, A; Hanna, A; Essen, S; Focosi-Snyman, R; Manvell, R; Jha, VC; Chapagain, S; Koirala, P; Air, TB; Reid, S
EPI475766	HA	Nepal	2013-Apr-21	A/chicken/Nepal/T-273/13	Central Veterinary Laboratory	Animal and Plant Health Agency (APHA)	Collins, S; Hanna, A; Essen, S; Focosi-Snyman, R; Manvell, R; Jha, VC; Chapagain, S; Koirala, P; Air, TB; Reid, S

EPI475764	HA	Nepal	2013-Apr-21	A/chicken/Nepal/T-272/13	Central Veterinary Laboratory	Animal and Plant Health Agency (APHA)	Collins, S; Hanna, A; Essen, S; Focosi-Snyman, R; Manvell, R; Jha, VC; Chapagain, S; Koirala, P; Air, TB; Reid, S
EPI475762	HA	Nepal	2013-Apr-21	A/chicken/Nepal/T-271/13	Central Veterinary Laboratory	Animal and Plant Health Agency (APHA)	Collins, S; Hanna, A; Essen, S; Focosi-Snyman, R; Manvell, R; Jha, VC; Chapagain, S; Koirala, P; Air, TB; Reid, S
EPI464957	HA	Italy	2012-Sep-18	A/chicken/Italy/12VIR-7257/2012	Istituto Zooprofilattico Sperimentale Delle Venezie	Istituto Zooprofilattico Sperimentale Delle Venezie	Monne, I.; Salviato, A.; Tassoni, L.; Cattoli, G.
EPI464946	HA	Italy	2012-Sep-28	A/chicken/Italy/12VIR-7785-67/2012	Istituto Zooprofilattico Sperimentale Delle Venezie	Istituto Zooprofilattico Sperimentale Delle Venezie	Monne, I.; Salviato, A.; Tassoni, L.; Cattoli, G.
EPI446008	HA	Nepal	2013-Mar-28	A/chicken/Nepal/225/13	Central Veterinary Laboratory	Animal and Plant Health Agency (APHA)	Puranik, A; Hanna, A; Essen, S; Focosi-Snyman, R; Manvell, R; Manandhar, P; Reid, S
EPI356869	HA	Nepal	2012-Jan-27	A/chicken/Nepal/T1P/12	Central Veterinary Laboratory	Animal and Plant Health Agency (APHA)	Puranik, A; Hanna, A; Essen, S; Focosi-Snyman, R; Manvell, R.J; Sedai, D; Chapagain, S; Manandhar, S; Koirala, P; Karki, K.B; Pandey, K.R; Air, T.B; Reid, S
EPI356861	HA	Nepal	2011-Nov-11	A/chicken/Nepal/T9-BH/11	Central Veterinary Laboratory	Animal and Plant Health Agency (APHA)	Puranik, A; Hanna, A; Essen, S; Focosi-Snyman, R; Manvell, R.J; Sedai, D; Chapagain, S; Manandhar, S; Koirala, P; Karki, K.B; Pandey, K.R; Air, T.B; Reid, S
EPI348164	HA	Egypt	2010-Jan-01	A/chicken/Egypt/11VIR4453-18/VRLCU/2010	Istituto Zooprofilattico Sperimentale Delle Venezie	Istituto Zooprofilattico Sperimentale Delle Venezie	Valastro, V.; Fusaro, A.; Monne, I.; Hussein, H.A.; Rohiam, M.; El Sanousi, A.A.; Abdel hamid, H.S.; Sultan, H.A.; Adel hafez, A.; Sedik, M.; Cattoli, G.
EPI348162	HA	Egypt	2010-Jan-01	A/chicken/Egypt/11VIR4453-7/VRLCU/2010	Istituto Zooprofilattico Sperimentale Delle Venezie	Istituto Zooprofilattico Sperimentale Delle Venezie	Valastro, V.; Fusaro, A.; Monne, I.; Hussein, H.A.; Rohiam, M.; El Sanousi, A.A.; Abdel hamid, H.S.; Sultan, H.A.; Adel hafez, A.; Sedik, M.; Cattoli, G.
EPI348159	HA	Egypt	2011-Jan-01	A/chicken/Egypt/11VIR4453-137/VRLCU/2011	Istituto Zooprofilattico Sperimentale Delle Venezie	Istituto Zooprofilattico Sperimentale Delle Venezie	Valastro, V.; Fusaro, A.; Monne, I.; Hussein, H.A.; Rohiam, M.; El Sanousi, A.A.; Abdel hamid, H.S.; Sultan, H.A.; Adel hafez, A.; Sedik, M.; Cattoli, G.
EPI348156	HA	Egypt	2010-Jan-01	A/chicken/Egypt11VIR4453-269/2010	Istituto Zooprofilattico Sperimentale Delle Venezie	Istituto Zooprofilattico Sperimentale Delle Venezie	Valastro, V.; Fusaro, A.; Monne, I.; Hussein, H.A.; Rohiam, M.; El Sanousi, A.A.; Abdel hamid, H.S.; Sultan, H.A.; Adel hafez, A.; Sedik, M.; Cattoli, G.
EPI348154	HA	Egypt	2010-Jan-01	A/chicken/Egypt/11VIR4453-264/2010	Istituto Zooprofilattico Sperimentale Delle Venezie	Istituto Zooprofilattico Sperimentale Delle Venezie	Valastro, V.; Fusaro, A.; Monne, I.; Hussein, H.A.; Rohiam, M.; El Sanousi, A.A.; Abdel hamid, H.S.; Sultan, H.A.; Adel hafez, A.; Sedik, M.; Cattoli, G.
EPI348153	HA	Egypt	2010-Jan-01	A/chicken/Egypt/11VIR4453-266/2010	Istituto Zooprofilattico Sperimentale Delle Venezie	Istituto Zooprofilattico Sperimentale Delle Venezie	Valastro, V.; Fusaro, A.; Monne, I.; Hussein, H.A.; Rohiam, M.; El Sanousi, A.A.; Abdel hamid, H.S.; Sultan, H.A.; Adel hafez, A.; Sedik, M.; Cattoli, G.
EPI348148	HA	Egypt	2011-Jan-01	A/chicken/Egypt/11VIR4453-59/VRLCU/2011	Istituto Zooprofilattico Sperimentale Delle Venezie	Istituto Zooprofilattico Sperimentale Delle Venezie	Valastro, V.; Fusaro, A.; Monne, I.; Hussein, H.A.; Rohiam, M.; El Sanousi, A.A.; Abdel hamid, H.S.; Sultan, H.A.; Adel hafez, A.; Sedik, M.; Cattoli, G.
EPI340810	HA	Indonesia	2010-Jan-01	A/chicken/Lampung/BPPVRIII-10-161/2010	Disease Investigation Centre Regional III Bandar Lampung	CSIRO Australian Animal Health Laboratory	Suryantana,.; Ma'arif,Syamsul; Suatmodjo,Musny; Bruce,Kerri; Davies,Kelly R; Stevens,Vittoria; Kim,Mia; Daniels,Peter; Wong,Frank YK
EPI340806	HA	Indonesia	2009-Jan-01	A/chicken/Tanah Datar/BPPVRII-770/2009	Disease Investigation Centre Regional II Bukittinggi (BPPVRII)	CSIRO Australian Animal Health Laboratory	Miswati,Yuli; Yulfitria,.; Azfirman,.; Suatmodjo,Musny; Bruce,Kerri; Davies,Kelly R; Stevens,Vittoria; Kim,Mia; Daniels,Peter; Wong,Frank YK
EPI340794	HA	Indonesia	2010-Jan-01	A/chicken/Banjarbaru/BPPVRV-26/2010	Disease Investigation Centre Regional V Banjarbaru (BPPVRV)	CSIRO Australian Animal Health Laboratory	Utami,Wiwin Sri; Hadi,Sulaxono; Suatmodjo,Musny; Bruce,Kerri; Davies,Kelly R; Stevens,Vittoria; Kim,Mia; Daniels,Peter; Wong,Frank YK
EPI340792	HA	Indonesia	2009-Jan-01	A/chicken/Kapuas/BPPVRV-342/2009	Disease Investigation Centre Regional V Banjarbaru (BPPVRV)	CSIRO Australian Animal Health Laboratory	Utami,Wiwin Sri; Hadi,Sulaxono; Suatmodjo,Musny; Bruce,Kerri; Davies,Kelly R; Stevens,Vittoria; Kim,Mia; Daniels,Peter; Wong,Frank YK
EPI340778	HA	Indonesia	2009-Jan-01	A/chicken/Temanggung/BBVW-1203d-VIII/2009	Disease Investigation Centre Wates (BBVW)	CSIRO Australian Animal Health Laboratory	Dhamawan,Rama; Usman,Tri Bhakti; Junaidi,Akhmad; Suatmodjo,Musny; Bruce,Kerri; Davies,Kelly R; Stevens,Vittoria; Kim,Mia; Daniels,Peter; Wong,Frank YK
EPI340776	HA	Indonesia	2009-Jan-01	A/chicken/Temanggung/BBVW-1203b-VIII/2009	Disease Investigation Centre Wates (BBVW)	CSIRO Australian Animal Health Laboratory	Dhamawan,Rama; Usman,Tri Bhakti; Junaidi,Akhmad; Suatmodjo,Musny; Bruce,Kerri; Davies,Kelly R; Stevens,Vittoria; Kim,Mia; Daniels,Peter; Wong,Frank YK
EPI332698	HA	Vietnam	2011-Feb-01	A/chicken/Vietnam/NCVD-700/2011	National Centre of Veterinary Diagnostics	Centers for Disease Control and Prevention	Davis,Todd; Rivailler,Pierre; Nguyen,Tung
EPI330997	HA	Vietnam	2011-Feb-01	A/chicken/Vietnam/NCVD-686/2011	National Centre of Veterinary Diagnostics	Centers for Disease Control and Prevention	Davis,Todd; Rivailler,Pierre; Nguyen,Tung
EPI305608	HA	Turkey	2008-Feb-07	A/Chicken/Turkey-Sakarya/09rs2843-4/2008	Istituto Zooprofilattico Sperimentale Delle Venezie	Istituto Zooprofilattico Sperimentale Delle Venezie	Schivo,A.; Valastro,V.; Monne,I.; Coven,F.; Fusaro,A.; Dakman,A.; Akcadag,B.; Salviato,A.; DeBattisti,C.; Capua,I.; Cattoli,G.
EPI305606	HA	Turkey	2008-Feb-04	A/Chicken/Turkey-Sakarya/09rs2843-2/2008	Istituto Zooprofilattico Sperimentale Delle Venezie	Istituto Zooprofilattico Sperimentale Delle Venezie	Schivo,A.; Valastro,V.; Monne,I.; Coven,F.; Fusaro,A.; Dakman,A.; Akcadag,B.; Salviato,A.; DeBattisti,C.; Capua,I.; Cattoli,G.
EPI305605	HA	Turkey	2008-Feb-15	A/Chicken/Turkey-Samsun/09rs2842-113/08	Istituto Zooprofilattico Sperimentale Delle Venezie	Istituto Zooprofilattico Sperimentale Delle Venezie	Schivo,A.; Valastro,V.; Monne,I.; Coven,F.; Fusaro,A.; Dakman,A.; Akcadag,B.; Salviato,A.; DeBattisti,C.; Capua,I.; Cattoli,G.
EPI305603	HA	Turkey	2008-Jan-15	A/Chicken/Turkey-Zonguldak/09rs2842-111/2008	Istituto Zooprofilattico Sperimentale Delle Venezie	Istituto Zooprofilattico Sperimentale Delle Venezie	Schivo,A.; Valastro,V.; Monne,I.; Coven,F.; Fusaro,A.; Dakman,A.; Akcadag,B.; Salviato,A.; DeBattisti,C.; Capua,I.; Cattoli,G.
EPI305601	HA	Turkey	2007-Feb-15	A/Chicken/Turkey-Batman/09rs2842-109/2007	Istituto Zooprofilattico Sperimentale Delle Venezie	Istituto Zooprofilattico Sperimentale Delle Venezie	Schivo,A.; Valastro,V.; Monne,I.; Coven,F.; Fusaro,A.; Dakman,A.; Akcadag,B.; Salviato,A.; DeBattisti,C.; Capua,I.; Cattoli,G.
EPI305595	HA	Turkey	2007-Feb-15	A/Chicken/Turkey-Diyarbakir/09rs2842-91/2007	Istituto Zooprofilattico Sperimentale Delle Venezie	Istituto Zooprofilattico Sperimentale Delle Venezie	Schivo,A.; Valastro,V.; Monne,I.; Coven,F.; Fusaro,A.; Dakman,A.; Akcadag,B.; Salviato,A.; DeBattisti,C.; Capua,I.; Cattoli,G.
EPI305592	HA	Turkey	2006-Feb-28	A/Chicken/Turkey-Izmir/09rs2841-119/2006	Istituto Zooprofilattico Sperimentale Delle Venezie	Istituto Zooprofilattico Sperimentale Delle Venezie	Schivo,A.; Valastro,V.; Monne,I.; Coven,F.; Fusaro,A.; Dakman,A.; Akcadag,B.; Salviato,A.; DeBattisti,C.; Capua,I.; Cattoli,G.
EPI305587	HA	Turkey	2006-Jan-31	A/Chicken/Turkey-Malatya/09rs2841-108/2006	Istituto Zooprofilattico Sperimentale Delle Venezie	Istituto Zooprofilattico Sperimentale Delle Venezie	Schivo,A.; Valastro,V.; Monne,I.; Coven,F.; Fusaro,A.; Dakman,A.; Akcadag,B.; Salviato,A.; DeBattisti,C.; Capua,I.; Cattoli,G.
EPI305584	HA	Turkey	2006-Feb-21	A/Chicken/Turkey-Siirt/09rs2841-104/2006	Istituto Zooprofilattico Sperimentale Delle Venezie	Istituto Zooprofilattico Sperimentale Delle Venezie	Schivo,A.; Valastro,V.; Monne,I.; Coven,F.; Fusaro,A.; Dakman,A.; Akcadag,B.; Salviato,A.; DeBattisti,C.; Capua,I.; Cattoli,G.
EPI305579	HA	Turkey	2006-Feb-14	A/Chicken/Turkey-Malatya/09rs2841-91/2006	Istituto Zooprofilattico Sperimentale Delle Venezie	Istituto Zooprofilattico Sperimentale Delle Venezie	Schivo,A.; Valastro,V.; Monne,I.; Coven,F.; Fusaro,A.; Dakman,A.; Akcadag,B.; Salviato,A.; DeBattisti,C.; Capua,I.; Cattoli,G.
EPI305578	HA	Turkey	2006-Jan-31	A/Chicken/Turkey-Malatya/09rs2841-90/2006	Istituto Zooprofilattico Sperimentale Delle Venezie	Istituto Zooprofilattico Sperimentale Delle Venezie	Schivo,A.; Valastro,V.; Monne,I.; Coven,F.; Fusaro,A.; Dakman,A.; Akcadag,B.; Salviato,A.; DeBattisti,C.; Capua,I.; Cattoli,G.
EPI305577	HA	Turkey	2006-Feb-14	A/Chicken/Turkey-Malatya/09rs2841-89/2006	Istituto Zooprofilattico Sperimentale Delle Venezie	Istituto Zooprofilattico Sperimentale Delle Venezie	Schivo,A.; Valastro,V.; Monne,I.; Coven,F.; Fusaro,A.; Dakman,A.; Akcadag,B.; Salviato,A.; DeBattisti,C.; Capua,I.; Cattoli,G.
EPI305572	HA	Turkey	2006-Feb-14	A/Chicken/Turkey-Aksaray/09rs2841-83/2006	Istituto Zooprofilattico Sperimentale Delle Venezie	Istituto Zooprofilattico Sperimentale Delle Venezie	Schivo,A.; Valastro,V.; Monne,I.; Coven,F.; Fusaro,A.; Dakman,A.; Akcadag,B.; Salviato,A.; DeBattisti,C.; Capua,I.; Cattoli,G.
EPI305571	HA	Turkey	2006-Feb-08	A/Chicken/Turkey-Konya/09rs2841-82/2006	Istituto Zooprofilattico Sperimentale Delle Venezie	Istituto Zooprofilattico Sperimentale Delle Venezie	Schivo,A.; Valastro,V.; Monne,I.; Coven,F.; Fusaro,A.; Dakman,A.; Akcadag,B.; Salviato,A.; DeBattisti,C.; Capua,I.; Cattoli,G.
EPI305570	HA	Turkey	2006-Feb-08	A/Chicken/Turkey-Gaziantep/09rs2841-81/2006	Istituto Zooprofilattico Sperimentale Delle Venezie	Istituto Zooprofilattico Sperimentale Delle Venezie	Schivo,A.; Valastro,V.; Monne,I.; Coven,F.; Fusaro,A.; Dakman,A.; Akcadag,B.; Salviato,A.; DeBattisti,C.; Capua,I.; Cattoli,G.
EPI305568	HA	Turkey	2006-Jan-27	A/Chicken/Turkey-Van/09rs2841-79/2006	Istituto Zooprofilattico Sperimentale Delle Venezie	Istituto Zooprofilattico Sperimentale Delle Venezie	Schivo,A.; Valastro,V.; Monne,I.; Coven,F.; Fusaro,A.; Dakman,A.; Akcadag,B.; Salviato,A.; DeBattisti,C.; Capua,I.; Cattoli,G.
EPI305564	HA	Turkey	2006-Jan-26	A/Chicken/Turkey-Diyarbakir/09rs2841-70/2006	Istituto Zooprofilattico Sperimentale Delle Venezie	Istituto Zooprofilattico Sperimentale Delle Venezie	Schivo,A.; Valastro,V.; Monne,I.; Coven,F.; Fusaro,A.; Dakman,A.; Akcadag,B.; Salviato,A.; DeBattisti,C.; Capua,I.; Cattoli,G.
EPI305561	HA	Turkey	2006-Jan-27	A/Chicken/Turkey-Mus/09rs2841-65/2006	Istituto Zooprofilattico Sperimentale Delle Venezie	Istituto Zooprofilattico Sperimentale Delle Venezie	Schivo,A.; Valastro,V.; Monne,I.; Coven,F.; Fusaro,A.; Dakman,A.; Akcadag,B.; Salviato,A.; DeBattisti,C.; Capua,I.; Cattoli,G.
EPI305559	HA	Turkey	2006-Jan-27	A/Chicken/Turkey-Eskisehir/09rs2841-63/2006	Istituto Zooprofilattico Sperimentale Delle Venezie	Istituto Zooprofilattico Sperimentale Delle Venezie	Schivo,A.; Valastro,V.; Monne,I.; Coven,F.; Fusaro,A.; Dakman,A.; Akcadag,B.; Salviato,A.; DeBattisti,C.; Capua,I.; Cattoli,G.
EPI305558	HA	Turkey	2006-Jan-23	A/Chicken/Turkey-Siirt/09rs2841-56/2006	Istituto Zooprofilattico Sperimentale Delle Venezie	Istituto Zooprofilattico Sperimentale Delle Venezie	Schivo,A.; Valastro,V.; Monne,I.; Coven,F.; Fusaro,A.; Dakman,A.; Akcadag,B.; Salviato,A.; DeBattisti,C.; Capua,I.; Cattoli,G.
EPI305550	HA	Turkey	2006-Jan-08	A/Chicken/Turkey-Ankara/09rs2841-31/2006	Istituto Zooprofilattico Sperimentale Delle Venezie	Istituto Zooprofilattico Sperimentale Delle Venezie	Schivo,A.; Valastro,V.; Monne,I.; Coven,F.; Fusaro,A.; Dakman,A.; Akcadag,B.; Salviato,A.; DeBattisti,C.; Capua,I.; Cattoli,G.
EPI305548	HA	Turkey	2006-Jan-07	A/Chicken/Turkey-Erzurum/09rs2841-25/2006	Istituto Zooprofilattico Sperimentale Delle Venezie	Istituto Zooprofilattico Sperimentale Delle Venezie	Schivo,A.; Valastro,V.; Monne,I.; Coven,F.; Fusaro,A.; Dakman,A.; Akcadag,B.; Salviato,A.; DeBattisti,C.; Capua,I.; Cattoli,G.

EPI305545	HA	Turkey	2006-Jan-04	A/Chicken/Turkey-Erzurum/09rs2841-21/2006	Istituto Zooprofilattico Sperimentale Delle Venezie	Istituto Zooprofilattico Sperimentale Delle Venezie	Schivo,A.; Valastro,V.; Monne,I.; Coven,F.; Fusaro,A.; Dakman,A.; Akcadag,B.; Salviato,A.; DeBattisti,C.; Capua,I.; Cattoli,G.
EPI305542	HA	Turkey	2006-Jan-04	A/Chicken/Turkey-Igdir/09rs2841-14/2006	Istituto Zooprofilattico Sperimentale Delle Venezie	Istituto Zooprofilattico Sperimentale Delle Venezie	Schivo,A.; Valastro,V.; Monne,I.; Coven,F.; Fusaro,A.; Dakman,A.; Akcadag,B.; Salviato,A.; DeBattisti,C.; Capua,I.; Cattoli,G.
EPI293912	HA	Turkey	2006-Jan-23	A/Chicken/Turkey-Isparta/09rs2841-37/2006	Istituto Zooprofilattico Sperimentale Delle Venezie	Istituto Zooprofilattico Sperimentale Delle Venezie	Schivo,A.; Valastro,V.; Monne,I.; Coven,F.; Fusaro,A.; Dakman,A.; Akcadag,B.; Salviato,A.; DeBattisti,C.; Capua,I.; Cattoli,G.
EPI287379	HA	Egypt	2010-Jan-01	A/chicken/Egypt/3982-52/2010	Istituto Zooprofilattico Sperimentale Delle Venezie	Istituto Zooprofilattico Sperimentale Delle Venezie	
EPI287378	HA	Egypt	2010-Jan-01	A/chicken/Egypt/3982-50/2010	Istituto Zooprofilattico Sperimentale Delle Venezie	Istituto Zooprofilattico Sperimentale Delle Venezie	
EPI287377	HA	Egypt	2010-Jan-01	A/chicken/Egypt/3982-44/2010	Istituto Zooprofilattico Sperimentale Delle Venezie	Istituto Zooprofilattico Sperimentale Delle Venezie	
EPI287376	HA	Egypt	2010-Jan-01	A/chicken/Egypt/3982-43/2010	Istituto Zooprofilattico Sperimentale Delle Venezie	Istituto Zooprofilattico Sperimentale Delle Venezie	
EPI287371	HA	Egypt	2010-Mar-07	A/chicken/Egypt/3982-13/2010	Istituto Zooprofilattico Sperimentale Delle Venezie	Istituto Zooprofilattico Sperimentale Delle Venezie	
EPI287369	HA	Egypt	2010-Feb-24	A/chicken/Egypt/3982-9/2010	Istituto Zooprofilattico Sperimentale Delle Venezie	Istituto Zooprofilattico Sperimentale Delle Venezie	
EPI287368	HA	Egypt	2010-Feb-15	A/chicken/Egypt/3982-7/2010	Istituto Zooprofilattico Sperimentale Delle Venezie	Istituto Zooprofilattico Sperimentale Delle Venezie	
EPI287366	HA	Egypt	2010-Jan-15	A/chicken/Egypt/3982-4/2010	Istituto Zooprofilattico Sperimentale Delle Venezie	Istituto Zooprofilattico Sperimentale Delle Venezie	
EPI287364	HA	Egypt	2010-Jan-25	A/chicken/Egypt/3982-2/2010	Istituto Zooprofilattico Sperimentale Delle Venezie	Istituto Zooprofilattico Sperimentale Delle Venezie	
EPI287358	HA	Egypt	2010-Jan-01	A/chicken/Egypt/2095-50/2010	Istituto Zooprofilattico Sperimentale Delle Venezie	Istituto Zooprofilattico Sperimentale Delle Venezie	
EPI287357	HA	Egypt	2010-Jan-01	A/chicken/Egypt/2095-49/2010	Istituto Zooprofilattico Sperimentale Delle Venezie	Istituto Zooprofilattico Sperimentale Delle Venezie	
EPI287356	HA	Egypt	2010-Jan-01	A/chicken/Egypt/2095-46/2010	Istituto Zooprofilattico Sperimentale Delle Venezie	Istituto Zooprofilattico Sperimentale Delle Venezie	
EPI287355	HA	Egypt	2010-Feb-01	A/chicken/Egypt/2095-39/2010	Istituto Zooprofilattico Sperimentale Delle Venezie	Istituto Zooprofilattico Sperimentale Delle Venezie	
EPI287353	HA	Egypt	2010-Jan-01	A/chicken/Egypt/1553-26/2010	Istituto Zooprofilattico Sperimentale Delle Venezie	Istituto Zooprofilattico Sperimentale Delle Venezie	
EPI287350	HA	Egypt	2010-Feb-01	A/chicken/Egypt/1553-6/2010	Istituto Zooprofilattico Sperimentale Delle Venezie	Istituto Zooprofilattico Sperimentale Delle Venezie	
EPI210043	HA	Russian Federation	2006-Mar-01	A/chicken/Krasnodar/199/06	Federal Centre for Animal Health (ARRIAH)	Istituto Zooprofilattico Sperimentale Delle Venezie	
EPI173704	HA	Vietnam	2008-Jan-01	A/chicken/Vietnam/NCVD-016/2008	National Centre of Veterinary Diagnostics	Centers for Disease Control and Prevention	Davis,Todd; Rivailler,Pierre; Nguyen,Tung
EPI160532	HA	Vietnam	2007-Jul-01	A/chicken/Vietnam/NCVD-74/07		Centers for Disease Control and Prevention	
EPI156797	HA	Poland	2007-Dec-04	A/chicken/Poland/38-140V08/2007		National Veterinary Research Institute	
EPI156748	HA	Egypt	2007-Mar-01	A/chicken/Egypt/2628-3/2007		Istituto Zooprofilattico Sperimentale Delle Venezie	
EPI156746	HA	Egypt	2007-Mar-01	A/chicken/Egypt/2628-2/2007		Istituto Zooprofilattico Sperimentale Delle Venezie	
EPI586534	HA	Canada	2014-Dec-12	A/chicken/BC/FAV22/2014	Animal Health Centre, Ministry of Agriculture	Canadian Food Inspection Agency	
EPI579784	HA	Egypt	2015-Jan-04	A/chicken/Egypt/CLEVB-18_N00232/2015	The Central Laboratory for Evaluation of Veterinary Biologics (CLEVB)	NAMRU-3	Younan, M.; Defang, G.; Mohareb, E.; Ali, A.M.; Nassif, S.A.; Mourad,A.A.; Fouad, E.M.; Ragab, A.S.; Khelfa, D.G.
EPI579325	HA	Egypt	2015-Jan-05	A/chicken/Egypt/CLEVB-16_N00230/2014	The Central Laboratory for Evaluation of Veterinary Biologics (CLEVB)	NAMRU-3	Younan, M.; Defang, G.; Mohareb, E.; Ali, A.M.; Nassif, S.A.; Mourad,A.A.; Fouad, E.M.; Ragab, A.S.; Khelfa, D.G.
EPI579324	HA	Egypt	2015-Jan-04	A/chicken/Egypt/CLEVB-19_N00233/2015	The Central Laboratory for Evaluation of Veterinary Biologics (CLEVB)	NAMRU-3	Younan, M.; Defang, G.; Mohareb, E.; Ali, A.M.; Nassif, S.A.; Mourad,A.A.; Fouad, E.M.; Ragab, A.S.; Khelfa, D.G.
EPI573192	HA	Korea, Republic of	2014-Feb-13	A/breeder chicken/Korea/H503/2014	Animal and Plant Quarantine Agency	Animal and Plant Quarantine Agency	
EPI573163	HA	Netherlands	2014-Nov-19	A/chicken/Netherlands/14015766/2014	Central Veterinary Institute	Central Veterinary Institute	Heutink, Rene; Harders, Frank; Verschuren-Pritz, Sylvia; Bossers, Alex; Koch, Guus; Bouwstra, Ruth
EPI552776	HA	Netherlands	2014-Nov-21	A/chicken/Netherlands/emc-3/2014	Erasmus Medical Center	Erasmus Medical Center	Fouchier, Ron A.M.; Bestebroer, Theo; Van den Brand, Judith M.A.; Van der Vliet, Stefan; Verhagen, Josanne H.
EPI548623	HA	Netherlands	2014-Nov-15	A/chicken/Netherlands/14015531/2014	Central Veterinary Institute	Central Veterinary Institute	Heutink, Rene; Harders, Frank; Verschuren-Pritz, Sylvia; Bossers, Alex; Koch, Guus; Bouwstra, Ruth
EPI547678	HA	Netherlands	2014-Nov-14	A/Chicken/Netherlands/14015526/2014	Central Veterinary Institute	Central Veterinary Institute	Heutink, Rene; Harders, Frank; Verschuren-Pritz, Sylvia; Bossers, Alex; Koch, Guus; Bouwstra, Ruth
EPI533461	HA	Indonesia	2009-Jan-01	A/chicken/North Sumatra/27/2009	Dr. Teguh Y. Prajitno	Erasmus Medical Center	Koel,B.F.; van der Vliet,S.; Burke,D.F.; Bestebroer,T.M.; Bharoto,E.E.; Yasa,I.W.; Herliana,I.; Laksono,B.M.; Xu,K.; Skepner,E.; Russell,C.A.; Rimmelzwaan,G.F.; Perez,D.R.; Osterhaus,A.D.; Smith,D.J.; Prajitno,T.Y.; Fouchier,R.A.; Yasa,I.W.W.; Osterhaus,A.D.M.E.; Fouchier,R.A.M.
EPI533460	HA	Indonesia	2010-Jan-01	A/chicken/North Sumatra/72/2010	Dr. Teguh Y. Prajitno	Erasmus Medical Center	Koel,B.F.; van der Vliet,S.; Burke,D.F.; Bestebroer,T.M.; Bharoto,E.E.; Yasa,I.W.; Herliana,I.; Laksono,B.M.; Xu,K.; Skepner,E.; Russell,C.A.; Rimmelzwaan,G.F.; Perez,D.R.; Osterhaus,A.D.; Smith,D.J.; Prajitno,T.Y.; Fouchier,R.A.; Yasa,I.W.W.; Osterhaus,A.D.M.E.; Fouchier,R.A.M.
EPI533458	HA	Indonesia	2011-Jan-01	A/chicken/North Sumatra/198/2011	Dr. Teguh Y. Prajitno	Erasmus Medical Center	Koel,B.F.; van der Vliet,S.; Burke,D.F.; Bestebroer,T.M.; Bharoto,E.E.; Yasa,I.W.; Herliana,I.; Laksono,B.M.; Xu,K.; Skepner,E.; Russell,C.A.; Rimmelzwaan,G.F.; Perez,D.R.; Osterhaus,A.D.; Smith,D.J.; Prajitno,T.Y.; Fouchier,R.A.; Yasa,I.W.W.; Osterhaus,A.D.M.E.; Fouchier,R.A.M.
EPI533457	HA	Indonesia	2011-Jan-01	A/chicken/Jambi/184/2011	Dr. Teguh Y. Prajitno	Erasmus Medical Center	Koel,B.F.; van der Vliet,S.; Burke,D.F.; Bestebroer,T.M.; Bharoto,E.E.; Yasa,I.W.; Herliana,I.; Laksono,B.M.; Xu,K.; Skepner,E.; Russell,C.A.; Rimmelzwaan,G.F.; Perez,D.R.; Osterhaus,A.D.; Smith,D.J.; Prajitno,T.Y.; Fouchier,R.A.; Yasa,I.W.W.; Osterhaus,A.D.M.E.; Fouchier,R.A.M.
EPI533456	HA	Indonesia	2011-Jan-01	A/chicken/South Sumatra/170/2011	Dr. Teguh Y. Prajitno	Erasmus Medical Center	Koel,B.F.; van der Vliet,S.; Burke,D.F.; Bestebroer,T.M.; Bharoto,E.E.; Yasa,I.W.; Herliana,I.; Laksono,B.M.; Xu,K.; Skepner,E.; Russell,C.A.; Rimmelzwaan,G.F.; Perez,D.R.; Osterhaus,A.D.; Smith,D.J.; Prajitno,T.Y.; Fouchier,R.A.; Yasa,I.W.W.; Osterhaus,A.D.M.E.; Fouchier,R.A.M.
EPI533455	HA	Indonesia	2007-Jan-01	A/chicken/Lampung/007/2007	Dr. Teguh Y. Prajitno	Erasmus Medical Center	Koel,B.F.; van der Vliet,S.; Burke,D.F.; Bestebroer,T.M.; Bharoto,E.E.; Yasa,I.W.; Herliana,I.; Laksono,B.M.; Xu,K.; Skepner,E.; Russell,C.A.; Rimmelzwaan,G.F.; Perez,D.R.; Osterhaus,A.D.; Smith,D.J.; Prajitno,T.Y.; Fouchier,R.A.; Yasa,I.W.W.; Osterhaus,A.D.M.E.; Fouchier,R.A.M.
EPI533450	HA	Indonesia	2008-Jan-01	A/chicken/West Java/6-1/2008	Dr. Teguh Y. Prajitno	Erasmus Medical Center	Koel,B.F.; van der Vliet,S.; Burke,D.F.; Bestebroer,T.M.; Bharoto,E.E.; Yasa,I.W.; Herliana,I.; Laksono,B.M.; Xu,K.; Skepner,E.; Russell,C.A.; Rimmelzwaan,G.F.; Perez,D.R.; Osterhaus,A.D.; Smith,D.J.; Prajitno,T.Y.; Fouchier,R.A.; Yasa,I.W.W.; Osterhaus,A.D.M.E.; Fouchier,R.A.M.
EPI533448	HA	Indonesia	2009-Jan-01	A/chicken/West Java/59/2009	Dr. Teguh Y. Prajitno	Erasmus Medical Center	Koel,B.F.; van der Vliet,S.; Burke,D.F.; Bestebroer,T.M.; Bharoto,E.E.; Yasa,I.W.; Herliana,I.; Laksono,B.M.; Xu,K.; Skepner,E.; Russell,C.A.; Rimmelzwaan,G.F.; Perez,D.R.; Osterhaus,A.D.; Smith,D.J.; Prajitno,T.Y.; Fouchier,R.A.; Yasa,I.W.W.; Osterhaus,A.D.M.E.; Fouchier,R.A.M.
EPI533447	HA	Indonesia	2010-Jan-01	A/chicken/West Java/094/2010	Dr. Teguh Y. Prajitno	Erasmus Medical Center	Koel,B.F.; van der Vliet,S.; Burke,D.F.; Bestebroer,T.M.; Bharoto,E.E.; Yasa,I.W.; Herliana,I.; Laksono,B.M.; Xu,K.; Skepner,E.; Russell,C.A.; Rimmelzwaan,G.F.; Perez,D.R.; Osterhaus,A.D.; Smith,D.J.; Prajitno,T.Y.; Fouchier,R.A.; Yasa,I.W.W.; Osterhaus,A.D.M.E.; Fouchier,R.A.M.

EPI533440	HA	Indonesia	2008-Jan-01	A/chicken/West Java/32/031/2008	Dr. Teguh Y. Prajitno	Erasmus Medical Center	Koel,B.F.; van der Vliet,S.; Burke,D.F.; Bestebroer,T.M.; Bharoto,E.E.; Yasa,I.W.; Herliana,I.; Laksono,B.M.; Xu,K.; Skepner,E.; Russell,C.A.; Rimmelzwaan,G.F.; Perez,D.R.; Osterhaus,A.D.; Smith,D.J.; Prajitno,T.Y.; Fouchier,R.A.; Yasa,I.W.W.; Osterhaus,A.D.M.E.; Fouchier,R.A.M.
EPI533439	HA	Indonesia	2008-Jan-01	A/chicken/West Java/34/2008	Dr. Teguh Y. Prajitno	Erasmus Medical Center	Koel,B.F.; van der Vliet,S.; Burke,D.F.; Bestebroer,T.M.; Bharoto,E.E.; Yasa,I.W.; Herliana,I.; Laksono,B.M.; Xu,K.; Skepner,E.; Russell,C.A.; Rimmelzwaan,G.F.; Perez,D.R.; Osterhaus,A.D.; Smith,D.J.; Prajitno,T.Y.; Fouchier,R.A.; Yasa,I.W.W.; Osterhaus,A.D.M.E.; Fouchier,R.A.M.
EPI533434	HA	Indonesia	2010-Jan-01	A/chicken/West Java/091/2010	Dr. Teguh Y. Prajitno	Erasmus Medical Center	Koel,B.F.; van der Vliet,S.; Burke,D.F.; Bestebroer,T.M.; Bharoto,E.E.; Yasa,I.W.; Herliana,I.; Laksono,B.M.; Xu,K.; Skepner,E.; Russell,C.A.; Rimmelzwaan,G.F.; Perez,D.R.; Osterhaus,A.D.; Smith,D.J.; Prajitno,T.Y.; Fouchier,R.A.; Yasa,I.W.W.; Osterhaus,A.D.M.E.; Fouchier,R.A.M.
EPI533433	HA	Indonesia	2010-Jan-01	A/chicken/West Java/119/2010	Dr. Teguh Y. Prajitno	Erasmus Medical Center	Koel,B.F.; van der Vliet,S.; Burke,D.F.; Bestebroer,T.M.; Bharoto,E.E.; Yasa,I.W.; Herliana,I.; Laksono,B.M.; Xu,K.; Skepner,E.; Russell,C.A.; Rimmelzwaan,G.F.; Perez,D.R.; Osterhaus,A.D.; Smith,D.J.; Prajitno,T.Y.; Fouchier,R.A.; Yasa,I.W.W.; Osterhaus,A.D.M.E.; Fouchier,R.A.M.
EPI533432	HA	Indonesia	2010-Jan-01	A/chicken/West Java/120/2010	Dr. Teguh Y. Prajitno	Erasmus Medical Center	Koel,B.F.; van der Vliet,S.; Burke,D.F.; Bestebroer,T.M.; Bharoto,E.E.; Yasa,I.W.; Herliana,I.; Laksono,B.M.; Xu,K.; Skepner,E.; Russell,C.A.; Rimmelzwaan,G.F.; Perez,D.R.; Osterhaus,A.D.; Smith,D.J.; Prajitno,T.Y.; Fouchier,R.A.; Yasa,I.W.W.; Osterhaus,A.D.M.E.; Fouchier,R.A.M.
EPI533431	HA	Indonesia	2010-Jan-01	A/chicken/West Java/134/2010	Dr. Teguh Y. Prajitno	Erasmus Medical Center	Koel,B.F.; van der Vliet,S.; Burke,D.F.; Bestebroer,T.M.; Bharoto,E.E.; Yasa,I.W.; Herliana,I.; Laksono,B.M.; Xu,K.; Skepner,E.; Russell,C.A.; Rimmelzwaan,G.F.; Perez,D.R.; Osterhaus,A.D.; Smith,D.J.; Prajitno,T.Y.; Fouchier,R.A.; Yasa,I.W.W.; Osterhaus,A.D.M.E.; Fouchier,R.A.M.
EPI533427	HA	Indonesia	2011-Jan-01	A/chicken/West Java/200/2011	Dr. Teguh Y. Prajitno	Erasmus Medical Center	Koel,B.F.; van der Vliet,S.; Burke,D.F.; Bestebroer,T.M.; Bharoto,E.E.; Yasa,I.W.; Herliana,I.; Laksono,B.M.; Xu,K.; Skepner,E.; Russell,C.A.; Rimmelzwaan,G.F.; Perez,D.R.; Osterhaus,A.D.; Smith,D.J.; Prajitno,T.Y.; Fouchier,R.A.; Yasa,I.W.W.; Osterhaus,A.D.M.E.; Fouchier,R.A.M.
EPI533426	HA	Indonesia	2006-Jan-01	A/chicken/West Java/028/2006	Dr. Teguh Y. Prajitno	Erasmus Medical Center	Koel,B.F.; van der Vliet,S.; Burke,D.F.; Bestebroer,T.M.; Bharoto,E.E.; Yasa,I.W.; Herliana,I.; Laksono,B.M.; Xu,K.; Skepner,E.; Russell,C.A.; Rimmelzwaan,G.F.; Perez,D.R.; Osterhaus,A.D.; Smith,D.J.; Prajitno,T.Y.; Fouchier,R.A.; Yasa,I.W.W.; Osterhaus,A.D.M.E.; Fouchier,R.A.M.
EPI533425	HA	Indonesia	2008-Jan-01	A/chicken/West Java/028/2008	Dr. Teguh Y. Prajitno	Erasmus Medical Center	Koel,B.F.; van der Vliet,S.; Burke,D.F.; Bestebroer,T.M.; Bharoto,E.E.; Yasa,I.W.; Herliana,I.; Laksono,B.M.; Xu,K.; Skepner,E.; Russell,C.A.; Rimmelzwaan,G.F.; Perez,D.R.; Osterhaus,A.D.; Smith,D.J.; Prajitno,T.Y.; Fouchier,R.A.; Yasa,I.W.W.; Osterhaus,A.D.M.E.; Fouchier,R.A.M.
EPI533422	HA	Indonesia	2010-Jan-01	A/chicken/West Java/093/2010	Dr. Teguh Y. Prajitno	Erasmus Medical Center	Koel,B.F.; van der Vliet,S.; Burke,D.F.; Bestebroer,T.M.; Bharoto,E.E.; Yasa,I.W.; Herliana,I.; Laksono,B.M.; Xu,K.; Skepner,E.; Russell,C.A.; Rimmelzwaan,G.F.; Perez,D.R.; Osterhaus,A.D.; Smith,D.J.; Prajitno,T.Y.; Fouchier,R.A.; Yasa,I.W.W.; Osterhaus,A.D.M.E.; Fouchier,R.A.M.
EPI533421	HA	Indonesia	2010-Jan-01	A/chicken/West Java/125/2010	Dr. Teguh Y. Prajitno	Erasmus Medical Center	Koel,B.F.; van der Vliet,S.; Burke,D.F.; Bestebroer,T.M.; Bharoto,E.E.; Yasa,I.W.; Herliana,I.; Laksono,B.M.; Xu,K.; Skepner,E.; Russell,C.A.; Rimmelzwaan,G.F.; Perez,D.R.; Osterhaus,A.D.; Smith,D.J.; Prajitno,T.Y.; Fouchier,R.A.; Yasa,I.W.W.; Osterhaus,A.D.M.E.; Fouchier,R.A.M.
EPI533420	HA	Indonesia	2007-Jan-01	A/chicken/West Java/129/2007	Dr. Teguh Y. Prajitno	Erasmus Medical Center	Koel,B.F.; van der Vliet,S.; Burke,D.F.; Bestebroer,T.M.; Bharoto,E.E.; Yasa,I.W.; Herliana,I.; Laksono,B.M.; Xu,K.; Skepner,E.; Russell,C.A.; Rimmelzwaan,G.F.; Perez,D.R.; Osterhaus,A.D.; Smith,D.J.; Prajitno,T.Y.; Fouchier,R.A.; Yasa,I.W.W.; Osterhaus,A.D.M.E.; Fouchier,R.A.M.
EPI533419	HA	Indonesia	2007-Jan-01	A/chicken/West Java/130/2007	Dr. Teguh Y. Prajitno	Erasmus Medical Center	Koel,B.F.; van der Vliet,S.; Burke,D.F.; Bestebroer,T.M.; Bharoto,E.E.; Yasa,I.W.; Herliana,I.; Laksono,B.M.; Xu,K.; Skepner,E.; Russell,C.A.; Rimmelzwaan,G.F.; Perez,D.R.; Osterhaus,A.D.; Smith,D.J.; Prajitno,T.Y.; Fouchier,R.A.; Yasa,I.W.W.; Osterhaus,A.D.M.E.; Fouchier,R.A.M.
EPI533418	HA	Indonesia	2007-Jan-01	A/chicken/West Java/132/2007	Dr. Teguh Y. Prajitno	Erasmus Medical Center	Koel,B.F.; van der Vliet,S.; Burke,D.F.; Bestebroer,T.M.; Bharoto,E.E.; Yasa,I.W.; Herliana,I.; Laksono,B.M.; Xu,K.; Skepner,E.; Russell,C.A.; Rimmelzwaan,G.F.; Perez,D.R.; Osterhaus,A.D.; Smith,D.J.; Prajitno,T.Y.; Fouchier,R.A.; Yasa,I.W.W.; Osterhaus,A.D.M.E.; Fouchier,R.A.M.
EPI533414	HA	Indonesia	2007-Jan-01	A/chicken/West Java/20/2007	Dr. Teguh Y. Prajitno	Erasmus Medical Center	Koel,B.F.; van der Vliet,S.; Burke,D.F.; Bestebroer,T.M.; Bharoto,E.E.; Yasa,I.W.; Herliana,I.; Laksono,B.M.; Xu,K.; Skepner,E.; Russell,C.A.; Rimmelzwaan,G.F.; Perez,D.R.; Osterhaus,A.D.; Smith,D.J.; Prajitno,T.Y.; Fouchier,R.A.; Yasa,I.W.W.; Osterhaus,A.D.M.E.; Fouchier,R.A.M.
EPI533412	HA	Indonesia	2009-Jan-01	A/chicken/Banten/019/2009	Dr. Teguh Y. Prajitno	Erasmus Medical Center	Koel,B.F.; van der Vliet,S.; Burke,D.F.; Bestebroer,T.M.; Bharoto,E.E.; Yasa,I.W.; Herliana,I.; Laksono,B.M.; Xu,K.; Skepner,E.; Russell,C.A.; Rimmelzwaan,G.F.; Perez,D.R.; Osterhaus,A.D.; Smith,D.J.; Prajitno,T.Y.; Fouchier,R.A.; Yasa,I.W.W.; Osterhaus,A.D.M.E.; Fouchier,R.A.M.
EPI533411	HA	Indonesia	2009-Jan-01	A/chicken/West Java/128/2009	Dr. Teguh Y. Prajitno	Erasmus Medical Center	Koel,B.F.; van der Vliet,S.; Burke,D.F.; Bestebroer,T.M.; Bharoto,E.E.; Yasa,I.W.; Herliana,I.; Laksono,B.M.; Xu,K.; Skepner,E.; Russell,C.A.; Rimmelzwaan,G.F.; Perez,D.R.; Osterhaus,A.D.; Smith,D.J.; Prajitno,T.Y.; Fouchier,R.A.; Yasa,I.W.W.; Osterhaus,A.D.M.E.; Fouchier,R.A.M.
EPI533406	HA	Indonesia	2007-Jan-01	A/chicken/West Java/30/2007	Dr. Teguh Y. Prajitno	Erasmus Medical Center	Koel,B.F.; van der Vliet,S.; Burke,D.F.; Bestebroer,T.M.; Bharoto,E.E.; Yasa,I.W.; Herliana,I.; Laksono,B.M.; Xu,K.; Skepner,E.; Russell,C.A.; Rimmelzwaan,G.F.; Perez,D.R.; Osterhaus,A.D.; Smith,D.J.; Prajitno,T.Y.; Fouchier,R.A.; Yasa,I.W.W.; Osterhaus,A.D.M.E.; Fouchier,R.A.M.
EPI533397	HA	Indonesia	2009-Jan-01	A/chicken/Central Java/51/2009	Dr. Teguh Y. Prajitno	Erasmus Medical Center	Koel,B.F.; van der Vliet,S.; Burke,D.F.; Bestebroer,T.M.; Bharoto,E.E.; Yasa,I.W.; Herliana,I.; Laksono,B.M.; Xu,K.; Skepner,E.; Russell,C.A.; Rimmelzwaan,G.F.; Perez,D.R.; Osterhaus,A.D.; Smith,D.J.; Prajitno,T.Y.; Fouchier,R.A.; Yasa,I.W.W.; Osterhaus,A.D.M.E.; Fouchier,R.A.M.
EPI533391	HA	Indonesia	2010-Jan-01	A/chicken/Central Java/75/2010	Dr. Teguh Y. Prajitno	Erasmus Medical Center	Koel,B.F.; van der Vliet,S.; Burke,D.F.; Bestebroer,T.M.; Bharoto,E.E.; Yasa,I.W.; Herliana,I.; Laksono,B.M.; Xu,K.; Skepner,E.; Russell,C.A.; Rimmelzwaan,G.F.; Perez,D.R.; Osterhaus,A.D.; Smith,D.J.; Prajitno,T.Y.; Fouchier,R.A.; Yasa,I.W.W.; Osterhaus,A.D.M.E.; Fouchier,R.A.M.
EPI533390	HA	Indonesia	2010-Jan-01	A/chicken/Central Java/075/2010	Dr. Teguh Y. Prajitno	Erasmus Medical Center	Koel,B.F.; van der Vliet,S.; Burke,D.F.; Bestebroer,T.M.; Bharoto,E.E.; Yasa,I.W.; Herliana,I.; Laksono,B.M.; Xu,K.; Skepner,E.; Russell,C.A.; Rimmelzwaan,G.F.; Perez,D.R.; Osterhaus,A.D.; Smith,D.J.; Prajitno,T.Y.; Fouchier,R.A.; Yasa,I.W.W.; Osterhaus,A.D.M.E.; Fouchier,R.A.M.
EPI533389	HA	Indonesia	2010-Jan-01	A/chicken/Central Java/99/2010	Dr. Teguh Y. Prajitno	Erasmus Medical Center	Koel,B.F.; van der Vliet,S.; Burke,D.F.; Bestebroer,T.M.; Bharoto,E.E.; Yasa,I.W.; Herliana,I.; Laksono,B.M.; Xu,K.; Skepner,E.; Russell,C.A.; Rimmelzwaan,G.F.; Perez,D.R.; Osterhaus,A.D.; Smith,D.J.; Prajitno,T.Y.; Fouchier,R.A.; Yasa,I.W.W.; Osterhaus,A.D.M.E.; Fouchier,R.A.M.
EPI533385	HA	Indonesia	2011-Jan-01	A/chicken/Yogyakarta/159/2011	Dr. Teguh Y. Prajitno	Erasmus Medical Center	Koel,B.F.; van der Vliet,S.; Burke,D.F.; Bestebroer,T.M.; Bharoto,E.E.; Yasa,I.W.; Herliana,I.; Laksono,B.M.; Xu,K.; Skepner,E.; Russell,C.A.; Rimmelzwaan,G.F.; Perez,D.R.; Osterhaus,A.D.; Smith,D.J.; Prajitno,T.Y.; Fouchier,R.A.; Yasa,I.W.W.; Osterhaus,A.D.M.E.; Fouchier,R.A.M.
EPI533384	HA	Indonesia	2010-Jan-01	A/chicken/East Java/121/2010	Dr. Teguh Y. Prajitno	Erasmus Medical Center	Koel,B.F.; van der Vliet,S.; Burke,D.F.; Bestebroer,T.M.; Bharoto,E.E.; Yasa,I.W.; Herliana,I.; Laksono,B.M.; Xu,K.; Skepner,E.; Russell,C.A.; Rimmelzwaan,G.F.; Perez,D.R.; Osterhaus,A.D.; Smith,D.J.; Prajitno,T.Y.; Fouchier,R.A.; Yasa,I.W.W.; Osterhaus,A.D.M.E.; Fouchier,R.A.M.
EPI533383	HA	Indonesia	2009-Jan-01	A/chicken/East Java/029/2009	Dr. Teguh Y. Prajitno	Erasmus Medical Center	Koel,B.F.; van der Vliet,S.; Burke,D.F.; Bestebroer,T.M.; Bharoto,E.E.; Yasa,I.W.; Herliana,I.; Laksono,B.M.; Xu,K.; Skepner,E.; Russell,C.A.; Rimmelzwaan,G.F.; Perez,D.R.; Osterhaus,A.D.; Smith,D.J.; Prajitno,T.Y.; Fouchier,R.A.; Yasa,I.W.W.; Osterhaus,A.D.M.E.; Fouchier,R.A.M.
EPI533382	HA	Indonesia	2011-Jan-01	A/chicken/East Java/151/2011	Dr. Teguh Y. Prajitno	Erasmus Medical Center	Koel,B.F.; van der Vliet,S.; Burke,D.F.; Bestebroer,T.M.; Bharoto,E.E.; Yasa,I.W.; Herliana,I.; Laksono,B.M.; Xu,K.; Skepner,E.; Russell,C.A.; Rimmelzwaan,G.F.; Perez,D.R.; Osterhaus,A.D.; Smith,D.J.; Prajitno,T.Y.; Fouchier,R.A.; Yasa,I.W.W.; Osterhaus,A.D.M.E.; Fouchier,R.A.M.
EPI533377	HA	Indonesia	2010-Jan-01	A/chicken/South Sulawesi/140/2010	Dr. Teguh Y. Prajitno	Erasmus Medical Center	Koel,B.F.; van der Vliet,S.; Burke,D.F.; Bestebroer,T.M.; Bharoto,E.E.; Yasa,I.W.; Herliana,I.; Laksono,B.M.; Xu,K.; Skepner,E.; Russell,C.A.; Rimmelzwaan,G.F.; Perez,D.R.; Osterhaus,A.D.; Smith,D.J.; Prajitno,T.Y.; Fouchier,R.A.; Yasa,I.W.W.; Osterhaus,A.D.M.E.; Fouchier,R.A.M.
EPI533375	HA	Indonesia	2011-Jan-01	A/chicken/South Sulawesi/157/2011	Dr. Teguh Y. Prajitno	Erasmus Medical Center	Koel,B.F.; van der Vliet,S.; Burke,D.F.; Bestebroer,T.M.; Bharoto,E.E.; Yasa,I.W.; Herliana,I.; Laksono,B.M.; Xu,K.; Skepner,E.; Russell,C.A.; Rimmelzwaan,G.F.; Perez,D.R.; Osterhaus,A.D.; Smith,D.J.; Prajitno,T.Y.; Fouchier,R.A.; Yasa,I.W.W.; Osterhaus,A.D.M.E.; Fouchier,R.A.M.

EPI533374	HA	Indonesia	2011-Jan-01	A/chicken/South Sulawesi/188/2011	Dr. Teguh Y. Prajitno	Erasmus Medical Center	Koel,B.F.; van der Vliet,S.; Burke,D.F.; Bestebroer,T.M.; Bharoto,E.E.; Yasa,I.W.; Herliana,I.; Laksono,B.M.; Xu,K.; Skepner,E.; Russell,C.A.; Rimmelzwaan,G.F.; Perez,D.R.; Osterhaus,A.D.; Smith,D.J.; Prajitno,T.Y.; Fouchier,R.A.; Yasa,I.W.W.; Osterhaus,A.D.M.E.; Fouchier,R.A.M.
EPI533371	HA	Indonesia	2003-Jan-01	A/chicken/Indonesia/2A/2003	Dr. Teguh Y. Prajitno	Erasmus Medical Center	Koel,B.F.; van der Vliet,S.; Burke,D.F.; Bestebroer,T.M.; Bharoto,E.E.; Yasa,I.W.; Herliana,I.; Laksono,B.M.; Xu,K.; Skepner,E.; Russell,C.A.; Rimmelzwaan,G.F.; Perez,D.R.; Osterhaus,A.D.; Smith,D.J.; Prajitno,T.Y.; Fouchier,R.A.; Yasa,I.W.W.; Osterhaus,A.D.M.E.; Fouchier,R.A.M.
EPI533368	HA	Indonesia	2009-Jan-01	A/chicken/Riau/071/2009	Dr. Teguh Y. Prajitno	Erasmus Medical Center	Koel,B.F.; van der Vliet,S.; Burke,D.F.; Bestebroer,T.M.; Bharoto,E.E.; Yasa,I.W.; Herliana,I.; Laksono,B.M.; Xu,K.; Skepner,E.; Russell,C.A.; Rimmelzwaan,G.F.; Perez,D.R.; Osterhaus,A.D.; Smith,D.J.; Prajitno,T.Y.; Fouchier,R.A.; Yasa,I.W.W.; Osterhaus,A.D.M.E.; Fouchier,R.A.M.
EPI623563	HA	United Arab Emirates	2014-Dec-01	A/Duck/Dubai/2459/2014		Friedrich-Loeffler-Institut	Chen, H.; Chan, KH. ; Wong, PC. ; Woo, C.Y.P.
EPI573335	HA	Egypt	2014-Nov-04	A/duck/Egypt/14227FAOS/2014		Friedrich-Loeffler-Institut	Arafa,A.M; Hagag, N; Elhusseiny, M.H; Yehia, N; Selim, A.A; Abdelhalim, A; Kilany, W.H; Samy, A; Hassan, M.K; Abdelwhab,E.M; Beer,M; Naguib, M.M; Harder,TC
EPI573316	HA	Egypt	2014-Dec-14	A/duck/Egypt/1427SL/2014		Friedrich-Loeffler-Institut	Arafa,A.M; Hagag, N; Elhusseiny, M.H; Yehia, N; Selim, A.A; Abdelhalim, A; Kilany, W.H; Samy, A; Hassan, M.K; Abdelwhab,E.M; Beer,M; Naguib, M.M; Harder,TC
EPI573241	HA	Korea, Republic of	2014-Feb-17	A/breeder duck/Korea/H566/2014		Animal and Plant Quarantine Agency	
EPI573240	HA	Korea, Republic of	2014-Feb-02	A/breeder duck/Korea/H345/2014		Animal and Plant Quarantine Agency	
EPI573230	HA	Korea, Republic of	2014-Oct-27	A/broiler duck/Korea/H1803/2014		Animal and Plant Quarantine Agency	
EPI573229	HA	Korea, Republic of	2014-Oct-11	A/broiler duck/Korea/H1763/2014		Animal and Plant Quarantine Agency	
EPI573224	HA	Korea, Republic of	2014-Sep-25	A/broiler duck/Korea/H1739/2014		Animal and Plant Quarantine Agency	
EPI573222	HA	Korea, Republic of	2014-Sep-25	A/broiler duck/Korea/H1733/2014		Animal and Plant Quarantine Agency	
EPI573217	HA	Korea, Republic of	2014-Jul-28	A/broiler duck/Korea/H1685/2014		Animal and Plant Quarantine Agency	
EPI573216	HA	Korea, Republic of	2014-Jul-25	A/broiler duck/Korea/H1683/2014		Animal and Plant Quarantine Agency	
EPI573213	HA	Korea, Republic of	2014-Jun-16	A/broiler duck/Korea/H1556/2014		Animal and Plant Quarantine Agency	
EPI573210	HA	Korea, Republic of	2014-May-20	A/broiler duck/Korea/H1414/2014		Animal and Plant Quarantine Agency	
EPI573196	HA	Korea, Republic of	2014-Feb-20	A/broiler duck/Korea/H651/2014		Animal and Plant Quarantine Agency	
EPI553308	HA	China	2009-Sep-01	A/Duck/Shandong/Y01/2009	Qingdao Agricultural University	Beijing Institute of Microbiology and Epidemiology	yanbo,yin;dongdong,wang;linlin,liu;xiliang,wang;penghui,yang;xin,liu;keyu,wang;chengcai,lai
EPI548493	HA	Japan	2014-Nov-18	A/duck/Chiba/26-372-61/2014	National Institute of Animal Health	National Institute of Animal Health	
EPI548485	HA	Japan	2014-Nov-18	A/duck/Chiba/26-372-48/2014	National Institute of Animal Health	National Institute of Animal Health	
EPI547673	HA	United Kingdom	2014-Nov-14	A/duck/England/36254/14	Animal and Plant Health Agency (APHA)	Animal and Plant Health Agency (APHA)	Hanna, Amanda; Ellis, Richard; Ceeraz, Vanessa; Seekings, James; Londt, Brandon; Brookes, Sharon; Banks, Jill; Essen, Stephen; Brown, Ian
EPI543002	HA	China	2014-Jan-20	A/duck/Beijing/FS01/2014	Institute of Microbiology, Chinese Academy of Sciences	Institute of Microbiology	
EPI530054	HA	China	2014-Jan-10	A/duck/Jiangxi/95/2014		BGI Shenzhen	Bing,Xu;Tao,Zhang;Xiaowen,Li
EPI485594	HA	Cambodia	2013-Feb-04	A/duck/Cambodia/202W6M1/2013	Institut Pasteur in Cambodia	Institut Pasteur in Cambodia	Rith, S.; Horm, SV.; Buchy, P.
EPI330995	HA	Vietnam	2011-Jan-01	A/duck/Vietnam/NCVD-672/2011	National Centre of Veterinary Diagnostics	Centers for Disease Control and Prevention	Davis,Todd; Rivailler,Pierre; Nguyen,Tung
EPI305551	HA	Turkey	2006-Jan-12	A/Duck/Turkey-Aydin/09rs2841-34/2006	Istituto Zooprofilattico Sperimentale Delle Venezie	Istituto Zooprofilattico Sperimentale Delle Venezie	Schivo,A.; Valastro,V.; Monne,I.; Coven,F.; Fusaro,A.; Dakman,A.; Akcadag,B.; Salviato,A.; DeBattisti,C.; Capua,I.; Cattoli,G.
EPI305544	HA	Turkey	2005-Dec-26	A/Duck/Turkey-Igdir/09rs2841-20/2005	Istituto Zooprofilattico Sperimentale Delle Venezie	Istituto Zooprofilattico Sperimentale Delle Venezie	Schivo,A.; Valastro,V.; Monne,I.; Coven,F.; Fusaro,A.; Dakman,A.; Akcadag,B.; Salviato,A.; DeBattisti,C.; Capua,I.; Cattoli,G.
EPI287370	HA	Egypt	2010-Apr-13	A/duck/Egypt/3982-12/2010	Istituto Zooprofilattico Sperimentale Delle Venezie	Istituto Zooprofilattico Sperimentale Delle Venezie	
EPI169306	HA	France	2006-Mar-30	A/duck/France/06436/2006		AFSSA	
EPI164197	HA	Vietnam	2007-Jul-01	A/muscovy duck/Vietnam/NCVD-67/2007		Centers for Disease Control and Prevention	
EPI164189	HA	Vietnam	2007-Jul-01	A/muscovy duck/Vietnam/NCVD-66/2007		Centers for Disease Control and Prevention	
EPI164181	HA	Vietnam	2007-Jul-01	A/muscovy duck/Vietnam/NCVD-52/2007		Centers for Disease Control and Prevention	
EPI164173	HA	Vietnam	2007-Jul-01	A/muscovy duck/Vietnam/NCVD-51/2007		Centers for Disease Control and Prevention	
EPI161702	HA	Nigeria	2008-Jan-01	A/duck/Nigeria/3724-2/2008		Istituto Zooprofilattico Sperimentale Delle Venezie	
EPI160548	HA	Vietnam	2007-Jul-01	A/duck/Vietnam/NCVD-81/07		Centers for Disease Control and Prevention	
EPI160540	HA	Vietnam	2007-Jul-01	A/duck/Vietnam/NCVD-76/07		Centers for Disease Control and Prevention	
EPI160524	HA	Vietnam	2007-Jul-01	A/duck/Vietnam/NCVD-71/07		Centers for Disease Control and Prevention	
EPI160484	HA	Vietnam	2007-Jul-01	A/duck/Vietnam/NCVD-63/07		Centers for Disease Control and Prevention	
EPI160468	HA	Vietnam	2007-Jul-01	A/duck/Vietnam/NCVD-61/07		Centers for Disease Control and Prevention	
EPI160452	HA	Vietnam	2007-Jul-01	A/duck/Vietnam/NCVD-59/07		Centers for Disease Control and Prevention	
EPI160444	HA	Vietnam	2007-Jul-01	A/duck/Vietnam/NCVD-58/07		Centers for Disease Control and Prevention	
EPI160396	HA	Vietnam	2007-Jul-01	A/duck/Vietnam/NCVD-50/07		Centers for Disease Control and Prevention	
EPI160388	HA	Vietnam	2007-Jul-01	A/duck/Vietnam/NCVD-49/07		Centers for Disease Control and Prevention	
EPI160380	HA	Vietnam	2007-Jul-01	A/duck/Vietnam/NCVD-48/07		Centers for Disease Control and Prevention	
EPI154509	HA	Saudi Arabia	2007-Jan-01	A/duck/Saudi Arabia/6732-7/2007		Istituto Zooprofilattico Sperimentale Delle Venezie	
EPI568474	HA	Korea, Republic of	2015-Jan-22	A/green-winged teal/Korea/KU-12/2015	Avian diaseases laboratory, College of Veterinary Medicine, Konkuk University	Konkuk University	Jung-Hoon,Kwon; Seong-Su,Yuk; Erdene-Ochir,TO; Jin-Yong,Noh; Jae-Keun,Park; Dong-Hun,Lee; Chang-Seon,Song
EPI585111	HA	Netherlands	2015-Feb-25	A/eurasian wigeon/Netherlands/1/2015	Erasmus Medical Center	Erasmus Medical Center	Verhagen, Josanne; Vuong, Oanh; Van der Jeugd, Henk; Nolet, Bart; Fouchier, Ron
EPI552760	HA	Netherlands	2014-Nov-24	A/eurasian wigeon/Netherlands/emc-1/2014	Erasmus Medical Center	Erasmus Medical Center	Fouchier, Ron A.M.; Verhagen, Josanne H.; Vuong, Oanh; Bestebroer, Theo; Van Vliet, Stefan; Van der Jeugd, Henk
EPI595079	HA	Korea, Republic of	2015-Feb-06	A/mallard/Korea/N15-99/2015	Avian diaseases laboratory, College of Veterinary Medicine, Konkuk University	Konkuk University	Kwon,Jung-Hoon; Yuk,Seong-Su; Erdene-Ochir,TO; Noh,Jin-Yong; Hong,Woo-Tack; Jeong,Jei-Hyun; Jeong,Sol; Song,Chang-Seon
EPI595066	HA	Korea, Republic of	2015-Jan-29	A/mallard/Korea/KU3-2/2015	Avian diaseases laboratory, College of Veterinary Medicine, Konkuk University	Konkuk University	Kwon,Jung-Hoon; Yuk,Seong-Su; Erdene-Ochir,TO; Noh,Jin-Yong; Hong,Woo-Tack; Jeong,Jei-Hyun; Jeong,Sol; Song,Chang-Seon
EPI573238	HA	Korea, Republic of	2014-Dec-19	A/mallard/Korea/H2003/2014		Animal and Plant Quarantine Agency	
EPI210108	HA	Italy	2007-Jan-01	A/mallard/Italy/5582-31/2007	Istituto Zooprofilattico Sperimentale Delle Venezie	Istituto Zooprofilattico Sperimentale Delle Venezie	
EPI169275	HA	France	2006-Nov-22	A/duck/France/061054/2006		AFSSA	
EPI573236	HA	Korea, Republic of	2014-Dec-16	A/spot-billed duck/Korea/H1981/2014		Animal and Plant Quarantine Agency	
EPI169513	HA	Saudi Arabia	2007-Jan-01	A/duck/Saudi Arabia/3489-61VIR08/2007		Istituto Zooprofilattico Sperimentale Delle Venezie	
EPI573208	HA	Korea, Republic of	2014-May-08	A/gadwall/Korea/H1351/2014		Animal and Plant Quarantine Agency	
EPI586558	HA	Canada	2014-Dec-19	A/chicken/BC/FAV25/2014	Animal Health Centre, Ministry of Agriculture	Canadian Food Inspection Agency	
EPI462793	HA	Austria	2013-Jun-07	A/mynah/Austria-quarantine/13063485-026/13	Institute for Veterinary Disease Control Moedling, Austrian Agency for Health and Food Safety	Animal and Plant Health Agency (APHA)	Collins, S; Hanna, A; Essen, S; Focosi-Snyman, R; Manvell, R; Wodak, E; Revilla-Fern?ndez, S; Bag?, Z; Schmoll, F; Reid, S
EPI595082	HA	Korea, Republic of	2014-Dec-24	A/mandarin duck/Korea/K14-363-1/2014	Avian diaseases laboratory, College of Veterinary Medicine, Konkuk University	Konkuk University	Kwon,Jung-Hoon; Yuk,Seong-Su; Erdene-Ochir,TO; Noh,Jin-Yong; Hong,Woo-Tack; Jeong,Jei-Hyun; Jeong,Sol; Song,Chang-Seon
